# Redox-Switchable Halogen Bonding in Haloanthracene Mediators Enables Efficient Electrocatalytic C–N Coupling

**DOI:** 10.1021/jacs.5c18175

**Published:** 2026-01-08

**Authors:** Atsuki Hirama, Kayo Suda, Shohei Yoshinaga, Moto Kikuchi, Su-Gi Chong, Azusa Kikuchi, Yusuke Ishigaki, Daisuke Yokogawa, Mahito Atobe, Naoki Shida

**Affiliations:** † Department of Chemistry and Life Science, 13154 Yokohama National University, 79-5 Tokiwadai, Hodogaya-ku, Yokohama 240-8501, Japan; ‡ Graduate School of Arts and Sciences, 13143 The University of Tokyo, Komaba, Meguro-ku, Tokyo 153-8902, Japan; § Department of Chemistry, Faculty of Science, 12810 Hokkaido University, Sapporo 060-0810, Japan; ∥ Institute of Advanced Sciences, Yokohama National University, 79-5 Tokiwadai, Hodogaya-ku, Yokohama 240-8501, Japan; ⊥ PRESTO, Japan Science and Technology Agency (JST), 4-1-8 Honcho, Kawaguchi, Saitama 332-0012, Japan

## Abstract

We report the development of redox mediators based on 9-halo-10-arylanthracenes that engage in halogen bonding only upon one-electron oxidation. This redox-switchable interaction enables an effective substrate preorganization and promotes intramolecular C–N bond formation via electrocatalysis. Systematic evaluation of halogenated mediators (**1a–1c**) across various *N*-protected 2-aminobiphenyl substrates revealed that the iodoanthracene derivative **1a** exhibited superior catalytic performance. Building on this, we synthesized a series of 10-aryl-substituted iodoanthracenes (**1d–1h**) to further optimize the mediator structure. Kinetic analysis by foot-of-the-wave analysis identified **1h**, bearing a 3,5-bis­(trifluoromethyl)­phenyl group, as a highly active mediator with an apparent rate constant over an order of magnitude higher than that of its counterparts. Bulk electrolysis experiments confirmed its remarkable performance, achieving high yields in short reaction times. Computational studies demonstrated that halogen bonding is markedly strengthened in the radical cation state, and that this interaction significantly enhances the acidity of the N–H bond in the substrates, enabling proton-coupled electron transfer (PCET) even with a weak base. Energy diagrams constructed from density functional theory calculations supported a mechanism in which the mediator not only facilitates PCET but also stabilizes cationic intermediates throughout the catalytic cycle. This work establishes a new design paradigm for redox mediators, where redox-induced noncovalent interactions can be harnessed to control both reactivity and selectivity. The concept of halogen-bonding-assisted PCET provides a powerful platform for advancing molecular electrocatalysis.

## Graphical abstract

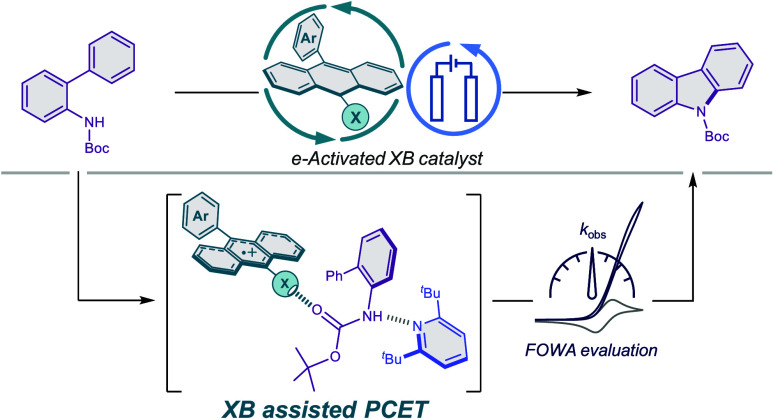

## Introduction

Redox mediators are essential components in electrocatalysis, facilitating electron transfer between electrodes and substrates while often enabling reactions under milder and more selective conditions.^1,2^ Their role becomes particularly critical in organic electrosynthesis, where direct electrolysis can lead to undesired side reactions due to high overpotentials, electrode fouling, or limited control over molecular recognition.^1–5^ Over the past decades, a wide variety of redox mediators, including ferrocene, triarylamines, and nitroxyl radicals, have been developed to overcome these limitations (Figure 1a).^1,6–8^ However, these systems predominantly rely on either outer-sphere electron transfer or the formation of covalent intermediates, which often lack dynamic, substrate-specific interaction capabilities.

**Figure 1:**
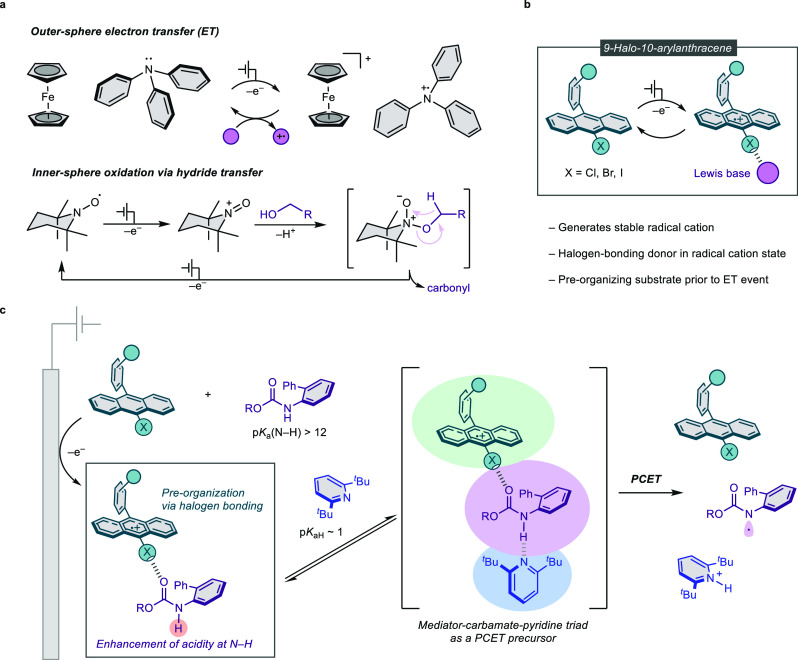
Conceptual overview of this work. (a) Comparison of redox mediators by mode of substrate interaction and electron transfer. (b) Design of redox-switchable haloanthracene mediators enabling halogen bonding upon oxidation. (c) Mechanistic proposal involving halogen-bonding-induced preorganization and PCET.

In contrast, noncovalent interactions, such as hydrogen bonding and π–π stacking, offer a means of transient substrate association, but their integration into redox mediator design remains underexplored. Among them, halogen bonding—a directional, tunable interaction involving polarized halogen atoms—has recently emerged as a powerful tool in molecular recognition and catalysis.^9–13^ Yet, its application in the context of redox catalysis, especially as a redox-switchable interaction, has not been realized. Although several redox-active scaffolds, such as ferrocene and tetrathiafulvalene derivatives, are known to exhibit switchable halogen-bonding behavior upon electrochemical oxidation, these systems have not been utilized as mediators to control electron or proton transfer in catalytic transformations.^14–17^

Herein, we report a new class of redox mediators based on haloanthracene derivatives, which exhibit stronger halogen bonding upon one-electron oxidation to stable radical cation states (Figure 1b). This redox-triggered halogen bonding allows for dynamic substrate capture and spatial preorganization, which, in turn, promotes efficient intramolecular C–N bond formation via electrocatalysis (Figure 1c). Quantitative kinetic evaluation using foot-of-the-wave analysis (FOWA),^18,19^ spectroscopic and structural characterization of the radical cations, and theoretical studies provide a comprehensive mechanistic understanding of this system.^20,21^ This work presents a new strategy in redox mediator design where redox-switchable noncovalent interactions are leveraged to control molecular proximity, electron transfer, and reaction pathways.

## Results and Discussion

### Design and Synthesis of Haloanthracene Mediators

We aimed to develop a mediator capable of electrochemically switchable halogen bonding, enabling a controllable substrate association under anodic conditions. Such a mediator requires a molecular framework that becomes sufficiently electron-deficient upon one-electron oxidation, thereby increasing the electrophilicity of the adjacent halogen atom. Stabilization of the oxidized electron-deficient state is crucial, as the enhanced σ-hole on the halogen must persist long enough to mediate substrate activation while avoiding undesirable side reactions. To meet these requirements, we focused on scaffolds capable of delocalizing the unpaired electron and the cation charge through an extended π-conjugation.

Less-conjugated aromatic systems such as benzene and naphthalene were expected to form unstable radical cations, whereas excessive π-extension was anticipated to lower the oxidation potential, making subsequent one-electron transfer from the substrate difficult. Based on these considerations, anthracene, phenanthrene, and pyrene, which possess comparable degrees of ring fusion, were evaluated as candidate frameworks. Cyclic voltammetry (CV) studies revealed that the anthracene framework provides the most favorable balance between radical cation stability and oxidation potential, establishing it as the optimal platform for constructing haloanthracene-based redox mediators (Figure S4).

Guided by this design, we synthesized a series of haloanthracene-based mediators bearing different halogen substituents (I, Br, Cl) at the 9-position (Figure 2a). The anthracene core was functionalized with a bulky mesityl group at the 10-position to enhance solubility and sterically protect the radical cation state. 9-Halo-10-mesitylanthracenes were obtained in three variants—iodo (**1a**), bromo (**1b**), and chloro (**1c**)—via electrophilic halogenation of a common 10-mesitylanthracene precursor under mild conditions (see Supporting Information for details). Their structures were confirmed by NMR and HRMS analyses.

**Figure 2:**
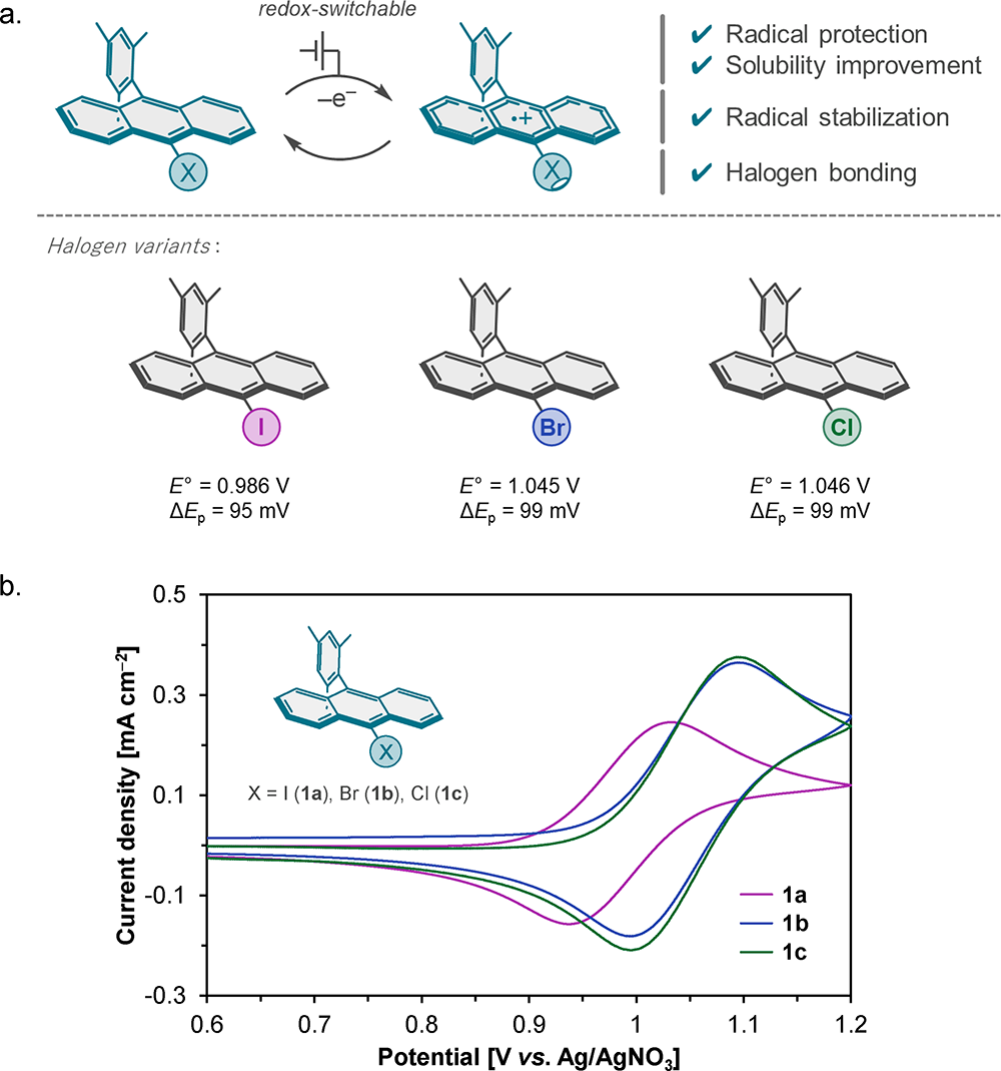
(a) The designs of haloanthracene-based redox mediators and their redox potentials and peak separations. (b) Redox behavior of **1a**–**1c**. Cyclic voltammograms of 1 mM **1a**–**1c** in 0.1 M LiOTf/MeCN + CH_2_Cl_2_ (6:4 in vol) at a scan rate of 0.1 V s^–1^.

CV measurements showed quasi-reversible oxidation waves for all three mediators, corresponding to the formation of their radical cation states (**1a** ^**•+**^–**1c** ^**•+**^) (Figure 2b). The formal oxidation potentials (*E*°) showed only modest variation: 0.99 V vs Ag/AgNO_3_ for **1a** and slightly higher values around 1.05 V for **1b** and **1c**. These results indicate that the halogen substituent exerts only a minor influence on redox potential, while all three mediators exhibit sufficient electrochemical reversibility (Δ*E*_p_ = 95–99 mV) and stability to operate under the electrocatalytic conditions explored in this study.^22^

### Application to Intramolecular C–N Bond Formation

To evaluate the catalytic performance of the haloanthracene mediators, we applied them to the intramolecular oxidative cyclization of a series of *N*-protected 2-aminobiphenyl derivatives. The reaction conditions were systematically optimized, and the full set of screening data and discussion of their implications are provided in the Supporting Information (Section 3–5). Four substrates with different nitrogen protecting groups were examined: acetyl (Ac: **2a**), methoxycarbonyl (Moc: **2b**), *tert*-butoxycarbonyl (Boc: **2c**), and tosyl (Ts: **2d**). These substrates undergo two-electron oxidation to furnish the corresponding carbazole products (**3a–3d**).^20,21^ The reaction proceeded successfully with several aminobiphenyl derivatives bearing diverse substituents, suggesting that the catalytic system can accommodate various functional groups (see Supporting Information, Section 2–19).

Electrolysis was conducted in an undivided cell equipped with platinum electrodes and a Ag/AgNO_3_ reference electrode. Reactions were performed under a constant potential of 1.2 V vs Ag/AgNO_3_ in MeCN/CH_2_Cl_2_ with 0.1 M lithium triflate (LiOTf) as the supporting electrolyte. Each experiment contained 5 mM substrate, 1 mM mediator, and 4 mM 2,6-di-*tert*-butylpyridine (^*t*^Bu_2_Py) as the base. Electrolysis was performed at 50 °C until 5 F mol^–1^ of charge was passed.

The catalytic performance of iodoanthracene mediator **1a** was first evaluated across the four substrates (Figure 3). Efficient C–N bond formation was observed in all cases, with yields of 52% for **2a** (Ac), 60% for **2b** (Moc), 82% for **2c** (Boc), and 14% for **2d** (Ts). The highest efficiency was seen with the Boc-protected substrate, while the Ts group significantly suppressed the reactivity. The lower reactivity is attributed to its strong electron-withdrawing nature and poor charge delocalization, resulting in insufficient intermediate stabilization.^23^

**Figure 3:**
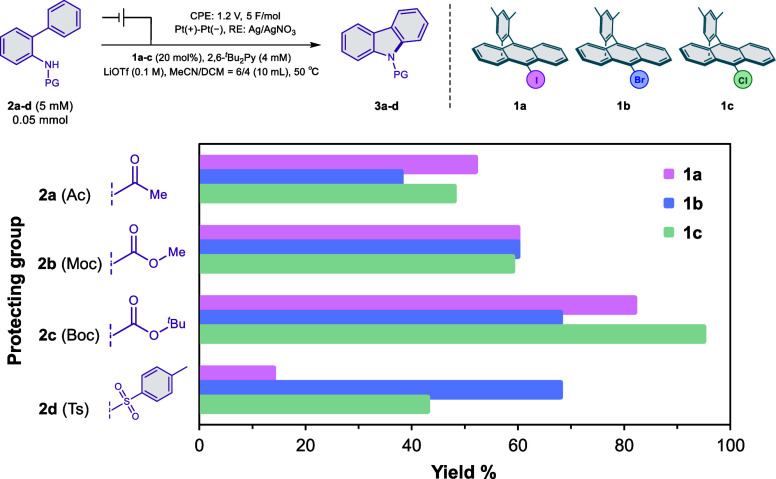
Intramolecular C–N bond formation reaction of various *N*-protected aminobiphenyls.

For comparison, bromo (**1b**) and chloro (**1c**) mediators were also tested. In most cases, **1a** delivered product yields that were comparable to or higher than those obtained with **1b** and **1c**. For example, with **2a** (Ac) and **2b** (Moc), **1a** afforded the highest yields (52% and 60%, respectively). In the case of **2c** (Boc), although **1c** gave the highest yield (95%), **1a** also performed well (82%). For **2d** (Ts), **1a** gave a lower yield (14%) compared to **1b** and **1c**. These results suggest that **1a** shows a particularly favorable performance with moderately electron-withdrawing protecting groups such as Boc and Moc. This trend hints at potential noncovalent interactions, such as redox-induced halogen bonding, that may enhance reactivity under specific substrate electronic environments.

### Quantitative Kinetic Evaluation via Foot-of-the-Wave Analysis (FOWA)

To gain deeper insights into the reactivity trends observed in electrocatalytic C–N bond formation, we conducted FOWA to determine apparent rate constants (*k*_obs_) for each mediator–substrate combination. FOWA is a widely used electrochemical method that enables kinetic analysis of catalytic systems by extracting rate constants from the rising portion (“foot”) of a catalytic wave in cyclic voltammograms.^24–28^ It offers the advantage of requiring only minimal catalytic current, allowing reliable comparison under steady-state conditions even in the presence of diffusion limitations.^29^

The results are summarized in Figure 4. **1a** (X = I) consistently exhibited higher *k*_obs_ values across almost all of the substrates, indicating its superior ability to facilitate electron transfer under catalytic conditions. For instance, with **2c** (Boc), **1a** achieved the highest rate constant of 8.4 × 10^2^ M^–1^ s^–1^, followed by 6.8 × 10^2^ M^–1^ s^–1^ with **2b** (Moc) and 3.9 × 10^2^ M^–1^ s^–1^ with **2a** (Ac). Even for the more challenging Ts-substituted substrate **2d**, **1a** maintained a moderate *k*_obs_ of 5.1 × 10^2^ M^–1^ s^–1^. In contrast, bromo-substituted mediator **1b** displayed higher overall rate constants. The chloro variant **1c** showed a similar or slightly lower trend. These observations underscore the kinetic advantage of the iodo-substituted system, which is attributed to its high polarizability and redox-stable radical cation state that likely enhances substrate preorganization via halogen bonding.^30^ Among the protecting groups examined, the Boc group (**2c**) consistently exhibited the most favorable reactivity. Moreover, a clear positive correlation between the rate constant (*k*_obs_) and the product yield was observed across all substrates (**2a**–**2d**), indicating that the kinetic efficiency of the mediators directly reflects their effectiveness in promoting product formation.

**Figure 4:**
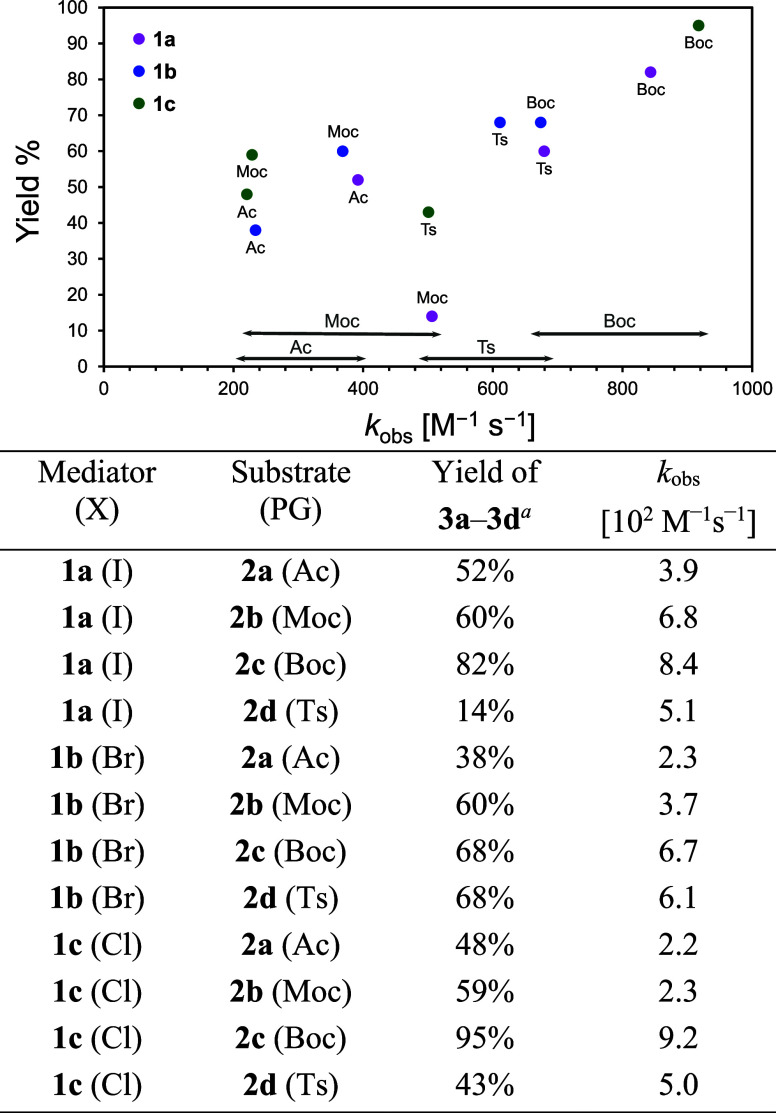
Yield vs *k*_obs_ plot (top) and table (bottom) of intramolecular C–N bond formation reaction using haloanthracene mediators **1a**–**1c**.

### Spectroscopic and Structural Characterization of Radical Cation Intermediates and Their Interaction with a Lewis Base

CV studies of **1a**–**1c** revealed reversible oxidation waves, indicating that radical cation species (**1a** ^**•+**^–**1c** ^**•+**^) can be stable enough to be characterized. To confirm the generation of these intermediates under catalytic conditions, EPR spectroscopy was performed in the same electrolyte solution used for electrocatalysis (Figure 5a). In all cases, characteristic EPR signals attributable to radical species were observed, consistent with the formation of persistent π-radical cations.

**Figure 5:**
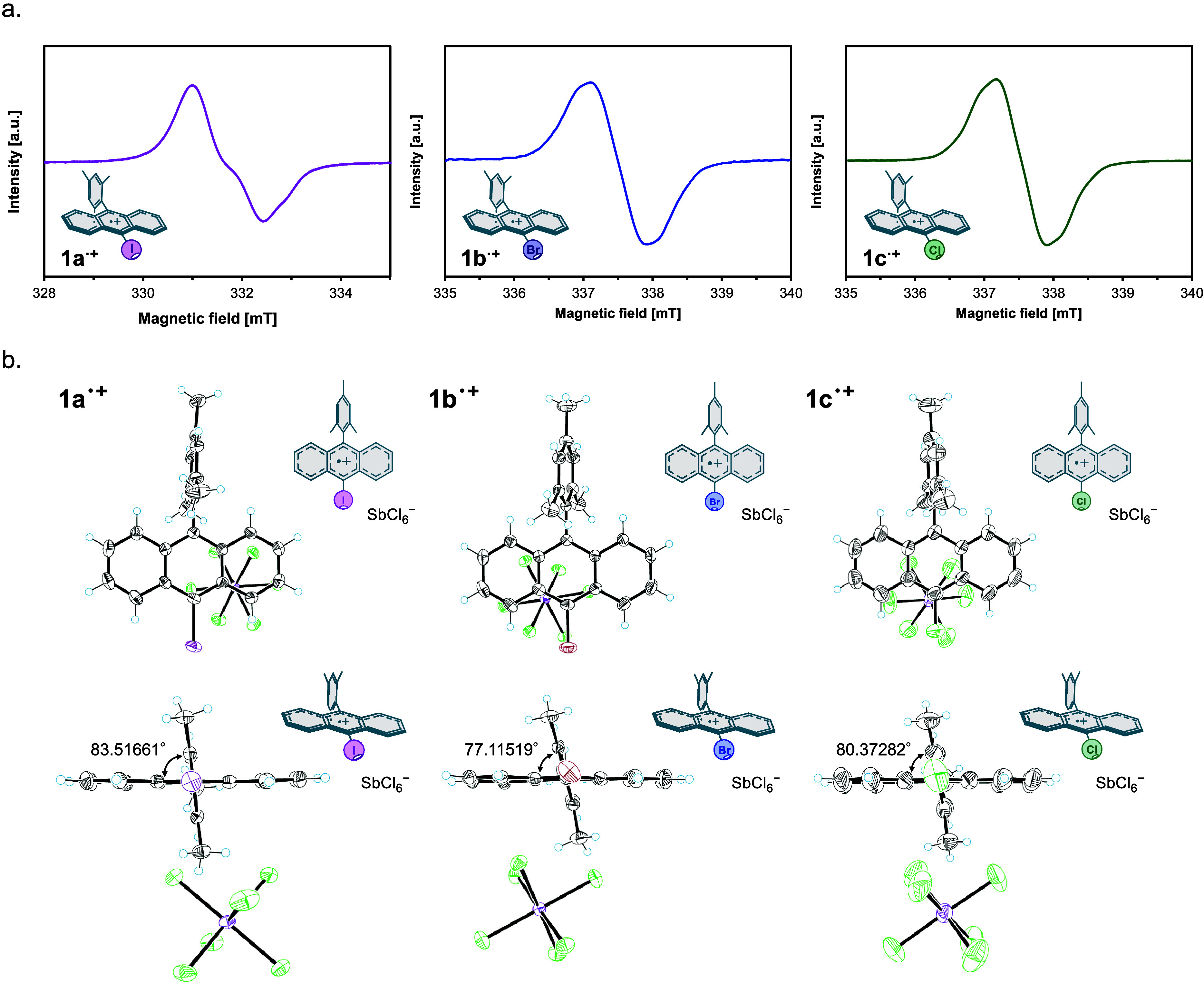
(a) EPR spectrum of **1a** ^**•+**^ (top left) and **1b** ^**•+**^ (top middle) and **1c** ^**•+**^ (top right). (b) X-ray structures of the SbCl_6_^–^ salts of **1a** ^**•+**^ and **1b** ^**•+**^ at 150 K and **1c** ^**•+**^ at 200 K. The thermal ellipsoids are shown in 50% probability and the solvent molecules (CH_2_Cl_2_ and Et_2_O) are omitted for clarity.

We further succeeded in the chemical oxidation of **1a**–**1c** using tris­(2,4-dibromophenyl)­ammoniumyl hexachloroantimonate (magic green) as a one-electron oxidant. The resulting radical cation salts were purified and subjected to slow crystallization at −20 °C, which allowed us to isolate single crystals of all three radical cations (**1a** ^•+^–**1c** ^•+^). To the best of our knowledge, this represents the first crystallographic characterization of haloanthracene radical cations.

The solid-state structures revealed that the anthracene π-framework remains largely planar upon oxidation with only minor changes in bond lengths and angles across the three systems. This structural rigidity indicates that the π-radical is well delocalized and structurally stable, supporting its suitability as a one-electron redox mediator. The mesityl substituent was found to adopt a nearly orthogonal orientation relative to the anthracene core, sterically shielding the π-surface and likely contributing to the stability of the radical cations.

Qualitative observations during crystallization and handling revealed that differences in chemical stability were as follows. **1a** (I) exhibited the highest persistence under ambient conditions, while **1c** (Cl) degraded most rapidly, suggesting that the heavier halogen imparts greater oxidative stability.

We have also performed voltammetric analysis to clarify the presence of halogen-bonding interactions between **1a** and Lewis base (see Supporting Information, Section 3.4 for detail). Square wave voltammetry of **1a** ^**•+**^ was recorded with successive addition of a halogen-bond acceptor, ClO_4_^–^ anion, leading to systematic shifts in the oxidation potential. This result indicates that a more Lewis basic substrate such as **2a** can readily interact with **1a** ^**•+**^. In addition, the yield of **3a** was significantly decreased when nonhalogen-bonding analogues, 9-methyl-10-mesitylanthracene (**1i**) and 9-methyl-10-(3,5-bis­(trifluoromethyl)­phenyl)­anthracene (**1j**), were used as mediators (see Supporting Information, Section 3.6).

### Structural Diversification of Iodoanthracene Mediators

Encouraged by the promising results of bulk electrolysis, kinetic analysis, and structural characterization, we further explored the structural tunability of the iodoanthracene scaffold. To this end, we synthesized a series of derivatives in which the 10-position mesityl group of the parent mediator **1a** was replaced with other aryl groups to modulate the electronic and steric environment (Figure 6a). Specifically, we prepared five new derivatives, **1d**–**1h**. These compounds maintain the core 9-iodoanthracene structure and allow systematic evaluation of aryl substituent effects on mediator performance.

**Figure 6:**
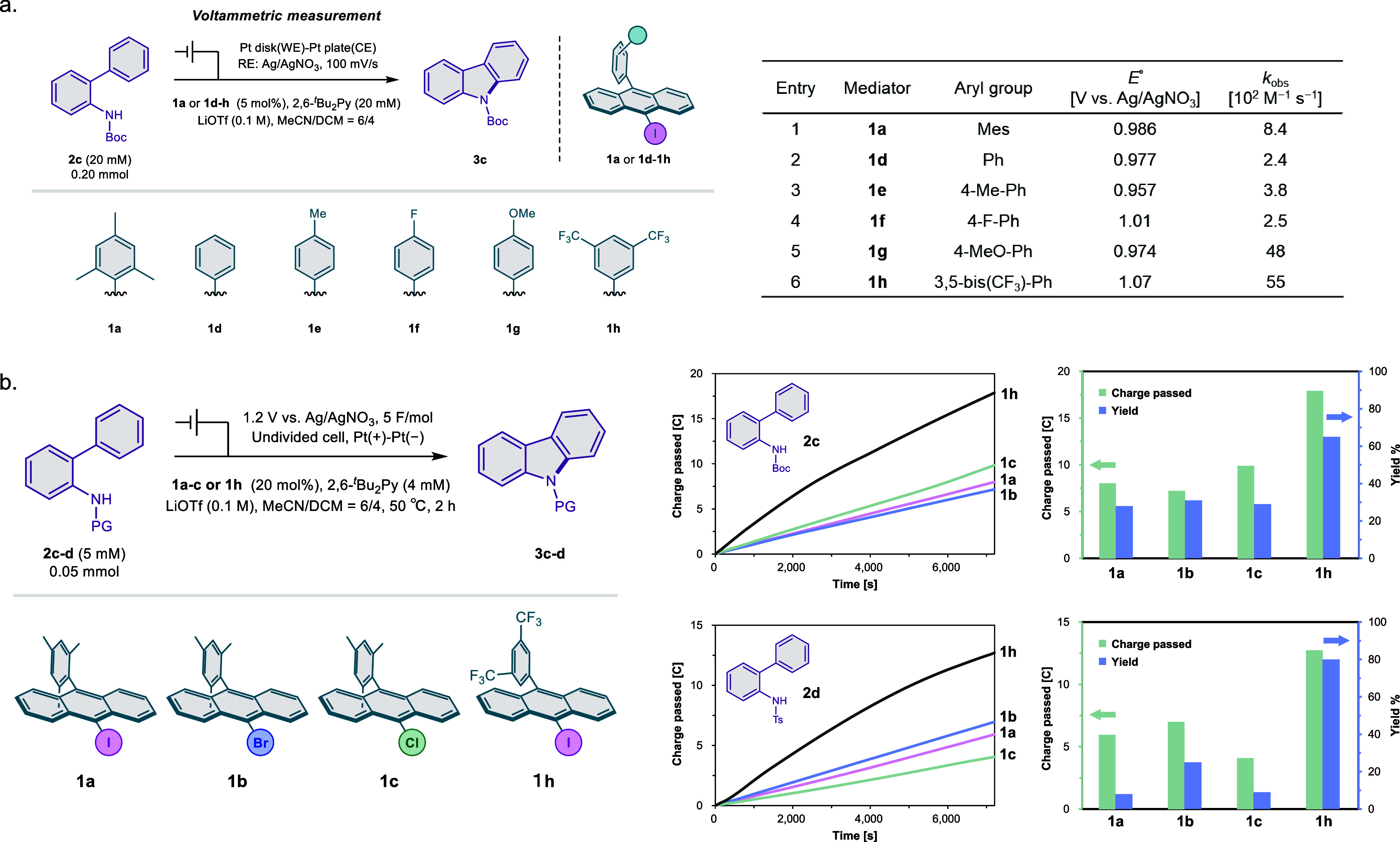
(a) Voltammetric kinetic analysis of 9-Iodo-10-arylanthracene (**1a** and **1d**–**1h**) (top left). *E*° of **1a** and **1d–1h** and yield and *k*_obs_ of the reaction are shown in the top-right table. (b) *Q*–*t* plots in the C–N bond formation of **2c**–**2d** using **1a**–**1c** and **1h** and charge passed and yield in the C–N bond formation (bottom right).

Using **2c** as a model substrate, we performed FOWA to determine the apparent rate constants (*k*_obs_) for each new mediator (Figure 6a). The results revealed large variations in catalytic activity depending on the aryl substituent. These results indicate that **1h** exhibits exceptional catalytic activity, surpassing that of the original **1a** by nearly an order of magnitude. This enhancement is likely due to the highly electron-withdrawing nature of the bis­(trifluoromethyl) groups, which increase the halogen-bond-donor ability.

To confirm this kinetic advantage under preparative conditions, we conducted bulk electrolysis using **1a–1c** and **1h** with substrates **2c** (Boc) and **2d** (Ts) (Figure 6b). Under constant potential electrolysis (1.2 V vs Ag/AgNO_3_), the total charge passed was monitored over time. The **1h**-mediated reactions exhibited the fastest current response, indicating significantly accelerated catalytic turnover. Upon completion of electrolysis, the target carbazole products were obtained in high yields 65% **3c** (from **2c**, 3.7 F mol^–1^) and 80% **3d** (from **2d**, 2.6 F mol^–1^). These results confirm that aryl substitution at the 10-position of the iodoanthracene core can dramatically influence mediator activity.

### Computational Analysis of Halogen Bonding and PCET Pathway

To elucidate the origin of the high reactivity exhibited by the iodoanthracene-based mediators, we conducted detailed computational studies focusing on halogen-bonding interactions and the proton-coupled electron transfer (PCET) mechanism. These studies employed density functional theory geometry optimizations at the (U)­CAM-B3LYP/def2-SVP level, followed by high-level single-point energy calculations using DLPNO-CCSD­(T)/ma-def2-SVP (Figure 7a,b,d), allowing us to construct a reliable energy profile for the full two-electron oxidation process. To evaluate the solvation effect, the RISM-SCF-cSED approach, which is one of the hybrid methods between quantum mechanics and statistical mechanics, was employed. In the energy diagram, the thermal correction was included by employing vibrational frequency analysis. The computational details are summarized in Supporting Information.

**Figure 7:**
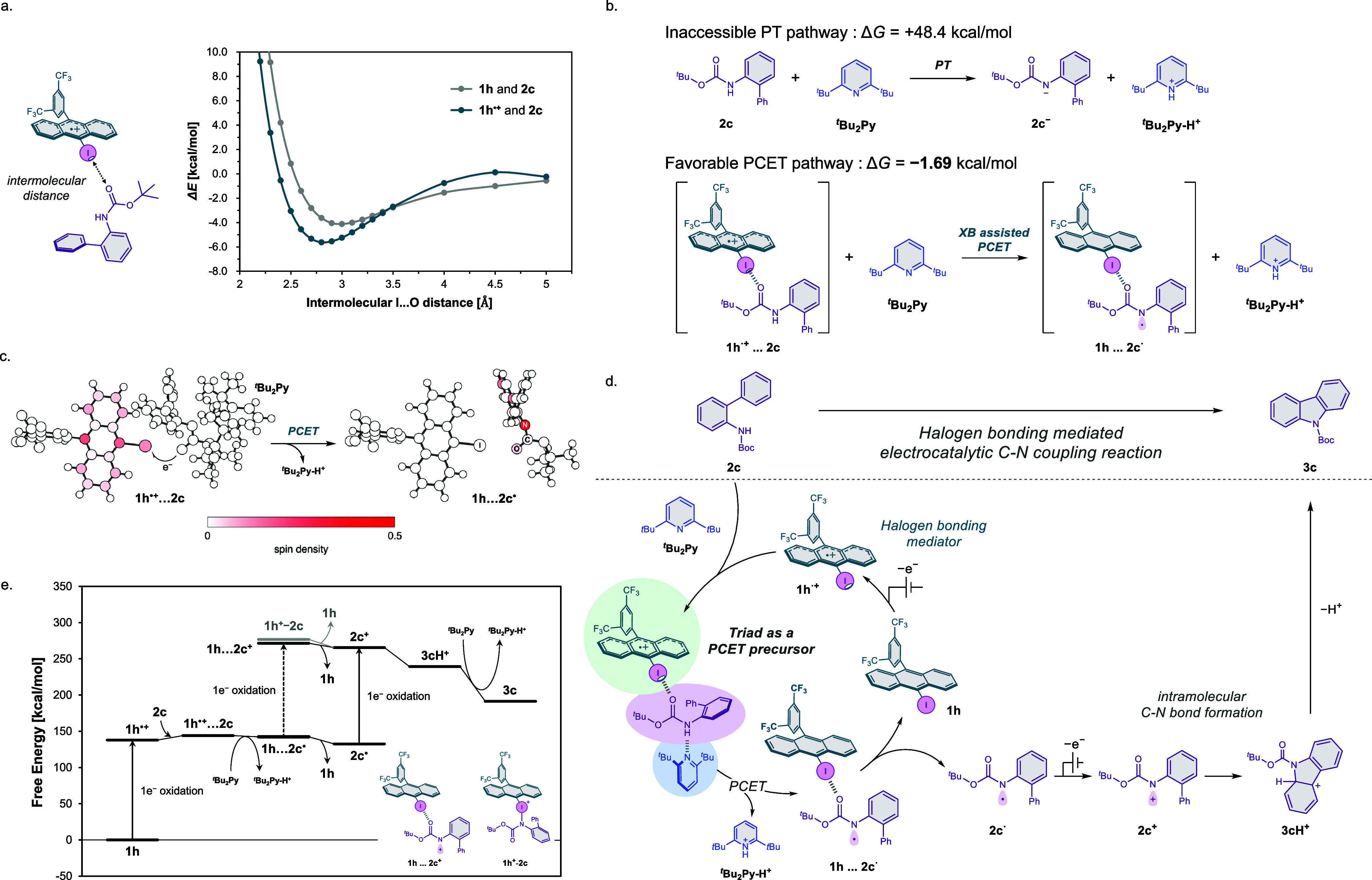
(a) Potential energy curves for **1h** and **1h** ^**•+**^ complexed with **2c**. (b) Δ*G* comparison between the PT process and halogen bonding (XB)-assisted PCET process. (c) Spin densities of **1h···2c** ^**•**^ interacted with ^*t*^Bu_2_Py-H^+^ and (top) and **1h···2c** ^**•**^ (bottom). (d) Plausible reaction mechanism. (e) Energy diagram of the electrocatalytic C–N coupling reaction.

Our first objective was to assess the presence of halogen-bonding interactions between the mediators and the substrate. Using **2c** as a model substrate, we systematically varied the distance between the halogen atom of the mediator **1h** and the carbonyl oxygen of the substrate and evaluated the interaction energies in both neutral and oxidized states (Figure 7a). In their neutral forms, the mediators exhibited only weak interactions with the substrate. However, upon one-electron oxidation, the interaction was strengthened, as evidenced by the appearance of a relatively lower local minimum in the energy curves at X···O distances shorter than the sum of the van der Waals radii (3.20–3.55 Å).^31^ These results indicate that halogen bonding becomes significantly more favorable in the radical cation state. These interactions were observed consistently across all four mediators studied (Figure S32).

Next, we examined the thermodynamics of the abstraction of proton from the substrate by ^*t*^Bu_2_Py. Given the approximate p*K*_a_ of more than 12 for the N–H moiety of *N*-acyl aniline, which possesses electronic properties comparable to those of the substrate, and the p*K*_aH_ of around 1 for the base, such deprotonation is thermodynamically uphill in the absence of mediation.^32,33^ This expectation was confirmed by calculations of the deprotonation free energy of **2c** with and without the radical cation of **1h**. The deprotonation free energies are +48.4 kcal/mol without **1h** ^**•+**^ and −1.7 kcal/mol with **1h** ^**•+**^. By taking the complex form between **2c** and **1h** ^**•+**^, deprotonation by the weak base from the N–H moiety of *N*-acyl aniline becomes possible.

The large stabilization in the deprotonation process with **1h** ^**•+**^ is caused by the PCET. Performing the spin density analyses further clarified the PCET nature of the process (Figure 7c). When the resulting pyridinium ion remained in the vicinity of the mediator-substrate complex, the unpaired electron was localized on the anthracene moiety of the mediator. In contrast, when the pyridinium ion was computationally removed, the spin density shifted onto the substrate. This redistribution implies that the hydrogen bond plays a critical role in stabilizing the charge and spin on the mediator during the PCET event and that the departure of the base triggers electron transfer to the substrate. These findings are fully consistent with a PCET mechanism.^34–36^

To contextualize these mechanistic insights, we proposed a plausible mechanism (Figure 7d) and constructed an energy diagram for the two-electron oxidation process (Figure 7e). The initial one-electron oxidation leads to the generation of a mediator radical cation that forms a halogen-bonded complex with the substrate in its oxidized form. The base also interacts with this complex to yield the key intermediate in the PCET step. After the PCET, the resulting halogen-bonded complex **1h···2c** ^**•**^ can either dissociate to give the free radical **2c** ^**•**^ or undergo direct oxidation while remaining associated. The oxidation energies of both **1h···2c** ^**•**^ and free **2c** ^**•**^ are both smaller than that of the first oxidation step (**1h** → **1h** ^**•+**^), indicating that the second oxidation is energetically feasible through either pathway. On the other hand, the dissociation of **1h···2c** ^**•**^ is exothermic, making it more likely that the second oxidation occurs after the dissociation event. Cationic species **2c** ^**+**^ generated by the second oxidation can interact with the mediator **1h** to form a halogen-bonding complex **1h···2c** ^**+**^ and a hypervalent state **1h** ^**+**^ **–2c**. Such hypervalent iodine species are well-documented to engage in C–N or C–O coupling reactions, suggesting that analogous reactivity could be operative in this system.^20,21,37–39^ However, because both complexes are not stable compared to the free form, the complex form is not important after the **2c** ^**+**^ is produced. Intramolecular ring formation of **2c** ^**+**^ takes place to afford **3cH** ^**+**^ and **3c** is obtained after deprotonation by ^*t*^Bu_2_Py.

Taken together, these computational findings provide strong support for the proposed mechanism involving redox-switchable halogen bonding and PCET. This mechanistic framework underpins the observed high reactivity and selectivity, particularly for optimized mediator **1h**, and provides a clear design rationale for future development of advanced redox mediators.

## Conclusions

In summary, we have developed a new class of redox mediators based on haloanthracene derivatives that exhibit redox-switchable halogen bonding in their radical cation states. These mediators enable efficient electrocatalytic intramolecular C–N bond formation through enhanced substrate preorganization and activation via noncovalent interactions. Among them, iodoanthracene mediator **1a** demonstrated broad substrate compatibility and favorable kinetics, which were further improved through structural tuning at the 10-position.

Systematic modification of the aryl substituent at the 10-position of the iodoanthracene scaffold revealed that the 3,5-bis­(trifluoromethyl)­phenyl-substituted derivative **1h** significantly outperformed its analogs, as evidenced by kinetic analysis and bulk electrolysis experiments. The pronounced enhancement in reactivity was traced to its ability to form stronger halogen-bonding interactions in the oxidized state.

Computational studies provided detailed mechanistic insights, confirming the emergence of halogen bonding only upon one-electron oxidation and demonstrating that this interaction promotes a PCET pathway by increasing the acidity of the substrate’s N–H bond.

This work highlights a new design principle for redox mediators that leverages redox-responsive noncovalent interactions to control both molecular recognition and electron–proton dynamics. The concept of halogen-bonding-assisted PCET opens new directions in electrocatalyst design, offering a broadly applicable strategy for enhancing selectivity and efficiency in synthetic electrochemistry.

## Supplementary Material



## Notes

Additional experimental details, materials, and methods, details of computation, crystallographic data, and ^1^H, ^13^C NMR spectra for all compounds (PDF)

## Abbreviations

Et_2_O: diethyl ether
NMR: nuclear magnetic resonance
HRMS: high-resolution mass spectrometry
EPR: electron paramagnetic resonance
