# Supplementary material for: Redox-Switchable Halogen Bonding in Haloanthracene Mediators Enables Efficient Electrocatalytic C–N Coupling
Source: J Am Chem Soc. 2026 Jan 8;148(6):6249–57. doi: 10.1021/jacs.5c18175 (PMC12921847; doi:10.1021/jacs.5c18175)
Supplement: Supplementary file 1 [file ja5c18175_si_001.pdf]

Supporting Information

Redox-Switchable Halogen Bonding in Haloanthracene Mediators Enables Efficient Electrocatalytic C-N Coupling

Atsuki Hirama,[1] Kayo Suda,[2] Shohei Yoshinaga,[1] Moto Kikuchi,[3] Su-Gi Chong,[4] Azusa Kikuchi,[1] Yusuke Ishigaki,[3] Daisuke Yokogawa,*[2] Mahito Atobe,*[1,4] Naoki Shida*[1,4,5]*

[1]Department of Chemistry and Life Science, Yokohama National University, 79-5 Tokiwadai, Hodogaya-ku, Yokohama 240-8501, Japan
[2]Department of Multidisciplinary Science, Graduate School of Arts and Sciences, The University of Tokyo, Komaba, Meguro-ku, Tokyo 153-8902, Japan
[3]Department of Chemistry, Faculty of Science, Hokkaido University, Sapporo 060-0810, Japan
[4]Institute of Advanced Sciences, Yokohama National University, 79-5 Tokiwadai, Hodogaya-ku, Yokohama 240-8501, Japan
[5]PRESTO, Japan Science and Technology Agency (JST), 4-1-8 Honcho, Kawaguchi, Saitama 332-0012, Japan

Tel: +81-45-339-4214
E-mail: suda.kayo@mail.u-tokyo.ac.jp, c-d.yokogawa@g.ecc.u-tokyo.ac.jp, atobe@ynu.ac.jp, shida-naoki-gz@ynu.ac.jp

Table of Contents

1. *General considerations* S4
2. *Synthesis* S5
   - *2-1. Synthesis of 9-iodo-10-mesitylanthracene **(1a)*** S5
   - *2-2. Synthesis of 9-bromo-10-mesitylanthracene **(1b)*** S6
   - *2-3. Synthesis of 9-chloro-10-mesitylanthracene **(1c)*** S6
   - *2-4. Synthesis of 9-iodo-10-phenylanthracene **(1d)*** S7
   - *2-5. Synthesis of 9-iodo-10-arylanthracene **(1e-1h)*** S8
   - *2-6. Synthesis of N-([1,1'-biphenyl]-2-yl)acetamide **(2a)*** S10
   - *2-7. Synthesis of methyl [1,1'-biphenyl]-2-ylcarbamate **(2b)*** S11
   - *2-8. Synthesis of tert-butyl[1,1'-biphenyl]-2-ylcarbamate **(2c)*** S11
   - *2-9. Synthesis of N-([1,1'-biphenyl]-2-yl)-4-methylbenzenesulfonamide **(2d)*** S12
   - *2-10 Synthesis of [1,1'-biphenyl]-2-amine derivatives* S13
   - *2-11 Synthesis of tert-butyl [1,1'-biphenyl]-2-ylcarbamate derivatives **(2e-i, 2l-o)*** S16
   - *2-12. Synthesis of 9-iodoanthracene **(Anth-I)*** S22
   - *2-13. Synthesis of tert-butyl ([1,1'-biphenyl]-2-yl-2',3',4',5',6'-d_5)carbamate **(2c-d_5)*** S22
   - *2-14. Synthesis of tert-butyl deuterium[1,1'-biphenyl]-2-ylcarbamate **(2c-d_1)*** S23
   - *2-15. Synthesis of 9-mesityl-10-methylanthracene **(1i)*** S24
   - *2-16. Synthesis of 9-(3,5-bis(trifluoromethyl)phenyl)-10-methylanthracene **(1j)*** S25
   - *2-17. General procedure for electrocatalytic intramolecular amination* S26
   - *2-18. General procedure for mediator applicability of N-Boc-aminobiphenyl* S26
   - *2-19 General procedure for substrate applicability of N-Boc- aminobiphenyls* S27
   - *2-20. Refinement details* S30
3. *Supporting data* S35
   - *3-1. Cyclic voltammetry analysis* S35
     - *3-1-1. Cyclic voltammetry of 9-iodoanthracene **(Anth-I)** and 9-iodophenanthrene **(Phen-I)** and 1-iodopyrene **(Pyr-I)*** S35
     - *3-1-2. Cyclic voltammetry of 9-iodoanthracene **(Anth-I)*** S35
     - *3-1-3. Cyclic voltammetry of 9-iodo10-mesitylanthracene **(1a)** and iodobenzene and 4-iodoanisole* S36
     - *3-1-4. Cyclic voltammetry of **1a** with base* S37
     - *3-1-5. Cyclic voltammetry of N-protected aminobiphenyl derivatives **(2a-2d)*** S38
     - *3-1-6. Cyclic voltammetry of **1a** with **2a*** S39
     - *3-1-7. Cyclic voltammetry of **1a** and **2c** or deuterated **2c** derivatives **(2c-d_1** and **2c-d_5)*** S39
     - *3-1-8. Cyclic voltammetry of 9-halo-10-arylanthrancene **(1a-1h)** and N-protected aminobiphenyl derivatives for FOWA* S40
   - *3-2. Electron paramagnetic spectroscopy (EPR) measurement* S46
   - *3-3. Computational analysis* S49
     - *3-3-1. Computational details* S49
     - *3-3-2. Spin density mapping* S50
     - *3-3-3. Electrostatic potential mapping* S50
     - *3-3-4. Comparison of halogen bonding interaction and π-π stacking* S51

3-3-5. Cartesian coordinates of the optimized structures S52
*3-3-6. Potential energy curves for **1a**-**1c** and **1a**$^{•+}$-**1c**$^{•+}$ complexed with **2c*** S61
3-4. Quantitative analysis of halogen-bonding interaction S61
3-5. Screening of electrolysis condition S63
3-6. Control experiments S65
3-7. Kinetic analysis S65
3-7-1. Introduction S65
3-7-2. The procedure of Foot-of the-wave analysis (FOWA) S66
3-7-3. Kinetic analysis of the catalytic reaction using FOWA S67
4. NMR charts S69
5. Supporting references S159

1. General considerations

Reagents and dry solvents were obtained from commercial sources and used without further purification. Reactions were performed under an inert atmosphere with the Schlenk technique unless otherwise noted. ^{1}H, ^{13}C NMR spectra were recorded on JEOL ECA500 (^{1}H: 500 MHz, ^{13}C: 126 MHz), Bruker DRX 500 (^{1}H: 500 MHz), Bruker AVANCE NEO 500 (^{13}C: 126 MHz), and Bruker AVANCE NEO 600 (^{1}H: 600 MHz) spectrometers using $CDCl_3$ as a solvent. The chemical shifts for ^{1}H, ^{13}C, NMR spectra are given in δ (ppm) relative to internal tetramethylsilane, deuterated solvent, respectively. Multiplicities are abbreviated as singlet (s), doublet (d), triplet, (t), doublet of doublets (dd), doublet of triplets (dt), triplet of doublets (td), doublet of doublet of doublets (ddd), multiplet (m). Electrospray-ionization time-of-flight mass spectra (ESI-TOF MS) were obtained on a Bruker Daltonics microTOF focus II. Single-crystal X-ray structure analyses were performed by a Rigaku XtaLAB Synergy (Cu−Kα radiation, λ=1.54184 Å) with HyPix diffractometer. Using Olex2,[1] the structure was solved with the SHELXT[2] structure solution program using Intrinsic Phasing and refined with the SHELXL[3] refinement package using Least Squares minimization. All the hydrogen atoms were located at the calculated positions and refined with riding. Electron paramagnetic resonance (EPR) spectroscopy measurement was performed using Electron Spin Resonance Spectrometer JES-FA200 X-band (JEOL). The experimental magnetic field was calibrated with an Echo Electronics EFM-2000AX proton NMR gauss meter. Cyclic voltammetry (CV) measurements were performed using ALS Instruments model 630E and 660E, and BAS model2323 bipotentiostat (BAS). Square wave voltammetry (SWV) measurements were performed at a step potential of 2 mV, an amplitude of 20 mV, and at a frequency of 25 Hz using potentiostat VSP-3A (Biologic). All CV and SWV measurements were carried out in the three-electrode system equipped with a platinum (Pt) disk working electrode (φ = 1.6 mm and 3.0 mm), a Pt plate counter electrode (20 mm × 20 mm), and Ag/$AgNO_3$ reference electrode (containing 10 mM $AgNO_3$ and 0.1 M Bu_4NClO_4 in acetonitrile). Constant Potential Electrolysis and Constant Current Electrolysis were performed using a potentiostat HABF-501A (Hokuto Denko) and VSP-3A (BioLogic).

2. Synthesis

2-1. Synthesis of 9-iodo-10-mesitylanthracene ***(1a)***

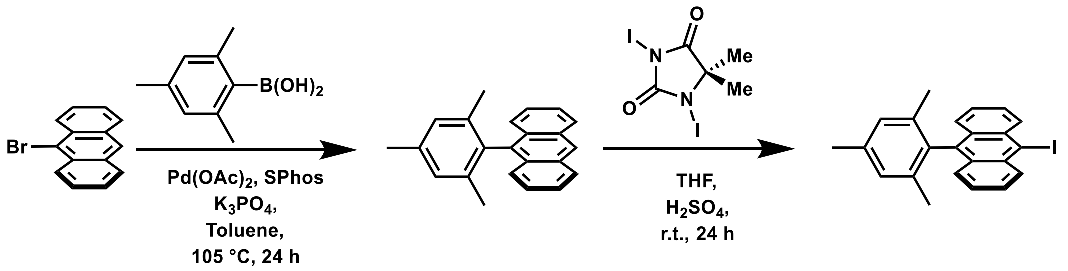

9-Iodo-10-mesitylanthracene was obtained from 9-bromoanthracene in two steps. 9-Bromoanthracene (3.82 g, 15 mmol), 2,4,6-trimethylphenylboronic acid (3.69 g, 22.5 mmol), $Pd(OAc)_2$ (0.101 g, 0.45 mmol), SPhos (0.3695 g, 0.90 mmol), K_3PO_4 (7.97 g, 37.5 mmol) were dissolved in anhydrous toluene (120 mL) under a nitrogen atmosphere and the mixture was reacted at 105 °C for 24 h under vigorous stirring. After cooling to room temperature, the reaction was quenched by adding water, and the reaction solution was filtered. The organic layer was separated, and the aqueous layer was extracted with CH_2Cl_2 twice. The organic extracts were washed with brine and dried over $MgSO_4$. After filtration and removal of the solvent, the obtained residue was purified by silica gel column chromatography using hexane as an eluent. The yellow solid was washed with ice-cold MeOH. The desired product, 9-mesitylanthracene was obtained as a light-yellow solid. (1.66 g, 5.6 mmol, 38%).

9-Mesitylanthracene: ^{1}H NMR (500MHz, $CDCl_3$, ppm): δ = 8.48 (s, 1H), 8.06 (d, J = 8.2 Hz, 2H), 7.51-7.41 (m, 4H), 7.32 (ddd, J = 8.8, 6,6 1,3 Hz, 2H), 7.09 (s, 2H), 2.45 (s, 3H), 1.70 (s, 6H); The obtained ^{1}H NMR spectrum corresponded to the reported data.[4]

9-Mesitylanthracene (1.66 g, 5.6 mmol) was dissolved in THF (40 mL). 1,3-Diiodo-5,5-dimethylhydantoin (2.75 g, 7.24 mmol) and concentrated H_2SO_4 (0.18 mL) were added to the solution at 0 °C. The mixture was reacted at room temperature for 24 h under vigorous stirring. The reaction was quenched by adding water and diluted with CH_2Cl_2. The organic layer was separated, and the aqueous layer was extracted with CH_2Cl_2 twice. The organic extracts were washed with saturated $NaHCO_3$ aq. and brine. When organic layer showed purple color derived from I_2, the organic layer was washed with mixed solution of L-ascorbic acid and KI in water. Then the organic layer was dried over $MgSO_4$. After filtration and removal of the solvent, the obtained residue was purified by silica gel column chromatography using hexane. The desired product, 9-iodo-10-mesitylanthracene was obtained as a light-yellow solid. (1.26 g, 2.98 mmol, 53%).[5]

9-Iodo-10-mesitylanthracene: ^{1}H NMR (500MHz, $CDCl_3$, ppm): δ = 8.5 (d, *J* = 8.9 Hz, 2H), 7.59-7.53 (m, 2H), 7.45 (dd, *J* = 8.7, 0.8 Hz, 2H), 7.37-7.32 (m, 2H), 7.09 (s, 2H), 2.46 (s, 3H), 1.69 (s, 6H); ^{13}C NMR (126 MHz, $CDCl_3$, ppm): δ = 138.08, 137.63, 137.50, 134.29, 134.01, 133.92, 130.72, 105.22, 21.4, 20.15; HRMS (ESI-TOF): calcd for $C_{23}H_{19}INa^{+}$ ($[M+Na]^{+}$) m/z 445.0424, found 445.0424.

2-2. Synthesis of 9-bromo-10-mesitylanthracene ***(1b)***

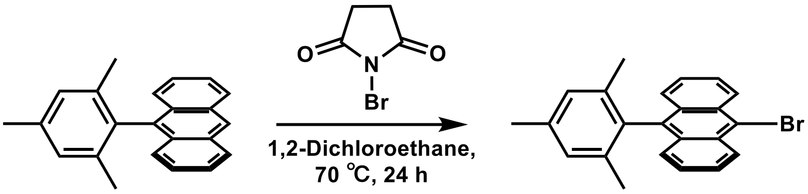

9-Mesitylanthracene (303.0 mg, 1.023 mmol) and was dissolved in 1,2-dichloroethane (10 mL). *N*-Bromosuccinimide (218.0 mg, 1.225 mmol) was added to the solution at ambient temperature, and the mixture was reacted at 70 °C for 24 h under vigorous stirring. The reaction was quenched by adding water and diluted with $CHCl_3$. The organic layer was separated and washed with water for three times. Then the organic layer was dried over $MgSO_4$. After filtration and removal of the solvent, the obtained residue was purified by silica gel column chromatography using hexane and ethyl acetate. The desired product, 9-bromo-10-mesitylanthracene was obtained as a light-yellow solid. (336.2 mg, 0.896 mmol, 88%).

9-Bromo-10-mesitylanthracene: ^{1}H NMR (500 MHz, $CDCl_3$, ppm) δ = 8.61 (d, *J* = 8.8 Hz, 2H), 7.59 (ddd, *J* = 8.9, 6.5, 1.2 Hz, 2H), 7.50 – 7.47 (dt, *J* = 9.0, 0.9, Hz, 2H), 7.36 (ddd, *J* = 8.7, 6.5, 1.2 Hz, 2H), 7.09 (s, 2H), 2.45 (s, 3H), 1.69 (s, 6H); ^{13}C NMR (126 MHz, $CDCl_3$, ppm): δ = 137.62, 137.57, 136.73, 134.23, 130.72, 130.67, 128.50, 128.24, 127.20, 126.53, 126.02, 122.38, 77.41, 77.16, 76.91, 21.38, 20.13; HRMS (ESI-TOF): calcd for $C_{23}H_{19}Br^{+}$ ($[M]^{+}$) m/z 374.0670, found 374.0672.

2-3. Synthesis of 9-chloro-10-mesitylanthracene ***(1c)***

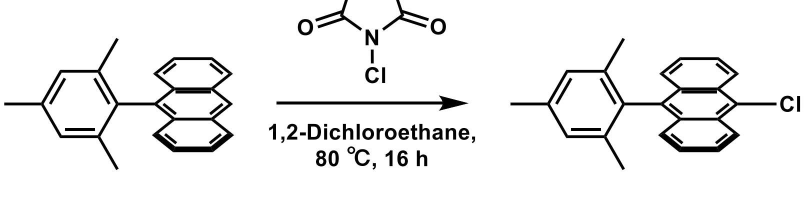

9-Mesitylanthracene (230 mg, 0.776 mmol) was dissolved in 1,2-dichloroethane (10 mL). *N*-Chrolosuccinimide (154 mg, 1.16 mmol) was added to the solution at ambient temperature, and the mixture was reacted at 80 °C for 16 h under vigorous stirring. The reaction was quenched by adding water and diluted with $CHCl_3$. The organic layer was separated and washed with water for three times. Then the organic layer was dried over $MgSO_4$. After filtration and removal of the solvent, the obtained residue was purified by silica gel column chromatography using hexane. The desired product, 9-chloro-10-mesitylanthracene was obtained as a light-yellow solid. (213 mg, 0.643 mmol, 83%).

9-Chloro-10-mesitylanthracene: ^1H NMR (500 MHz, $CDCl_3$, ppm) δ = 8.59 (d, *J* = 8.9 Hz, 2H), 7.59 (ddd, *J* = 8.9, 6.4, 1.2 Hz, 2H), 7.49 (d, *J* = 8.7 Hz, 2H), 7.37 (ddd, *J* = 8.8, 6.4, 1.2 Hz, 2H), 7.09 (s, 2H), 2.45 (s, 3H), 1.69 (s, 6H); ^{13}C NMR (126 MHz, $CDCl_3$, ppm): δ = 137.68, 137.59, 135.72, 134.16, 130.41, 128.95, 128.50, 128.28, 126.81, 126.49, 126.01, 125.26, 77.41, 77.16, 76.91, 21.38, 20.12; HRMS (ESI-TOF): calcd for $C_{23}H_{19}Cl^+$ ($[M]^+$) m/z 330.1175, found 330.1170.

2-4. Synthesis of 9-iodo-10-phenylanthracene ***(1d)***

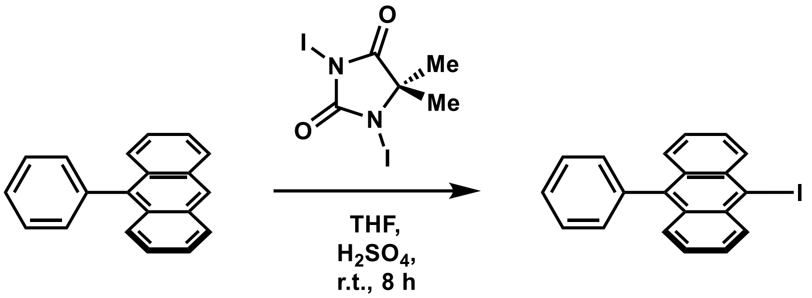

9-Phenylanthracene (1.00 g, 3.93 mmol) was dissolved in THF (30 mL). 1,3-Diiodo-5,5-dimethylhydantoin (1.57 g, 4.13 mmol) and concentrated H_2SO_4 (0.12 mL) were added to the solution at 0 °C. The mixture was reacted at room temperature for 8 h under vigorous stirring. The reaction was quenched by adding water and diluted with CH_2Cl_2. The organic layer was separated, and the aqueous layer was extracted with CH_2Cl_2 twice. When organic layer showed purple color derived from I_2, the organic layer was washed with mixed solution of L-ascorbic acid and KI in water. Then the organic layer was dried over $MgSO_4$. After filtration and removal of the solvent, the obtained residue was purified by silica gel column chromatography using hexane and ethyl acetate (9/1). The desired product, 9-iodo-10-phenylanthracene was obtained as an orange solid. (580 mg, 1.53 mmol, 38%).

9-Iodo-10-phenylanthracene: ^1H NMR (500MHz, $CDCl_3$, ppm): δ = 8.57 (dd, *J* = 8.9, 1.0 Hz, 2H), 7.62 – 7.54 (m, 7H), 7.42 – 7.39 (m, 2H), 7.38 – 7.34 (m, 2H).

2-5. Synthesis of 9-iodo-10-arylanthracene ***(1e-1h)***

General Procedure for the synthesis of biphenyl amine-derivatives

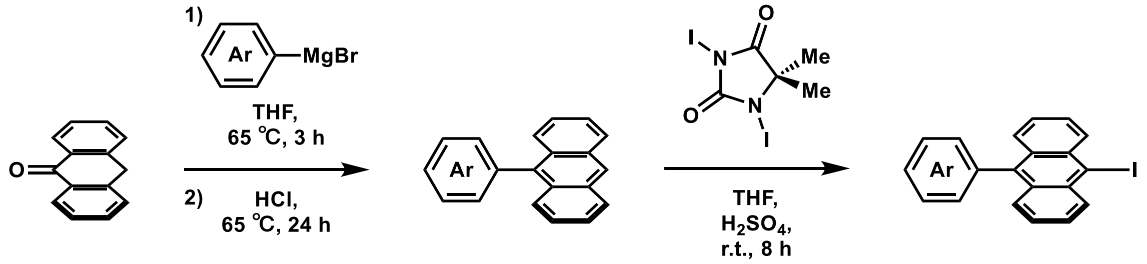

9-Iodo-10-arylanthracene was obtained from anthrone in two steps.

Anthrone (1.0 g, 5.1 mmol) was dissolved in anhydrous THF (40 mL) under a nitrogen atmosphere. Arylmagnessium bromide (8.22 mmol, 1.6 equiv.) was added dropwise, and the mixture was reacted at 65 ºC for 3 h under vigorous stirring. Aqueous hydrochloric acid (1 M, 8.6 mL) was added to the solution, and the mixture was reacted at 65 ºC for 24 h under vigorous stirring. After cooling to room temperature, the reaction was quenched by adding water and diluted with CH_2Cl_2. The organic layer was separated, and the aqueous layer was extracted with CH_2Cl_2 for three times. Then the organic layer was washed with brine and dried over $MgSO_4$. After filtration and removal of the solvent, the obtained residue was purified by silica gel column chromatography using cyclohexane/CH_2Cl_2 (50/1) to afford desired products.

9-Arylanthracene was dissolved in THF (15 mL). 1,3-Diiodo-5,5-dimethylhydantoin (1.29 equiv.) and concentrated H_2SO_4 (1.68 equiv.) were added to the solution at 0 ºC. The mixture was reacted at room temperature for 8 h under vigorous stirring. The reaction was quenched by adding water and diluted with CH_2Cl_2. The organic layer was separated, and the aqueous layer was extracted with CH_2Cl_2 twice. Then the organic layer was dried over $MgSO_4$. After filtration and removal of the solvent, the obtained residue was purified by silica gel column chromatography using hexane/ethyl acetate (9/1) to afford desired products.

9-(*p*-tolyl)anthracene: Prepared from *p*-tolylmagnessium bromide (1 M in THF), 9-(*p*-tolyl)anthracene was obtained as a white solid (219.6 mg, 0.818 mmol, 16%). ^{1}H NMR (500MHz, $CDCl_3$, ppm): δ = 8.48 (s, 1H), 8.04 (d, *J* = 8.5 Hz, 2H), 7.69 (dd, *J* = 8.9, 1.0 Hz, 2H), 7.45 (ddd, *J* = 8.5, 6.5 1.2 Hz, 2H), 7.39 (d, *J* = 7.5 Hz, 2H), 7.36 – 7.31 (m, 4H), 2.53 (s, 3H); The obtained ^{1}H NMR spectrum corresponded to the reported data.[6]

9-Iodo-10-(*p*-tolyl)anthracene (1e): Prepared from 9-(*p*-tolyl)anthracene (219.6 mg, 0.818 mmol) and 1,3-diiodo-5,5-dimethylhydantoin (400 mg, 1.06 mmol) and concentrated H_2SO_4 (26 µL), the product was washed with ice cold methanol. **1e** was obtained as an ocher solid (146.9 mg, 0.373 mmol, 46%). ^{1}H NMR (500MHz, $CDCl_3$, ppm): δ = 8.55 (d, *J* = 8.8 Hz, 2H), 7.63 (d, *J* = 8.7 Hz, 2H), 7.55 (ddd, *J* = 8.9, 6.5, 1.2 Hz, 2H), 7.39 (d, *J* = 7.5 Hz, 2H), 7.35 (ddd, *J* = 8.8, 6.5, 1.1 Hz, 2H), 7.28 (d, *J* = 8.0 Hz, 2H), 2.53 (s, 3H); ^{13}C NMR (126 MHz, $CDCl_3$, ppm): δ = 139.44, 137.57, 135.47, 133.78, 133.67, 131.31, 131.10, 129.29, 127.89, 127.58, 125.60, 105.53, 77.41, 77.16, 76.91, 21.54; HRMS (ESI-TOF): calcd for $C_{21}H_{15}I^+$ ($[M]^+$) m/z 394.0219, found 394.0208.

9-(4-fluorophenyl)anthracene: Prepared from 4-fluorophenylmagnessium bromide (2.0 M in Et_2O), 9-(4-fluorophenyl)anthracene was obtained as a white solid (239.4 mg, 0.879 mmol, 17%). ^{1}H NMR (600 MHz, $CDCl_3$, ppm) δ = 8.51 (s, 1H), 8.05 (d, *J* = 8.5 Hz, 2H), 7.63 (dd, *J* = 8.8, 1.0 Hz, 2H), 7.48 – 7.45 (m, 2H), 7.40 (td, *J* = 8.5, 5.5 Hz, 2H), 7.36 (ddd, *J* = 8.9, 6.5, 1.3 Hz, 2H), 7.28 (t, *J* = 8.7 Hz, 2H); The obtained ^{1}H NMR spectrum corresponded to the reported data.[7]

9-(4-fluorophenyl)-10-iodoanthracene (1f): Prepared from 9-(4-fluorophenyl)anthracene (239.4 mg, 0.879 mmol) and 1,3-diiodo-5,5-dimethylhydantoin (429.9 mg, 1.13 mmol) and concentrated H_2SO_4 (28 µL), **1f** was obtained as a light-yellow solid (87.0 mg, 0.218 mmol, 25%). ^{1}H NMR (500MHz, $CDCl_3$, ppm): δ = 8.55 (dd, *J* = 9.4, 1.1 Hz, 2H), 7.57 – 7.52 (m, 4H), 7.38 – 7.32 (m, 4H), 7.27 (t, *J* = 8.7 Hz, 2H); ^{13}C NMR (126 MHz, $CDCl_3$, ppm): δ = 163.63, 161.66, 137.98, 134.35 (d, *J* = 3.6 Hz), 133.90, 133.64, 132.82 (d, *J* = 8.2 Hz), 131.26, 127.55 (d, *J* = 22.2 Hz), 125.91, 115.69 (d, *J* = 21.2 Hz), 106.14; HRMS (ESI-TOF): calcd for $C_{20}H_{12}FI^+$ ($[M]^+$) m/z 397.9968, found 397.9957.

9-(4-methoxyphenyl)anthracene: Prepared from 4-methoxyphenylmagnessium bromide (0.5 M in THF), 9-(4-methoxyphenyl)anthracene was obtained as a white solid (227.0 mg, 0.798 mmol, 16%). ^{1}H NMR 1H NMR (600 MHz, $CDCl_3$, ppm) δ = 8.48 (s, 1H), 8.04 (d, *J* = 8.5 Hz, 2H), 7.71 (dd, *J* = 8.8, 1.0 Hz, 2H), 7.45 (ddd, *J* = 8.5, 6.5, 1.2 Hz, 2H), 7.36 – 7.33 (m, 4H), 7.12 (dt, *J* = 5.2, 0.0 Hz, 2H), 3.95 (s, 3H); The obtained 1H NMR

spectrum corresponded to the reported data.[6]

9-iodo-10-(4-methoxyphenyl)anthracene (1g): Prepared from 9-(4-methoxyphenyl)anthracene (227.0 mg, 0.798 mmol) and 1,3-diiodo-5,5-dimethylhydantoin (390 mg, 1.04 mmol) and concentrated H_2SO_4 (25 μL), **1g** was obtained as a yellow solid (68.9 mg, 0.168 mmol, 21%). ^{1}H NMR (500MHz, $CDCl_3$, ppm): δ = 8.56 (d, *J* = 9.0 Hz, 2H), 7.65 (d, *J* = 8.7 Hz, 2H), 7.56 (ddd, *J* = 8.9, 6.5, 1.2 Hz, 2H), 7.36 (ddd, *J* = 8.8, 6.5, 1.2 Hz, 2H), 7.31 (dt, *J* = 9.8, 2.1 Hz, 2H), 7.12 (dt, *J* = 8.6, 2.1 Hz, 2H), 3.95 (s, 3H); ^{13}C NMR (126 MHz, $CDCl_3$, ppm): δ = 159.38, 139.13, 133.80, 133.69, 132.32, 131.54, 130.58, 127.87, 127.58, 125.62, 114.06, 105.56, 77.41, 77.16, 76.91, 55.55; HRMS (ESI-TOF): calcd for $C_{21}H_{15}OI^+$ ($[M]^+$) m/z 410.0168, found 410.0163.

9-(3,5-bis(trifluoromethyl)phenyl)anthracene: Prepared from 3,5-bis(trifluoromethyl)phenylmagnessium bromide (0.5 M in THF), 9-(3,5-bis(trifluoromethyl)phenyl)anthracene was obtained as a light-yellow solid (862.5 mg, 2.21 mmol, 43%). ^{1}H NMR (500MHz, $CDCl_3$, ppm) δ = 8.58 (s, 1H), 8.09 (s, 1H), 8.08 (s, 2H), 7.92(s, 2H), 7.50 (ddd, *J* = 8.5, 6.1, 1.5 Hz, 2H), 7.47 – 7.39 (m, 4H); ^{13}C NMR (126 MHz, $CDCl_3$, ppm): δ = 141.42, 133.04, 132.17 (d, *J* = 33.2 Hz), 131.66, 131.38, 130.15, 128.87, 128.29, 126.64, 125.60 (d, *J* = 12.4 Hz), 124.64, 122.47, 121.83; HRMS (ESI-TOF): calcd for $C_{22}H_{12}F_6^+$ ($[M]^+$) m/z 390.0843, found 390.0843.

9-(3,5-bis(trifluoromethyl)phenyl)-10-iodoanthracene (1h): Prepared from 9-(3,5-bis(trifluoromethyl)phenyl)anthracene (608.2 mg, 1.54 mmol) and 1,3-diiodo-5,5-dimethylhydantoin (754 mg, 1.99 mmol) and concentrated H_2SO_4 (49 μL), **1h** was obtained as a yellow solid (146.9 mg, 0.373 mmol, 46%). ^{1}H NMR (500MHz, $CDCl_3$, ppm): δ = 8.61 (d, *J* = 8.9 Hz, 2H), 8.10 (s, 1H), 7.90 (s, 2H), 7.61 (ddd, *J* = 8.9, 6.4, 1.3 Hz, 2H), 7.44 (ddd, *J* = 8.8, 6.4, 1.1 Hz, 2H), 7.38 (d, *J* = 8.7 Hz, 2H); ^{13}C NMR (126 MHz, $CDCl_3$, ppm): δ = 141.09, 135.01, 134.33, 133.64, 132.32 (q, *J* = 33.6 Hz), 131.43, 130.75, 127.87, 126.67 (d, *J* = 54.8 Hz), 124.53, 122.36, 122.16, 107.77; HRMS (ESI-TOF): calcd for $C_{22}H_{11}F_6I^+$ ($[M]^+$) m/z 515.9810, found 515.9805.

2-6. Synthesis of N-([1,1'-biphenyl]-2-yl)acetamide ***(2a)***

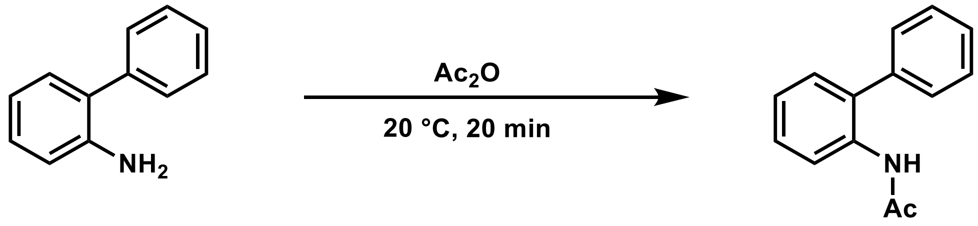

2-Aminobiphenyl (1.701 g, 10 mmol) was dissolved in acetic anhydride (10 mL). The mixture was reacted at room temperature for 20 min. The reaction was quenched by adding saturated K_2CO_3 aq. solution at 0 ºC. The resulting precipitate was collected by vacuum filtration. Then, the residual solid was washed with water and hexane and dried under reduced pressure. The desired product, *N*-([1,1'-biphenyl]-2-yl)acetamide (**2a**) was obtained as a white solid (1.827 g, 8.65 mmol, 86%).

***N*-([1,1'-biphenyl]-2-yl)acetamide**: ^{1}H NMR (500MHz, $CDCl_3$, ppm): δ = 8.27 (d, *J* = 8.26 Hz, 1H), 7.49 (t, *J* = 7.47 Hz, 2H), 7.46-7.34 (m, 4H), 7.25-7.15 (m, 2H), 7.12 (s, 1H), 2.02 (s, 3H); The obtained ^{1}H NMR spectrum corresponded to the reported data.[8]

*2-7. Synthesis of methyl [1,1'-biphenyl]-2-ylcarbamate (**2b**)*

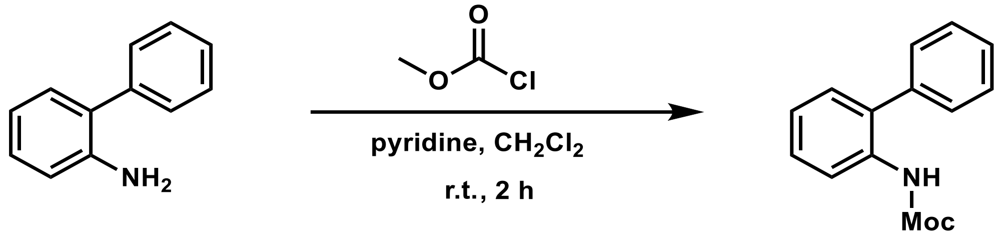

2-Aminobiphenyl (1.00 g, 5.91 mmol) was dissolved in CH_2Cl_2 (15 mL), and methyl chloroformate (0.67 g, 7.09 mmol) and pyridine (619 µL, 7.68 mmol) were added to the solution at 0 ºC. The mixture was reacted at room temperature for 2 h. The reaction was quenched by adding water. The organic layer was washed with diluted hydrochloric acid two times, saturated $NaHCO_3$ aq. solution, water, and brine and then, dried over Na_2SO_4. After filtration and removal of the solvent, the residue was purified by silica gel column chromatography using hexane/ethyl acetate (9/1). The desired product, methyl [1,1'-biphenyl]-2-ylcarbamate (**2b**) was obtained as a white solid. (0.675 g, 2.97 mmol, 50%).

Methyl [1,1'-biphenyl]-2-ylcarbamate: ^{1}H NMR (500MHz, $CDCl_3$, ppm): δ = 8.14 (d, *J* = 7.57 Hz, 1H), 7.53-7.44 (m, 2H), 7.44-7.33 (m, 4H), 7.21 (dd, *J* = 7.60, 1.70 Hz, 1H), 7.13 (td, *J* = 7.47, 7.46, 1.20 Hz, 1H), 6.65 (s, 1H), 3.71 (s, 3H); The obtained ^{1}H NMR spectrum corresponded to the reported data.[9]

*2-8. Synthesis of tert-butyl[1,1'-biphenyl]-2-ylcarbamate (**2c**)*

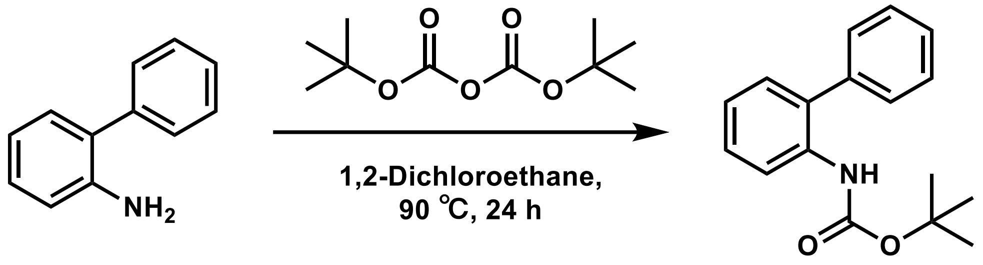

2-Aminobiphenyl (1.00 g, 5.91 mmol), di-*tert*-butyl-dicarbonate (6.45 g, 29.55 mmol) were dissolved in 1,2-dichloroethane (49 mL) under a nitrogen atmosphere and the mixture was heated at 90 ºC for 24 h under vigorous stirring. The reaction was quenched by adding 2,2,2-trifluoroethanol (2.1 mL, 29.55 mmol), DMAP (361.6 mg, 2.96 mmol). The organic layer was washed with water and brine, then dried over Na_2SO_4. The obtained residue was purified by silica gel column chromatography using hexane/ethyl acetate (19/1) to afford **2c**.

***tert*-Butyl [1,1'-biphenyl]-2-ylcarbamate**: ^{1}H NMR (500MHz, $CDCl_3$, ppm): δ = 8.11 (d, *J* = 8.3 Hz, 1H), 7.49 (dd, *J* = 8.1, 6.8 Hz, 2H), 7.43-7.31 (m, 4H), 7.20 (dd, *J* = 7.6, 1.7 Hz, 1H), 7.10 (td, *J* = 7.5, 1.2 Hz, 1H), 6.50 (s, 1H), 1.46 (s, 9H); The obtained ^{1}H NMR spectrum corresponded to the reported data.[10]

*2-9. Synthesis of N-([1,1'-biphenyl]-2-yl)-4-methylbenzenesulfonamide (**2d**)*

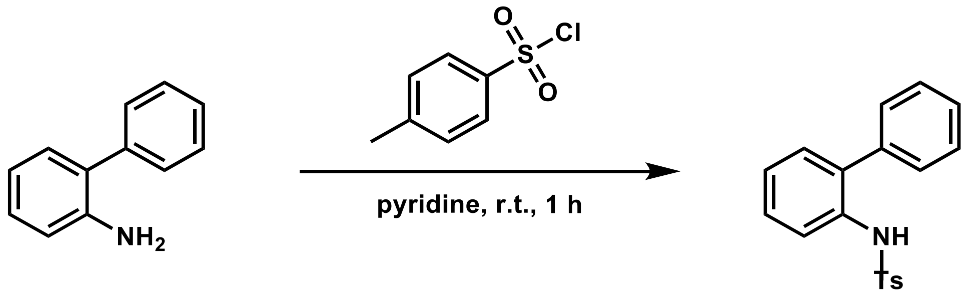

2-Aminobiphenyl (1.00 g, 5.91 mmol) was dissolved in pyridine (20 mL), and *p*-toluenesulfonyl chloride (1.61 g, 8.45 mmol) was added to the solution at 0 ºC. The mixture was reacted at room temperature for 1 h. The reaction was quenched by adding water. The organic layer was washed with diluted hydrochloric acid, saturated $NaHCO_3$ aq. solution, water, and brine and then, dried over Na_2SO_4. After filtration and removal of the solvent, the residue was purified by silica gel column chromatography using hexane/ethyl acetate (8/2). The desired product, *N*-([1,1'-biphenyl]-2-yl)-4-methylbenzenesulfonamide (**2d**) was obtained as a white solid. (1.269 g, 3.90 mmol, 66%).

***N*-([1,1'-Biphenyl]-2-yl)-4-methylbenzenesulfonamide**: ^{1}H NMR (500 MHz, $CDCl_3$, ppm) δ = 7.71 (dd, *J* = 8.2, 1.2 Hz, 1H), 7.47 (d, *J* = 8.3 Hz, 2H), 7.39 – 7.30 (m, 4H), 7.19 (dd, *J* = 8.6, 0.7 Hz, 2H), 7.15 (td, *J* = 7.4, 1.2 Hz, 1H), 7.10 (dd, *J* = 7.6, 1.7 Hz, 1H), 6.85 (dd, *J* = 7.8, 1.7 Hz, 2H), 6.57 (s, 1H), 2.40 (s, 3H); The obtained ^{1}H NMR spectrum corresponded to the reported data.[11]

2-10. Synthesis of [1,1'-biphenyl]-2-amine derivatives

General Procedure for the synthesis of biphenyl amine-derivatives

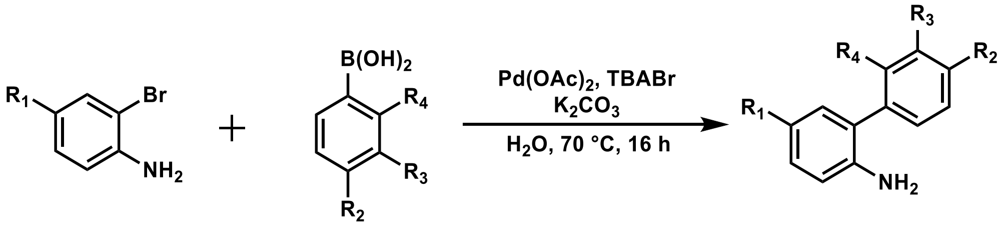

2-Bromoaniline-derivatives (1.00 equiv.), arylboronic acid-derivatives (1.50 equiv.), $Pd(OAc)_2$ (0.20 mol%), tetrabutylammonium bromide (1.00 equiv.), K_2CO_3 (2.50 equiv.) were dissolved in water (0.5 M) under a nitrogen atmosphere and the mixture was heated at 70 °C for 16 h under vigorous stirring. After cooling to room temperature and adding ethyl acetate (50 mL), the reaction solution was filtered over celite. The organic layer was separated, and the aqueous layer was extracted with ethyl acetate two times. The organic extracts were washed with brine and dried over $MgSO_4$. After filtration and removal of the solvent, the obtained residue purified by silica gel column chromatography using hexane/ethyl acetate (19/1) to afford to obtain a spectroscopically pure product.

5-Chloro-[1,1'-biphenyl]-2-amine: Prepared from 2-bromo-4-chloroaniline (1.52 g, 7.26 mmol), and phenylboronic acid (1.33 g, 10.89 mmol), **S2-2** was obtained as a colorless oil (1.25 g, 6.12 mmol, 83%). ^{1}H NMR (500MHz, $CDCl_3$, ppm): δ = 7.49-7.40 (m, 4H), 7.39-7.34 (m,1H), 7.10 (dd, J = 6.61, 2.46 Hz, 2H), 6.69 (d, J = 9.21 Hz, 1H), 3.75 (s, 2H); The obtained ^{1}H NMR spectrum corresponded to the reported data.[8]

5-Methoxy-[1,1'-biphenyl]-2-amine: Prepared from 2-bromo-4-methoxyaniline (0.875 g, 4.95 mmol), and phenylboronic acid (0.905 g, 7.42 mmol), **S2-6** was obtained as a colorless oil (0.43 g, 2.17 mmol, 50%). ^{1}H NMR (500MHz, $CDCl_3$, ppm): δ = 7.49-7.43 (m, 4H), 7.36 (ddt, J = 6.75, 5.78, 2,41, 2,41 Hz, 1H), 6.78 (dd, J = 8.48, 2.92 Hz, 1H), 6.75-6.71 (m, 2H), 3.77 (s, 3H), 3.49 (s, 2H); The obtained ^{1}H NMR spectrum corresponded to the reported data.[8]

4'-Methoxy-[1,1'-biphenyl]-2-amine: Prepared from 2-bromoaniline (1.52 g, 8.72 mmol), and 4-methoxyphenylboronic acid (2.00 g, 13.1 mmol), **S2-9** was obtained as a colorless oil (1.45 g, 7.30 mmol, 83%). ^{1}H NMR (500MHz, $CDCl_3$, ppm): δ = 7.43-7.34 (m, 2H), 7.19-7.09 (m, 2H), 7.03-6.94 (m, 2H), 6.83 (tt, J = 7.42, 7.42, 0.97, 0.97 Hz, 1H), 6.77 (dt, J = 8.02, 0.92, 0.92 Hz, 1H), 3.86 (s, 3H), 3.75 (s, 2H) ; The obtained ^{1}H NMR spectrum corresponded to the reported data.[8]

4'-Nitro-[1,1'-biphenyl]-2-amine: Prepared from 2-bromoaniline (0.931 g, 5.39 mmol), and phenylboronic acid (1.12 g, 6.68 mmol), **S2-10** was obtained as a yellow solid (0.545 g, 2.55 mmol, 43%). ^{1}H NMR (500MHz, $CDCl_3$, ppm): δ = 8.31 (d, J = 8.68 Hz, 1H), 7.67 (d, J = 8.73 Hz, 1H), 7.22 (td, J = 7.78, 7.70, 1.60 Hz, 1H), 7.13 (dd, J = 7.68, 1.58 Hz, 1H), 6.87 (td, J = 7.46, 7.46, 1.14 Hz, 1H), 6.80 (dd, J = 8.0, 1.10 Hz, 1H), 3.75 (s, 1H). ; The obtained ^{1}H NMR spectrum corresponded to the reported data.[12]

[1,1':4',1''-Terphenyl]-2-amine: Prepared from 2-bromoaniline (1.51 g, 8.72 mmol), and 4-biphenylboronic acid (2.59 g, 13.1 mmol), **S2-11** was obtained as a colorless oil (1.66 g, 6.78 mmol, 77%). ^{1}H NMR (500MHz, $CDCl_3$, ppm): δ = 7.70-7.66 (m, 2H), 7.66-7.63 (m, 2H), 7.56-7.53 (m, 2H), 7.49-7.43 (m, 2H), 7.40-7.35 (m, 1H), 7.18 (td, J = 7.18, 6.93, 1.55 Hz, 2H), 6.83 (td, J = 7.43, 7.42, 1.20 Hz, 1H), 6.80 (dd, J = 8.49, 1.25 Hz, 1H), 3.81 (s, 2H) ; The obtained ^{1}H NMR spectrum corresponded to the reported data.[8]

4',5-Dichloro-[1,1'-biphenyl]-2-amine: Prepared from 2-bromo-4-chloroaniline (1.51 g, 7.26 mmol), and 4-chlorophenylboronic acid (1.74 g, 10.9 mmol), **S2-12** was obtained as a white solid (1.03 g, 4.32 mmol, 59%). ^{1}H NMR (500MHz, $CDCl_3$, ppm): δ = 7.45-7.40 (m,2H), 7.39-7.34 (m, 2H), 7.11 (dd, J = 8.51, 2.50 Hz, 1H), 7.06 (d, J = 2.45 Hz, 1H), 6.68 (d, J = 8.48 Hz, 1H), 3.71 (s, 2H); The obtained ^{1}H NMR spectrum corresponded to the reported data.[8]

Methyl 6-amino-[1,1'-biphenyl]-3-carboxylate: Prepared from methyl 4-amino-3-bromobenzoate (2.01 g, 8,69 mmol), and phenylboronic acid (1.59 g, 13.0 mmol), **S2-13** was quantitatively obtained as a white solid. ^{1}H NMR (500MHz, $CDCl_3$, ppm): δ = 7.87-7.78 (m,2H), 7.49-7.41 (m, 4H), 7.40-7.34 (m, 1H), 6.73 (dd, J = 7.97, 0.84 Hz, 1H), 4.18 (s, 2H), 3.85 (s, 3H); The obtained ^{1}H NMR spectrum corresponded to the reported data.[8]

Synthesis of 5-bromo-[1,1'-biphenyl]-2-amine

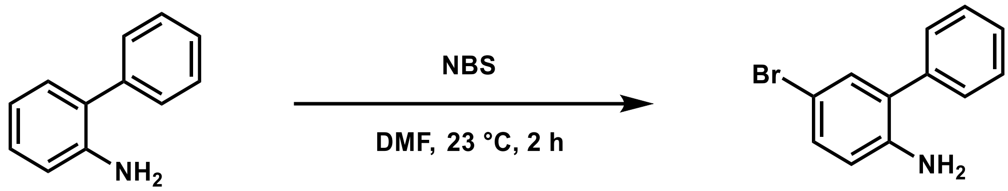

2-Aminobiphenyl (1.50 g, 8.86 mmol) was dissolved in DMF (40 mL), and *N*-bromo succinimide (NBS, 1.893 g, 10.6 mmol) dissolved in DMF (10 mL) was added to the solution at 0 °C. The mixture was reacted at 23 °C for 2 h. The reaction was quenched by adding water (100 mL). After adding CH_2Cl_2 (50 mL), the organic layer was washed with water and brine, then dried over Na_2SO_4. After filtration and removal of the solvent, the residue was purified by silica gel column chromatography using hexane/ethyl acetate (19/1) as an eluent. The desired product, 5-bromo-[1,1'-biphenyl]-2-amine (**S2-3**) was obtained as yellow solid. (1,26 g, 5.06 mmol, 57 %).

5-Bromo-[1,1'-biphenyl]-2-amine: ^{1}H NMR (500MHz, $CDCl_3$, ppm): δ = 7.50-7.34 (m, 6H), 7.29-7.22 (m, 1H), 6.66 (dt, *J* = 8.87, 1.23 Hz, 1H), 3.77 (s, 2H); The obtained ^{1}H NMR spectrum corresponded to the reported data.[8]

Synthesis of 4'-bromo-[1,1'-biphenyl]-2-amine

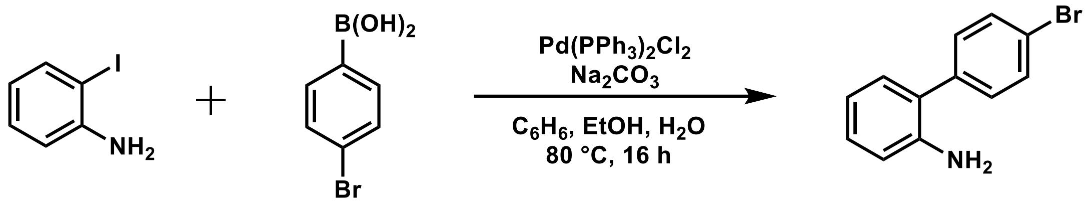

2-Iodoaniline (1.51 g, 6.85 mmol), 4-bromophenylboronic acid (2.06 g, 10.3 mmol), $Pd(PPh_3)_2Cl_2$ (0.439 g, 0.626 mmol), Na_2CO_3 (2.47 g, 23.2 mmol) were dissolved in benzene (60 mL), EtOH (4 mL), and water (12 mL) under a nitrogen atmosphere and the mixture was heated at 80 °C for 16 h under vigorous stirring. After cooling to room temperature and adding ethyl acetate (50 mL), the reaction solution was filtered over celite. The organic layer was separated, and the aqueous layer was extracted with ethyl acetate three times. The organic extracts were washed with brine and dried over Na_2SO_4. After filtration and removal of the solvent, the residue was purified by silica gel column chromatography using hexane/ethyl acetate (19/1). The desired product, 4'-bromo-[1,1'-biphenyl]-2-amine (**S2-5**) was obtained as a white solid (0.763 g, 3.07 mmol, 45 %).

4'-Bromo-[1,1'-biphenyl]-2-amine: ^{1}H NMR (500MHz, $CDCl_3$, ppm): δ = 7.60-7.55 (m, 2H), 7.38-7.31 (m, 2H), 7.20-7.13 (m, 1H), 7.09 (dd, *J* = 7.58, 1.57 Hz, 1H), 6.83 (td, *J* = 7.45, 1.11 Hz, 1H), 6.77 (dd, *J* = 7.91, 1.17 Hz, 1H), 3.72 (s, 2H); The obtained ^{1}H NMR spectrum corresponded to the reported data.[8]

Synthesis of 5-nitro-[1,1'-biphenyl]-2-amine

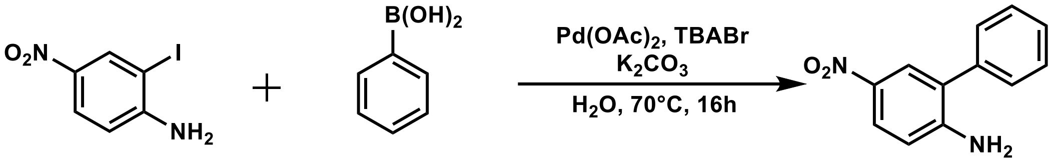

2-Iodoaniline (2.00 g, 7.58 mmol), phenylboronic acid (1.39 g, 11.4 mmol), $Pd(OAc)_2$ (3.40 mg, 0.20 mol%), tetrabutylammonium bromide (2.458 g, 7.58 mmol), K_2CO_3 (2.64 g, 19.1 mmol) were dissolved in water (40 mL) under a nitrogen atmosphere and the mixture was heated at 70 °C for 16 h under vigorous stirring. After cooling to room temperature and adding ethyl acetate (50 mL), the reaction solution was filtered by celite. The organic layer was separated, and the aqueous layer was extracted with ethyl acetate several times. The organic extracts were washed with brine and dried over Na_2SO_4. After filtration and removal of the solvent, the residue was purified by silica gel column chromatography using hexane/ethyl acetate (19/1) as an eluent. The desired product, 5-nitro-[1,1'-biphenyl]-2-amine (**S2-7**) was obtained as white solid. (1.35 g, 6.28 mmol, 82 %).

5-Nitro-[1,1'-biphenyl]-2-amine: ^{1}H NMR (500MHz, $CDCl_3$, ppm): δ = 8.09-8.05 (m, 2H), 7.54-7.47 (m, 2H), 7.45-7.41 (m, 3H), 6.72 (dt, *J* = 3.17, 1.29 Hz, 1H), 4.48 (s, 2H); The obtained ^{1}H NMR spectrum corresponded to the reported data.[8]

2-11. Synthesis of tert-butyl [1,1'-biphenyl]-2-ylcarbamate derivatives ***(2e-i, 2l-o)***

General Procedure for the synthesis of tert-butyl [1,1'-biphenyl]-2-ylcarbamate derivatives

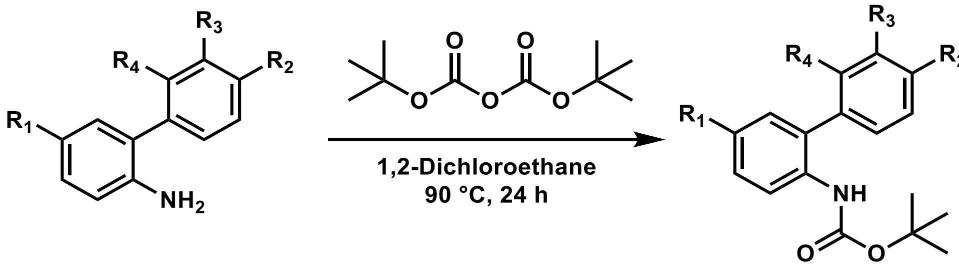

The appropriate substituted [1,1'-biphenyl]-2-amine (1.00 equiv.), di-*tert*-butyl-dicarbonate (5.00 equiv.) were dissolved in 1,2-dichloroethane (0.12 M) under a nitrogen atmosphere and the mixture was heated at 90 °C for 24 h under vigorous stirring. The reaction was quenched by adding 2,2,2-trifluoroethanol (5 equiv.), DMAP (0.5 equiv.). The organic layer was washed with water and brine, then dried over Na_2SO_4. the obtained

residue was purified by silica gel column chromatography using hexane/ethyl acetate (19/1) to afford a spectroscopically pure product.[10]

***tert*-Butyl (5-chloro-[1,1'-biphenyl]-2-yl)carbamate (2e)**: Prepared from 5-chloro-[1,1'-biphenyl]-2-amine (1.12 g, 5.52 mmol), **2e** was obtained as a yellow solid (1.17 g, 3.85 mmol, 70%). ^{1}H NMR (500MHz, $CDCl_3$, ppm): δ = 8.09 (d, J = 8.92 Hz, 1H), 7.50 (ddd, J = 7.65, 6.40, 1.16 Hz, 2H), 7.47-7.42 (m, 1H), 7.37-7.33 (m, 2H), 7.32-7.27 (m, 1H), 7.18 (dd, J = 2.60, 1.08 Hz, 1H), 6.46 (s, 1H), 1.46 (s, 9H); ^{13}C NMR (126 MHz, $CDCl_3$, ppm): δ = 152.83, 137.20, 134.11, 132.88, 130.00, 129.38, 129.25, 128.43, 128.32, 128.05, 121.06, 80.95, 28.40; HRMS (ESI-TOF): calcd for $C_{17}H_{18}ClNNaO_2^+$ ([M+Na]$^+$) m/z 326.0918, found 326.0918.

***tert*-Butyl (5-bromo-[1,1'-biphenyl]-2-yl)carbamate (2f)**: Prepared from 5-bromo-[1,1'-biphenyl]-2-amine (0.91 g, 3.66 mmol), **2f** was obtained as a yellow solid (0.762 g, 2.19 mmol, 60%). ^{1}H NMR (500MHz, $CDCl_3$, ppm): δ = 8.05 (d, J = 8.94 Hz, 1H), 7.49 (td, J = 7.65, 7.29, 1.52 Hz, 2H), 7.47-7.41 (m, 2H), 7.38-7.31 (m, 3H), 6.46 (s, 1H), 1.46 (d, J = 1.4 Hz, 9H); ^{13}C NMR (126 MHz, $CDCl_3$, ppm): δ = 152.76, 137.07, 134.65, 133.16, 132.85, 131.27, 129.39, 129.26, 128.45, 121.28, 115.59, 80.99, 28.40; HRMS (ESI-TOF): calcd for $C_{17}H_{18}BrNNaO_2^+$ ([M+Na]$^+$) m/z 370.0413, found 370.0413.

***tert*-Butyl (4'-chloro-[1,1'-biphenyl]-2-yl)carbamate (2g)**: Prepared from 4'-chloro-[1,1'-biphenyl]-2-amine (1.00 g, 4.91 mmol), **2g** was obtained as a yellow solid (1.21 g, 4.18 mmol, 85%). ^{1}H NMR (500MHz, $CDCl_3$, ppm): δ = 8.07 (d, *J* = 8.33 Hz, 1H), 7.46 (d, *J* = 8.51 Hz, 2H), 7.38-7.27 (m, 3H), 7.16 (dd, *J* = 7.59, 1.71 Hz, 1H), 7.10 (td, *J* = 7.45, 1.20 Hz, 1H), 6.36 (s, 1H), 1.47 (s, 9H); ^{13}C NMR (126 MHz, $CDCl_3$, ppm): δ = 152.98, 136.98, 135.30, 134.00, 130.79, 130.54, 130.21, 129.40, 128.86, 123.44, 120.40, 80.80, 28.42; HRMS (ESI-TOF): calcd for $C_{17}H_{18}ClNNaO_2^+$ ([M+Na]$^+$) m/z 326.0918, found 326.0918.

***tert*-Butyl (4'-bromo-[1,1'-biphenyl]-2-yl)carbamate (2h)**: Prepared from 4'-bromo-[1,1'-biphenyl]-2-amine (0.693 g, 2.79 mmol), **2h** was obtained as a yellow solid (0.533 g, 1.53 mmol, 55%). ^{1}H NMR (500MHz, $CDCl_3$, ppm): δ = 8.07 (d, J = 8.33 Hz, 1H), 7.68-7.57 (m, 2H), 7.35 (ddd, J = 8.88, 7.45, 1.78 Hz, 1H), 7.29-7.22 (m, 2H), 7.16 (dd, J = 7.55, 1.73 Hz, 1H), 7.13-7.09 (m, 1H), 6.35 (s, 1H), 1.47 (s, 9H); ^{13}C NMR (126 MHz, $CDCl_3$, ppm): δ = 152.98, 137.48, 135.24, 132.36, 131.12, 130.53, 130.16, 128.89, 123.46,

122.17, 120.43, 80.82, 28.43; HRMS (ESI-TOF): calcd for $C_{17}H_{18}ClNNaO_2^+$ ($[M+Na]^+$) m/z 370.0413, found 370.0413.

***tert*-Butyl (5-methoxy-[1,1'-biphenyl]-2-yl)carbamate (2i)**: Prepared from 5-methoxy-[1,1'-biphenyl]-2-amine (0.418 g, 2.09 mmol), **2i** was obtained as a white solid (0.393 g, 1.31 mmol, 63%). ^{1}H NMR (500MHz, $CDCl_3$, ppm): δ = 7.88 (s, 1H), 7.52-7.42 (m, 2H), 7.44-7.33 (m, 3H), 6.89 (dd, J = 8.97, 3.00 Hz, 1H), 6.81-6.74 (m, 1H), 6.23 (s, 1H), 3.80 (s, 3H), 1.45 (s, 9H); ^{13}C NMR (126 MHz, $CDCl_3$, ppm): δ = 155.85, 153.59, 138.65, 131.49, 129.31, 129.06, 128.45, 127.91, 122.84, 115.64, 113.66, 80.31, 55.70, 28.45; HRMS (ESI-TOF): calcd for $C_{18}H_{21}NNaO_3^+$ ($[M+Na]^+$) m/z 322.1414, found 322.1414.

***tert*-Butyl (4'-methoxy-[1,1'-biphenyl]-2-yl)carbamate (2l)**: Prepared from 4'-methoxy-[1,1'-biphenyl]-2-amine (0.927 g, 4.65 mmol), **2l** was obtained as a white solid (1.05 g, 3.49 mmol, 75%). ^{1}H NMR (500MHz, $CDCl_3$, ppm): δ = 8.10 (d, J = 8.26 Hz, 1H), 7.30 (ddd, J = 8.79, 6.41, 1.18 Hz, 3H), 7.17 (dt, J = 7.55, 1.28 Hz, 1H), 7.08 (tt, J = 7.43, 1.12 Hz, 1H), 7.04-6.98 (m, 2H), 6.51 (s, 1H), 3.88 (s, 3H), 1.47 (s, 9H); ^{13}C NMR (126 MHz, $CDCl_3$, ppm): δ = 159.34, 153.05, 135.56, 131.12, 130.66, 130.58, 130.40, 128.23, 123.08, 119.72, 114.61, 80.55, 55.49, 28.45; HRMS (ESI-TOF): calcd for $C_{18}H_{21}NNaO_3^+$ ($[M+Na]^+$) m/z 322.1414, found 322.1414.

***tert*-Butyl (4'-nitro-[1,1'-biphenyl]-2-yl)carbamate (2m)**: Prepared from 4'-nitro-[1,1'-biphenyl]-2-amine (0.500 g, 2.33 mmol), **2m** was obtained as a yellow solid (0.459 g, 1.46 mmol, 63%). ^{1}H NMR (500MHz, $CDCl_3$, ppm): δ = 8.38-8.29 (m, 2H), 8.02 (d, J = 8.31 Hz, 1H), 7.61-7.54 (m, 2H), 7.41 (ddd, J = 8.63, 7.19, 1.87 Hz, 1H), 7.23-7.14 (m, 2H), 6.24 (s, 1H), 1.45 (s, 9H); ^{13}C NMR (126 MHz, $CDCl_3$, ppm): δ = 152.90, 147.49, 145.70, 135.08, 130.35, 130.11, 129.78, 124.37, 124.14, 121.66, 90.72, 81.12, 28.39; HRMS (ESI-TOF): calcd for $C_{17}H_{18}N_2NaO_4^+$ ($[M+Na]^+$) m/z 337.1159, found 337.1159.

***tert*-Butyl ([1,1':4',1''-terphenyl]-2-yl)carbamate (2n)**: Prepared from [1,1':4',1''-terphenyl]-2-amine (1.48 g, 6.03 mmol), **2n** was obtained as a white solid (1.67 g, 4.83 mmol, 80%). ^{1}H NMR (500MHz, $CDCl_3$, ppm): δ = 8.12 (d, J = 8.29 Hz, 1H), 7.75-7.70 (m, 2H), 7.69-7.63 (m, 2H), 7.52-7.43 (m, 4H), 7.37 (dtd, J = 15.25, 7.44, 7.37, 1.41 Hz, 2H), 7.26-7.23 (m, 1H), 7.13 (td, J = 7.46, 1.13 Hz, 1H), 6.56 (s, 1H), 1.48 (s, 9H); ^{13}C NMR (126 MHz, $CDCl_3$, ppm): δ = 153.09, 140.71, 140.57, 137.47, 135.45, 131.18, 130.35, 129.86, 129.07, 128.59, 127.87, 127.74, 127.22, 123.31, 120.11, 80.67, 28.46; HRMS (ESI-TOF): calcd for $C_{23}H_{23}NNaO_2^+$ ($[M+Na]^+$) m/z 368.1621, found 368.1621.

***tert*-Butyl (4',5-chloro-[1,1'-biphenyl]-2-yl)carbamate (2o)**: Prepared from 4',5-chloro-[1,1'-biphenyl]-2-amine (0.688 g, 2.88 mmol), **2o** was obtained as a yellow solid (0.645 g, 1.91 mmol, 46%). ^{1}H NMR (500MHz, $CDCl_3$, ppm): δ = 8.06 (d, J = 8.90 Hz, 1H), 7.50-7.42 (m, 2H), 7.30 (tt, J = 7.15, 6.45, 1.68, 1.68 Hz, 3H), 7.15 (dd, J = 2.46, 0.87 Hz, 1H), 6.31 (s, 1H), 1.46 (s, 9H); ^{13}C NMR (126 MHz, $CDCl_3$, ppm): δ = 152.75, 135.62, 134.58, 134.03, 131.85, 130.62, 129.87, 129.60, 128.68, 128.32, 121.52, 81.15, 28.38; HRMS (ESI-TOF): calcd for $C_{17}H_{17}Cl_2NNaO_2^+$ ($[M+Na]^+$) m/z 360.0529, found 360.0529.

Synthesis of tert-butyl (5-nitro-[1,1'-biphenyl]-2-yl)carbamate ***(2j)***

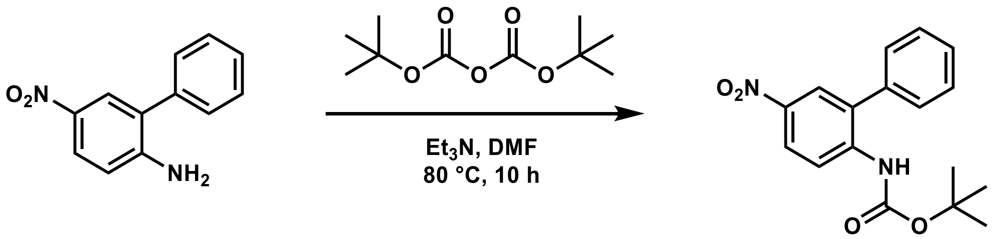

5-Nitro-[1,1'-biphenyl]-2-amine (0.502 g, 2.33 mmol), di-*tert*-butyl-dicarbonate (1.072 mL, 4.67 mmol), and triethylamine (0.976 mL, 7.00 mmol) were dissolved in DMF (2.33 mL) under a nitrogen atmosphere and the mixture was heated at 80 °C for 10 h under vigorous stirring. After cooling to room temperature, the reaction was quenched by adding saturated $NaHCO_3$ aq. solution (30 mL) and ethyl acetate (50 mL). The organic layer was washed with water and brine, then dried over Na_2SO_4. After filtration and removal of the solvent, the residue was purified by silica gel column chromatography using hexane/ethyl acetate (19/1) as an eluent. The desired product, *tert*-butyl (5-nitro-[1,1'-biphenyl]-2-yl)carbamate (**2d-7**) was obtained as yellow solid. (0.135 g, 0.429 mmol, 18%).

***tert*-Butyl (5-nitro-[1,1'-biphenyl]-2-yl)carbamate**: ^{1}H NMR (500MHz, $CDCl_3$, ppm): δ = 8.44 (d, J = 9.20 Hz, 1H), 8.22 (dd, J = 9.19, 2.71 Hz, 1H), 8.09 (d, J = 2.71 Hz, 1H), 7.56 (dd, J = 8.16, 6.59 Hz, 2H), 7.53-7.48 (m, 1H), 7.43-7.33 (m, 2H), 6.85 (s, 1H), 1.48 (s, 9H); ^{13}C NMR (126 MHz, $CDCl_3$, ppm): δ = 152.05, 142.43, 141.74, 136.02, 130.83, 129.82, 129.25, 129.14, 125.89, 124.42, 118.20, 82.10, 28.31; HRMS (ESI-TOF): calcd for $C_{17}H_{18}N_2NaO_4^+$ ($[M+Na]^+$) m/z 337.1159, found 337.1159.

Synthesis of tert-butyl (5-formyl-[1,1'-biphenyl]-2-yl)carbamate ***(2k)***

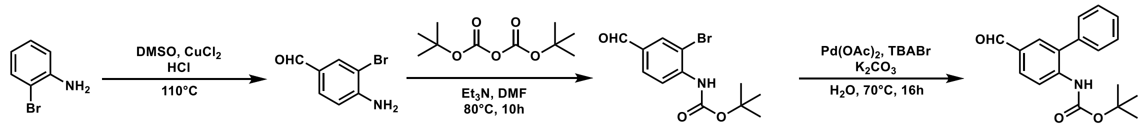

tert-Butyl (5-formyl-[1,1'-biphenyl]-2-yl)carbamate was obtained from 2-bromoaniline in three steps.

2-Bromoaniline (3.50 g, 20 mmol), $CuCl_2$ (5.35 g, 40 mmol) were dissolved in DMSO (150 mL) and concentrated HCl (15 mL) and then the mixture was heated at 90 °C for 8 h under vigorous stirring. After cooling to room temperature, the reaction was quenched by adding 10 wt% NaOH aq. solution (until reaching pH 7). After adding diethyl ether (50 mL), the reaction solution was filtered over celite. The organic layer was separated, and the aqueous layer was extracted with diethyl ether three times. The organic extracts were washed with brine and dried over Na_2SO_4. After filtration and removal of the solvent, the residue was purified by silica gel column chromatography using hexane/ethyl acetate (9/1) an an eluent. The desired product, 4-amino-3-bromobenzaldehyde was obtained as white solid. (0.580 g, 2.90 mmol, 14%).

4-Amino-3-bromobenzaldehyde: ^{1}H NMR (500MHz, $CDCl_3$, ppm): δ = 9.71 (s, 1H), 7.95 (d, J = 1.88 Hz, 1H), 7.64 (dd, J = 8.29, 1.83 Hz, 1H), 6.80 (d, J = 8.29 Hz, 1H), 4.71 (s, 3H); The obtained ^{1}H NMR spectrum corresponded to the reported data.[13]

4-Amino-3-bromobenzaldehyde (0.530 g, 2.65 mmol), di-*tert*-butyl-dicarbonate (2.87 mL, 12.50 mmol), and triethylamine (1.04 mL, 7.50 mmol) were dissolved in 1,2-dichloroethane (20 mL) under a nitrogen atmosphere and the mixture was heated at 90 °C for 24 h under vigorous stirring. After cooling to room temperature, the reaction was quenched by adding 2,2,2-trifluoroethanol (5 equiv.), DMAP (5 equiv.). After adding ethyl acetate (50 mL), the organic layer was washed with water two times and brine two times and dried over Na_2SO_4. After filtration and removal of the solvent, the residue was purified by silica gel column chromatography using hexane/ethyl acetate (9/1) as an eluent. The desired product, *tert*-butyl (2-bromo-4-formylphenyl)carbamate was obtained as a white solid. (0.28 g, 0.933 mmol, 35%).

***tert*-Butyl (2-bromo-4-formylphenyl)carbamate**: ^{1}H NMR (500MHz, $CDCl_3$, ppm): δ = 9.84 (s, 1H), 8.40 (d, J = 8.52 Hz, 1H), 8.05 (d, J = 1.89 Hz, 1H), 7.79 (dd, J = 8.58, 1.89 Hz, 1H), 7.30 (s, 1H), 1.55 (s, 9H); ^{13}C NMR (126 MHz, $CDCl_3$, ppm): δ = 189.81, 151.84, 141.80, 133.48, 131.89, 130.78, 118.94, 112.38, 82.32, 28.32; HRMS (ESI-TOF): calcd for $C_{12}H_{14}BrNNaO_3^+$ ($[M+Na]^+$) m/z 322.0049, found 322.0049.

tert-Butyl (2-bromo-4-formylphenyl)carbamate (0.260 g, 0.866 mmol), phenylboronic acid (1.71 g, 1.40 mmol), $Pd(OAc)_2$ (0.418 g, 2.00 mol%), tetrabutylammonium bromide (0.301 g, 0.934 mmol), K_2CO_3 (0.325 g, 2.35 mmol) were dissolved in water (5 mL) under a nitrogen atmosphere and the mixture was heated at 70 °C for 16 h under vigorous stirring. After cooling to room temperature and adding ethyl acetate (50 mL), the reaction solution was filtered by celite. The organic layer was separated, and the aqueous layer was extracted with ethyl acetate three times. The organic extracts were washed with brine and dried over Na_2SO_4. After filtration and removal of the solvent, the residue was purified by silica gel column chromatography using hexane/ethyl acetate (9/1) as an eluenet. The desired product, *tert*-butyl (5-formyl-[1,1'-biphenyl]-2-yl)carbamate (**2k**) was obtained as white solid (0.187 g, 0.629 mmol, 73%).

***tert*-Butyl (5-formyl-[1,1'-biphenyl]-2-yl)carbamate**: ^{1}H NMR (500MHz, $CDCl_3$, ppm): δ = 9.92 (s, 1H), 8.43 (d, J = 8.58 Hz, 1H), 7.85 (dd, J = 8.59, 2.04 Hz, 1H), 7.71 (d, J = 2.00 Hz, 1H), 7.53 (dd, J = 8.08, 6.71 Hz, 2H), 7.49-7.44 (m, 1H), 7.43-7.32 (m, 2H), 6.82 (s, 1H), 1.47 (s, 9H); ^{13}C NMR (126 MHz, $CDCl_3$, ppm): δ = 191.20, 152.22, 141.34, 136.93, 131.72, 131.03, 130.95, 130.92, 129.59, 129.30, 128.66, 118.41, 81.64, 28.31; HRMS (ESI-TOF): calcd for $C_{18}H_{19}NNaO_3^+$ ([M+Na]$^+$) m/z 320.1257, found 320.1257.

*Synthesis of methyl 6-((tert-butoxycarbonyl)amino)-[1,1'-biphenyl]-3-carboxylate **(2p)***

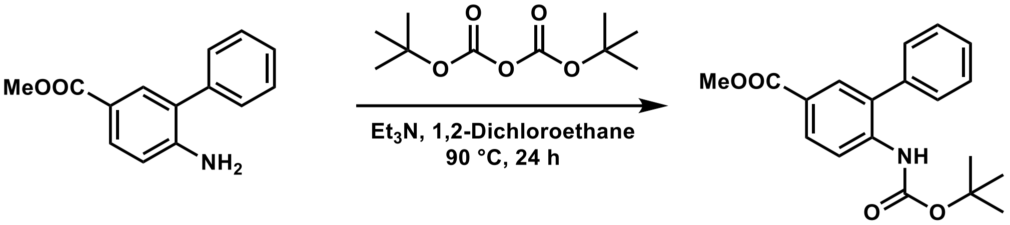

Methyl 6-amino-[1,1'-biphenyl]-3-carboxylate (0.970 g, 4.267 mmol), di-*tert*-butyl-dicarbonate (4.90 mL, 21.33 mmol), and triethylamine (1.78 mL, 12.8 mmol) were dissolved in 1,2-dichloroethane (30 mL) under a nitrogen atmosphere and the mixture was heated at 90 °C for 24 h under vigorous stirring. After cooling to room temperature, the reaction was quenched by adding saturated NH_4Cl aq. solution (30 mL) and ethyl acetate (50 mL). The organic layer was washed with water two times and brine two times and dried over Na_2SO_4. After filtration and removal of the solvent, the residue was purified by silica gel column chromatography using hexane/ethyl acetate (19/1). The desired product, *tert*-butyl methyl 6-((*tert*-butoxycarbonyl)amino)-[1,1'-biphenyl]-3-carboxylate (**2d-13**) was obtained as yellow solid (0.645 g, 1.97 mmol, 46%).

2-12. Synthesis of 9-iodoanthracene ***(Anth-I)***

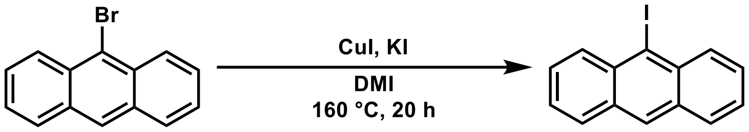

9-Bromoanthracene (2.00 g, 7.778 mmol), CuI (11.1 g, 58.335 mmol), KI (19.36 g, 116.67 mmol) were dissolved in DMI (30 mL) under a nitrogen atmosphere and the mixture was reacted at 70 °C for 20 h under vigorous stirring. After cooling to room temperature, the reaction was quenched by adding CH_2Cl_2, and the reaction solution was filtered. The organic layer was washed with water and brine for several times, and then the organic layer was dried over $MgSO_4$. After filtration and removal of the solvent, the residue was washed with methanol, and purified by silica gel column chromatography using hexane to give a yellow solid (0.7501 g, 32%).

9-Iodoanthracene: ^{1}H NMR (500MHz, $CDCl_3$, ppm): δ = 8.48 (s, 1H), 8.46 (d, J = 6.2 Hz, 2H), 7.96 (d, J = 8.6 Hz, 2H), 7.59 (ddd, J = 8.9, 6,5 1,3 Hz, 2H), 7.50 (ddd, J = 7.8, 6.5, 1.1 Hz, 2H); The obtained ^{1}H NMR spectrum corresponded to the reported data.[14]

2-13. Synthesis of tert-butyl ([1,1'-biphenyl]-2-yl-2',3',4',5',6'-d$_5$*)carbamate* ***(2c-d*$_5$***)***

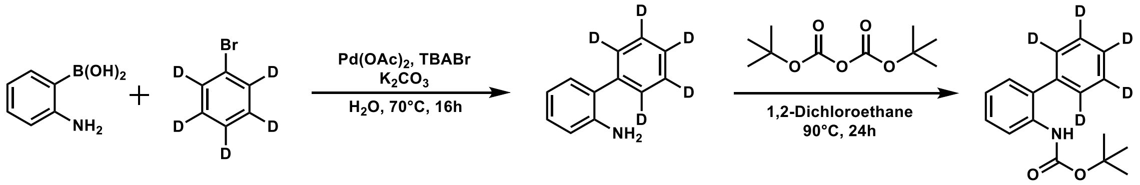

tert-Butyl ([1,1'-biphenyl]-2-yl-2',3',4',5',6'-d$_5$)carbamate was obtained from bromobenzene-d$_5$ in two steps.

Bromobenzene-d$_5$ (0.64 mL, 6.08 mmol), 2-aminophenylboronic acid (1.01 g, 7.30 mmol), $Pd(PPh_3)_4$ (0.352 g, 0.304 mmol), K_2CO_3 (1.68 g, 12.2 mmol) were dissolved in toluene (35 mL), EtOH (15 mL), and water (7.5 mL) under a nitrogen atmosphere and the mixture was heated at 100 °C for 24 h under vigorous stirring. After cooling to room temperature, the reaction solution was filtered over celite, and then adding CH_2Cl_2 (45 mL) and saturated NH_4Cl aq. solution. The organic layer was separated, and the aqueous layer was extracted with CH_2Cl_2 two times. The organic extracts were washed with saturated $NaHCO_3$ aq. solution and brine and dried over Na_2SO_4. After filtration and removal of the solvent, the residue was purified by silica gel column chromatography using hexane/ethyl acetate (19/1) as an eluent. The desired product, [1,1'-biphenyl]-2',3',4',5',6'-d$_5$-2-amine was obtained as a white solid (0.862 g, 4.95 mmol, 81%).

[1,1'-Biphenyl]-2',3',4',5',6'-d5-2-amine: ^{1}H NMR (500MHz, $CDCl_3$, ppm): δ = 7.23-7.05 (m, 2H), 6.86-6.81 (m, 1H), 6.78 (dd, *J* = 8.0, 1.1 Hz, 1H), 3.8 (s, 2H); The obtained ^{1}H NMR spectrum corresponded to the reported data.[15]

[1,1'-Biphenyl]-2',3',4',5',6'-d5-2-amine (0.534 g, 3.07 mmol), di-*tert*-butyl-dicarbonate (3.30 mL, 14.3 mmol), and triethylamine (0.300 mL, 5.74 mmol) were dissolved in 1,2-dichloroethane (25 mL) under a nitrogen atmosphere and the mixture was heated at 90 °C for 24 h under vigorous stirring. After cooling to room temperature, the reaction was quenched by adding 2,2,2-trifluoroethanol (1.03 mL, 14.4 mmol), DMAP (0.175 g, 1.44 mmol). The organic layer was washed with water two times and brine two times and dried over Na_2SO_4. After filtration and removal of the solvent, the residue was purified by silica gel column chromatography using hexane/ethyl acetate (9/1). The desired product, *tert*-butyl ([1,1'-biphenyl]-2-yl-2',3',4',5',6'-d5)carbamate (**2d-d5**) was obtained as a yellow solid (0.502 g, 1.83 mmol, 60%).

***tert*-Butyl ([1,1'-biphenyl]-2-yl-2',3',4',5',6'-d5)carbamate**: ^{1}H NMR (500MHz, $CDCl_3$, ppm): δ = 8.20-8.04 (m, 1H), 7.38-7.32 (m, 1H), 7.20 (dd, *J* = 7.6, 1.7 Hz, 1H), 7.14-7.08 (m, 1H), 6.51 (s, 1H), 1.47 (s, 9H); ^{13}C NMR (126 MHz, $CDCl_3$, ppm): δ = 153.03, 138.32, 135.36, 131.44, 130.33, 129.43, 129.19, 128.99, 128.87, 128.79, 128.68, 128.50, 127.89, 127.38, 123.17, 119.89, 80.59, 28.42; HRMS (ESI-TOF): calcd for $C_{17}H_{14}D_5NNaO_2^+$ ($[M+Na]^+$) m/z 297.1622, found 297.1622.

2-14. Synthesis of tert-butyl deuterium[1,1'-biphenyl]-2-ylcarbamate ***(2c-d1)***

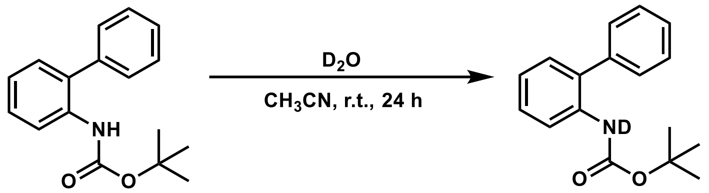

tert-Butyl [1,1'-biphenyl]-2-ylcarbamate (0.1 g, 0.371 mmol) was dissolved in D_2O (10 mL) and CH_3CN (10 mL). The mixture was reacted at room temperature for 24 h. After adding CH_2Cl_2, the organic layer was separated, and the aqueous layer was extracted with CH_2Cl_2 two times. The organic extracts were dried over Na_2SO_4. After filtration and removal of the solvent, the desired product, *tert*-butyl deuterium[1,1'-biphenyl]-2-ylcarbamate (**2c-d1**) was obtained as white solid (0.041 g, 0.152 mmol, 41%).

***tert*-Butyl deuterium[1,1'-biphenyl]-2-ylcarbamate**: ^{1}H NMR (500MHz, $CDCl_3$, ppm): δ = 8.13 (d, *J* = 8.4 Hz, 1H), 7.52-7.46 (m, 2H), 7.45-7.32 (m, 4H), 7.20 (dd, *J* = 7.7, 1.6 Hz, 1H), 7.14-7.05 (m, 1H), 1.47 (s, 7H); ^{13}C NMR (126 MHz, $CDCl_3$, ppm): δ = 152.96, 138.49, 135.27, 131.36, 130.32, 129.40, 129.19, 128.51, 127.89, 123.13, 119.76, 80.59,

28.42; HRMS (ESI-TOF): calcd for $C_{17}H_{18}DNNaO_2^+$ ($[M+Na]^+$) m/z 293.1371, found 293.1371.

*2-15. Synthesis of 9-mesityl-10-methylanthracene (**1i**)*

9-Mesityl-10-methylanthracene (**1i**) was synthesized by the two steps written below.

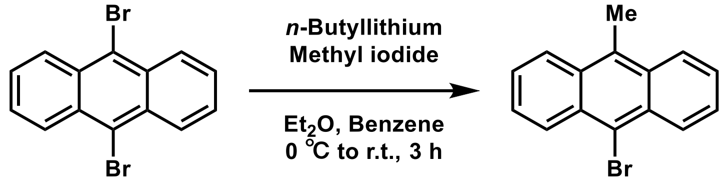

9,10-Dibromoanthracene (1.68 g, 5.0 mmol) was dissolved in Et_2O (10 mL) and benzene (10 mL) under nitrogen atmosphere at 0 °C. *n*-Buthyllithium (1.6 M in hexane, 3.3 mL, 5.0 mmol) was added to the solution by dropwise and the mixture was stirred under N_2 atmosphere at 0 °C for 1 h. After the addition of methyl iodide (1.07 g, 7.5 mmol), The reaction solution was reacted at room temperature for 3 h. The reaction was quenched by saturated NH_4Cl aq. (20 mL). The organic layer was separated, and the aqueous layer was extracted with toluene twice. The combined organic layer was washed with water, and then the organic layer was dried over Na_2SO_4. After filtration and removal of the solvent, the residue was purified by silica gel column chromatography using hexane and ethyl acetate to give 9-Bromo-10-methylanthracene as a yellowish brown solid (935.0 mg, 69%).

9-Bromo-10-methylanthracene: ^1H NMR (500MHz, $CDCl_3$, ppm): δ = 8.60 (d, *J* = 8.9 Hz, 2H), 8.33 (d, *J* = 8.7 Hz, 2H), 7.63 – 7.59 (m, 2H), 7.58 – 7.53 (m, 2H), 3.11 (s, 2H); The obtained ^1H NMR spwctrum corresponded to the repotyted data.[16]

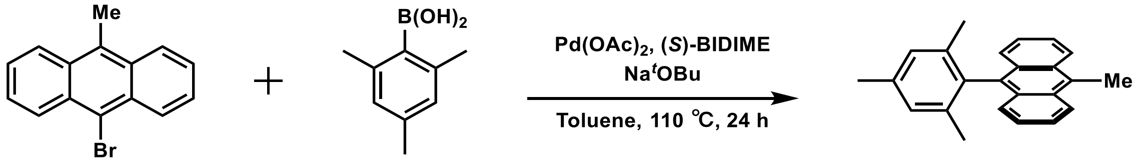

9-Bromo-10-methylanthracene (272.1 mg, 1.00 mmol), mesitylboronic acid (246.3 mg, 1.5 mmol), $Pd(OAc)_2$ (6.6 mg, 0.03 mmol), (*S*)-BIDIME (19.8 mg, 0.06 mmol), and NaO^{t}Bu (287.6 mg, 2.99 mmol) were dissolved in toluene (7 mL) under a nitrogen atmosphere, and the mixture was heated at 110 °C for 24 h under vigorous stirring. After cooling to room temperature, H_2O (10 mL) was added to the solution and the reaction mixture was extracted with CH_2Cl_2 (10 mL). The organic extracts were washed with brine and dried over Na_2SO_4. After filtration and removal of the solvent, the obtained residue

was purified by preparative thin layer chromatography twice using hexane to give compound **1i** as a reddish brown solid (23.6 mg, 8%).

9-Mesityl-10-methylanthracene (1i): ^{1}H NMR (500MHz, $CDCl_3$, ppm): δ = 8.36 (d, *J* = 8.5 Hz, 2H), 7.53 – 7.47 (m, 4H), 7.35 – 7.28 (m, 2H), 7.08 (s, 2H), 3.18 (s, 2H), 2.45 (s, 2H), 1.69 (s, 5H); ^{13}C NMR (126 MHz, $CDCl_3$, ppm): δ = 137.85, 137.13, 135.15, 134.35, 130.17, 129.73, 129.60, 128.35, 126.83, 125.20, 125.14, 77.42, 77.16, 76.91, 21.39, 20.16, 14.28; HRMS (APCI): calcd for $C_{24}H_{22}^{+}$ ($[M+H]^{+}$) m/z 311.1794, found 331.1787.

*2-16. Synthesis of 9-(3,5-bis(trifluoromethyl)phenyl)-10-methylanthracene (**1j**)*

9-(3,5-bis(trifluoromethyl)phenyl)-10-methylanthracene (**1j**) was synthesized by the two steps written below using 9-bromo-10-methylanthracene synthesized with the previous procedure (*2-13*).

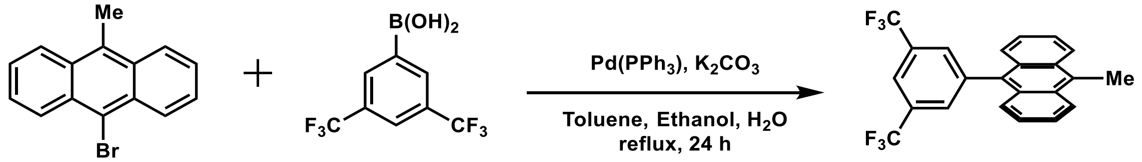

9-Bromo-10-methylanthracene (270.9 mg, 0.999 mmol) , (3,5-bis(trifluoromethyl)phenyl)boronic acid (386.9 mg, 1.5 mmol) and $Pd(PPh_3)_4$ (60 mg, 0.05 mmol) were dissolved in toluene (9 mL) , ethanol (2 mL) and 4.9 M K_2CO_3 aq. (2 mL) under nitrogen atmosphere, and the mixture was refluxed for 24 h under vigorous stirring. After cooling to room temperature, the reaction solution was extracted with CH_2Cl_2. The combined organic layer was washed with brine and dried over Na_2SO_4. After filtration and removal of the solvent, the obtained residue was purified by silica gel column chromatography, eluting with hexane until the desired product was obtained, and then with CH_2Cl_2 to give compound **1j** as a yellow solid (392.2 mg, 97%).

9-(3,5-bis(trifluoromethyl)phenyl)-10-methylanthracene (1j): ^{1}H NMR (500MHz, $CDCl_3$, ppm): δ = 8.40 (d, *J* = 9.0 Hz, 2H), 8.07 (s, 1H), 7.90 (s, 2H), 7.57 – 7.53 (m, 2H), 7.46 – 7.39 (m, 4H), 3.21 (s, 3H); ^{13}C NMR (126 MHz, $CDCl_3$, ppm): δ = 142.00, 132.23 (d, *J* = 6.4 Hz), 131.99, 131.77, 129.87 (d, *J* = 8.3 Hz), 128.47, 127.88, 126.50, 126.03, 125.34 (d, *J* = 21.1 Hz), 124.66, 122.52 (d, *J* = 8.2 Hz), 121.38, 14.54; HRMS (APCI): calcd for $C_{23}H_{14}F_6^{\cdot+}$ ($M^{\cdot+}$) m/z 404.0994, found 404.0988.

Methyl 6-((*tert*-butoxycarbonyl)amino)-[1,1'-biphenyl]-3-carboxylate: ^{1}H NMR (500MHz, $CDCl_3$, ppm): δ = 8.31 (d, J = 8.71 Hz, 1H), 8.01 (dd, J = 8.73, 2.12 Hz, 1H), 7.88 (d, J = 2.08 Hz, 1H), 7.55-7.48 (m, 2H), 7.47-7.42 (m, 1H), 7.39-7.36 (m, 2H), 6.74

(s, 1H), 3.89 (s, 3H), 1.47 (s, 9H); ^{13}C NMR (126 MHz, $CDCl_3$, ppm): δ = 166.90, 152.42, 139.85, 137.39, 131.88, 130.44, 130.26, 129.47, 129.40, 128.43, 124.21, 118.17, 81.35, 52.11, 28.36; HRMS (ESI-TOF): calcd for $C_{19}H_{21}NNaO_4^+$ ([M+Na]$^+$) m/z 350.1363, found 350.1363.

2-17. General procedure for electrocatalytic intramolecular C-N coupling

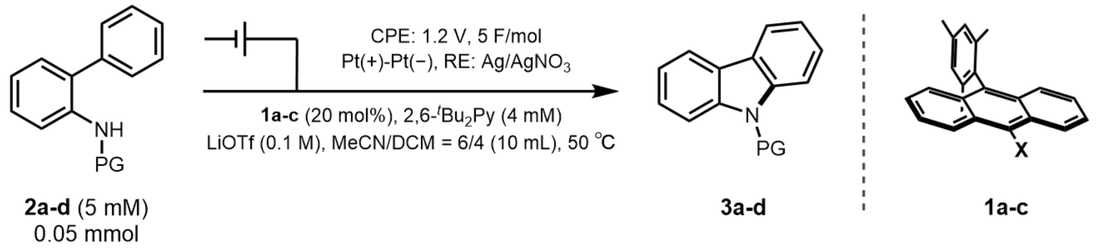

2-Aminobiphenyl derivative (**2a-d**, 0.05 mmol, 5 mM), 9-halo-10-mesitylanthracene (**1a-c**, 0.01 mmol, 1 mM), and 2,6-Di-*tert*-butylpyridine were dissolved in a 10 mL of electrolyte solution (0.1 M LiTfO in MeCN/CH_2Cl_2 (6:4 in vol.)). The electrolysis was performed in a three-electrode setup in an undivided cell. Platinum plates were used as an anode (2 cm × 2 cm) and a cathode (2 cm × 2 cm). Ag/$AgNO_3$ was used as a reference electrode. The electrolysis was performed by applying a constant potential of 1.2 V under vigorous stirring. After the electrolysis, an electrolyte solution was collected, and the solvent was removed under reduced pressure. The resulting mixture was then extracted with CH_2Cl_2, and insoluble LiOTf was removed by filtration to obtain a crude product. The yield was determined by ^{1}H NMR yield.

2-18. General procedure for mediator applicability of N-Boc-aminobiphenyl

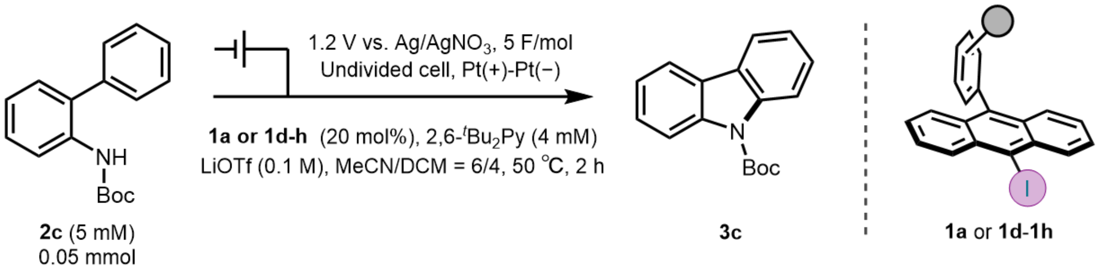

tert-Butyl [1,1'-biphenyl]-2-ylcarbamate (**2c**, 0.05 mmol, 5 mM), 9-iodo-10-mesitylanthracene (**1a** and **1d-h**, 0.01 mmol, 1 mM), and 2,6-di-*tert*-butylpyridine (^{t}Bu$_2$Py, 0.04 mmol, 4 mM) were dissolved in a 10 mL electrolyte solution (0.1 M LiTfO in MeCN/CH_2Cl_2 (6:4 in vol.)). The electrolysis was performed in a three-electrode setup in a divided cell. Platinum plates were used as an anode (2 cm × 2 cm) and a cathode (2 cm × 2 cm). Ag/$AgNO_3$ was used as a reference electrode. The electrolysis was performed

by applying a constant potential of 1.2 V, and 5.0 F/mol of charge was passed under vigorous stirring. After the electrolysis, an electrolyte solution was collected, and the solvent was removed under reduced pressure. The resulting mixture was then extracted with CH_2Cl_2, and insoluble LiOTf was removed by filtration to obtain a crude product. The yield was determined by ^{1}H NMR.

2-19.General procedure for substrate applicability of N-Boc- aminobiphenyls

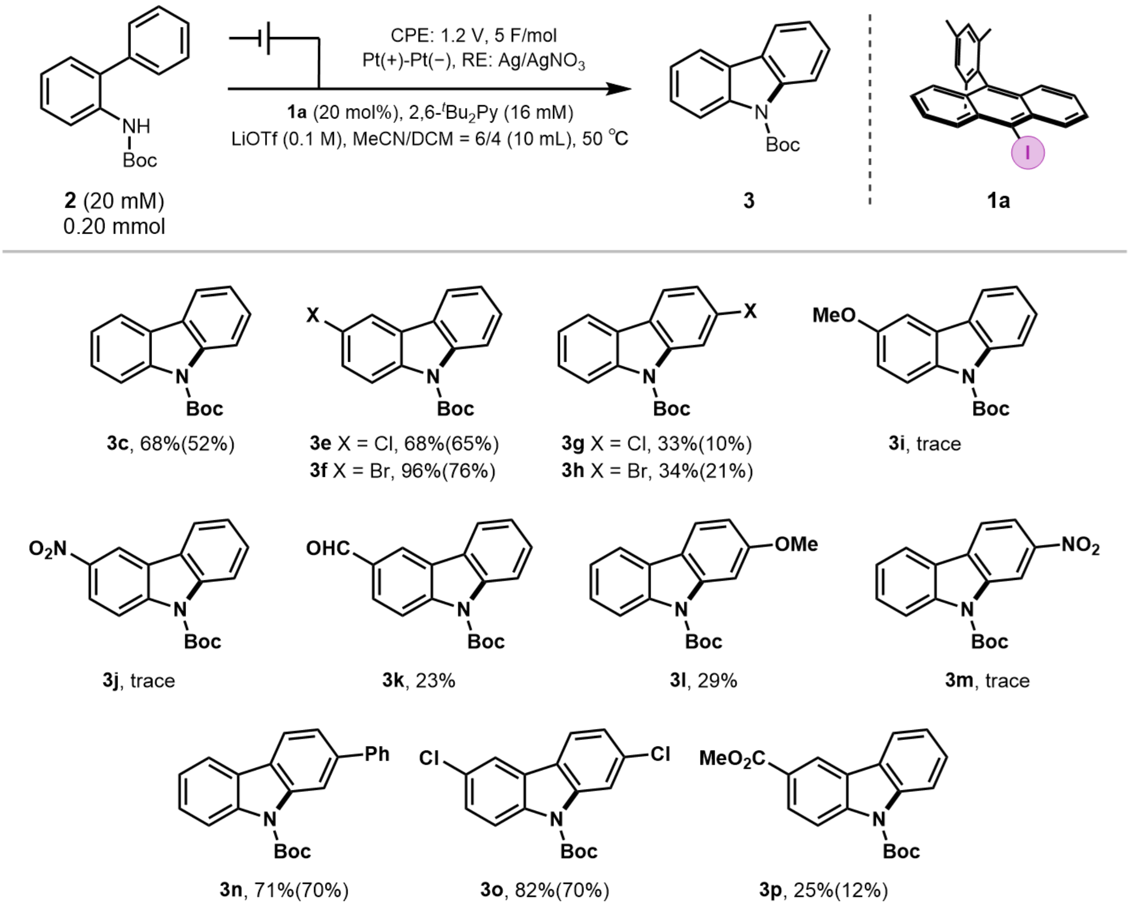

tert-Butyl [1,1'-biphenyl]-2-ylcarbamate derivative (**2c** and **2e-p**, 0.2 mmol, 20 mM), 9-iodo-10-mesitylanthracene (**1a**, 0.04 mmol, 4 mM), and 2,6-di-*tert*-butylpyridine (^{t}Bu$_2$Py, 0.16 mmol, 16 mM) were dissolved in a 10 mL electrolyte solution (0.1 M LiTfO in MeCN/CH_2Cl_2 (6:4 in vol.)). The electrolysis was performed in a three-electrode setup in an undivided cell. Platinum plates were used as an anode (2 cm × 2 cm) and a cathode (2 cm × 2 cm). Ag/$AgNO_3$ was used as a reference electrode. The electrolysis was performed by applying a constant potential of 1.2 V, and 5.0 F/mol of charge was passed under vigorous stirring. After the electrolysis, an electrolyte solution was collected, and the solvent was removed under reduced pressure. The resulting mixture was then extracted with CH_2Cl_2 to obtain a crude product and determine yield by ^{1}H NMR. In case the

carbazole derivative is a new compound, the obtained crude product was purified by silica gel preparative thin-layer chromatography using hexane/ethyl acetate (4/1) to afford a spectroscopically pure product.

***tert*-Butyl 9*H*-carbazole-9-carboxylate (3c)**: Prepared from **2c** (53.8 mg). ^{1}H NMR yield: 68%; Isolated yield: 52% (27.8 mg); ^{1}H NMR (500MHz, $CDCl_3$, ppm): δ = 8.32 (d, *J* = 8.31 Hz, 2H), 7.99 (ddd, *J* = 7.69, 1.32, 0.70 Hz, 2H), 7.48 (ddd, *J* = 8.45, 7.22, 1.33 Hz, 2H), 7.36 (td, *J* = 7.44, 7.38, 1.00 Hz, 2H), 1.78 (s, 9H); The obtained ^{1}H NMR spectrum corresponded to the reported data.[15]

***tert*-Butyl 3-chloro-9*H*-carbazole-9-carboxylate (3e)**: Prepared from **2e** (65.5 mg). ^{1}H NMR yield: 68%; Isolated yield: 65% (42.4 mg); ^{1}H NMR (500MHz, $CDCl_3$, ppm): δ = 8.27 (d, *J* = 8.39 Hz, 1H), 8.24 (d, *J* = 8.87 Hz, 1H), 7.93-7.89 (m, 2H), 7.49 (ddd, *J* = 8.55, 7.20, 1.30 Hz, 1H), 7.41 (dd, *J* = 8.86, 2.18 Hz, 1H), 7.35 (td, *J* = 7.47, 7.25, 0.91 Hz, 1H), 1.76 (s, 9H); ^{13}C NMR (126 MHz, $CDCl_3$, ppm): δ = 150.94, 138.98, 136.98, 128.68, 127.87, 127.21, 127.10, 124.78, 123.33, 119.88, 119.48, 117.48, 116.50, 84.43, 28.48; HRMS (ESI-TOF): calcd for $C_{17}H_{16}ClNNaO_2^+$ ([M+Na]$^+$) m/z 324.0762, found 324.0762.

***tert*-Butyl 3-bromo-9*H*-carbazole-9-carboxylate (3f)**: Prepared from **2f** (70.2 mg). ^{1}H NMR yield: 96%; Isolated yield: 76% (52.7 mg); ^{1}H NMR (500MHz, $CDCl_3$, ppm): δ = 8.27 (d, *J* = 8.41 Hz, 1H), 8.18 (d, *J* = 9.00 Hz, 1H), 8.05 (t, *J* = 1.65 Hz, 1H), 7.93-7.87 (m, 1H), 7.54 (dt, *J* = 8.86, 1.73, 1.73 Hz, 1H), 7.49 (ddt, *J* = 8.73, 7.29, 1.41, 1.41 Hz, 1H), 7.39-7.32 (m, 1H), 1.77 (d, *J* = 1.5 Hz, 9H); ^{13}C NMR (126 MHz, $CDCl_3$, ppm): δ = 150.90, 138.80, 137.35, 129.82, 127.88, 127.66, 124.63, 123.34, 122.48, 119.85, 117.87, 116.46, 116.23, 84.44, 28.47; HRMS (ESI-TOF): calcd for $C_{17}H_{16}BrNNaO_2^+$ ([M+Na]$^+$) m/z 368.0257, found 368.0257.

***tert*-Butyl 2-chloro-9*H*-carbazole-9-carboxylate (3g)**: Prepared from **2g** (60.7 mg). ^{1}H NMR yield: 33%; Isolated yield: 10% (6.2 mg); ^{1}H NMR (500MHz, $CDCl_3$, ppm): δ = 8.37 (s, 1H), 8.27 (d, *J* = 8.39 Hz, 1H), 7.94 (d, *J* = 7.68 Hz, 1H), 7.88 (d, *J* = 8.23 Hz, 1H), 7.48 (ddt, *J* = 8.44, 7.26, 1.20, 1.20 Hz, 1H), 7.39-7.28 (m, 2H), 1.77 (s, 9H); ^{13}C NMR (126 MHz, $CDCl_3$, ppm): δ = 150.92, 139.14, 138.79, 132.86, 127.45, 125.15, 124.41, 123.56, 123.43, 120.38, 119.71, 116.86, 116.47, 84.65, 28.47; HRMS (ESI-TOF): calcd for $C_{17}H_{16}ClNNaO_2^+$ ([M+Na]$^+$) m/z 324.0762, found 324.0762.

***tert*-Butyl 2-bromo-9*H*-carbazole-9-carboxylate (3h)**: Prepared from **2h** (71.0 mg). ^{1}H NMR yield: 34%; Isolated yield: 21% (14.7 mg); ^{1}H NMR (500MHz, $CDCl_3$, ppm): δ = 8.54 (s, 1H), 8.27 (d, *J* = 8.37 Hz, 1H), 7.98-7.93 (m, 1H), 7.82 (dd, *J* = 8.23, 1.09 Hz, 1H), 7.52-7.46 (m, 2H), 7.39-7.33 (m, 1H), 1.77 (s, 9H); ^{13}C NMR (126 MHz, $CDCl_3$, ppm): δ = 150.90, 139.36, 138.62, 127.60, 126.31, 125.43, 125.16, 124.80, 123.44, 120.77, 120.72, 119.74, 116.46, 84.67, 28.47; HRMS (ESI-TOF): calcd for $C_{17}H_{16}BrNNaO_2^+$ ($[M+Na]^+$) m/z 368.0257, found 368.0257.

***tert*-Butyl 3-formyl-9*H*-carbazole-9-carboxylate (3k)**: Prepared from **2k** (61.4 mg). ^{1}H NMR yield: 23%; The obtained ^{1}H NMR spectrum corresponded to the reported data.[17]

***tert*-Butyl 2-methoxy-9*H*-carbazole-9-carboxylate (3l)**: Prepared from **2l** (60.6 mg). ^{1}H NMR yield: 29%; The obtained ^{1}H NMR spectrum corresponded to the reported data.[18]

***tert*-Butyl 2-phenyl-9*H*-carbazole-9-carboxylate (3n)**: Prepared from **2n** (70.0 mg). ^{1}H NMR yield: 71%; Isolated yield: 70% (48.7 mg); ^{1}H NMR (500MHz, $CDCl_3$, ppm): δ = 8.63 (s, 1H), 8.34 (d, *J* = 8.32 Hz, 1H), 8.06-7.98 (m, 2H), 7.77-7.69 (m, 2H), 7.62 (dd, *J* = 7.99, 1.60 Hz, 1H), 7.49 (td, *J* =7.95, 1.74, 3H), 7.42-7.34 (m, 2H), 1.80 (s, 9H); ^{13}C NMR (126 MHz, $CDCl_3$, ppm): δ = 151.26, 141.88, 140.47, 139.26, 139.07, 128.96, 127.61, 127.36, 127.20, 125.68, 125.08, 123.25, 122.53, 119.91, 119.76, 116.46, 115.27, 84.10, 28.54; HRMS (ESI-TOF): calcd for $C_{23}H_{21}NNaO_2^+$ ($[M+Na]^+$) m/z 366.1465, found 366.1465.

***tert*-Butyl 2,6-dichloro-9*H*-carbazole-9-carboxylate (3o)**: Prepared from **2o** (68.6 mg). ^{1}H NMR yield: 82%; Isolated yield: 70% (47.9 mg); ^{1}H NMR (500MHz, $CDCl_3$, ppm): 8.30 (s, 1H), 8.17 (d, *J* = 8.87 Hz, 1H), 7.83 (d, *J* = 2.16 Hz, 1H), 7.77 (d, *J* = 8.26 Hz, 1H), 7.43-7.35 (m, 1H), 7.31 (dd, *J* = 8.26, 1.88 Hz, 1H), 1.77 (s, 9H); ^{13}C NMR (126 MHz, $CDCl_3$, ppm): δ = 150.53, 139.40, 137.06, 133.59, 129.01, 127.36, 126.39, 123.80, 123.23, 120.51, 119.45, 117.50, 116.91, 85.06, 28.42; HRMS (ESI-TOF): calcd for $C_{17}H_{15}Cl_2NNaO_2^+$ ($[M+Na]^+$) m/z 358.0372, found 358.0372.

9-(*tert*-Butyl) 3-methyl *9H*-carbazole-3,9-dicarboxylate (3p): Prepared from **2p** (65.7 mg). ^{1}H NMR yield: 27%; Isolated yield: 12% (7.8 mg); ^{1}H NMR (500MHz, $CDCl_3$, ppm): δ = 8.69 (dd, *J* = 1.73, 0.60 Hz, 1H), 8.36 (d, *J* = 8.73 Hz, 1H), 8.30 (d, *J* = 8.41 Hz, 1H), 8.17 (dd, *J* = 8.79, 1.79 Hz, 1H), 8.05 (dt, *J* = 7.61, 1.07, 1.07 Hz, 1H), 7.51 (ddd, *J* = 8.48, 7.30, 1.33 Hz, 1H), 7.40 (td, *J* = 7.52, 7.48, 0.98 Hz, 1H), 3.99 (s, 3H),

1.77 (s, 9H); ^{13}C NMR (126 MHz, $CDCl_3$, ppm): δ = 167.41, 150.94, 141.58, 139.14, 128.61, 127.84, 125.89, 125.44, 124.98, 123.62, 121.72, 120.07, 116.52, 116.07, 84.78, 52.31, 28.49; HRMS (ESI-TOF): calcd for $C_{19}H_{19}NNaO_4^+$ ($[M+Na]^+$) m/z 348.1206, found 348.1206.

2-20. Refinement details

CCDC deposition numbers 2481846 for **1a**$^{•+}SbCl_6^-$, 2481847 for **1b**$^{•+}SbCl_6^-$ · $0.5CH_2Cl_2$, and 2481848 for **1c**$^{•+}SbCl_6$ contain the supplementary crystallographic data for this paper. These data can be obtained free of charge from The Cambridge Crystallographic Data Centre via www.ccdc.cam.ac.uk/data_request/cif.

Preparation of single crystals of radical cation salt 1a$^{•+}SbCl_6^-$

To a solution of **1a** (7.83 mg, 18.5 μmol) in dry CH_2Cl_2 (0.5 mL) was added tris(2,4-dibromophenyl)ammoniumyl hexachloroantimonate (19.3 mg, 18.3 μmol) at 0 °C to generate a deep blue solution, and the mixture was stirred at 0 °C for 5 min. The addition of dry Et_2O led to precipitation of the radical cation salt. The solvent was decantated and the resulting precipitates were washed with dry Et_2O three times, and dried in vacuo to give **1a**$^{•+}SbCl_6^-$ (10.4 mg) as a dark blue powder (theoretical yield: 14.0 mg). Single crystals of **1a**$^{•+}SbCl_6^-$ suitable for X-ray analysis were obtained as a dark blue plate by recrystallization from CH_2Cl_2/Et_2O with trifluoroacetic anhydride (TFAA) at −20 °C.

Preparation of single crystals of radical cation salt 1b$^{•+}SbCl_6^-$

To a solution of **1b** (7.62 mg, 20.3 μmol) in dry CH_2Cl_2 (0.4 mL) was added tris(2,4-dibromophenyl)ammoniumyl hexachloroantimonate (21.3 mg, 20.2 μmol) at 0 °C to generate a deep blue solution, and the mixture was stirred at 0 °C for 5 min. The addition of dry Et_2O led to precipitation of the radical cation salt. The solvent was decantated and the resulting precipitates were washed with dry Et_2O three times, and dried in vacuo to give **1b**$^{•+}SbCl_6^-$ (6.0 mg) as a dark blue powder (theoretical yield: 14.4 mg). Single crystals of **1b**$^{•+}SbCl_6^-$ · $0.5CH_2Cl_2$ suitable for X-ray analysis were obtained as a dark blue plate by recrystallization from CH_2Cl_2/Et_2O with TFAA at −20 °C.

Preparation of single crystals of radical cation salt 1c$^{•+}SbCl_6^-$

To a solution of **1c** (14.5 mg, 43.8 μmol) in dry CH_2Cl_2 (0.5 mL) was added tris(2,4-dibromophenyl)ammoniumyl hexachloroantimonate (46.1 mg, 43.8 μmol) at 0 °C to generate a deep green solution, and the mixture was stirred in the dark at 0 °C for 5 min. The addition of dry Et_2O led to precipitation of the radical cation salt. The solvent was

decantated and the resulting precipitates were washed with dry Et_2O three times, and dried in vacuo to give **1c**$^{\bullet+}SbCl_6^-$ (21.9 mg) as a dark green powder (theoretical yield: 29.2 mg). Single crystals of **1c**$^{\bullet+}SbCl_6^-$ suitable for X-ray analysis were obtained as a dark green block by recrystallization from CH_2Cl_2/hexane with TFAA at −20 °C.

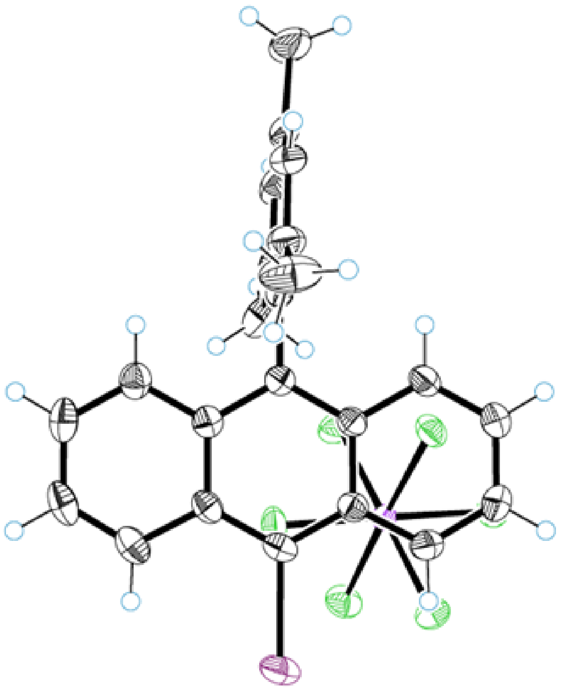

Figure S1. X-ray structure of **1a**$^{\bullet+}$**SbCl**$_6^-$ with thermal anisotropic displacement ellipsoids shown at the 50% probability level.

Table S1. Crystal and refinement data for **1a**$^{\bullet+}$**SbCl**$_6^-$

	**1a**$^{\bullet+}$**SbCl**$_6^-$
CCDC Number	2481846
Empirical formula	$C_{23}H_{19}Cl_6SbI$
Formula weight	756.73
Temperature/K	150
Crystal system	monoclinic
Space group	$P2_1/n$
a/Å	17.50462(12)
b/Å	18.37907(12)
c/Å	18.08569(13)
α/°	90
β/°	115.3171(9)
γ/°	90
Volume/Å^3	5259.66(7)

Z	8
$\rho_{calc}g/cm^3$	1.911
μ/mm^{-1}	23.229
F(000)	2904.0
Crystal size/mm^3	0.23 × 0.164 × 0.034
Radiation	CuKα (λ = 1.54184)
2Θ range for data collection/°	5.882 to 154.824
Index ranges	-19 ≤ h ≤ 21, -23 ≤ k ≤ 22, -22 ≤ l ≤ 22
Reflections collected	51706
Independent reflections	10754 [R_{int} = 0.0382, R_{sigma} = 0.0258]
Data/restraints/parameters	10754/0/566
Goodness-of-fit on F^2	1.050
Final R indexes [I>=2σ (I)]	R_1 = 0.0239, wR_2 = 0.0625
Final R indexes [all data]	R_1 = 0.0259, wR_2 = 0.0636
Largest diff. peak/hole / e $Å^{-3}$	0.80/-0.66

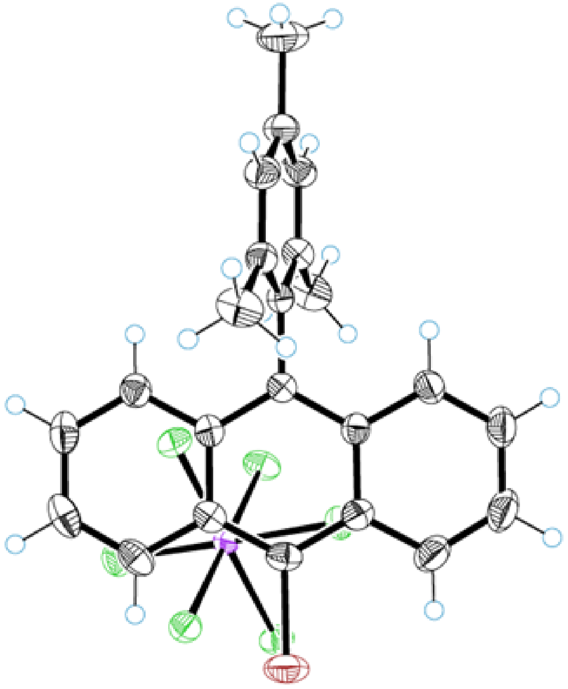

Figure S2. X-ray structure of **$1b^{\cdot+}SbCl_6^-$** with thermal anisotropic displacement ellipsoids shown at the 50% probability level. Solvent molecule is omitted for clarity.

Table S2. Crystal and refinement data for **$1b^{\cdot+}SbCl_6^- \cdot 0.5CH_2Cl_2$**

	**$1b^{\cdot+}SbCl_6^- \cdot 0.5CH_2Cl_2$**
CCDC Number	2481847
Empirical formula	$C_{23.5}H_{20}Cl_7BrSb$
Formula weight	752.20

Temperature/K	150
Crystal system	triclinic
Space group	*P*-1
a/Å	8.13793(9)
b/Å	13.01481(14)
c/Å	13.52022(14)
α/°	79.1751(9)
β/°	77.8760(9)
γ/°	85.0878(9)
Volume/Å^3	1373.53(3)
Z	2
ρ_{calc}g/cm^3	1.819
μ/mm^{-1}	16.032
F(000)	732.0
Crystal size/mm^3	0.214 × 0.151 × 0.061
Radiation	CuKα (λ = 1.54184)
2Θ range for data collection/°	6.922 to 155.01
Index ranges	-10 ≤ h ≤ 10, -16 ≤ k ≤ 16, -17 ≤ l ≤ 15
Reflections collected	25851
Independent reflections	5654 [R_{int} = 0.0476, R_{sigma} = 0.0272]
Data/restraints/parameters	5654/0/302
Goodness-of-fit on F^2	1.068
Final R indexes [I>=2σ (I)]	R_1 = 0.0262, wR_2 = 0.0700
Final R indexes [all data]	R_1 = 0.0266, wR_2 = 0.0704
Largest diff. peak/hole / e Å^{-3}	0.95/-0.84

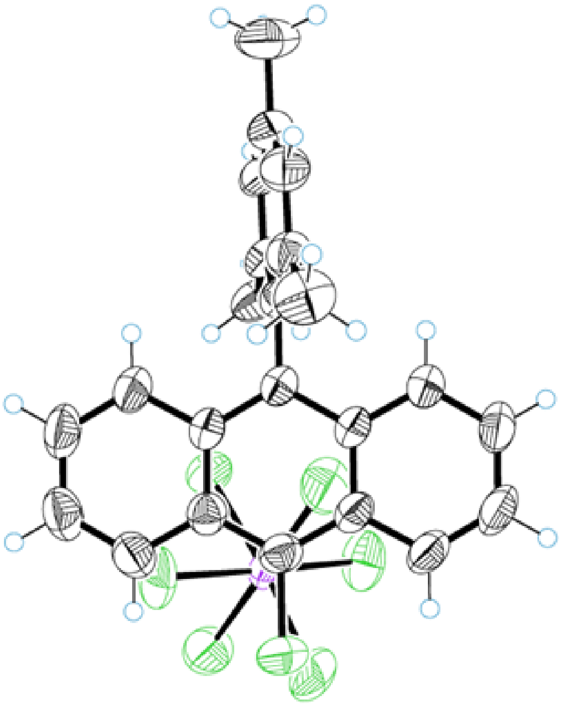

Figure S3. X-ray structure of $\mathbf{1c^{\cdot+}SbCl_6^-}$ with thermal anisotropic displacement ellipsoids shown at the 50% probability level.

Table S3. Crystal and refinement data for $\mathbf{1c^{\cdot+}SbCl_6^-}$

	$\mathbf{1c^{\cdot+}SbCl_6^-}$
CCDC Number	2481848
Empirical formula	$C_{23}H_{19}Cl_7Sb$
Formula weight	665.28
Temperature/K	200
Crystal system	monoclinic
Space group	*C*2/*c*
a/Å	14.86948(9)
b/Å	16.45086(11)
c/Å	21.94122(16)
α/°	90
β/°	99.4923(7)
γ/°	90
Volume/Å^3	5293.68(6)
Z	8
ρ_{calc}g/cm^3	1.670
μ/mm^{-1}	14.849
F(000)	2616.0
Crystal size/mm^3	0.3 × 0.054 × 0.036
Radiation	CuKα (λ = 1.54184)
2Θ range for data collection/°	8.076 to 154.928
Index ranges	-18 ≤ h ≤ 18, -20 ≤ k ≤ 20, -25 ≤ l ≤ 27
Reflections collected	50781
Independent reflections	5479 [R_{int} = 0.0319, R_{sigma} = 0.0146]
Data/restraints/parameters	5479/2/304
Goodness-of-fit on F^2	1.073
Final R indexes [I>=2σ (I)]	R_1 = 0.0270, wR_2 = 0.0712
Final R indexes [all data]	R_1 = 0.0292, wR_2 = 0.0724
Largest diff. peak/hole / e Å^{-3}	0.64/-0.59

3. Supporting data

3-1. Cyclic voltammetry analysis

*3-1-1. Cyclic voltammetry of 9-iodoanthracene (**Anth-I**) and 9-iodophenanthrene (**Phen-I**) and 1-iodopyrene (**Pyr-I**)*

CV measurement of **Anth-I** and **Phen-I** and **Pyr-I** with tetrabutylammonium tetrakis(pentafluorophenyl)borate ($Bu_4NB(C_6F_5)_4$) as the supporting electrolyte, desired reversible voltammogram was observed when **Anth-I** was used (Figure S1). This result indicates that the radical cation state of **Anth-I** is the most stable in these three π-extended structures, and anthracene is a suitable structure for mediator design.

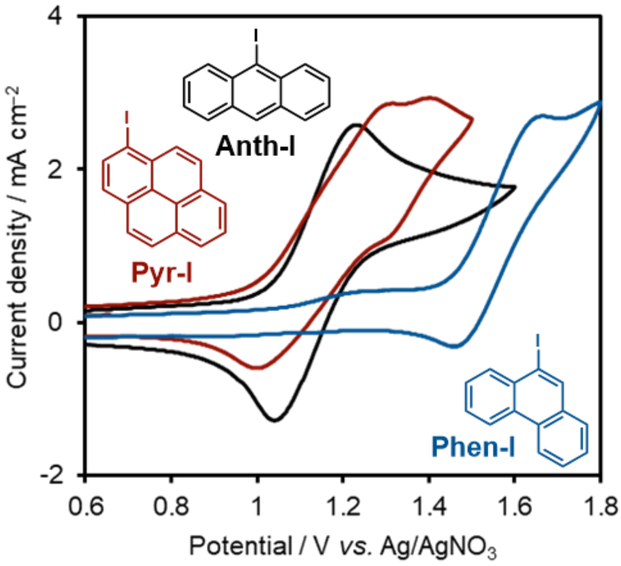

Figure S4. Cyclic voltammograms of 5 mM **Anth-I** (black line) and 5 mM **Phen-I** (blue line) and 5 mM **Pyr-I** (red line) in 0.1 M $Bu_4NB(C_6F_5)_4/CH_2Cl_2$ at 0.1 V s^{-1}.

*3-1-2. Cyclic voltammetry of 9-iodoanthracene (**Anth-I**)*

CV measurement of 9-iodoanthracene (**Anth-I**) with trifluoromethanesulfonate (TfO^-) as the supporting electrolyte, an irreversible voltammogram was observed (Figure S2). This result indicates that the radical cations state of **Anth-I** is highly unstable thus the interaction with TfO^- anion induces a further irreversible chemical process.

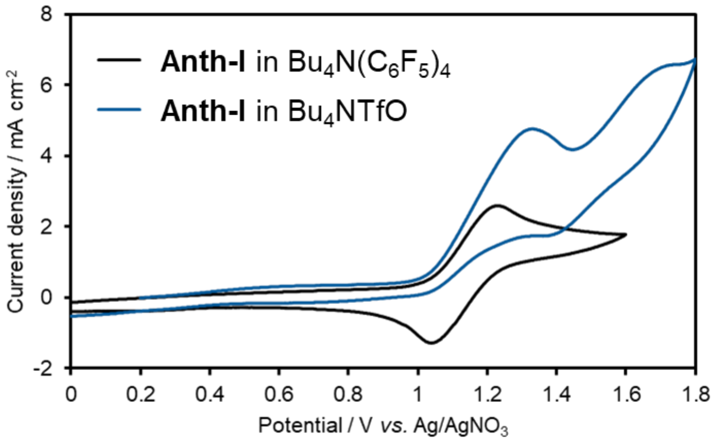

Figure S5. Cyclic voltammograms of 5 mM 9-iodoanthracene (**Anth-I**) in 0.1 M Bu_4NX/CH_2Cl_2. [X = $B(C_6F_5)_4$ (black), TfO^- (blue line)] at a scan rate of 0.1 V/s.

*3-1-3. Cyclic voltammetry of 9-iodo10-mesitylanthracene (**1a**) and iodobenzene and 4-iodoanisole*

1a showed the only reversible voltammogram among the three substrates, and its oxidation potential was confirmed to be lower than that of monocyclic iodoarene. This suggests that the radical cation state in **1a** does not only suppress side reaction but also realizes wide range of substrates due to its sufficiently low oxidation potential.

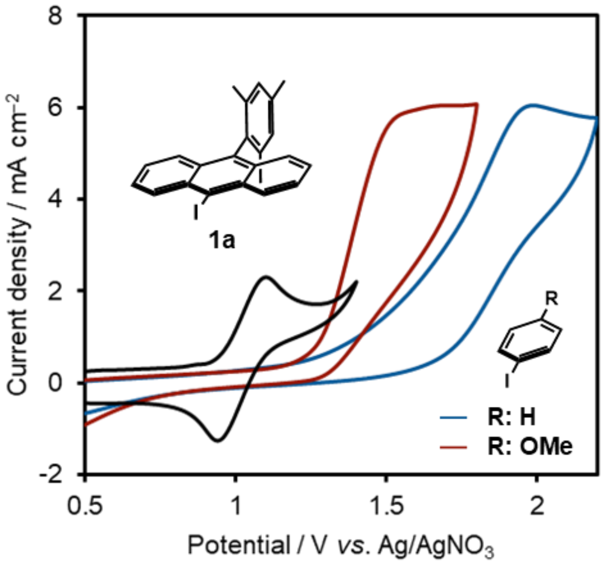

Figure S6. Cyclic voltammograms of 5 mM **1a** (black line) and 5 mM monocyclic iodoarenes (blue and red lines) in 0.1 M $LiTfO/CH_2Cl_2$ at 0.1 V s^{-1}.

*3-1-4. Cyclic voltammetry of **1a** with base*

CV measurements of **1a** recorded in 0.1 M LiTfO/MeCN+CH_2Cl_2 (6:4 in vol.) showed a fully reversible oxidation response. When bases such as pyridine and 2,6-lutidine were added to the electrolytic solution, the voltammograms changed to irreversible with an increase of the oxidation current (Figures S4, 5). This result suggests that the pyridine and 2,6-lutidine interacted with the **1a**$^{\bullet+}$ to induce further chemical processes. On the other hand, when 2,6-di-*tert*-butylpyridine was used as a base, no change was observed in the voltammogram (Figure S6).

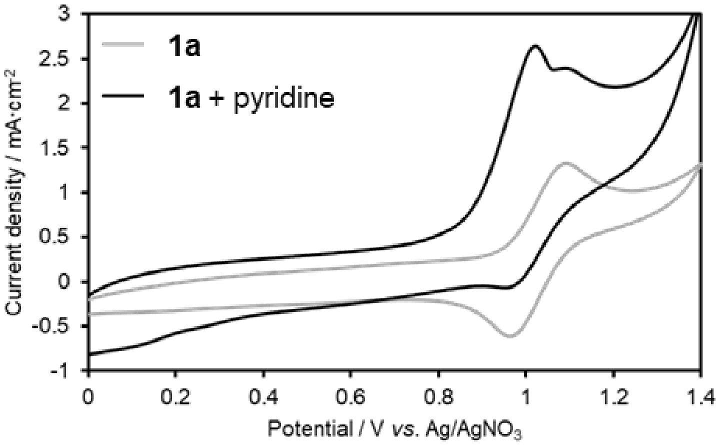

Figure S7. Cyclic voltammograms of 2 mM **1a** (gray line) and 2 mM **1a** with 2 mM pyridine (black line) in 0.1 M LiTfO/MeCN+CH_2Cl_2 (6:4 in vol.) at a scan rate of 0.1 V/s.

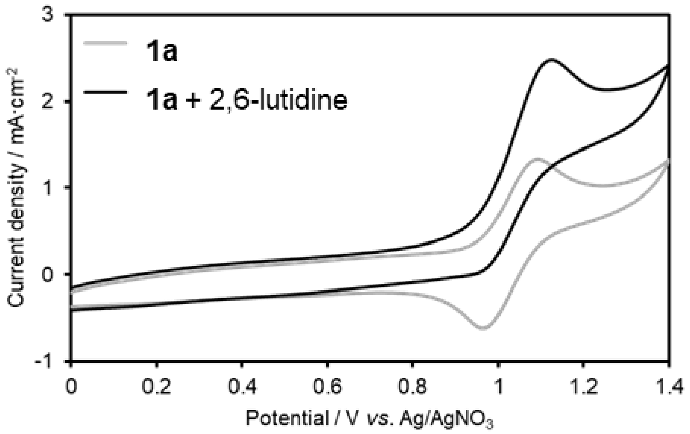

Figure S8. Cyclic voltammograms of 2 mM **1a** (gray line) and 2 mM **1a** with 2 mM 2,6-lutidine (black line) in 0.1 M LiTfO/MeCN+CH_2Cl_2 (6:4 in vol.) at a scan rate of 0.1 V/s.

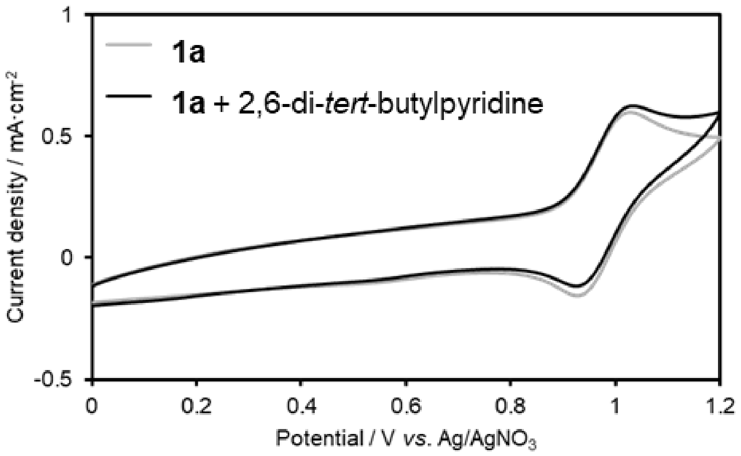

Figure S9. Cyclic voltammograms of 1 mM **1a** (gray line) and 2 mM **1a** with 1 mM 2,6-di-*tert*-butylpyridine (black line) in 0.1 M LiTfO/MeCN+CH_2Cl_2 (6:4 in vol.) at a scan rate of 0.1 V/s.

*3-1-5. Cyclic voltammetry of N-protected aminobiphenyl derivatives (**2a-2d**)*

CV measurements of *N*-protected aminobiphenyl derivatives (**2a-d**) showed irreversible waves (Figure S6). The order of the oxidation potential is **2d** > **2a** > **2b** > **2c**.

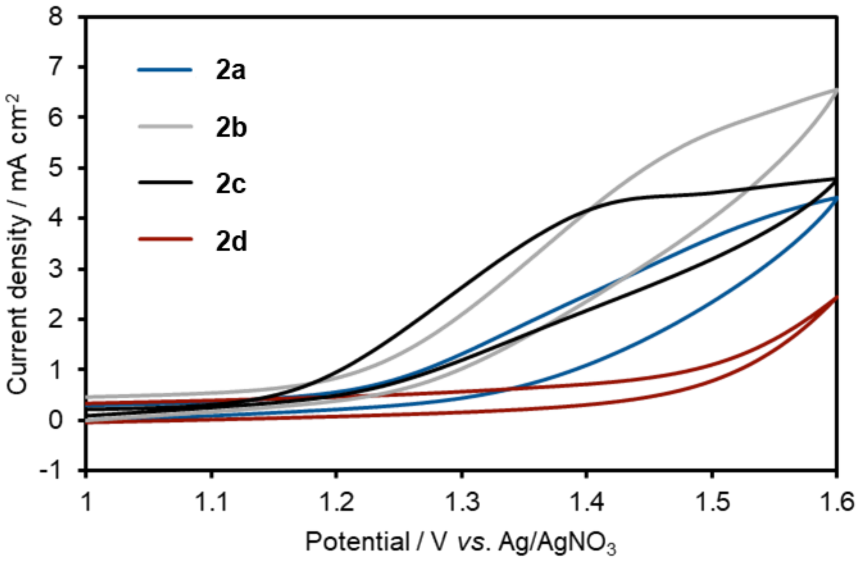

Figure S10. Cyclic voltammograms of *N*-protected aminobiphenyl derivatives 5 mM **2a-d** in 0.1 M LiTfO/MeCN+CH_2Cl_2 (6:4 in vol.) at a scan rate of 0.1 V/s. Protecting groups of amine: acetyl (**2a**), Moc(**2b**), Boc (**2c**), Ts (**2d**).

*3-1-6. Cyclic voltammetry of **1a** with **2a***

The addition of **2a** to the electrolytic solution containing **1a** resulted in an increased oxidation current and a disappearance of reduction current compared to the case without **2a**. The oxidation current even increased in the presence of 2,6-di-*tert*-butylpyridine (^{t}Bu$_2$Py).

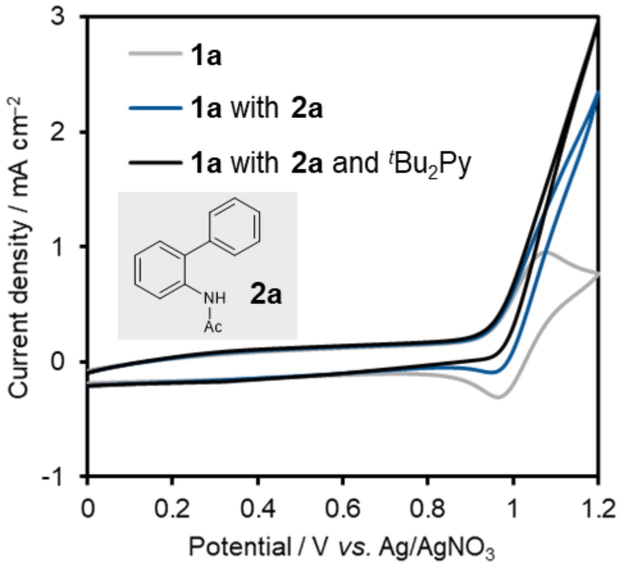

Figure S11. Cyclic voltammograms of 1 mM **1a** (gray line), 1 mM **1a** with 20 mM **2a** (blue line), and 1 mM **1a** with 20 mM **2a** and 4 mM ^{t}Bu$_2$Py (black line) in 0.1 M LiTfO / MeCN+CH_2Cl_2 (6/4 in vol.) solution at a scan rate of 0.1 V s^{-1}.

*3-1-7. Cyclic voltammetry of **1a** and **2c** or deuterated **2c** derivatives (**2c-d$_1$** and **2c-d$_5$**)*

Deuterated **2c** derivatives (**2c-d$_1$** and **2c-d$_5$**) were used instead of **2c** in CV measurement to clarify whether deprotonation steps in overall reaction are rate-determining step or not. There is no significant change in the oxidation current in the voltammograms, indicating that each deprotonation step is not a rete-determining step.

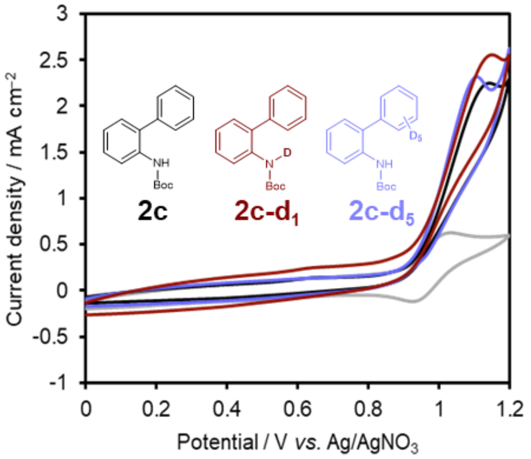

Figure S12. Cyclic voltammograms of 1 mM **1a** (gray line) and 1 mM **1a** with 20 mM **2c** (black line) or **2c-d₁** (reddish brown line) or **2c-d₅** (purple line) with 4 mM ^{t}Bu$_2$Py in 0.1 M LiTfO / MeCN+CH_2Cl_2 (6/4 in vol.) solution at a scan rate of 0.1 V s^{-1}.

*3-1-8. Cyclic voltammetry of 9-halo-10-arylanthrancene (**1a-1h**) and N-protected aminobiphenyl derivatives for FOWA*

CV measurement of 9-halo-10-arylanthracene (**1a-1h**) with *N*-protected aminobiphenyl derivatives (**2a-2d**) for Foot-of-the-wave analysis. The background current value is subtracted from all the current values.

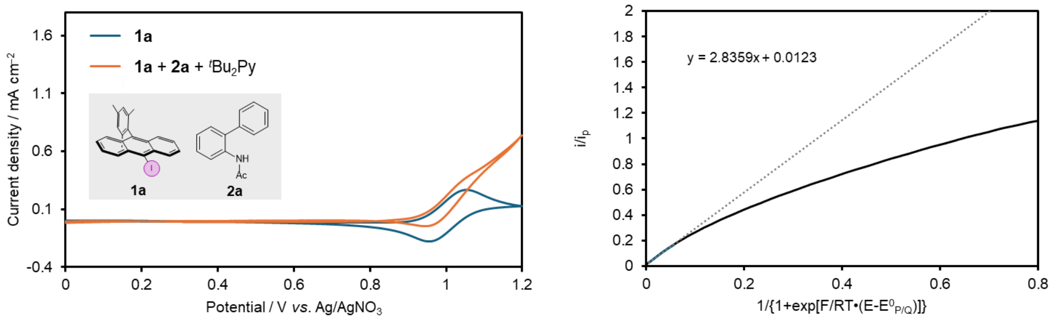

Figure S13. Cyclic voltammograms of 1 mM **1a** (blue line) and 1 mM **1a** with 5 mM **2a** and 4 mM ^{t}Bu$_2$Py (orange line) in 0.1 M LiTfO / MeCN+CH_2Cl_2 (6/4 in vol.) solution at a scan rate of 0.1 V s^{-1} (left). The catalytic current in the left figure (orange line) was converted to the right figure (black line) using a mathematical analysis method used for FOWA.

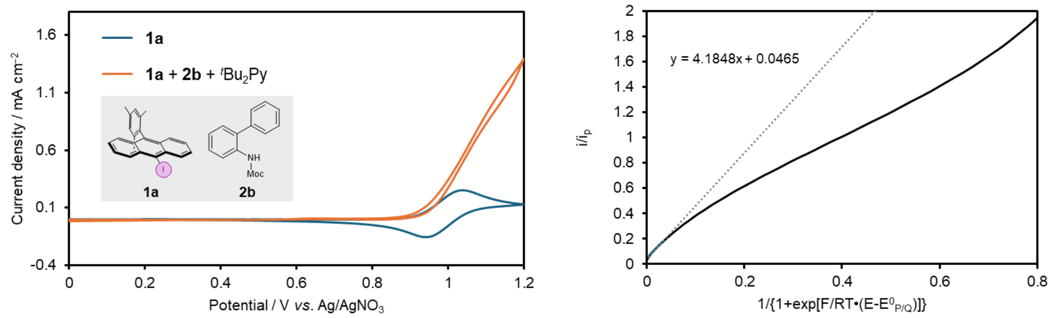

Figure S14. Cyclic voltammograms of 1 mM **1a** (blue line) and 1 mM **1a** with 5 mM **2b** and 4 mM ^{t}Bu$_2$Py (orange line) in 0.1 M LiTfO / MeCN+CH$_2$Cl$_2$ (6/4 in vol.) solution at a scan rate of 0.1 V s^{-1} (left). The catalytic current in the left figure (orange line) was converted to the right figure (black line) using a mathematical analysis method used for FOWA.

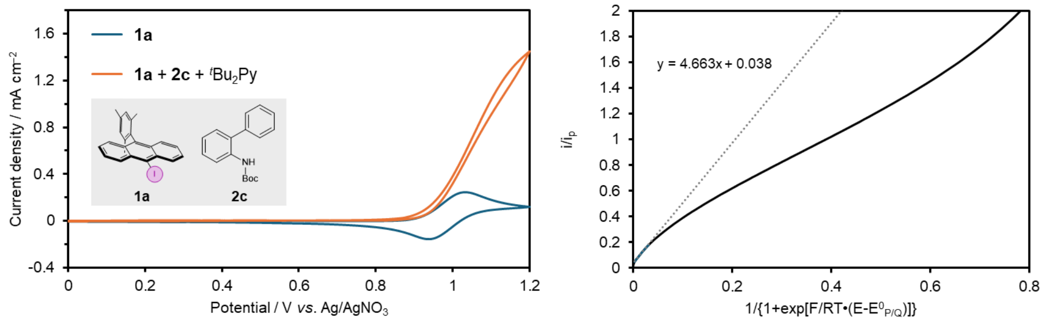

Figure S15. Cyclic voltammograms of 1 mM **1a** (blue line) and 1 mM **1a** with 5 mM **2c** and 4 mM ^{t}Bu$_2$Py (orange line) in 0.1 M LiTfO / MeCN+CH$_2$Cl$_2$ (6/4 in vol.) solution at a scan rate of 0.1 V s^{-1} (left). The catalytic current in the left figure (orange line) was converted to the right figure (black line) using a mathematical analysis method used for FOWA.

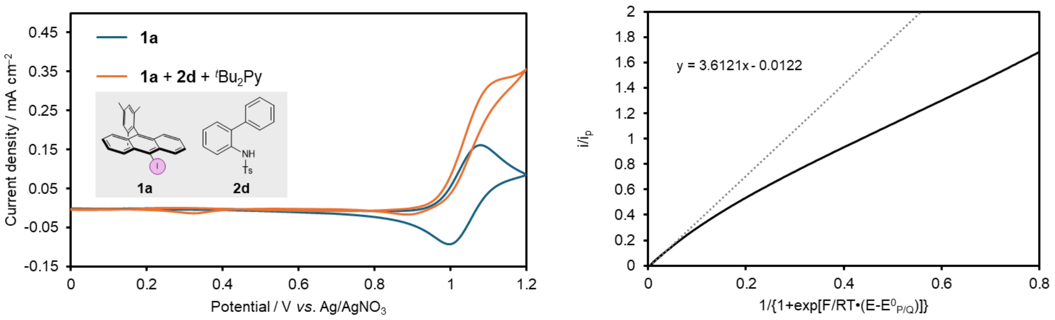

Figure S16. Cyclic voltammograms of 1 mM **1a** (blue line) and 1 mM **1a** with 5 mM **2d** and 4 mM ^{t}Bu$_2$Py (orange line) in 0.1 M LiTfO / MeCN+CH$_2$Cl$_2$ (6/4 in vol.) solution at a scan rate of 0.1 V s^{-1} (left). The catalytic current in the left figure (orange line) was

converted to the right figure (black line) using a mathematical analysis method used for FOWA.

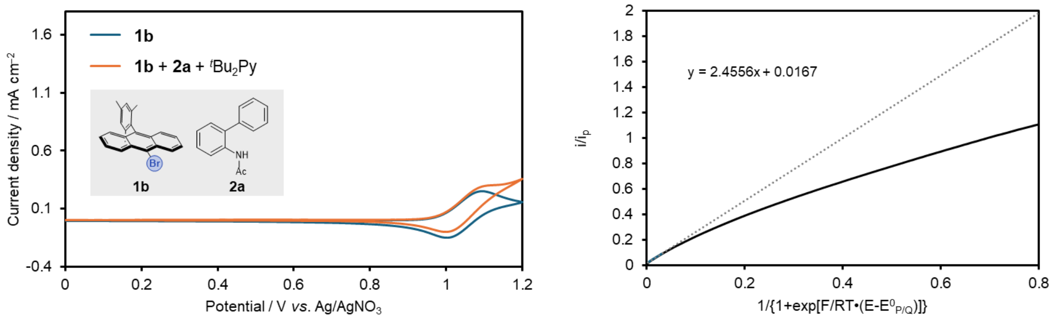

Figure S17. Cyclic voltammograms of 1 mM **1b** (blue line) and 1 mM **1b** with 5 mM **2a** and 4 mM ^{t}Bu$_2$Py (orange line) in 0.1 M LiTfO / MeCN+CH_2Cl_2 (6/4 in vol.) solution at a scan rate of 0.1 V s^{-1} (left). The catalytic current in the left figure (orange line) was converted to the right figure (black line) using a mathematical analysis method used for FOWA.

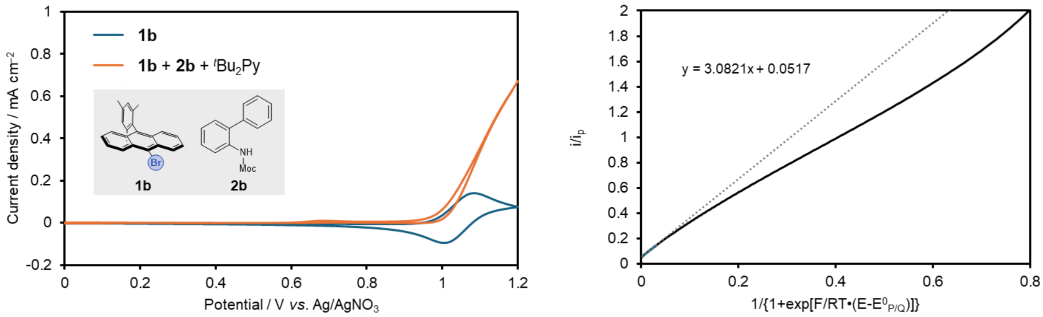

Figure S18. Cyclic voltammograms of 1 mM **1b** (blue line) and 1 mM **1b** with 5 mM **2b** and 4 mM ^{t}Bu$_2$Py (orange line) in 0.1 M LiTfO / MeCN+CH_2Cl_2 (6/4 in vol.) solution at a scan rate of 0.1 V s^{-1} (left). The catalytic current in the left figure (orange line) was converted to the right figure (black line) using a mathematical analysis method used for FOWA.

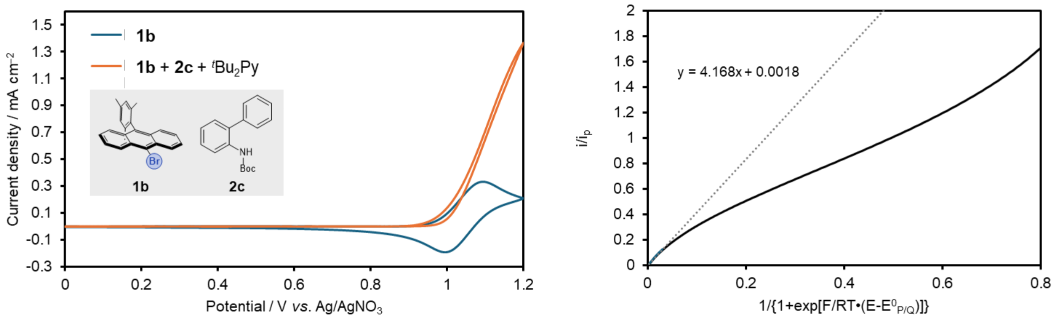

Figure S19. Cyclic voltammograms of 1 mM **1b** (blue line) and 1 mM **1b** with 5 mM **2c** and 4 mM ^{t}Bu$_2$Py (orange line) in 0.1 M LiTfO / MeCN+CH_2Cl_2 (6/4 in vol.) solution at a scan rate of 0.1 V s^{-1} (left). The catalytic current in the left figure (orange line) was converted to the right figure (black line) using a mathematical analysis method used for FOWA.

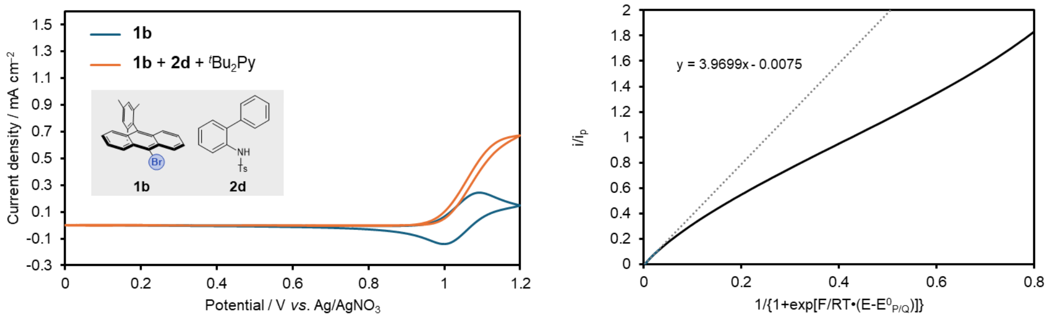

Figure S20. Cyclic voltammograms of 1 mM **1b** (blue line) and 1 mM **1b** with 5 mM **2d** and 4 mM ^{t}Bu$_2$Py (orange line) in 0.1 M LiTfO / MeCN+CH_2Cl_2 (6/4 in vol.) solution at a scan rate of 0.1 V s^{-1} (left). The catalytic current in the left figure (orange line) was converted to the right figure (black line) using a mathematical analysis method used for FOWA.

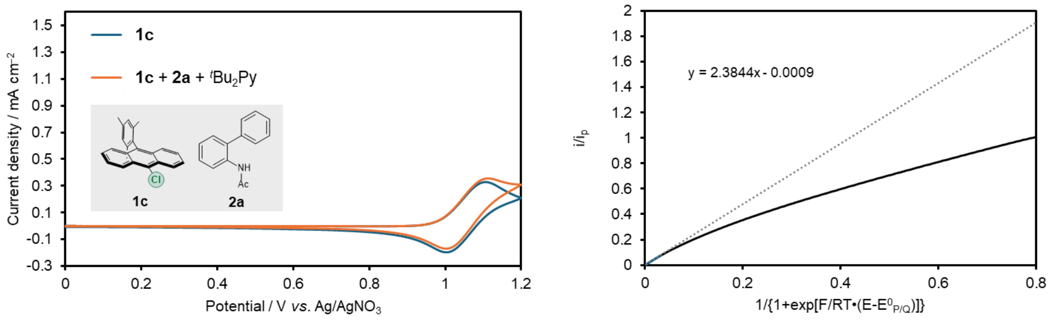

Figure S21. Cyclic voltammograms of 1 mM **1c** (blue line) and 1 mM **1c** with 5 mM **2a** and 4 mM ^{t}Bu$_2$Py (orange line) in 0.1 M LiTfO / MeCN+CH_2Cl_2 (6/4 in vol.) solution at a scan rate of 0.1 V s^{-1} (left). The catalytic current in the left figure (orange line) was converted to the right figure (black line) using a mathematical analysis method used for FOWA.

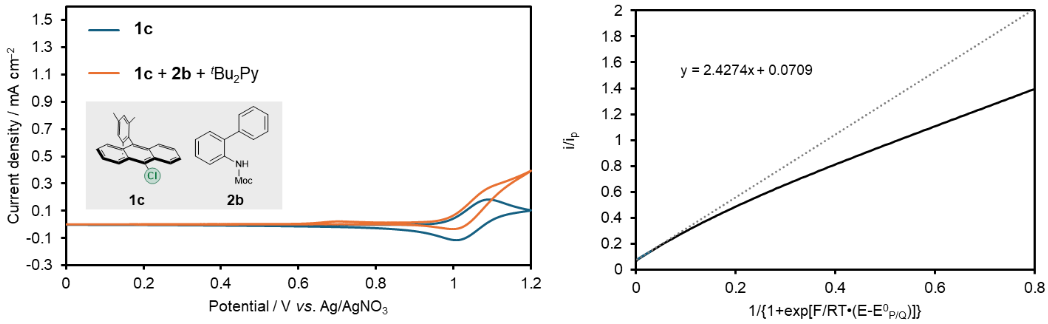

Figure S22. Cyclic voltammograms of 1 mM **1c** (blue line) and 1 mM **1c** with 5 mM **2b** and 4 mM ^{t}Bu$_2$Py (orange line) in 0.1 M LiTfO / MeCN+CH_2Cl_2 (6/4 in vol.) solution at a scan rate of 0.1 V s^{-1} (left). The catalytic current in the left figure (orange line) was converted to the right figure (black line) using a mathematical analysis method used for FOWA.

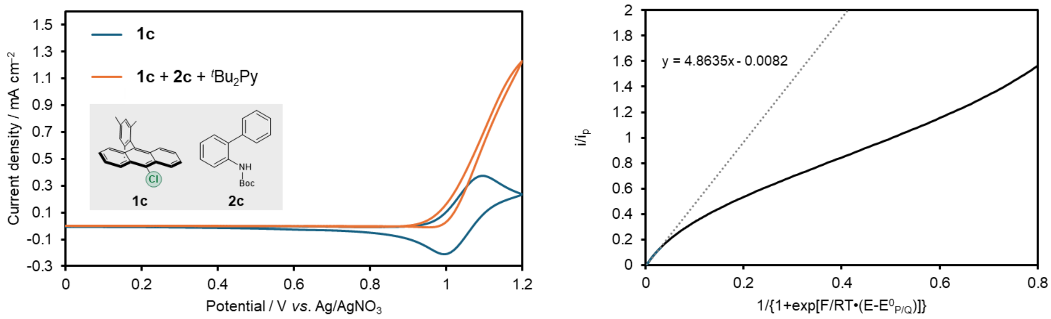

Figure S23. Cyclic voltammograms of 1 mM **1c** (blue line) and 1 mM **1c** with 5 mM **2c** and 4 mM ^{t}Bu$_2$Py (orange line) in 0.1 M LiTfO / MeCN+CH_2Cl_2 (6/4 in vol.) solution at a scan rate of 0.1 V s^{-1} (left). The catalytic current in the left figure (orange line) was converted to the right figure (black line) using a mathematical analysis method used for FOWA.

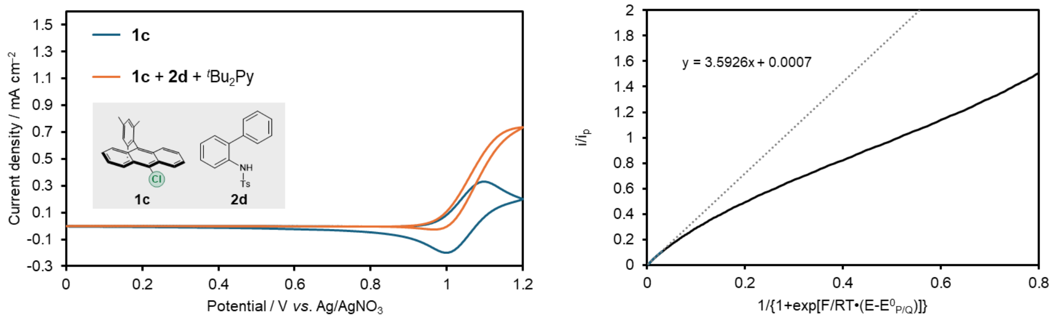

Figure S24. Cyclic voltammograms of 1 mM **1c** (blue line) and 1 mM **1c** with 5 mM **2d** and 4 mM ^{t}Bu$_2$Py (orange line) in 0.1 M LiTfO / MeCN+CH_2Cl_2 (6/4 in vol.) solution at a scan rate of 0.1 V s^{-1} (left). The catalytic current in the left figure (orange line) was

converted to the right figure (black line) using a mathematical analysis method used for FOWA.

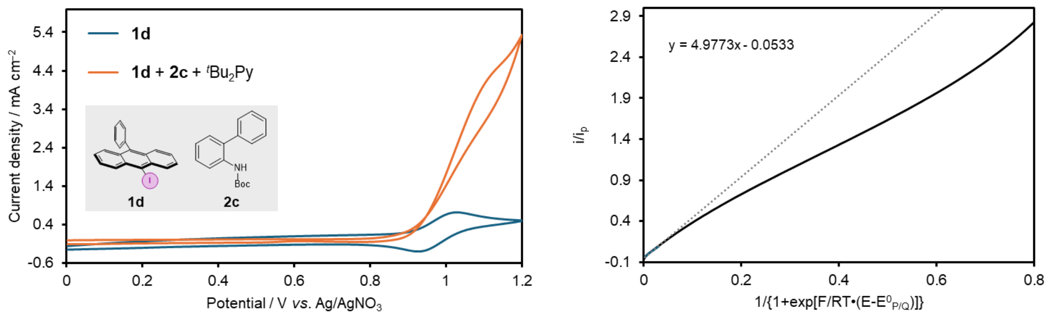

Figure S25. Cyclic voltammograms of 1 mM **1d** (blue line) and 1 mM **1d** with 20 mM **2c** and 20 mM $^{t}Bu_2Py$ (orange line) in 0.1 M LiTfO / $MeCN+CH_2Cl_2$ (6/4 in vol.) solution at a scan rate of 0.1 V s^{-1} (left). The catalytic current in the left figure (orange line) was converted to the right figure (black line) using a mathematical analysis method used for FOWA.

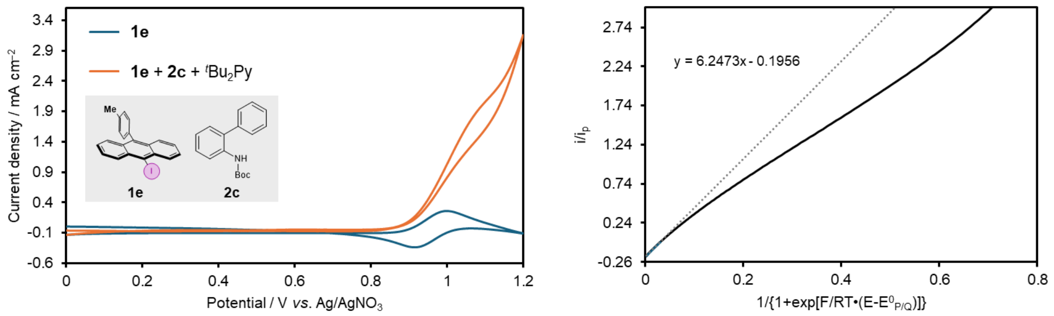

Figure S26. Cyclic voltammograms of 1 mM **1e** (blue line) and 1 mM **1e** with 20 mM **2c** and 20 mM $^{t}Bu_2Py$ (orange line) in 0.1 M LiTfO / $MeCN+CH_2Cl_2$ (6/4 in vol.) solution at a scan rate of 0.1 V s^{-1} (left). The catalytic current in the left figure (orange line) was converted to the right figure (black line) using a mathematical analysis method used for FOWA.

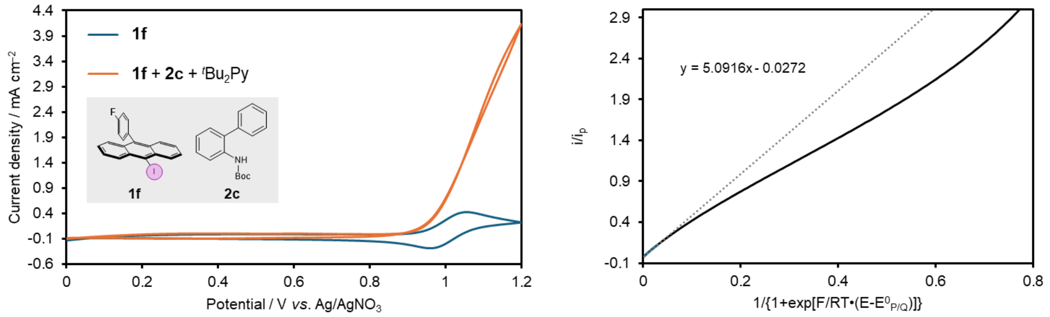

Figure S27. Cyclic voltammograms of 1 mM **1f** (blue line) and 1 mM **1f** with 20 mM **2c** and 20 mM ${}^{t}Bu_2Py$ (orange line) in 0.1 M LiTfO / $MeCN+CH_2Cl_2$ (6/4 in vol.) solution at a scan rate of 0.1 V s^{-1} (left). The catalytic current in the left figure (orange line) was converted to the right figure (black line) using a mathematical analysis method used for FOWA.

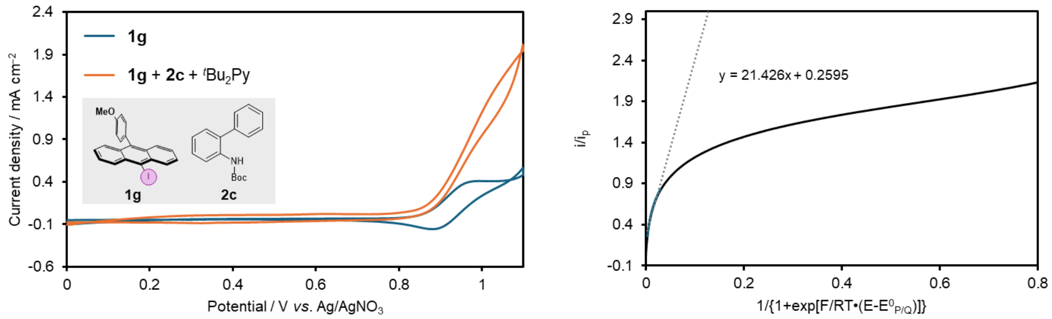

Figure S28. Cyclic voltammograms of 1 mM **1g** (blue line) and 1 mM **1g** with 20 mM **2c** and 20 mM ${}^{t}Bu_2Py$ (orange line) in 0.1 M LiTfO / $MeCN+CH_2Cl_2$ (6/4 in vol.) solution at a scan rate of 0.1 V s^{-1} (left). The catalytic current in the left figure (orange line) was converted to the right figure (black line) using a mathematical analysis method used for FOWA.

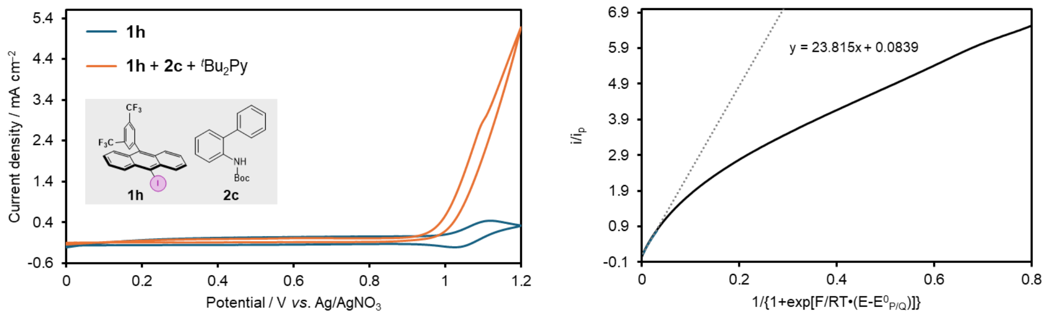

Figure S29. Cyclic voltammograms of 1 mM **1h** (blue line) and 1 mM **1h** with 20 mM **2c** and 20 mM ${}^{t}Bu_2Py$ (orange line) in 0.1 M LiTfO / $MeCN+CH_2Cl_2$ (6/4 in vol.) solution at a scan rate of 0.1 V s^{-1} (left). The catalytic current in the left figure (orange line) was converted to the right figure (black line) using a mathematical analysis method used for FOWA.

3-2. Electron paramagnetic spectroscopy (EPR) measurement

EPR spectra was recorded at room temperature on EPR spectrometer operated at 9453.909 MHz. Typical spectrometer parameters are shown as follows, scan range: 15 mT; center field set: 337.510 mT; time constant: 1.00 s; sweep time: 1 min; modulation

frequency: 100.00 kHz; microwave power: 1.00 mW. **1a**$^{\cdot+}$ was generated in bulk scale based on the electrosynthetic approach using a divided cell. Bulk electrolysis was conducted in the divided cell equipped with a Pt plate anode (2 cm × 2 cm), a Pt plate cathode (2 cm× 2 cm), and an Ag/$AgNO_3$ reference electrode. After placing a magnetic stirrer bar, **1a** (0.05 mmol in the anodic chamber) and Bu_4NTfO supporting electrolyte (1 mmol for each chamber), CH_2Cl_2 (10 mL for each chamber) was added as a solvent. 1 F/mol of charge was passed under constant potential application (1.2 V) at room temperature, givint deep blue color solution in anodic chamber derived from **1a**$^{\cdot+}$. After the electrolysis, the reaction mixture in the anodic chamber was transferred into a tube, and EPR measurement was performed at room temperature (g = 2.033, Figure S30).

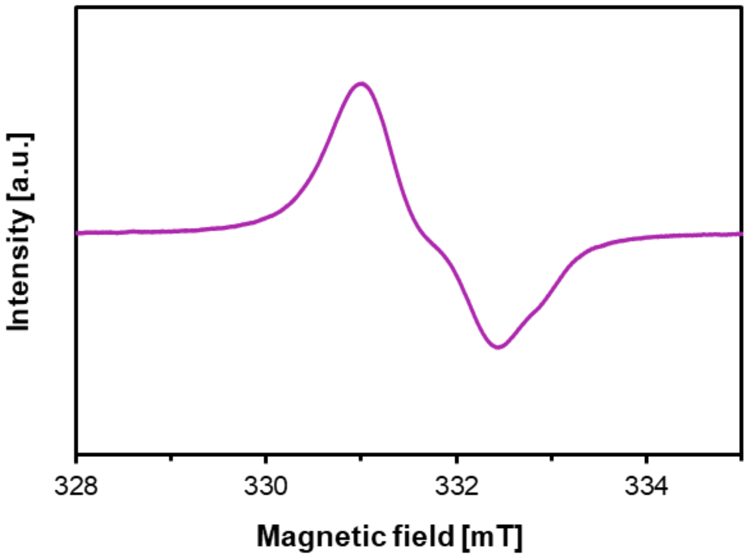

Figure S30. EPR spectrum of radical cation state of 9-iodo-10-mesitylanthracene (**1a**$^{\cdot+}$).

EPR spectra was recorded at room temperature on EPR spectrometer operated at 9454.151 MHz. Typical spectrometer parameters are shown as follows, scan range: 5 mT; center field set: 331.723 mT; time constant: 1.00 s; sweep time: 1 min; modulation frequency: 100.00 kHz; microwave power: 4.00 mW. **1b**$^{\cdot+}$ was generated in bulk scale based on the electrosynthetic approach using a divided cell. Bulk electrolysis was conducted in the divided cell equipped with a Pt plate anode (2 cm × 2 cm), a Pt plate cathode (2 cm× 2 cm), and an Ag/$AgNO_3$ reference electrode. After placing a magnetic stirrer bar, **1b** (0.05 mmol in the anodic chamber) and Bu_4NTfO supporting electrolyte (1 mmol for each chamber), CH_2Cl_2 (10 mL for each chamber) was added as a solvent. 0.67 F/mol of charge was passed under constant potential application (1.2 V) at −78 °C, givint deep green color solution in anodic chamber derived from **1b**$^{\cdot+}$. After the electrolysis, the

reaction mixture in the anodic chamber was transferred into a tube, and EPR measurement was performed at room temperature (g = 2.001, Figure S31).

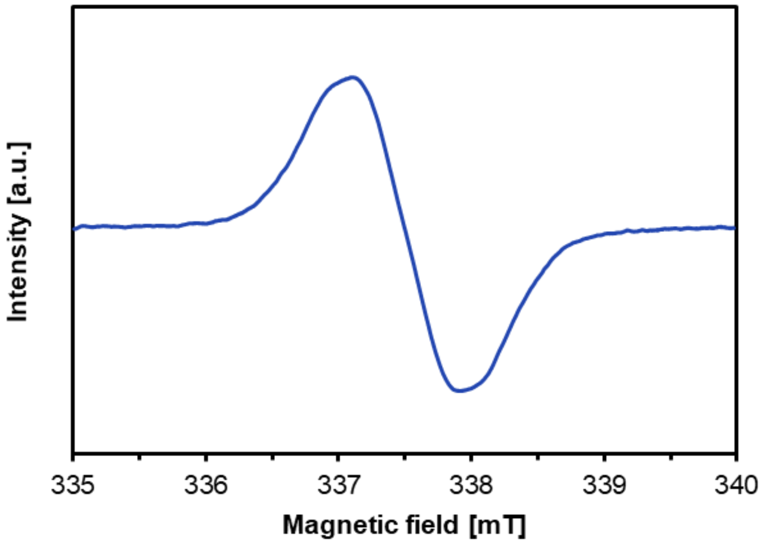

Figure S31. EPR spectrum of radical cation state of 9-bromo-10-mesitylanthracene (**1b**$^{\cdot+}$).

EPR spectra was recorded at room temperature on EPR spectrometer operated at 9454.949 MHz. Typical spectrometer parameters are shown as follows, scan range: 5 mT; center field set: 337.523 mT; time constant: 1.00 s; sweep time: 1 min; modulation frequency: 100.00 kHz; microwave power: 4.00 mW. **1c**$^{\cdot+}$ was generated in bulk scale based on the electrosynthetic approach using a divided cell. Bulk electrolysis was conducted in the divided cell equipped with a Pt plate anode (2 cm × 2 cm), a Pt plate cathode (2 cm× 2 cm), and an Ag/$AgNO_3$ reference electrode. After placing a magnetic stirrer bar, **1c** (0.05 mmol in the anodic chamber) and Bu_4NTfO supporting electrolyte (1 mmol for each chamber), CH_2Cl_2 (10 mL for each chamber) was added as a solvent. 0.62 F/mol of charge was passed under constant potential application (1.2 V) at −78 °C, givint deep green color solution in anodic chamber derived from **1c**$^{\cdot+}$. After the electrolysis, the reaction mixture in the anodic chamber was transferred into a tube, and EPR measurement was performed at room temperature (g = 2.001, Figure S32).

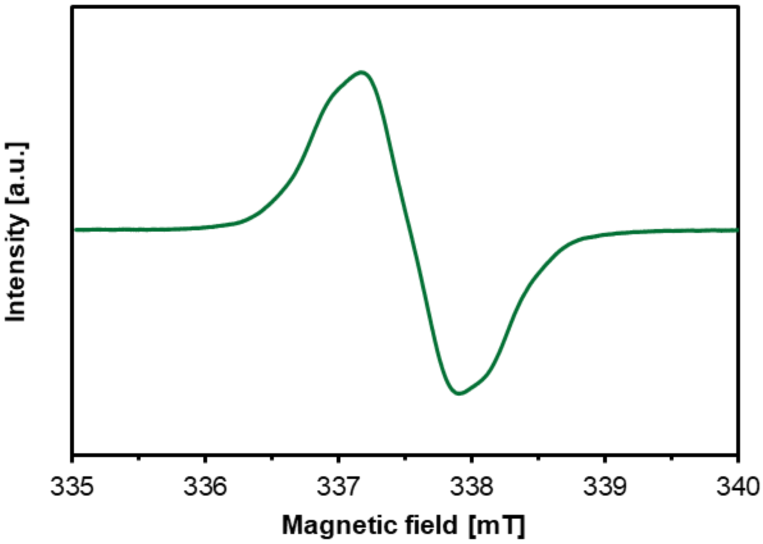

Figure S32. EPR spectrum of radical cation state of 9-chloro-10-mesitylanthracene (**1c**$^{\cdot+}$).

3-3. Computational analysis

3-3-1. Computational details

All geometries were optimized at CAM-B3LYP level of theory in gas phase. The optimized structures were confirmed to have no imaginary frequency mode in the harmonic approximation. With the optimized geometries, the DLPNO-CCSD(T) calculations were performed. The total free energy ΔG was computed with the following equation.

$$\Delta G = E_{\mathrm{cc}} + G_{\mathrm{corr}} + \Delta\mu$$

where E_{cc} is the DLPNO-CCSD(T) energy obtained in gas phase, G_{corr} is the thermal correction to Gibbs energy, and $\Delta\mu$ is the solvation free energy in acetonitrile. The G_{corr} was computed using frequency calculations at 323.15 K. The basis sets for the DFT and DLPNO-CCSD(T) calculations are def2-SVP and ma-def2-SVP, respectively.

In the calculations with solvation effect, dielectric continuum models, such as PCM, are widely employed. By employing the dielectric continuum model, we can evaluate the solvation effect for large molecules. However, the parameters employed in the model were optimized at room temperature and it is unclear whether the dielectric continuum model calculation with the default parameters can be applied to our system (323.15 K). Therefore, in this study, we employed RISM-SCF-cSED, which is one of the hybrid methods between quantum mechanics and statistical mechanics, and performed the calculations at 323.15 K. Because of the system size, we employed the

RISM-SCF-cSED for single point calculation. $\Delta\mu$ was computed with the following equation.

$$\Delta\mu = E_{\mathrm{DFT/RISM}} - E_{\mathrm{DFT}}$$

where $E_{\mathrm{DFT/RISM}}$ and E_{DFT} are the CAM-B3LYP energies in acetonitrile and gas phases, respectively. The RISM-SCF-cSED calculations were performed using the acetonitrile solvent data at 323.15 K with Kovalenko-Hirata closure.

The DLPNO-CCSD(T) calculations were performed with ORCA 6.0 program and other calculations were performed with GAMESS program.

3-3-2. Electrostatic potential mapping

Electrostatic potential (ESP) mapping of radical cation of (a) 9-iodo-10-mesitylanthracene (**1a**$^{\cdot+}$) and (b) 9-bromo-10-mesitylanthracene (**1b**$^{\cdot+}$) and (c) 9-chloro-10-mesitylanthracene (**1c**$^{\cdot+}$) are shown in Figure S31. In each of the radical cation **1a**$^{\cdot+}$-**1c**$^{\cdot+}$, the electron density of halogen atoms is anisotropically distributed. We can see the electron-deficient region (σ–hole), which is located along the extension line of the covalent bond.

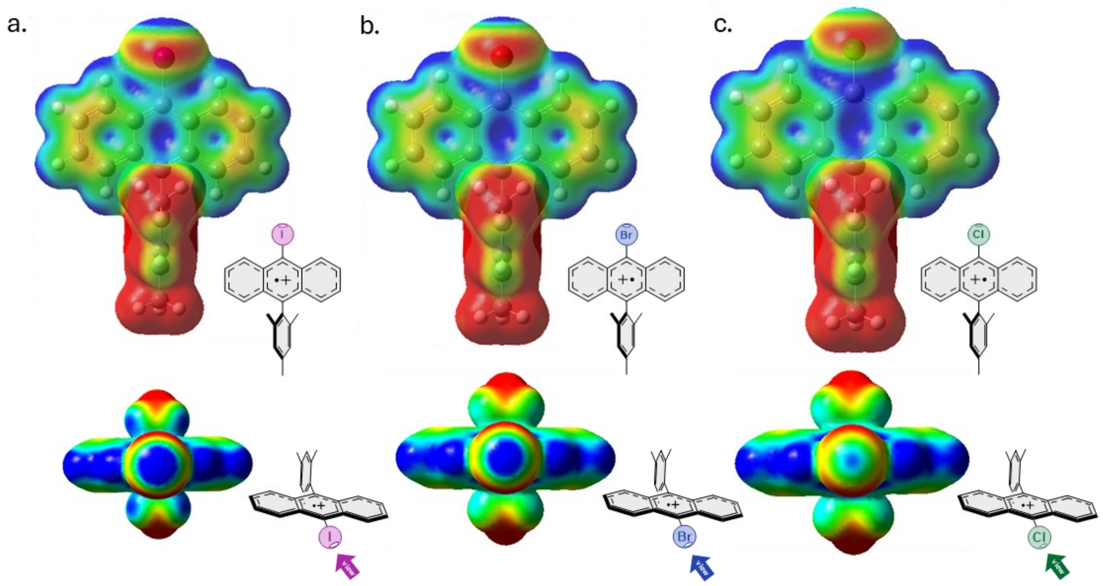

Figure S31. Electrostatic potential (ESP) mapping of radical cation of (a) 9-iodo-10-mesitylanthracene (**1a**$^{\cdot+}$), (b) 9-bromo-10-mesitylanthracene (**1b**$^{\cdot+}$), and (c) 9-chloro-10-mesitylanthracene (**1c**$^{\cdot+}$). Computed electrostatic potentials on 0.004 au molecular surfaces and the color scale corresponds to 69.0 kcal/mol to 94.1 kcal/mol (red to blue).

3-3-3. spin density mapping

Spin densities of **1h…2c**$^{\cdot}$ interacted with **^{t}Bu$_2$Py-H**$^{+}$ and **1h…2c**$^{\cdot}$ are computed with natural population analysis using CAM-B3LYP/def2-SVP level of theory (Figure S32). The spin density is localized on haloanthracene in the case of complex form between **1h…2c**$^{\cdot}$ and **^{t}Bu$_2$Py-H**$^{+}$, while the radical electron is localized on N site when the **^{t}Bu$_2$Py-H**$^{+}$ is released from **1h…2c**$^{\cdot}$. The spin density change shows that the electron transfer occurs from N site to the haloanthracene in the deprotonation process.

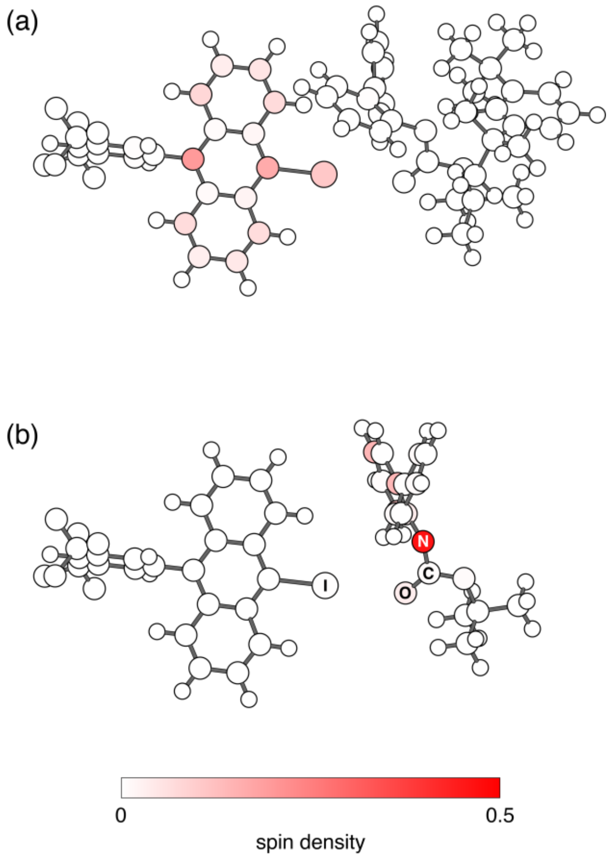

Figure S32. Spin densities of (a) **1h…2c**$^{\cdot}$ interacted with **^{t}Bu$_2$Py-H**$^{+}$ and (b) **1h…2c**$^{\cdot}$.

3-3-4. Comparison of halogen bonding interaction and π-π stacking

The optimized structures of **1h**$^{\cdot+}$ and **2c** coordinated with halogen bonding interaction or π-π interaction in the presence of TfO^{-} are shown in Figure S33. The π-π stacked adduct is energetically less favorable than the corresponding halogen-bonded structure. Furthermore, the ability to adopt a halogen bonding interaction state is key to promoting the PCET step that constitutes the crucial stage of this reaction. This interpretation is consistent with previous reports in the literature, where electron delocalization arising from orbital mixing between halogenated electrophiles and nucleophiles has been shown to reduce the barrier for electron transfer.[19]

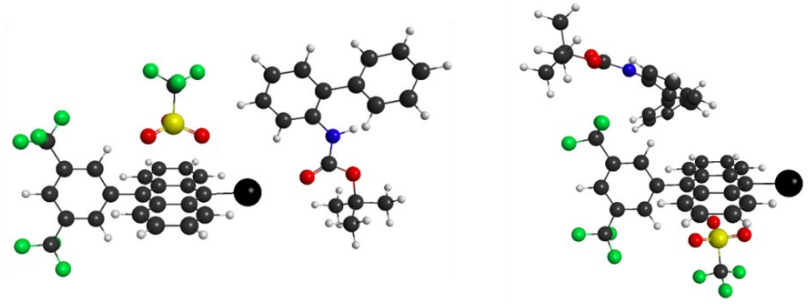

[**1h**$^{\bullet+}$…**2c**]$_X$TfO$^-$ [**1h**$^{\bullet+}$…**2c**]$_\pi$TfO$^-$

ΔE (kcal/mol) 0.00 +2.18

Figure S33. Optimized structure of **1h$^{\bullet+}$** and **2c** and TfO$^-$ coordinated with halogen bonding interaction (left) or stacked with π-π interaction (right), and the energy difference of each structure.

3-3-5. Cartesian coordinates of the optimized structures

1a$^{\bullet+}$

C	-1.126313	-3.680610	-0.000294
C	-1.842154	-2.497237	-0.000521
C	-1.193310	-1.241428	0.000159
C	0.243355	-1.227400	0.001118
C	0.944703	-2.455646	0.001277
C	0.274202	-3.663688	0.000600
C	-1.895785	0.000039	-0.000071
C	0.959623	-0.000031	0.001849
C	0.243420	1.227367	0.001790
C	-1.193243	1.241467	0.000798
C	-1.842012	2.497315	0.000809
H	-2.930006	2.539202	0.000096
C	-1.126106	3.680648	0.001763
C	0.274411	3.663656	0.002793
C	0.944836	2.455577	0.002820
H	-1.661672	-4.631785	-0.000810
H	-2.930152	-2.539056	-0.001223
H	2.034692	-2.433072	0.002075
H	0.834480	-4.600196	0.000793
H	-1.661420	4.631850	0.001753
H	0.834734	4.600137	0.003628
H	2.034824	2.432925	0.003824
C	2.448946	-0.000072	0.002558
C	3.141971	-0.001412	1.225806
C	3.143801	0.001009	-1.223302
C	4.537778	-0.001609	1.198327
C	4.536351	0.000680	-1.195251
C	5.255182	-0.000470	0.002991
H	5.080243	-0.002807	2.146772
H	5.079642	0.001204	-2.143772
C	2.407849	0.002610	-2.538365
H	1.758502	-0.880147	-2.644289
H	1.764575	0.889731	-2.645230
H	3.109828	-0.000177	-3.381817
C	6.758569	0.000797	-0.008802
H	7.149491	-0.873989	-0.550176
H	7.147555	0.895459	-0.518448
H	7.172333	-0.016526	1.007943
C	2.408118	-0.002841	2.542040
H	1.760818	0.881202	2.649885
H	1.763218	-0.888730	2.649240
H	3.111459	-0.002221	3.384344
I	-3.986383	0.000088	-0.001697

1b$^{\bullet+}$

C	-1.555268	-3.675991	-0.001222
C	-2.266677	-2.489541	-0.001323
C	-1.604455	-1.241912	-0.000412
C	-0.169397	-1.228445	0.000689
C	0.525148	-2.459973	0.000680
C	-0.154267	-3.663936	-0.000240
C	-2.300215	0.000068	-0.000595
C	0.547520	-0.000086	0.001711
C	-0.169271	1.228353	0.001830
C	-1.604340	1.241977	0.000579
C	-2.266470	2.489726	0.000677
H	-3.355076	2.518933	-0.000270
C	-1.554924	3.676064	0.002024
C	-0.153913	3.663831	0.003414
C	0.525396	2.459824	0.003324
H	-2.093314	-4.625633	-0.001923
H	-3.355291	-2.518587	-0.002118
H	1.615335	-2.444594	0.001528
H	0.401019	-4.603460	-0.000174
H	-2.092851	4.625770	0.002059
H	0.401469	4.603303	0.004625
H	1.615579	2.444387	0.004540
C	2.036468	-0.000138	0.002587
C	2.729321	-0.002237	1.226004
C	2.731395	0.001720	-1.223303
C	4.125097	-0.002370	1.198615
C	4.123912	0.001388	-1.195054
C	4.842587	-0.000478	0.003307
H	4.667503	-0.004133	2.147089
H	4.667342	0.002469	-2.143491
C	1.995507	0.004286	-2.538413
H	1.345000	-0.877597	-2.644515
H	1.353550	0.892363	-2.645407
H	2.697496	0.000565	-3.381847
C	6.345947	0.000812	-0.008345
H	6.736873	-0.873662	-0.550213
H	6.734917	0.895760	-0.517493
H	6.759647	-0.017076	1.008408
C	1.995251	-0.004522	2.542128
H	1.347630	0.879247	2.650371
H	1.350771	-0.890773	2.648956
H	2.698425	-0.003980	3.384565
Br	-4.166918	0.000177	-0.002190

1c$^{•+}$			
C	-2.01450	-3.67103	0.00003
C	-2.72059	-2.48132	-0.00085
C	-2.04630	-1.24130	-0.00037
C	-0.61272	-1.22911	0.00100
C	0.07506	-2.46407	0.00198
C	-0.61292	-3.66388	0.00149
C	-2.73894	0.00006	-0.00111
C	0.10484	0.00004	0.00145
C	-0.61270	1.22919	0.00069
C	-2.04628	1.24140	-0.00059
C	-2.72055	2.48143	-0.00132
H	-3.80973	2.49805	-0.00225
C	-2.01445	3.67112	-0.00084
C	-0.61287	3.66396	0.00037
C	0.07510	2.46415	0.00112
H	-2.55546	-4.61900	-0.00036
H	-3.80976	-2.49794	-0.00189
H	1.16540	-2.45584	0.00330
H	-0.06255	-4.60635	0.00228
H	-2.55539	4.61911	-0.00141
H	-0.06250	4.60643	0.00076
H	1.16543	2.45589	0.00219
C	1.59348	0.00001	0.00260
C	2.28609	0.00053	1.22621
C	2.28873	-0.00083	-1.22314
C	3.68185	0.00022	1.19914
C	3.68122	-0.00110	-1.19458
C	4.39960	-0.00046	0.00397
H	4.22406	0.00039	2.14772
H	4.22489	-0.00192	-2.14288
C	1.55305	-0.00143	-2.53839
H	0.90651	-0.88625	-2.64455
H	0.90726	0.88382	-2.64566
H	2.25517	-0.00225	-3.38172
C	5.90295	0.00080	-0.00734
H	6.29397	-0.87470	-0.54746
H	6.29200	0.89477	-0.51813
H	6.31644	-0.01514	1.00953
C	1.55166	0.00097	2.54215
H	0.90507	0.88567	2.64901
H	0.90621	-0.88444	2.65015
H	2.25460	0.00196	3.38478
Cl	-4.44745	0.00009	-0.00267

1h

C	-1.0943282602	3.1803102464	-0.3746771621
C	-0.5264274368	1.9573280216	-0.1773829719
C	-1.3187581271	0.7686261373	-0.0566410630
C	-2.7502979508	0.9063508910	-0.1480373429
C	-3.3017904100	2.2137871778	-0.3576462707
C	-2.5065097889	3.3137927492	-0.4680507475
C	-0.7789895530	-0.5142619310	0.1444452195
C	-3.5770794235	-0.2243991977	-0.0330206150
C	-3.0196007903	-1.4985415241	0.1693953352
C	-1.5900251786	-1.6571344383	0.2610089182
C	-1.0789267792	-2.9805963369	0.4671612817
H	-0.0017059077	-3.1203965985	0.5382409728
C	-1.9044429104	-4.0592503064	0.5761430085
C	-3.3143245992	-3.9007323086	0.4864040083
C	-3.8480435854	-2.6633053325	0.2898962126
H	-0.4597908503	4.0644755265	-0.4620446300
H	0.5570069596	1.8764747456	-0.1077435895
H	-4.3840168339	2.3180210261	-0.4297958965
H	-2.9504730328	4.2983098731	-0.6276601774
H	-1.4800730912	-5.0528521166	0.7332086304
H	-3.9662984410	-4.7718918029	0.5752167138
H	-4.9289212502	-2.5439715297	0.2206562590
I	1.3316706235	-0.7310703447	0.2710294051
C	-5.0609611996	-0.0689666085	-0.1234288579
C	-5.8195026695	0.1807898537	1.0250075512
C	-5.7158329167	-0.1738790122	-1.3501940947
C	-7.2000178634	0.3207652595	0.9424309856
H	-5.3250351787	0.2638294389	1.9933964842
C	-7.1004129662	-0.0266333381	-1.4289996023
H	-5.1412380870	-0.3797567914	-2.2548441374
C	-7.8495564820	0.2193443426	-0.2858079653
H	-8.9332641033	0.3242666489	-0.3496055941
C	-7.7812490306	-0.0959595008	-2.7699474573
C	-8.0280286326	0.5565181283	2.1775857483
F	-7.7750138017	1.0932314506	-3.3828716939
F	-7.1721124227	-0.9612126828	-3.5855699688
F	-9.0575277924	-0.4730995547	-2.6543493065
F	-8.8241056527	1.6209598776	2.0314773357
F	-7.2717738556	0.7613920432	3.2577846596
F	-8.8209378151	-0.4898554074	2.4336760484

1h•+

C	-1.0050918275	3.1793465737	-0.4190076591
C	-0.4363998335	1.9300937061	-0.2494178712
C	-1.2285242017	0.7632721226	-0.1530815017
C	-2.6571687265	0.9100073146	-0.2164056073
C	-3.2034578371	2.2011197627	-0.4151072907
C	-2.3950479722	3.3173335153	-0.5114667534
C	-0.6717288874	-0.5421516570	-0.0148570610
C	-3.5030747535	-0.2265271151	-0.1054314997
C	-2.9368303029	-1.5219198402	0.0404229772
C	-1.5091447966	-1.6930378471	0.0710460879
C	-1.0007090816	-3.0052999196	0.2049196056
H	0.0760855290	-3.1662179937	0.2226654512
C	-1.8394721318	-4.0989178365	0.3191875711
C	-3.2288264472	-3.9281861614	0.3169617314
C	-3.7647117436	-2.6614985918	0.1851747826
H	-0.3616149665	4.0580322629	-0.4903765101
H	0.6481884356	1.8515347896	-0.1933722107
H	-4.2828485190	2.3135674893	-0.5034583358
H	-2.8408088553	4.3017708951	-0.6633913485
H	-1.4103929781	-5.0973513512	0.4207134784
H	-3.8887636409	-4.7907003728	0.4240177183
H	-4.8459277774	-2.5341269381	0.2001178788
I	1.4032004113	-0.7704070577	0.0615178515
C	-4.9794032146	-0.0546004238	-0.1310598802
C	-5.6307293468	0.5552324214	0.9483379277
C	-5.7297439432	-0.4993189625	-1.2206868119
C	-7.0109886978	0.7062118160	0.9337274036
H	-5.0626772985	0.9024875527	1.8122125989
C	-7.1119497303	-0.3225687103	-1.2351548620
H	-5.2414866063	-0.9813226554	-2.0695549801
C	-7.7590709397	0.2729109522	-0.1597833955
H	-8.8435698450	0.3942890628	-0.1723336463
C	-7.8954275392	-0.7593953721	-2.4511757166
C	-7.7261009957	1.3241002469	2.1128135717
F	-7.8822768849	0.1865511789	-3.3903374890
F	-7.3626567500	-1.8620791789	-2.9842284564
F	-9.1638783563	-1.0142527060	-2.1444921082
F	-8.5146365609	2.3225250402	1.7188630564
F	-6.8605954704	1.8073274893	3.0060170927
F	-8.4903208058	0.4227660010	2.7268759239

^{t}Bu$_2$Py

C	-2.5343340515	0.1528172902	-0.0078601163
C	-1.1355507907	0.1681000698	-0.0118892089
C	-0.4802068178	1.3920725642	-0.0100037871
C	-1.2268697822	2.5625733400	-0.0045631730
C	-2.6225381860	2.4711863072	-0.0008560518
N	-3.2327819799	1.2870278455	-0.0023096148
H	0.6116277611	1.4335370429	-0.0128980253
H	-0.5650259819	-0.7614840350	-0.0164008951
H	-0.7308846124	3.5338764467	-0.0021006713
C	-3.4941080191	3.7323568862	0.0071794355
C	-3.3075798518	-1.1717923055	-0.0042781045
C	-3.1842662718	4.5552252617	1.2691896822
H	-2.1336717003	4.8797326801	1.3027002462
H	-3.8133509238	5.4582652109	1.3001213367
H	-3.3857202889	3.9705692228	2.1795720446
C	-3.1854281643	4.5743706052	-1.2423257725
H	-3.3978433118	4.0078060429	-2.1615813805
H	-3.8073912634	5.4827898811	-1.2524444210
H	-2.1326142413	4.8912846110	-1.2773562453
C	-4.9798166871	3.3671333582	0.0047227305
H	-5.2445416967	2.7733840370	-0.8802163853
H	-5.2466022898	2.7692876569	0.8863118595
H	-5.5870256421	4.2855107115	0.0063536167
C	-2.9540576864	-1.9578585186	1.2693854399
H	-3.2237507367	-1.3883287145	2.1715908206
H	-3.5027807735	-2.9123140470	1.2951625388
H	-1.8800616953	-2.1892585038	1.3253886653
C	-4.8163145653	-0.9197115837	-0.0324650295
H	-5.1106992431	-0.3586491429	-0.9293737807
H	-5.3547810927	-1.8801162777	-0.0265599392
H	-5.1395777166	-0.3313588804	0.8362044126
C	-2.9155037569	-1.9964596167	-1.2413476809
H	-3.1479914364	-1.4514684027	-2.1686994265
H	-1.8429153755	-2.2401751976	-1.2532535955
H	-3.4721511738	-2.9463702549	-1.2582223781

^{t}Bu$_2$Py-H$^+$

C	-2.4931788989	0.1059961181	-0.0166382011
C	-1.1069648765	0.1570983812	-0.0232562535
C	-0.4652820933	1.3917762057	-0.0216658217
C	-1.1995652666	2.5736766899	-0.0124619673
C	-2.5857103221	2.5183854345	-0.0060738897
N	-3.1474210008	1.2888062963	-0.0095700722
H	0.6260873456	1.4335652958	-0.0261953150
H	-0.5301675334	-0.7668452642	-0.0281538886
H	-0.6965599912	3.5396785323	-0.0102198809
C	-3.4871730384	3.7470334821	0.0108378000
C	-3.2991877920	-1.1881727905	-0.0033625575
C	-3.1834829017	4.5532361793	1.2876294110
H	-2.1399211778	4.8963317677	1.3188631786
H	-3.8259338325	5.4444673975	1.3200698935
H	-3.3777324648	3.9623659968	2.1950308261
C	-3.1796797535	4.5988405480	-1.2346749139
H	-3.3784577181	4.0440835659	-2.1635421007
H	-3.8173456914	5.4940438374	-1.2315368616
H	-2.1345898264	4.9378821481	-1.2544538250
C	-4.9711908416	3.3606591638	0.0010303246
H	-5.2564334399	2.8068277751	-0.9079549383
H	-5.2615559783	2.7793308102	0.8911566494
H	-5.5822042057	4.2726278323	0.0139609761
C	-2.9576299392	-1.9516780936	1.2898783529
H	-3.2158815184	-1.3668290941	2.1851250833
H	-3.5266338324	-2.8916924340	1.3246847018
H	-1.8904650493	-2.2085744345	1.3448455538
C	-4.8075657660	-0.9146898972	-0.0452697998
H	-5.1140519087	-0.3818312749	-0.9597924684
H	-5.3498507458	-1.8694007913	-0.0455318123
H	-5.1614073726	-0.3594956782	0.8386739534
C	-2.9044113668	-2.0279017378	-1.2315148252
H	-3.1427359563	-1.5076885180	-2.1708512585
H	-1.8333964364	-2.2734415185	-1.2373323756
H	-3.4591635966	-2.9767948966	-1.2193785189
H	-4.1663497462	1.2490607846	-0.0074406005

1h•+…2c

C	3.7835250002	-0.1830792521	3.0484710703
C	2.6234563273	-0.1160825839	2.2991924597
C	1.3499071301	-0.0506938425	2.9081109415
C	1.2911821428	-0.0655691425	4.3434750387
C	2.4989546955	-0.1218980864	5.0815929137
C	3.7237222611	-0.1821605906	4.4479627231
C	0.1408505356	0.0384097582	2.1488290215
C	0.0389171973	-0.0070844934	5.0091241112
C	-1.1618835094	0.0845959179	4.2581317537
C	-1.1189516420	0.1194995369	2.8221437104
C	-2.3460332922	0.2210835603	2.1285332214
H	-2.3378030867	0.2581228434	1.0406877000
C	-3.5556410375	0.2736401782	2.7958791625
C	-3.5952055278	0.2190287894	4.1950915548
C	-2.4186649987	0.1237212593	4.9106738399
H	4.7494425597	-0.2311925538	2.5426215901
H	2.6945881117	-0.1097040741	1.2129448618
H	2.4582836444	-0.1112494748	6.1699740955
H	4.6411482287	-0.2255400474	5.0373763367
H	-4.4824709809	0.3522857958	2.2248656022
H	-4.5518939136	0.2488302437	4.7192594249
H	-2.4531345086	0.0725492942	5.9980599052
I	0.2089086156	0.0239468398	0.0536094473
C	-0.0123663646	-0.0421696066	6.4971251728
C	0.1778453094	-1.2469725435	7.1793311114
C	-0.2442554865	1.1277283803	7.2235614235
C	0.1237116031	-1.2769745878	8.5692782851
H	0.3670120605	-2.1696386214	6.6275899664
C	-0.2709172325	1.0909493314	8.6150636868
H	-0.3972725710	2.0769234261	6.7064342600
C	-0.0937715327	-0.1092093322	9.2942608792
H	-0.1217389508	-0.1337775426	10.3838388858
C	-0.4440910036	2.3821251492	9.3778497552
C	0.2651683872	-2.5974128734	9.2877226131
F	0.7037675272	3.0613736870	9.4208873248
F	1.3495066831	3.1669905927	8.7876300641
F	0.8368326274	2.1619928971	10.6292654149
F	0.6885336894	-2.4256124570	10.5372946411
F	1.1329223744	-3.3941255815	8.6585077587
F	0.9033676929	-3.2390994619	9.3362080945
N	1.7615091372	1.0763659438	-3.7331182084
C	2.5043340463	1.6218016469	-4.8082982549
C	3.0294148357	2.9204068885	-4.6458053691
C	2.7852863270	0.8965109958	-5.9688132295
C	3.8338848707	3.4471728931	-5.6605233574
C	3.5700900224	1.4550509144	-6.9710698777
C	4.1012516044	2.7314725951	-6.8211775823
H	2.3919837192	-0.1100855151	-6.0801188977
H	4.2371922232	4.4541726618	-5.5364768683
H	3.7756127156	0.8766410199	-7.8739338335
H	4.7206034572	3.1703252085	-7.6052471900
C	0.7582797999	0.1590956433	-3.6939242480
O	0.1932014893	-0.1230753876	-2.6419745834
O	0.4685963823	-0.3700309624	-4.8660068774
C	-0.6154097036	-1.3397520871	-5.0680056835
C	-1.9471788935	-0.6941785781	-4.7056555622
H	-2.0910103634	0.2337344164	-5.2780650372
H	-2.7676237507	-1.3803700367	-4.9598097382
H	-1.9996276503	-0.4660724432	-3.6346918066
C	-0.3386603215	-2.6019991034	-4.2611559377
H	-1.0831514070	-3.3689954801	-4.5184193407
H	0.6566458249	-3.0024155987	-4.5035178520
H	-0.3918121044	-2.4073758528	-3.1840999172
C	-0.5396363975	-1.6231539115	-6.5612551596
H	0.4303779279	-2.0677156957	-6.8254417070
H	-1.3319156611	-2.3274452934	-6.8504488682
H	-0.6675282599	-0.6971748974	-7.1388029010
C	2.7686503158	3.7355185872	-3.4241680779
C	1.4643074569	4.0706370217	-3.0335812136
C	3.8376548942	4.2237973538	-2.6621635685
C	1.2383379895	4.8687983348	-1.9157192653
C	3.6117656957	5.0226141924	-1.5444673331
C	2.3113633466	5.3473183677	-1.1674267058
H	0.6186627702	3.7213766254	-3.6300214482
H	4.8593824058	3.9713701316	-2.9540529865
H	0.2156879598	5.1333462588	-1.6379807350
H	4.4596833138	5.3976108457	-0.9672534229
H	2.1338436282	5.9815927605	-0.2963846617
H	1.8926001208	1.5392948156	-2.8407060326

1h…2c•

C	-0.9772380640	3.1449509115	-0.6043622258
C	-0.4190957836	1.9184235085	-0.4005362018
C	-1.2220733609	0.7445741107	-0.2140809096
C	-2.6537921589	0.9030427406	-0.2469043478
C	-3.1954754803	2.2131993130	-0.4663109855
C	-2.3901443049	3.2978466857	-0.6398330604
C	-0.6815443401	-0.5383093581	-0.0019373654
C	-3.4908548626	-0.2113111697	-0.0654767939
C	-2.9408770612	-1.4863138602	0.1517808105
C	-1.5109649904	-1.6603690324	0.1870360291
C	-1.0084674213	-2.9840305883	0.4143060341
H	0.0694952608	-3.1317149303	0.4469401623
C	-1.8403733094	-4.0487155284	0.5909857752
C	-3.2507618311	-3.8743489649	0.5520115404
C	-3.7776131309	-2.6363988450	0.3397422148
H	-0.3331569254	4.0163490876	-0.7391684687
H	0.6652165957	1.8230413485	-0.3743978374
H	-4.2783348001	2.3331117596	-0.4922640159
H	-2.8273924404	4.2847425453	-0.8044093873
H	-1.4215212568	-5.0422384529	0.7630397088
H	-3.9092029813	-4.7339508355	0.6924703702
H	-4.8590811075	-2.5058079418	0.3096721938
I	1.4291026304	-0.7852464797	0.0139975922
C	-4.9750940774	-0.0398353004	-0.1031753438
C	-5.6922334005	0.2133664944	1.0708861087
C	-5.6736834150	-0.1371197936	-1.3062823830
C	-7.0739771028	0.3623574630	1.0368374291
H	-5.1627631502	0.2907021209	2.0211303421
C	-7.0592806032	0.0186572382	-1.3370196576
H	-5.1312106021	-0.3452637530	-2.2299553578
C	-7.7668420785	0.2666184206	-0.1679240698
H	-8.8517168271	0.3764273112	-0.1934428488
C	-7.7880307691	-0.0479149258	-2.6526267962
C	-7.8566346582	0.6042530748	2.2995769063
F	-7.8132932196	1.1443404183	-3.2597197070
F	-7.2030620021	-0.9043627894	-3.4946933943
F	-9.0567532642	-0.4348661966	-2.4923284713
F	-8.6261084312	1.6932459608	2.1924172527
F	-7.0630382207	0.7729537204	3.3590635955
F	-8.6721146295	-0.4221061561	2.5669013372
N	5.0935448202	0.8617702138	0.9607723730
C	4.4632859238	1.9050103664	0.4276026146
C	4.1408549489	3.0238697274	1.2771458261
C	4.1365649190	1.9844107627	-0.9628435257
C	3.5657840363	4.1520092003	0.6947602240
C	3.5617228691	3.1144543127	-1.4965388740
C	3.2797758960	4.2081761746	-0.6664390918
H	4.3504383039	1.1234446474	-1.5972203771
H	3.3092416066	4.9998967624	1.3320214482
H	3.3227150474	3.1572319865	-2.5608065050
H	2.8216508151	5.1065559757	-1.0856664681
C	5.3181427205	-0.3085240629	0.2702560172
O	4.4352578115	-1.0669912857	-0.0781659407
O	6.6176166018	-0.5266186945	0.1236853073
C	7.1483254844	-1.7560026746	-0.4577325184
C	6.6738750084	-1.9000556095	-1.9004976514
H	6.9291742358	-0.9982750838	-2.4768255119
H	7.1792339846	-2.7568821056	-2.3692417560
H	5.5904411149	-2.0607736249	-1.9480588499
C	6.7447314968	-2.9479413837	0.4034720761
H	7.2382056180	-3.8566419183	0.0290885487
H	7.0615743942	-2.7902988403	1.4448446080
H	5.6595265686	-3.1023599983	0.3819559098
C	8.6533237355	-1.5317675892	-0.4033174288
H	8.9789839111	-1.3703627472	0.6337464616
H	9.1837960253	-2.4051728517	-0.8082204144
H	8.9315001312	-0.6471560854	-0.9933898912
C	4.4081301326	3.0000184507	2.7329475789
C	4.0663016547	1.8937956212	3.5236712359
C	4.9733701873	4.1199865692	3.3576783185
C	4.2813810158	1.9142792970	4.8967348914
C	5.1989122548	4.1340259682	4.7300928236
C	4.8511831071	3.0308108456	5.5046410032
H	3.6156087614	1.0188822597	3.0563348540
H	5.2622809200	4.9829460171	2.7541159052
H	3.9992906154	1.0480692775	5.4989513113
H	5.6524322173	5.0114074691	5.1960795137
H	5.0241540223	3.0404648952	6.5829512103

1h…2c⁺

C	-0.7569488618	2.4768238405	-2.4803166395
C	-0.1991354552	1.3939438369	-1.8671189336
C	-0.9749308221	0.5029978075	-1.0548466514
C	-2.3828204279	0.7782101511	-0.9194692574
C	-2.9204115995	1.9384586914	-1.5697151042
C	-2.1407497866	2.7630989624	-2.3228087230
C	-0.4473070865	-0.6205321047	-0.3965696654
C	-3.2015501904	-0.0799988320	-0.1638686164
C	-2.6560966089	-1.2112837820	0.4685107260
C	-1.2465333085	-1.4949116056	0.3602034775
C	-0.7492525710	-2.6624461888	1.0261757668
H	0.3095393846	-2.9041927376	0.9497528980
C	-1.5708505413	-3.4857972831	1.7366583525
C	-2.9611091624	-3.2076310258	1.8389155557
C	-3.4804377376	-2.1089685570	1.2242831028
H	-0.1430470032	3.1221473407	-3.1130445669
H	0.8578498764	1.1754834057	-2.0121439359
H	-3.9824146000	2.1535061552	-1.4557458491
H	-2.5768789971	3.6347243500	-2.8139793644
H	-1.1594011218	-4.3714930641	2.2246538298
H	-3.6098615027	-3.8787333040	2.4045239733
H	-4.5471925269	-1.9006039782	1.2971967737
I	1.6405012076	-1.0203492076	-0.5424216273
C	-4.6637908468	0.2056895030	-0.0423310950
C	-5.1685087222	0.8732353296	1.0748543683
C	-5.5502848163	-0.2007974648	-1.0421833802
C	-6.5327126889	1.1285756544	1.1885588757
H	-4.4930142810	1.1872402034	1.8723843741
C	-6.9130967028	0.0581736365	-0.9243706202
H	-5.1752419747	-0.7336818652	-1.9176613599
C	-7.4130322803	0.7189553604	0.1929897311
H	-8.4829970505	0.9025605191	0.2924329719
C	-7.8467489376	-0.3416731894	-2.0379238675
C	-7.0482763462	1.8892530859	2.3827829600
F	-7.9121990133	0.6115735807	-2.9730415599
F	-7.4357889698	-1.4596744278	-2.6415845728
F	-9.0851965601	-0.5468668685	-1.5889188067
F	-6.9580692385	3.2094604153	2.1897040591
F	-6.3427992341	1.6049624519	3.4825724821
F	-8.3268356122	1.6072336975	2.6299600834
N	4.7544708438	0.6806883109	1.1743390198
C	3.8773314139	1.4934881269	0.7139824030
C	2.9999277643	2.1935245938	1.6887704142
C	3.8001337381	1.8595522879	-0.7027698623
C	2.3137687369	3.3264207662	1.2224866300
C	3.0960983725	2.9488270728	-1.0858319406
C	2.3579428597	3.6846008018	-0.1087303311
H	4.3731892499	1.2693784003	-1.4189615686
H	1.6704748451	3.8864970397	1.9000383561
H	3.0690925951	3.2639091333	-2.1298316885
H	1.7720882424	4.5463124193	-0.4386525334
C	5.5339600521	-0.1352058010	0.3245823122
O	5.0389686428	-1.1336555320	-0.1323781064
O	6.7471230929	0.3213194847	0.2182033589
C	7.8236266668	-0.4088864746	-0.5064597677
C	7.4220381033	-0.5602034609	-1.9666090185
H	7.1939030940	0.4208948583	-2.4095401365
H	8.2623955860	-0.9912424417	-2.5284366164
H	6.5555930619	-1.2230846785	-2.0809047040
C	8.0539987111	-1.7455827292	0.1828162375
H	8.9134323424	-2.2472793174	-0.2837368174
H	8.2860742773	-1.5962383808	1.2470078092
H	7.1810509169	-2.4038884404	0.0920342728
C	9.0177233005	0.5160387693	-0.3481262625
H	9.2577364443	0.6640869404	0.7137186585
H	9.8942077067	0.0785717675	-0.8453575813
H	8.8152736436	1.4967636643	-0.8009790294
C	2.8706584058	1.7320206445	3.0454294874
C	2.9791299012	0.3588105831	3.3785259840
C	2.5999745411	2.6623441887	4.0806260330
C	2.8396068842	-0.0531577827	4.6935453576
C	2.4872690998	2.2429884940	5.3909801939
C	2.6040346929	0.8829596345	5.6995780253
H	3.1135807338	-0.3820754107	2.5928176368
H	2.5377459419	3.7274780299	3.8553455873
H	2.9020765325	-1.1147205788	4.9373648315
H	2.3114634202	2.9714807647	6.1839490058
H	2.4994286006	0.5527424959	6.7352988623

1h⁺–2c

C	-0.7896203780	2.8316805979	-2.0720842056
C	-0.2295257927	1.7999337565	-1.3718198665
C	-1.0362102282	0.8220113391	-0.7186132369
C	-2.4660220397	0.9746357153	-0.7871660282
C	-3.0060766624	2.0611164102	-1.5470973826
C	-2.1975726631	2.9624838263	-2.1739217562
C	-0.5432768689	-0.2874950219	0.0083716910
C	-3.3093371183	0.0630729230	-0.1181323906
C	-2.7820332639	-0.9974460244	0.6459043823
C	-1.3570162019	-1.1855776437	0.7385428278
C	-0.8697000333	-2.2445602337	1.5597249763
H	0.2008720588	-2.3656924097	1.7161179140
C	-1.7306356028	-3.0804990669	2.2146980131
C	-3.1351000298	-2.9176209554	2.1050696061
C	-3.6408540935	-1.9008336761	1.3510568591
H	-0.1421520001	3.5654196192	-2.5558668964
H	0.8566270115	1.7440532409	-1.3024717588
H	-4.0883320519	2.1606588970	-1.6176910507
H	-2.6284378831	3.7848801827	-2.7467395737
H	-1.3267539414	-3.8761923859	2.8436135642
H	-3.8052804430	-3.5962349528	2.6348965027
H	-4.7183436543	-1.7606307944	1.2797470701
I	1.5004350293	-0.7240487994	-0.1542070901
C	-4.7914160494	0.2337864805	-0.2034367730
C	-5.4446456239	1.1468212630	0.6267365997
C	-5.5407372894	-0.5328025685	-1.0966400294
C	-6.8273736248	1.2886161226	0.5611640938
H	-4.8746109271	1.7445574688	1.3399552374
C	-6.9244250457	-0.3851456318	-1.1564203285
H	-5.0479292347	-1.2614992542	-1.7429329269
C	-7.5752434521	0.5204807155	-0.3261802349
H	-8.6605545559	0.6184187163	-0.3608855548
C	-7.7087311395	-1.2000014905	-2.1552107220
C	-7.5109853762	2.3118754735	1.4343879309
F	-7.6639256207	-0.6415621475	-3.3669275431
F	-7.2056403520	-2.4325619283	-2.2669510667
F	-8.9884500890	-1.3051957001	-1.8036906894
F	-7.5452681787	3.5044152889	0.8346082442
F	-6.8617440535	2.4678942478	2.5916475672
F	-8.7656830441	1.9599868039	1.7066837930
N	2.5391027739	0.2773505237	1.3043065098
C	3.0728485817	1.5729403506	1.0421256713
C	2.6335061390	2.7104852014	1.7516303212
C	4.0623245108	1.6822935558	0.0590082861
C	3.2295212679	3.9359047436	1.4234341622
C	4.6187043443	2.9120437770	-0.2588723467
C	4.1953623620	4.0455997876	0.4307599519
H	4.4072009903	0.7793968378	-0.4499908954
H	2.9026687773	4.8309813723	1.9550180270
H	5.3873061337	2.9835945399	-1.0301386038
H	4.6215685142	5.0227339203	0.1957468697
C	2.6696717170	-0.4660751141	2.4992016579
O	2.0939696629	-1.5160198425	2.6501293088
O	3.4836827298	0.1408165700	3.3182428678
C	3.7978745116	-0.3965658473	4.6679830317
C	4.4876984723	-1.7440853413	4.5159534232
H	5.3823566785	-1.6537406697	3.8827221361
H	4.8099124555	-2.0957627962	5.5062643206
H	3.8138858793	-2.4945668854	4.0856884297
C	2.5147420848	-0.4783254937	5.4817781068
H	2.7633390501	-0.7594157964	6.5150489079
H	2.0080455035	0.4969901207	5.5047874566
H	1.8279907663	-1.2306640260	5.0757965233
C	4.7450099570	0.6515278847	5.2271937787
H	4.2486276166	1.6294197974	5.2880960372
H	5.0653077315	0.3599092126	6.2368329311
H	5.6376252689	0.7486880634	4.5936948700
C	1.6381335230	2.6732249211	2.8569198830
C	0.3909196848	2.0520653086	2.7296306296
C	1.9388975419	3.3177089093	4.0646600519
C	-0.5224899395	2.0649533809	3.7800674824
C	1.0266951270	3.3316222286	5.1147198336
C	-0.2080808312	2.7021494755	4.9770720748
H	0.1134135913	1.5732278924	1.7918300980
H	2.9087068611	3.8041935556	4.1868822310
H	-1.4932717533	1.5801584335	3.6580920410
H	1.2832072572	3.8376210715	6.0476094861
H	-0.9261391307	2.7144914686	5.7992499562

2c

N	8.2606784395	1.1042661325	-0.4506563899
C	7.8044531671	2.2264648403	-1.1625079150
C	7.1012289144	3.2170113761	-0.4380102582
C	8.0568747242	2.4281307899	-2.5247856112
C	6.6910795734	4.3757625114	-1.1016045899
C	7.6196663142	3.5843071090	-3.1611501272
C	6.9371606096	4.5685567689	-2.4559740029
H	8.6060169120	1.6740481016	-3.0793974435
H	6.1474655514	5.1334021388	-0.5330237247
H	7.8270153376	3.7143989379	-4.2256538048
H	6.5963262303	5.4780925532	-2.9538231863
C	8.7392940139	-0.1290413179	-0.8219054360
O	9.0544844335	-0.9522235511	0.0063091856
O	8.8030297273	-0.2965051449	-2.1437993470
C	9.2764730038	-1.5452494105	-2.7322426530
C	8.3532645653	-2.6909633000	-2.3313184591
H	7.3140461104	-2.4573420676	-2.6063658911
H	8.6503418535	-3.6062809048	-2.8637479841
H	8.4035578226	-2.8762663971	-1.2523020741
C	10.7233567218	-1.7984663192	-2.3211870296
H	11.1104100644	-2.6778419838	-2.8564778026
H	11.3502591012	-0.9340698045	-2.5856319758
H	10.7994663514	-1.9770772184	-1.2426941239
C	9.1814553655	-1.2765925432	-4.2284766209
H	9.8241971720	-0.4308438565	-4.5125614006
H	9.5056234220	-2.1622849839	-4.7930523609
H	8.1468995128	-1.0394916715	-4.5147916519
C	6.8024284915	3.0749226114	1.0168076647
C	6.0288446810	2.0133015645	1.5061251776
C	7.2638967412	4.0352222932	1.9258175810
C	5.7300435923	1.9150907320	2.8622353616
C	6.9635420895	3.9385446895	3.2814751307
C	6.1964525107	2.8773706118	3.7543529846
H	5.6470470387	1.2621798807	0.8111938648
H	7.8732719430	4.8643951625	1.5597876277
H	5.1220582983	1.0824610881	3.2224843670
H	7.3362031047	4.6965309094	3.9739797327
H	5.9623952913	2.7995379552	4.8181524724
H	8.1869918854	1.1674883922	0.5588931665

2c^{+}

N	5.3189129479	0.8841705891	0.9687402263
C	4.6922809451	1.8668964965	0.4337413628
C	4.5170899274	3.0895244558	1.2593580491
C	4.2881509734	1.8874180889	-0.9719402925
C	4.2267004302	4.2815770250	0.5684757218
C	3.9535972098	3.0578958424	-1.5587012521
C	3.9482161617	4.2609544361	-0.7795769685
H	4.3193738400	0.9528205121	-1.5345218180
H	4.1246541469	5.2173411868	1.1168518777
H	3.6893305843	3.1036993190	-2.6162919397
H	3.6716067013	5.1968965697	-1.2722460110
C	5.4741709287	-0.3720779380	0.3349010752
O	4.6188151482	-1.1985855564	0.4945401820
O	6.6130839906	-0.4396030997	-0.2915450533
C	7.1531640241	-1.7161819277	-0.8438115137
C	6.2296295874	-2.1981678346	-1.9529516978
H	6.1069356785	-1.4196113545	-2.7203905042
H	6.6763746507	-3.0770964191	-2.4385841615
H	5.2448771564	-2.4835302714	-1.5622053966
C	7.2859903657	-2.7195599365	0.2913715187
H	7.7813320278	-3.6248901742	-0.0863676034
H	7.9057200647	-2.3082961499	1.1012253482
H	6.3074554219	-3.0068320305	0.6957485156
C	8.5088035337	-1.2957731779	-1.3835042109
H	9.1362870006	-0.8848535501	-0.5805999403
H	9.0224168408	-2.1653745963	-1.8156492295
H	8.3982544755	-0.5333751869	-2.1671552131
C	4.6236012227	3.0384504774	2.6897720510
C	4.2917629448	1.8640699249	3.4154827081
C	5.0352290766	4.1888548503	3.4129968933
C	4.3898279863	1.8442848466	4.7963606835
C	5.1524434266	4.1490126572	4.7869305946
C	4.8269571703	2.9770984637	5.4816468847
H	3.9097647537	0.9885593218	2.8943396657
H	5.3190870906	5.0969140218	2.8802519523
H	4.1117101185	0.9441252950	5.3464567932
H	5.5011781284	5.0281569967	5.3308588882
H	4.9065250456	2.9549633777	6.5707467517

2c$^{\bullet}$

N	5.2468212029	0.8840798307	0.8251498206
C	4.5732127982	1.9287423655	0.3434357625
C	4.2953725184	3.0311818659	1.2297856064
C	4.1626966704	2.0311823613	-1.0230248628
C	3.6691123250	4.1608712404	0.7056956439
C	3.5404437278	3.1626808486	-1.4977489445
C	3.2950767947	4.2365947205	-0.6326571961
H	4.3403099445	1.1811620483	-1.6809855052
H	3.4436327646	4.9952089760	1.3719387256
H	3.2345051737	3.2207372582	-2.5441595515
H	2.7955577887	5.1339306664	-1.0039240854
C	5.4484824935	-0.2674673353	0.0868957974
O	4.5626369957	-0.9261588450	-0.4099245470
O	6.7428474333	-0.5779272920	0.0771438618
C	7.2457472770	-1.7897731205	-0.5545235599
C	6.9385619360	-1.7691064999	-2.0490003232
H	7.3180010039	-0.8407812017	-2.5014280324
H	7.4384448163	-2.6165360969	-2.5404096282
H	5.8600461791	-1.8412775841	-2.2327452575
C	6.6582123163	-3.0167886762	0.1357214619
H	7.1241614765	-3.9268281802	-0.2698032831
H	6.8621572670	-2.9791798254	1.2158989488
H	5.5745271776	-3.0745165505	-0.0193343330
C	8.7466651867	-1.7007785848	-0.3115141803
H	8.9572403724	-1.6619222455	0.7663934284
H	9.2556829574	-2.5759555449	-0.7397202225
H	9.1580372368	-0.7927746683	-0.7747878246
C	4.6677844237	2.9999893368	2.6634247836
C	4.4131403561	1.8814478243	3.4694377180
C	5.2509593370	4.1286740626	3.2554063072
C	4.7267941032	1.8988817204	4.8234264118
C	5.5749071229	4.1408742043	4.6081752915
C	5.3111187586	3.0255435682	5.3979363626
H	3.9574591926	0.9958836543	3.0286720742
H	5.4768828769	5.0017287107	2.6396937751
H	4.5123063132	1.0213563786	5.4371602365
H	6.0403298184	5.0265510236	5.0459814245
H	5.5619595944	3.0327867397	6.4608794686

3cH^{+}

N	5.0149210473	1.8080845851	1.5109274474
C	3.9463407721	1.7576901359	0.6406188871
C	2.8124155657	1.1858645295	1.2946343241
C	3.8649322710	2.1614383645	-0.7002245314
C	1.5796002007	1.0347993711	0.6115884385
C	2.6477143971	1.9957649009	-1.3334713006
C	1.5085677676	1.4392337262	-0.6954984259
H	4.7245984196	2.5945983919	-1.2038682243
H	0.7149767801	0.6034766590	1.1185012469
H	2.5581129497	2.3129668456	-2.3750395415
H	0.5783800780	1.3374046778	-1.2557857922
C	6.3327302430	2.1662750978	1.1367175033
O	6.5751791304	2.6498338577	0.0682708991
O	7.1657628790	1.8815802532	2.1144733084
C	8.6287145377	2.1542646819	2.0072867678
C	8.8433248232	3.6494760000	1.8263482307
H	8.3657114482	4.2129534265	2.6411830016
H	9.9212808749	3.8610005806	1.8578510417
H	8.4509308062	4.0025229630	0.8654274423
C	9.2011097544	1.3309387784	0.8632784972
H	10.2939423471	1.4469535042	0.8505980028
H	8.9776513730	0.2631440009	1.0023911798
H	8.8097598421	1.6595414867	-0.1067573022
C	9.1551706031	1.6764884824	3.3501770599
H	8.9327235894	0.6111682315	3.5045222770
H	10.2452554034	1.8066407070	3.3870372074
H	8.7191638726	2.2568091732	4.1764492544
C	3.1791261139	0.9060043309	2.6160967594
C	4.6353538423	1.1641723277	2.7570946962
C	2.4258817373	0.5376108188	3.7516662645
C	5.0836944347	1.6052080793	4.1094435069
C	2.9811483838	0.7477032053	4.9870823110
C	4.2922589555	1.3211418729	5.1658034986
H	5.0675001903	0.1302642743	2.7262913655
H	1.3908510999	0.2087136164	3.6509148611
H	6.0908316173	2.0035973485	4.2192857285
H	2.3899910156	0.5264571846	5.8793021891
H	4.6360011530	1.5351971536	6.1789558085

3c

N	5.1515111229	2.1644588274	2.2470175617
C	3.8802293027	1.6765470699	1.8607879261
C	3.0907443030	1.5167277165	3.0132453560
C	3.3804700468	1.3676905551	0.5949198072
C	1.7828037859	1.0431387881	2.9108555354
C	2.0740007993	0.8956447257	0.5152600000
C	1.2783407980	0.7328931301	1.6551217320
H	3.9955575482	1.4937061540	-0.2910435733
H	1.1690363448	0.9197522171	3.8054913540
H	1.6630740596	0.6472212195	-0.4657054294
H	0.2568680430	0.3609846375	1.5539754087
C	6.1688959591	2.4232658249	1.3357765089
O	6.0404791805	2.2711697370	0.1466163430
O	7.2721487293	2.8474954517	1.9424091932
C	8.4831401549	3.1811606741	1.1924739976
C	8.2042527380	4.3485941412	0.2527354378
H	7.7967960335	5.2024276730	0.8138879444
H	9.1435475065	4.6681808095	-0.2214542169
H	7.4924963897	4.0653473195	-0.5313627848
C	8.9909218027	1.9485242411	0.4515398441
H	9.9654443179	2.1728376315	-0.0059016403
H	9.1262779379	1.1115002719	1.1521452337
H	8.2927671750	1.6437080263	-0.3362619548
C	9.4592297440	3.5891588741	2.2878925331
H	9.6213743775	2.7588129553	2.9898421186
H	10.4273215106	3.8657999387	1.8473871164
H	9.0745177653	4.4523395829	2.8499058536
C	3.8986532927	1.9199719922	4.1495000293
C	5.1586844728	2.3128112409	3.6559495858
C	3.6271981019	1.9683890104	5.5164195169
C	6.1549106825	2.7572590227	4.5267173267
C	4.6162573691	2.4098418121	6.3847573222
C	5.8640540504	2.7984792261	5.8868163231
H	2.6491249351	1.6629999877	5.8938886335
H	7.1283308021	3.0622017865	4.1586200325
H	4.4217040653	2.4545435594	7.4580417329
H	6.6349361460	3.1444575730	6.5788238888

[**1h**$^{\cdot+}$…**2c**]$_{X}$TfO^{-}

C	-3.72467	-4.64833	6.08267
C	-4.84122	-4.75127	5.24322
C	-4.70089	-5.38211	3.98492
C	-3.44194	-5.85525	3.60267
C	-2.33767	-5.75511	4.44185
C	-2.49233	-5.15497	5.68768
H	-3.83296	-4.16211	7.04725
H	-3.34285	-6.33478	2.62663
H	-1.36708	-6.14064	4.12408
H	-1.63911	-5.06202	6.36320
C	-5.85202	-5.56185	3.05344
C	-7.00064	-6.26350	3.44880
C	-5.78563	-5.07731	1.74148
C	-8.04766	-6.47403	2.55553
H	-7.06728	-6.65298	4.46661
C	-6.83316	-5.28772	0.84843
H	-4.90159	-4.52066	1.42304
C	-7.96751	-5.98756	1.25212
H	-8.93331	-7.02374	2.88135
H	-6.76530	-4.89481	-0.16846
H	-8.79206	-6.14839	0.55439
N	-6.08912	-4.20041	5.56493
H	-6.74236	-4.10411	4.79559
C	-6.69399	-3.86762	6.75721
O	-7.82237	-3.43581	6.78209
O	-5.91511	-4.07571	7.81648
C	-6.38022	-3.80611	9.17559
C	-5.18858	-4.22011	10.02911
H	-5.42562	-4.09736	11.09532
H	-4.93324	-5.27186	9.84123
H	-4.30962	-3.60112	9.79679
C	-7.59723	-4.66774	9.49758
H	-7.87636	-4.52186	10.55132
H	-8.44985	-4.39523	8.86531
H	-7.36884	-5.73226	9.34979
C	-6.67370	-2.31796	9.33153
H	-7.52175	-2.01744	8.70519
H	-6.91212	-2.09766	10.38231
H	-5.79252	-1.72280	9.04930
C	-4.09046	-8.74534	0.13663
C	-2.77623	-9.05495	0.42766
C	-2.38024	-9.43927	1.72524
C	-3.39051	-9.51330	2.74115
C	-4.72631	-9.19358	2.40778
C	-5.07316	-8.80846	1.13032
C	-1.04594	-9.80276	2.05641
C	-3.05952	-9.94060	4.04652
C	-1.71701	-10.26880	4.37699
C	-0.69351	-10.20748	3.37891
C	0.61665	-10.57852	3.74938
H	1.40795	-10.55540	3.00268
C	0.91461	-10.98467	5.03453
C	-0.08894	-11.03969	6.01073
C	-1.38189	-10.68933	5.68285
H	-4.36140	-8.45972	-0.88118
H	-2.03350	-9.01645	-0.36669
H	-5.49450	-9.25790	3.17610
H	-6.10963	-8.55859	0.89992
H	1.93762	-11.27126	5.28467
H	0.14767	-11.36794	7.02423
H	-2.16580	-10.74545	6.43666
C	-4.12642	-10.04319	5.08294
C	-4.45028	-8.93363	5.86448
C	-4.80582	-11.25196	5.26529
C	-5.45325	-9.03627	6.82703
H	-3.92258	-7.98738	5.72373
C	-5.80328	-11.33513	6.23322
H	-4.52584	-12.10482	4.63674
C	-6.13110	-10.23483	7.01940
H	-6.90579	-10.31417	7.78138
C	-6.55675	-12.63071	6.39782
C	-5.82423	-7.81666	7.62380
I	0.43765	-9.70960	0.58822
F	-5.73961	-13.68101	6.34558
F	-7.46937	-12.78419	5.43460
F	-7.20442	-12.67438	7.56856
F	-6.54214	-8.12104	8.70849
F	-4.74343	-7.14394	8.02962
F	-6.56513	-6.96615	6.89153
S	-2.97851	-12.71426	1.81708
O	-3.13299	-12.73339	3.29121
O	-1.61093	-12.38540	1.37216
O	-4.05674	-12.02775	1.09661
C	-3.17359	-14.48942	1.35232
F	-2.24621	-15.23499	1.94388
F	-3.05678	-14.64129	0.03730
F	-4.36911	-14.93713	1.72145

[**1h**$^{\cdot+}$…**2c**]$_{\pi}$TfO^{-}

C	1.85498	-3.47035	2.05196
C	1.14738	-2.73573	1.12208
C	0.35575	-1.63008	1.50170
C	0.30327	-1.28821	2.89039
C	1.04635	-2.05284	3.81809
C	1.80777	-3.12743	3.41004
C	-0.36546	-0.83510	0.55794
C	-0.46334	-0.17378	3.32391
C	-1.20277	0.59062	2.39167
C	-1.15627	0.26959	0.99499
C	-1.86891	1.09922	0.10499
H	-1.83396	0.88849	-0.96187
C	-2.59338	2.18587	0.55288
C	-2.64298	2.49290	1.91713
C	-1.95901	1.70707	2.81856
H	2.46121	-4.31582	1.72187
H	1.20888	-3.00813	0.07011
H	1.02172	-1.77603	4.87104
H	2.37825	-3.70163	4.14206
H	-3.11967	2.81390	-0.16798
H	-3.20659	3.35958	2.26580
H	-1.98271	1.95536	3.87843
I	-0.22185	-1.25745	-1.47725
C	-0.50248	0.18176	4.77152
C	-1.42371	-0.43975	5.61544
C	0.37784	1.13866	5.28561
C	-1.47152	-0.10220	6.96575
H	-2.10908	-1.19308	5.22261
C	0.32539	1.45629	6.64050
H	1.09392	1.60742	4.60221
C	-0.59572	0.84475	7.48530
H	-0.63009	1.10488	8.54312
C	1.31636	2.44403	7.20197
C	-2.51247	-0.73466	7.84957
F	2.48856	1.85606	7.46202
F	1.54924	3.44184	6.35244
F	0.87870	2.97565	8.34977
F	-2.14015	-0.73409	9.13119
F	-2.75098	-2.00170	7.49346
F	-3.67829	-0.08224	7.77481
N	1.73908	-0.24671	-5.76289
C	2.49358	0.52829	-4.85521
C	3.80892	0.89489	-5.20549
C	1.94686	0.97031	-3.64981
C	4.53017	1.70114	-4.31780
C	2.69687	1.73608	-2.76551
C	3.99408	2.10934	-3.10150
H	0.92260	0.70662	-3.40569
H	5.55057	1.98383	-4.58611
H	2.26769	2.01560	-1.80289
H	4.58381	2.71132	-2.40797
C	0.70017	-1.08465	-5.49564
O	0.20007	-1.29225	-4.40737
O	0.30580	-1.65871	-6.64117
C	-0.79806	-2.60402	-6.70686
C	-2.09146	-1.93035	-6.25906
H	-2.26869	-1.01806	-6.84751
H	-2.93882	-2.61207	-6.42231
H	-2.05222	-1.66404	-5.19660
C	-0.47553	-3.84334	-5.87775
H	-1.25817	-4.60161	-6.02592
H	0.48392	-4.27605	-6.19753
H	-0.41640	-3.60141	-4.81039
C	-0.85923	-2.95120	-8.18879
H	0.09096	-3.39367	-8.51979
H	-1.66684	-3.67227	-8.37829
H	-1.04682	-2.04889	-8.78774
C	4.43996	0.46675	-6.48639
C	4.56106	-0.88789	-6.82771
C	4.96413	1.42080	-7.36816
C	5.18138	-1.27341	-8.01277
C	5.58675	1.03595	-8.55211
C	5.69610	-0.31286	-8.87982
H	4.17891	-1.64875	-6.14388
H	4.87293	2.48024	-7.11931
H	5.27220	-2.33456	-8.25538
H	5.98647	1.79724	-9.22568
H	6.18384	-0.61562	-9.80883
H	2.06465	-0.27278	-6.72253
S	1.71905	1.97446	1.43436
O	1.74884	1.01424	0.31412
O	1.93280	1.34652	2.75803
O	0.63870	2.96386	1.38547
C	3.26258	2.96099	1.18998
F	4.33861	2.18914	1.28513
F	3.35385	3.92518	2.09738
F	3.26624	3.52728	-0.02007

*3-3-6. Potential energy curves for **1a**-**1c** and **1a**$^{•+}$-**1c**$^{•+}$ complexed with **2c***

All calculations were performed using Gaussian 16 software. Geometry optimization and energy calculations were performed at CAM-B3LYP level of theory using LANL2DZ effective core potential for iodine, and 6-31+G(d,p) basis set for all other atoms at 298.15 K considering the solvent effect of acetonitrile. The potential energy change (ΔE) was calculated by placing the halogen atom of **1a-1c** or **1a**$^{•+}$**-1c**$^{•+}$ and the carbonyl oxygen of **2c** at a distance of 2.5 Å and varying the distance to 7 Å. All energy values are recorded as the difference from the energy value at a distance of 7 Å, which is the distance where the two molecules do not interact.

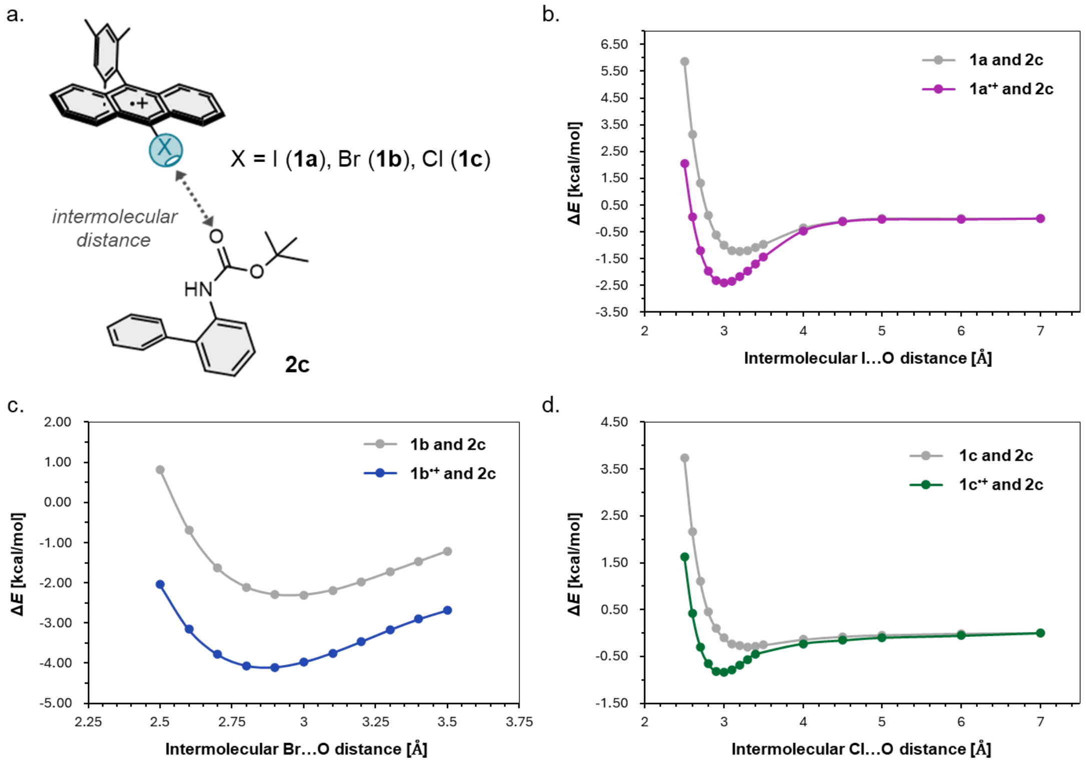

Figure S34. (a) Calculation image. (b) Potential energy curves for **1a** and **1a**$^{•+}$ complexed with **2c**. (c) Potential energy curves for **1b** and **1b**$^{•+}$ complexed with **2c**. (d) Potential energy curves for **1c** and **1c**$^{•+}$ complexed with **2c**.

3-4. Quantitative analysis of halogen-bonding interaction[20–22]

Halogen-bonded interactions are expressed using the association constant K as follows.

$$K = \frac{[\mathrm{HG}]}{[\mathrm{H}][\mathrm{G}]} \qquad \text{eq S1}$$

[HG] : host guest complex, [H] : host, [G] : guest

Although K can be determined by various titration techniques, **1a**$^{\bullet+}$ was found to decompose slowly under ambient conditions, causing difficulty in performing titration experiments. Therefore, binding enhancement factor (BEF), an index of change in the magnitude of halogen-bonding interactions due to redox response, was employed as a quantitative evaluation method for the interactions in the present study.[20–22] When the association constant of **1a** and **1a**$^{\bullet+}$ are K_{Red} and K_{Ox}, respectively, the BEF is expressed as follows.

$$\mathrm{BEF} = \frac{K_{\mathrm{Ox}}}{K_{\mathrm{Red}}} \qquad \text{eq S2}$$

If there are halogen-bonding interactions between **1a**$^{\bullet+}$ and anions, the voltammogram of **1a** is negatively shifted by the addition of anions. The maximum value of this shift, ΔE_{max}, is defined through a transformation from Nernst's equation as follows

$$\Delta E_{\mathrm{max}} = -\frac{RT}{nF}\ln\left(\frac{K_{\mathrm{Ox}}}{K_{\mathrm{Red}}}\right) \qquad \text{eq S3}$$

From eq S2 and eq S3,

$$BEF = 10^{\frac{\Delta E_{\mathrm{max}}\,[\mathrm{mV}]}{-59.0}} \qquad \text{eq S4}$$

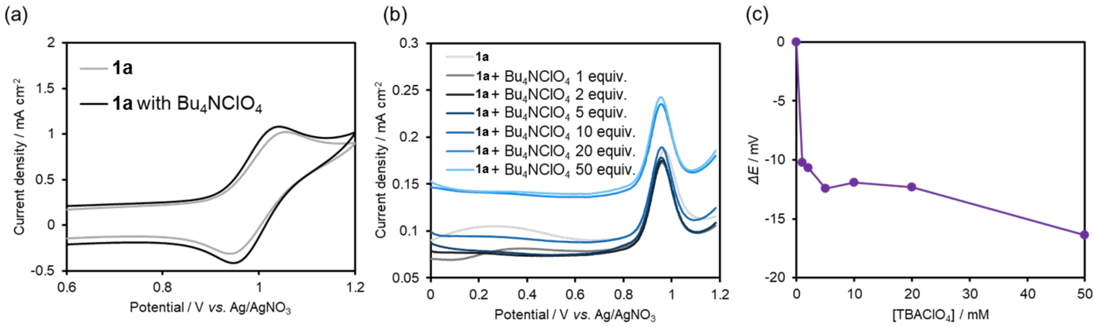

Figure S35. An experimental evaluation of halogen-bonding interactions. (a) Cyclic voltammograms of 1 mM **1a** (gray line) and 1 mM **1a** and 20 mM Bu_4NClO_4 (black line) in 0.1 M LiTfO/MeCN+CH_2Cl_2 (6:4 in vol.) solution at 0.1 V s^{-1}. (b) SWVs of 0.1 mM **1a** in the presence (X equiv.) and absence of Bu_4NClO_4 in 0.1 M LiTfO/MeCN+CH_2Cl_2 (6:4 in vol.) solution. (c) The shift of voltammogram ΔE_{max} calculated peak potentials of SWV. ΔE_{max} was defined as ΔE when the anion concentration was 50 mM.

3-5. Screening of electrolysis condition

The experimental procedure followed the method shown in General procedure for electrocatalytic intramolecular amination (2-11).

The nucleophilicity of the anions in the electrolyte did not make a significant difference with respect to the yield of the catalytic reaction (Table S4).

Table S4. Effect of type of supporting electrolyte on electrocatalytic intramolecular amination.

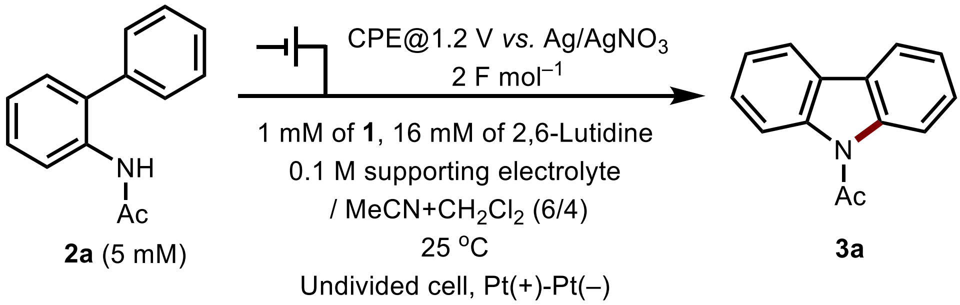

Entry	Supporting electrolyte	Yield of 3a
1	LiTfO	17%
2	LiTFSI	14%

When a glassy carbon electrode was used, the catalytic reaction hardly proceeded, suggesting that the use of a platinum electrode is preferable (Table S5).

Table S5. Effect of type of electrode on electrocatalytic intramolecular amination.

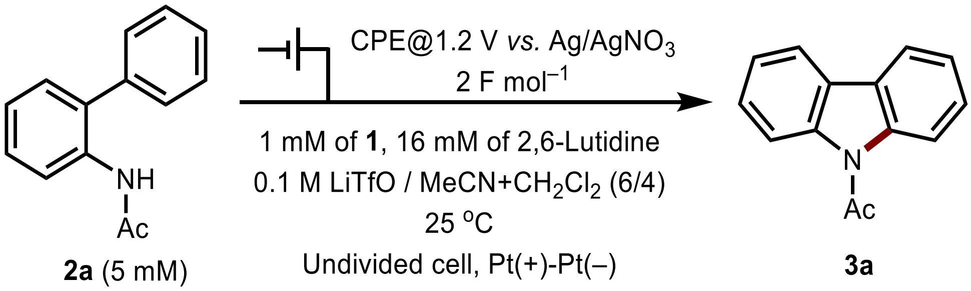

Entry	Electrode	Yield of 3a
1	Glassy carbon	3%
2	Pt	17%

The reaction did not proceed when LDA and LiHMDS were used. From these results, it was found that it is important that the nucleophilicity of the base is kept moderate by

steric hindrance and electron-withdrawing groups for the reaction to proceed well. The steric hindrance of the base also avoided side reactions due to the coordination of the base itself to the catalyst, which greatly contributed to the improvement of the reaction yield.

Table S6. Effect of type of base on electrocatalytic intramolecular amination.

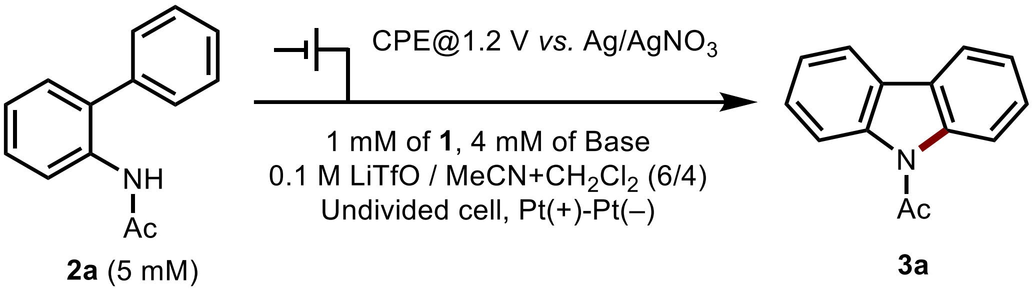

Entry	Base	Temperature	Charge Passed	Yield of 3a
1	2,6-Di-*tert*-butylpyridine	25 °C	2 F mol^{-1}	25%
2	-	25 °C	2 F mol^{-1}	25%
3	2,6-Difluoropyridine	25 °C	2 F mol^{-1}	22%
4	2,3,5,6-Tetrafluoropyridine	25 °C	2 F mol^{-1}	16%
5	Pentafluoropyridine	25 °C	2 F mol^{-1}	13%
6	LDA[a, b]	25 °C	2 F mol^{-1}	0%
7	Pottasium *tert*-butoxide[b]	25 °C	2 F mol^{-1}	13%
8	LiHMDS[b]	25 °C	2 F mol^{-1}	0%
9	2,6-Di-*tert*-butylpyridine	50 °C	5 F mol^{-1}	52%
10	2,6-difluoropyridine	50 °C	5 F mol^{-1}	34%
11	-	50 °C	5 F mol^{-1}	32%

[a] Electrolysis stopped at 0.5 F mol^{-1}. [b] Base concentration is 16 mM.

The yield did not improve when the applied potential was varied, suggesting that 1.2 V *vs.* Ag/$AgNO_3$ is the optimum potential.

Table S7. Effect of applied potential on electrocatalytic intramolecular amination.

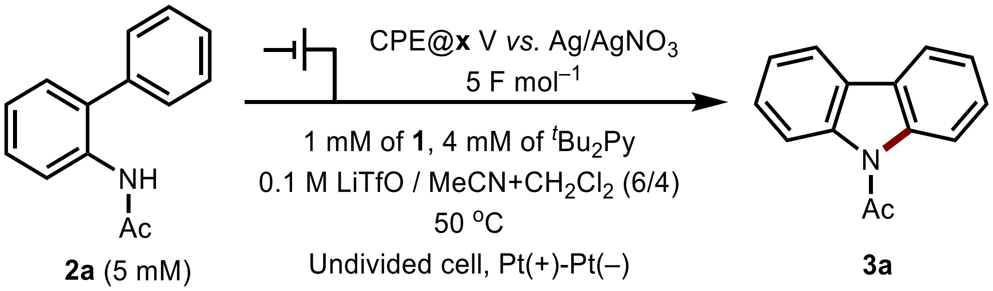

Entry	Potential	Yield of 3a
1	1.2 V	52%
2	1.1 V	42%
3	1.3 V	39%

3-6. Control experiments

The condition used in General procedure for electrocatalytic intramolecular amination (2-13) was set as standard condition, and control experiments were conducted under the conditions shown in Table S11. The current value decreased significantly without **1a**, resulting in passed charge of only 0.75 F/mol, and few **3c** were yielded. In constant current electrolysis without **1a**, the target product **3c** was obtained with a small amount of 18%. This is because **3c** is obtained by the direct oxidation of **2c**; however, the yield was significantly lower compared to the standard condition. Next, mediators **1i** and **1j**, in which the halogen at 9-position of the mediator was replaced with a methyl group, were used instead of **1a** for electrolysis. When **1i** and **1j**, which has almost the same oxidation potential as that of **1a**, were used, the yield remarkably decreased. Furthermore, since the target product was not obtained when chemical oxidizing agents were used, it was inferred that the reaction is promoted by the mediator with electrochemically activated halogen bonding interaction.

Table S8. Control experiments on electrocatalytic intramolecular amination

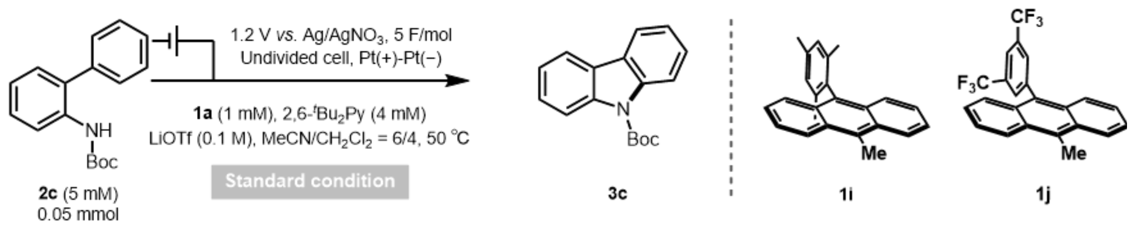

Entry	Deviation from standard condition	Yield of **3c**[a]
1	none	82%
2	No catalyst (**1a**), 0.75 F/mol	5%
3	No catalyst (**1a**), Constant current (4 mA), 2 F/mol	18%
4	**1i** instead of **1a**	27%
5	**1j** instead of **1a**	22%
6	No electrolysis, add Et_3OSbCl_6	n.d.[b]

[a]Determined by ^1H NMR using benzaldehyde as an internal standard. [b]Not detectable.

3-7. Kinetic analysis

3-7-1. Introduction

For quantitative evaluation of electrochemical mediators in a homogeneous system, analytical approaches based on voltammetry, known as S-shape analysis and foot-of-the-wave analysis (FOWA), have been developed by Savéant and Costentin.[23,24] Prerequisite is that the mediator needs to show reversible redox behavior in the absence of a substrate. In the presence of a substrate, the oxidation/reduction current of a meditor increases concomitant with the disappearance of the reverse current.

S-Shape analysis is applicable to an ideal electrocatalytic system. If the reaction is an ideal catalytic reaction, the current reaches a plateau and an S-shaped CV curve is observed, as the reaction reaches a steady state. This curve is independent of the scan rate, and the rate constant of the catalytic reaction and the turnover frequency (TOF) can be calculated based on the plateau current i_{pl}.

On the other hand, S-shape response is not obtained when side reactions such as substrate consumption and catalyst decomposition occur. In such situation, FOWA is a powerful approach that enables the extraction of kinetic information by voltammetry. In FOWA, the analysis is performed for the region of the beginning of the catalysis, thus the effect of side reaction can be ignored.

3-7-2. The procedure of Foot-of the-wave analysis (FOWA)

In an idealistic electrocatalysis, the current (i) and plateau currents (i_{pl}) are described as follows:

$$i = \frac{i_{pl}}{1 + \exp[f(E - E^0_{\mathrm{P/Q}})]} \quad \text{eq. S5}$$

$$i_{pl} = FSC^0_{cat}\sqrt{D_{cat}}\sqrt{k_{cat}C^0_{sub}} \quad \text{eq. S6}$$

where f is F/RT (F: faraday constant, R: gas constant, T: absolute temperature), S is electrode surface area, D_{cat} is diffusion coefficient of catalyst, k_{cat} is a rate constant of the catalytic process, $C_{sub}{}^0$ is a bulk concentration of substrate, respectively.[23]

In a voltammetry under ideal electrocatalytic conditions, S-shaped behavior is obtained and the rate constant is derived from i_{pl}. On the other hand, FOWA derives the rate constant by analyzing the region where the current curve can show the same behavior as in an ideal catalytic system. Specifically, the curve is transformed with the current value as the vertical axis and $(1 + \exp[f(E - E^0_{\mathrm{P/Q}})])^{-1}$ as the horizontal axis, and the ideal region is the area where the potential is close to $E^0_{\mathrm{P/Q}}$ and can be linearly approximated.

The mechanism we will treat here is an ECEC process, and the previous equation cannot be applied as such. For ECEC process, FOWA can be used by approximating the equation for each type of electron transfer steps (E_1, E_2) and chemical reaction steps (C_1, C_2). The following is the catalytic reaction system that will be treated in this report.

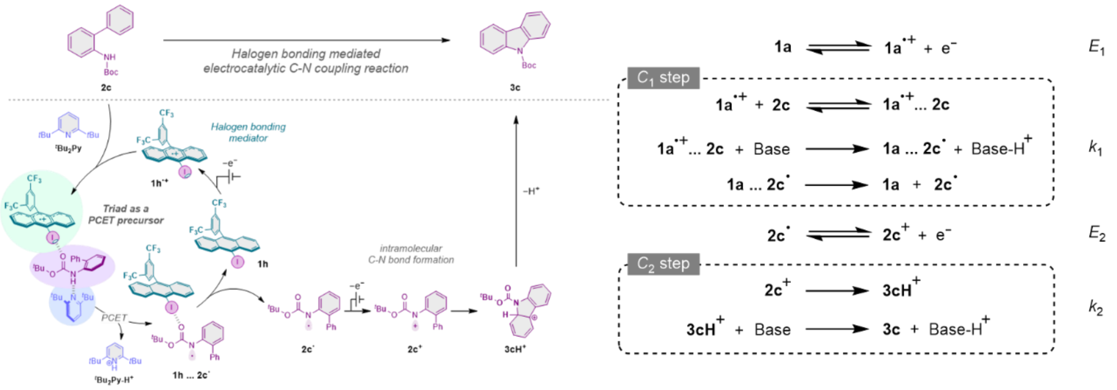

In the present system, the CV voltammograms showed that the E_2 process occurs more easily than the E_1 process, so we defined the currents as follows, based on the report by Savéant and Costantin.[23]

$$i = \frac{2FSC^0_{cat}\sqrt{D_{cat}}}{\frac{1}{\sqrt{k_1 C^0_{sub}}}\left(1+\exp\left[f\left(E-E^0_{\mathrm{P/Q}}\right)\right]\right)+\frac{1}{\sqrt{k_2 C^0_{base}}}} \quad \text{eq S7}$$

In addition, since k_2 is known to be sufficiently faster than k_1 from the verification of kinetic isotope effects (Figure S12), we can approximate $k_2C^0_{base} \gg k_1C^0_{sub}$, and eq S7 can be simplified as follows.

$$i = \frac{2FSC^0_{cat}\sqrt{D_{cat}}\sqrt{k_1 C^0_{sub}}}{\left(1+\exp\left[f\left(E-E^0_{\mathrm{P/Q}}\right)\right]\right)} \quad \text{eq S8}$$

The reversible voltammogram of the catalyst solely is expressed as eq S9, which can be combined with eq S8 to derive eq S10, which does not require a diffusion coefficient.

$$i^0_p = 0.446\, FSC^0_{cat}\sqrt{fvD_{cat}} \quad \text{eq S9}$$

$$\frac{i}{i^0_p} = \frac{4.48\sqrt{\frac{k_1}{fv}C^0_{sub}}}{\left(1+\exp\left[f\left(E-E^0_{\mathrm{P/Q}}\right)\right]\right)} \quad \text{eq S10}$$

This equation is utilized to derive the rate constant by FOWA in this study.

3-7-3. Kinetic analysis of the catalytic reaction using FOWA

All CV analyses for FOWA were conducted under the conditions written in cyclic voltammetry of 9-halo-10-arylanthrancene (**1a-1h**) and *N*-protected aminobiphenyl derivatives for FOWA (3-1-8). The oxidation peak current, i_p^0, and the formal potential of a catalyst, $E_{\mathrm{P/Q}}^0$, were obtained from the voltammogram acquired in the absence of **2a-d**. These values were then used to transform the voltammogram acquired with the substrate into a graph having i/i_p^0 as the vertical axis and $(1 + \exp[f(E - E_{\mathrm{P/Q}}^0)])^{-1}$ as the horizontal axis (Figure S13-29). The slope of the region in this graph within which a high degree of linearity was maintained at potentials above $E_{\mathrm{P/Q}}^0$ was derived by approximating a linear relationship. Using this slope and eq. S6 the rate constant, k_{obs}, was derived.

4. NMR charts

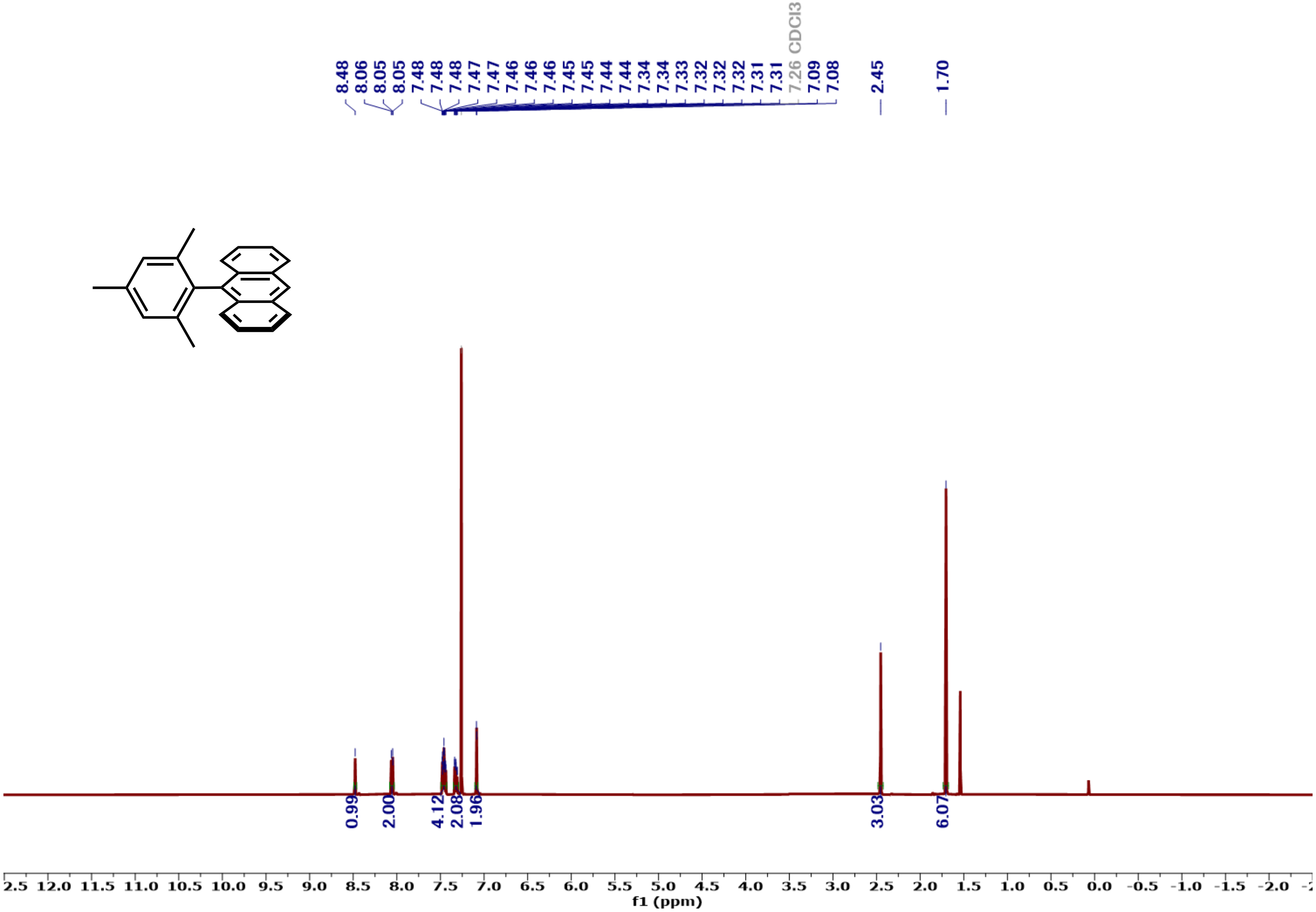

^{1}H NMR spectrum (500 MHz, $CDCl_3$, 25 °C) of **9-mesitylanthracene**.

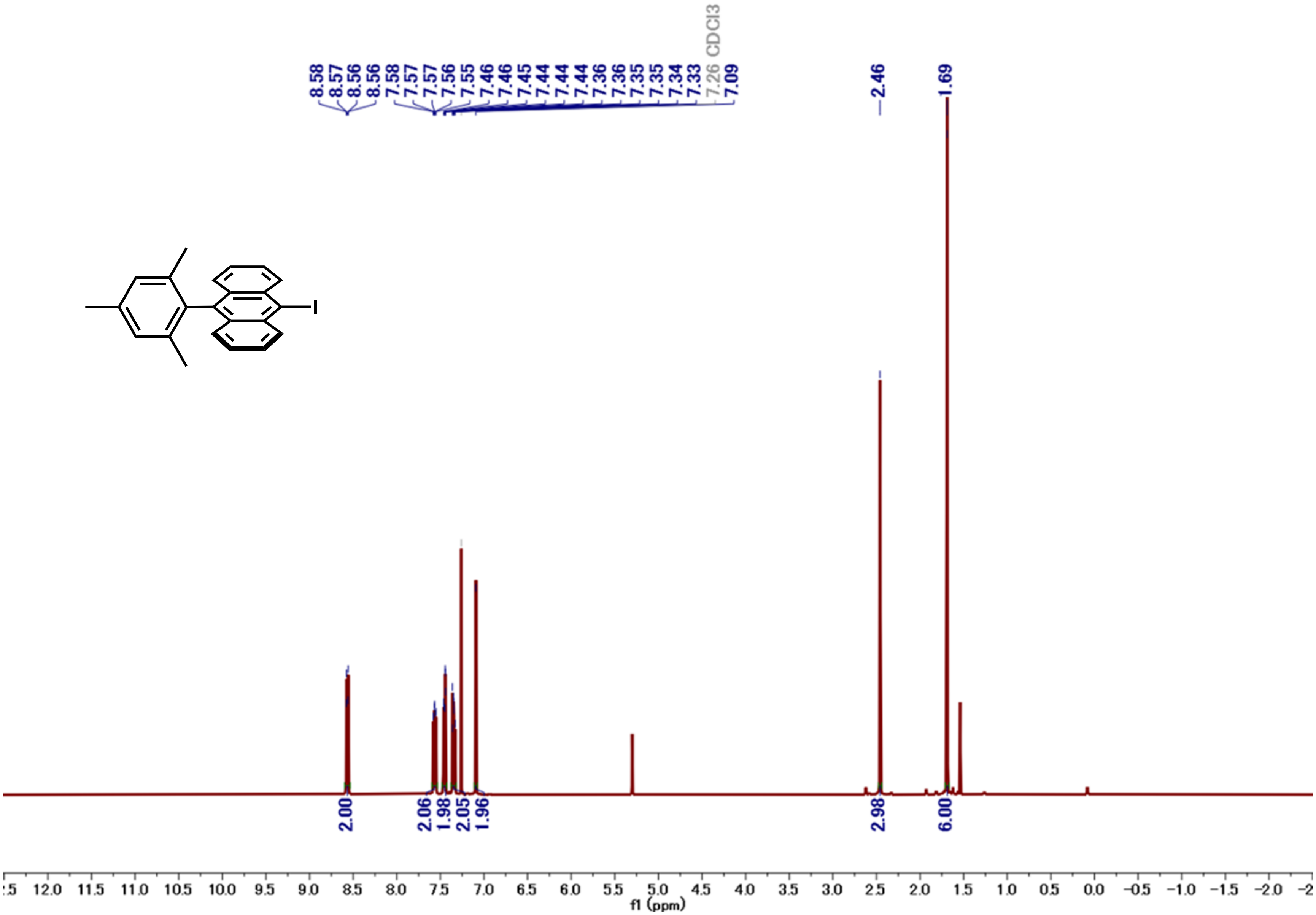

^{1}H NMR spectrum (500 MHz, $CDCl_3$, 25 °C) of **9-iodo-10-mesitylanthracene (1a)**.

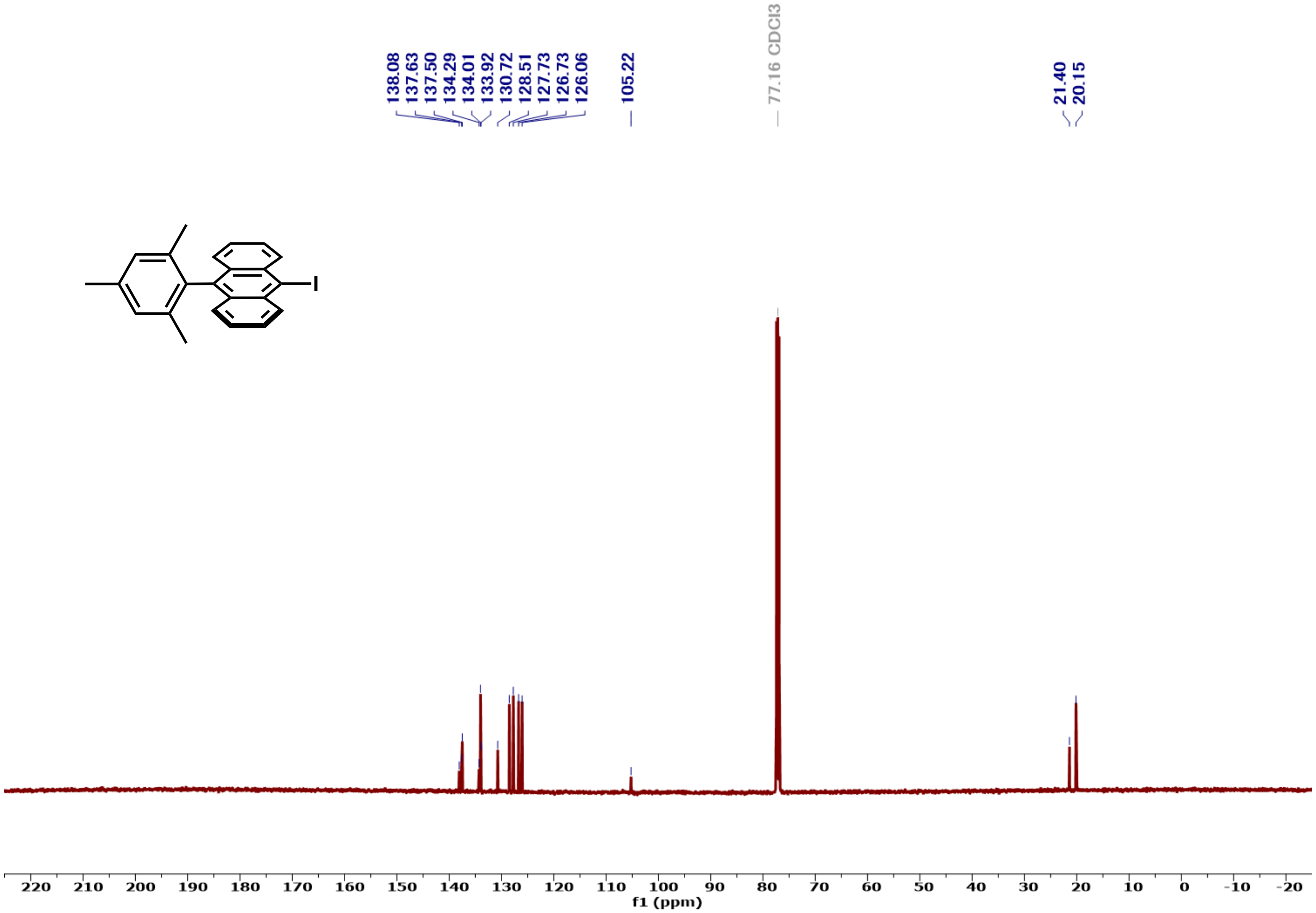

^{13}C NMR spectrum (126 MHz, $CDCl_3$, 25 °C) of **9-iodo-10-mesitylanthracene (1a)**.

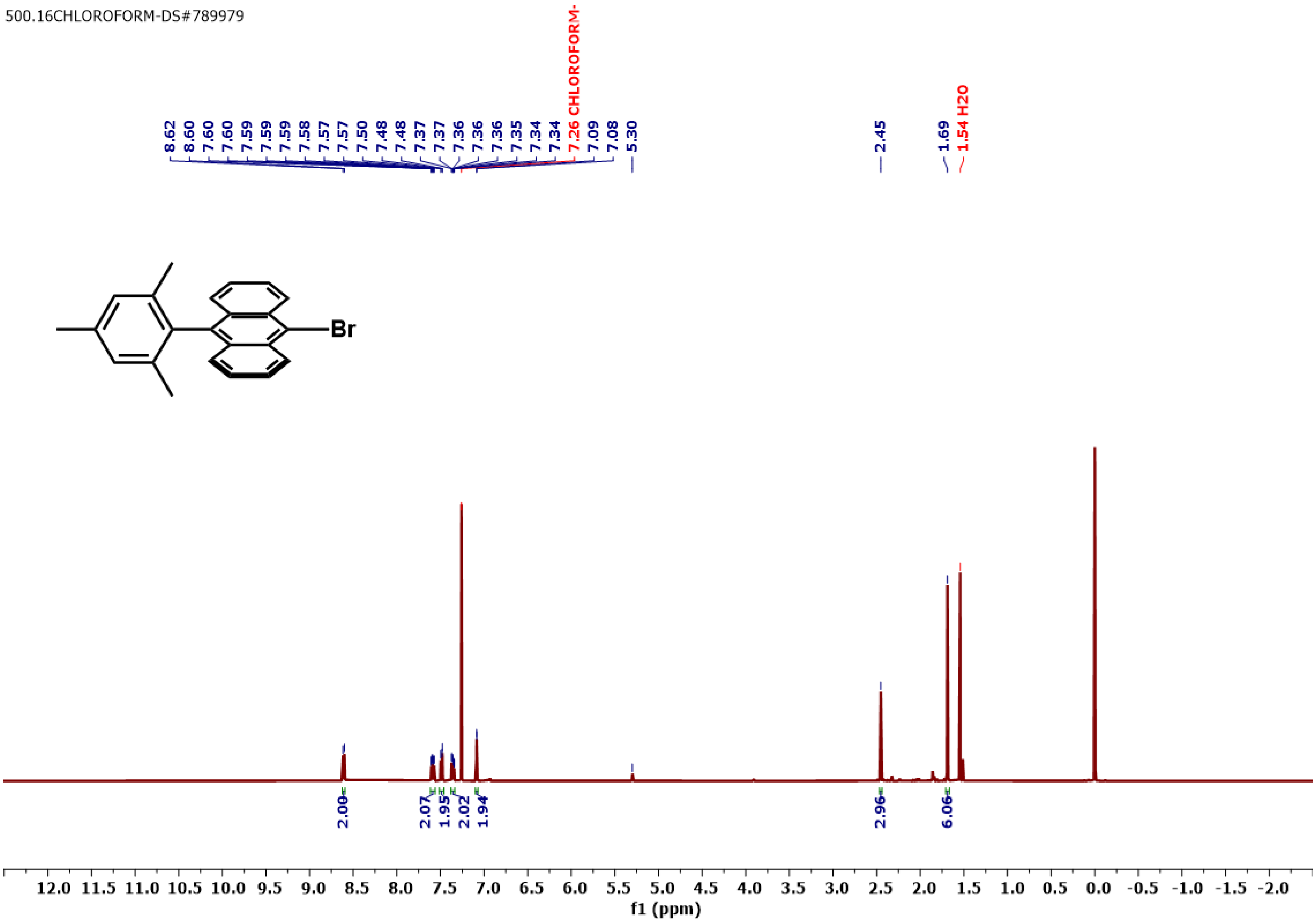

^{1}H NMR spectrum (500 MHz, $CDCl_3$, 25 °C) of **9-bromo-10-mesitylanthracene (1b)**.

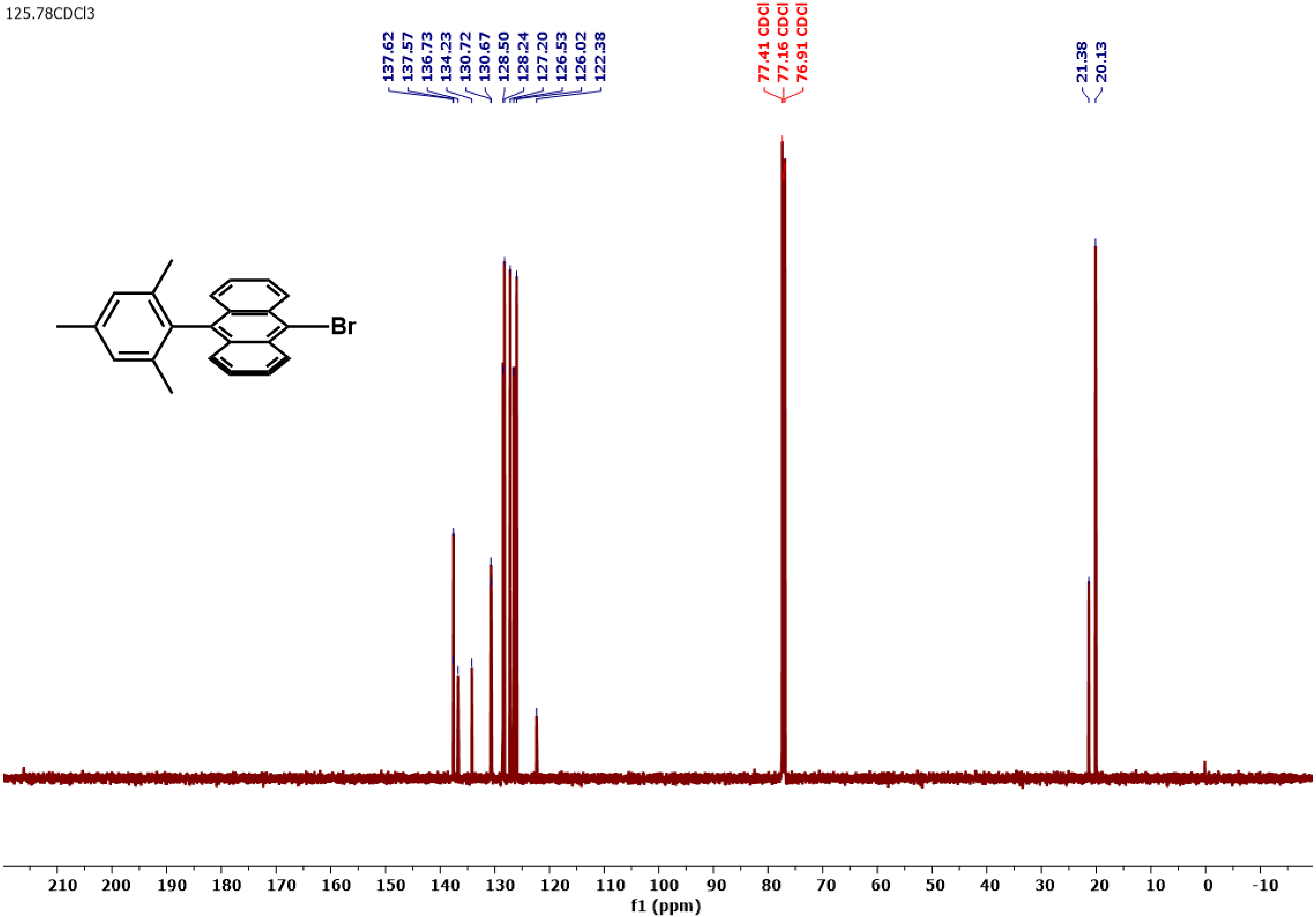

^{13}C NMR spectrum (126 MHz, $CDCl_3$, 25 °C) of **9-bromo-10-mesitylanthracene (1b)**.

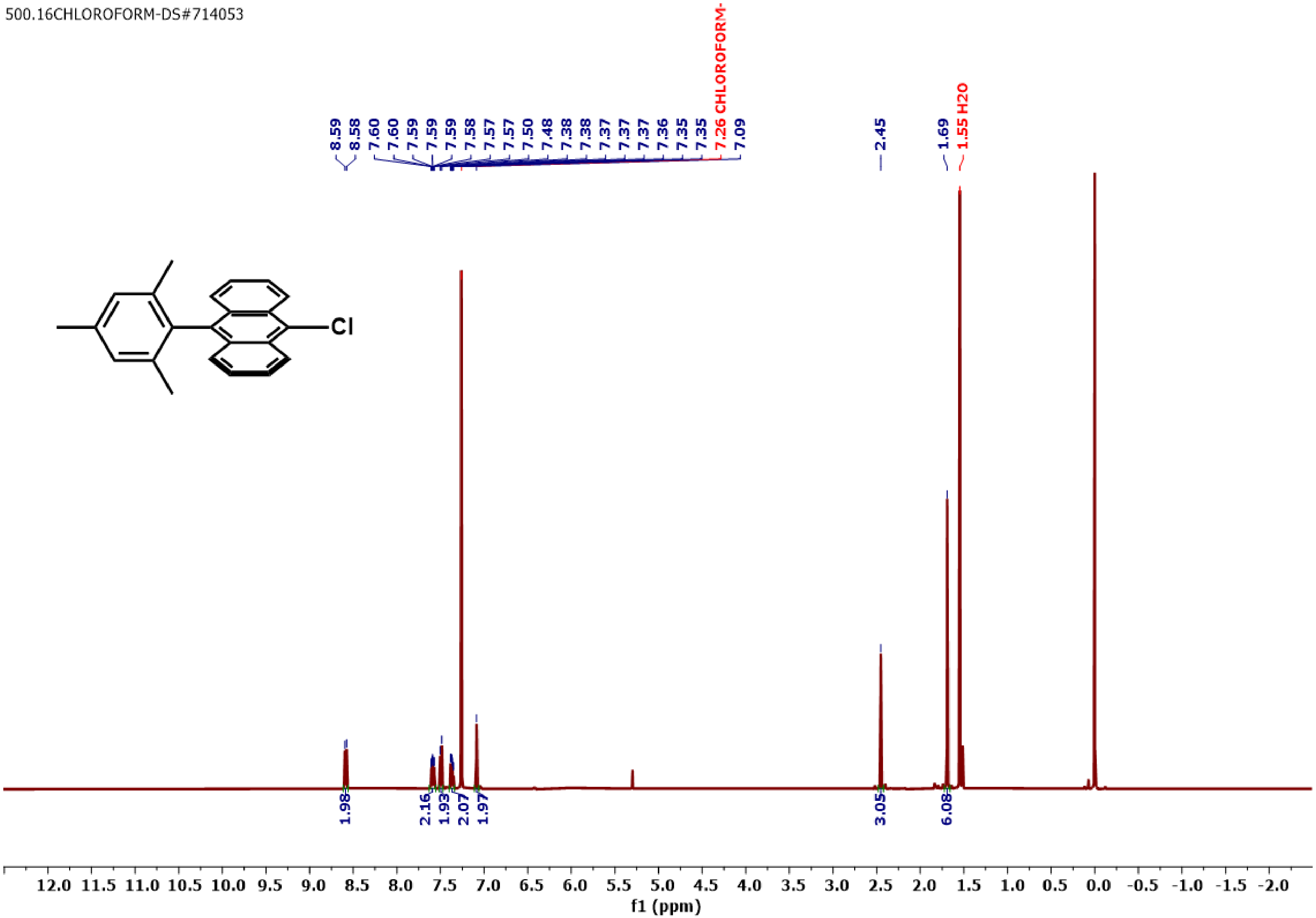

^{1}H NMR spectrum (500 MHz, $CDCl_3$, 25 °C) of **9-chloro-10-mesitylanthracene (1c)**.

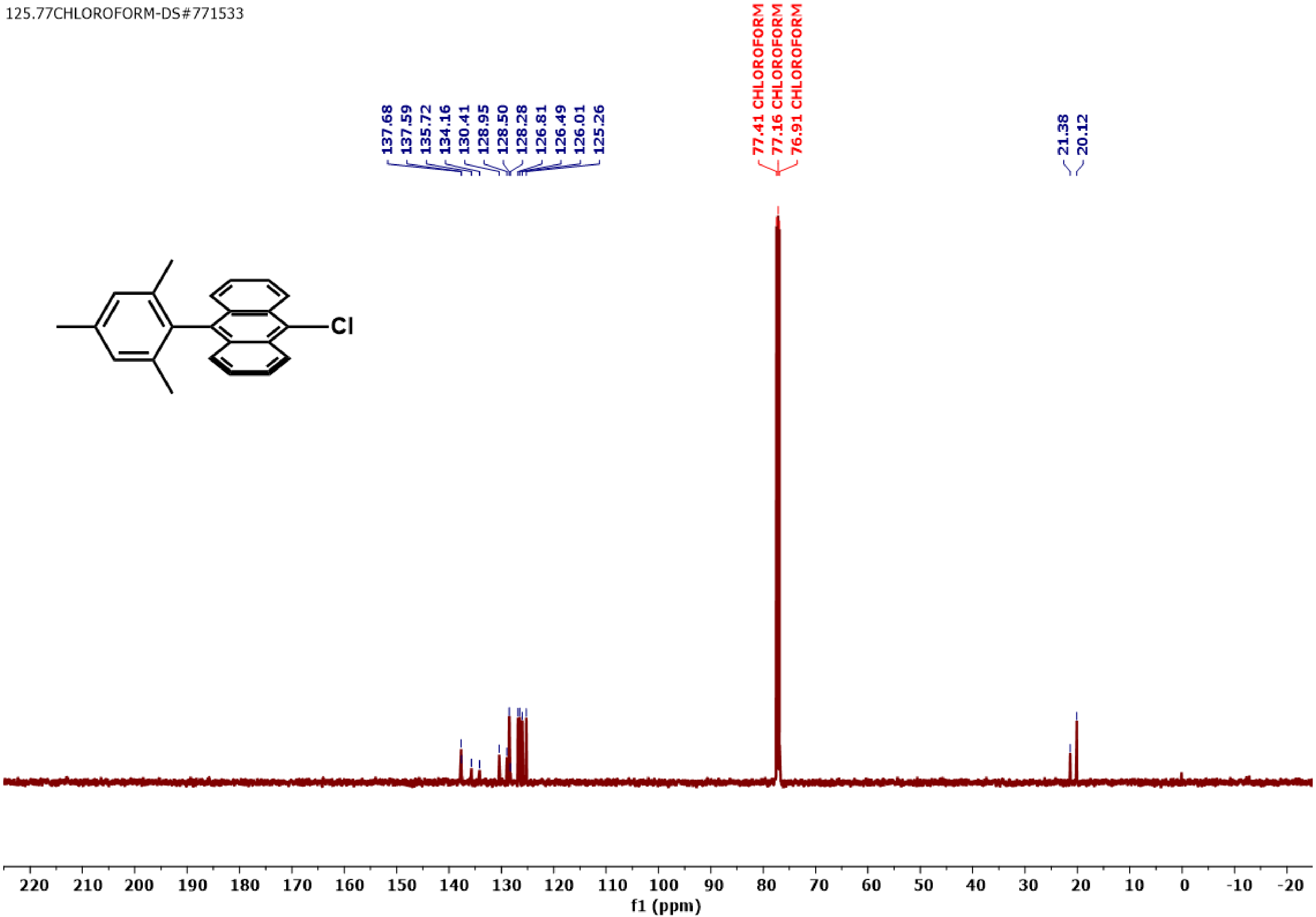

^{13}C NMR spectrum (126 MHz, $CDCl_3$, 25 °C) of **9-chloro-10-mesitylanthracene (1c)**.

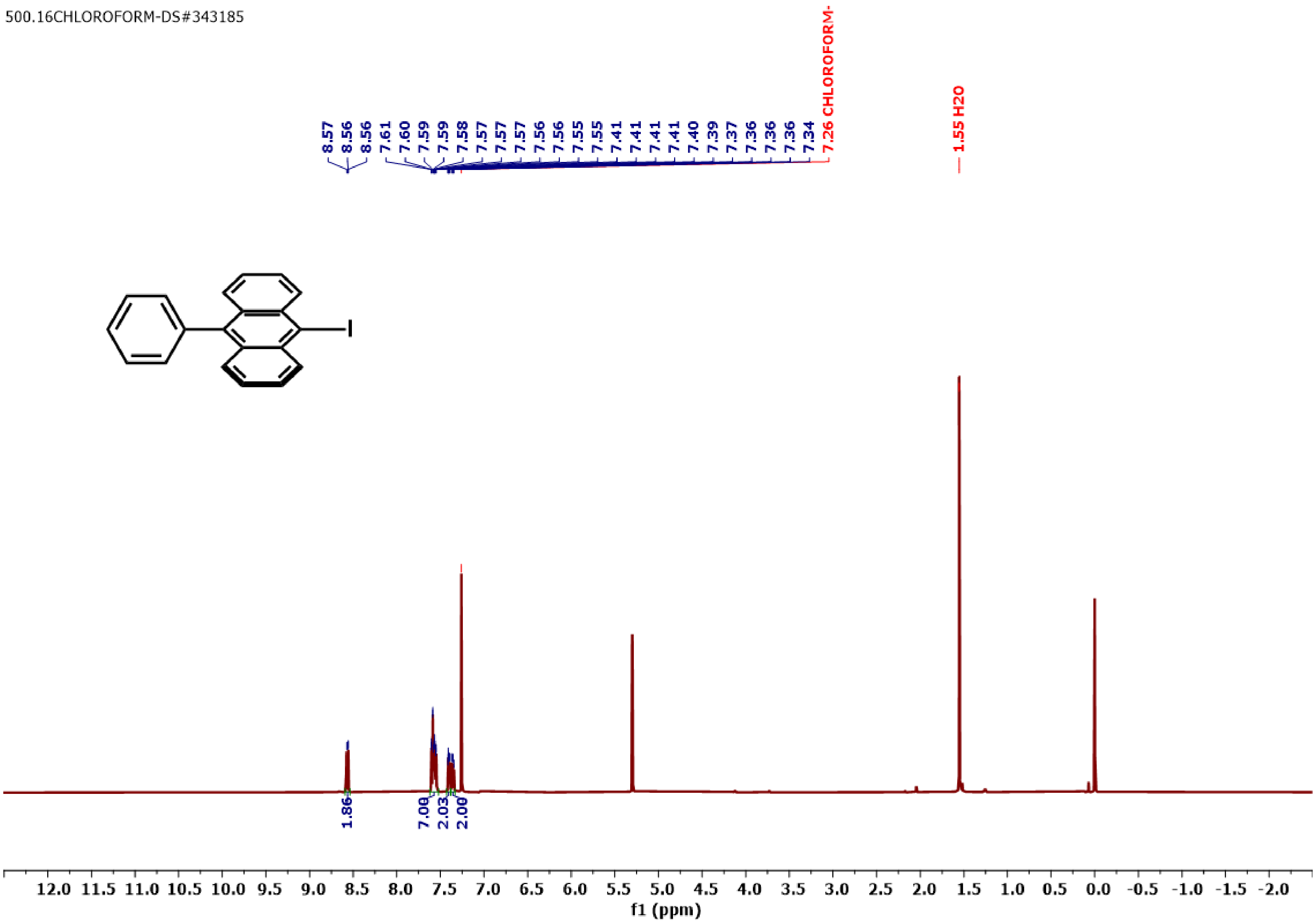

^{1}H NMR spectrum (500 MHz, $CDCl_3$, 25 °C) of **9-iodo-10-phenylanthracene (1d)**.

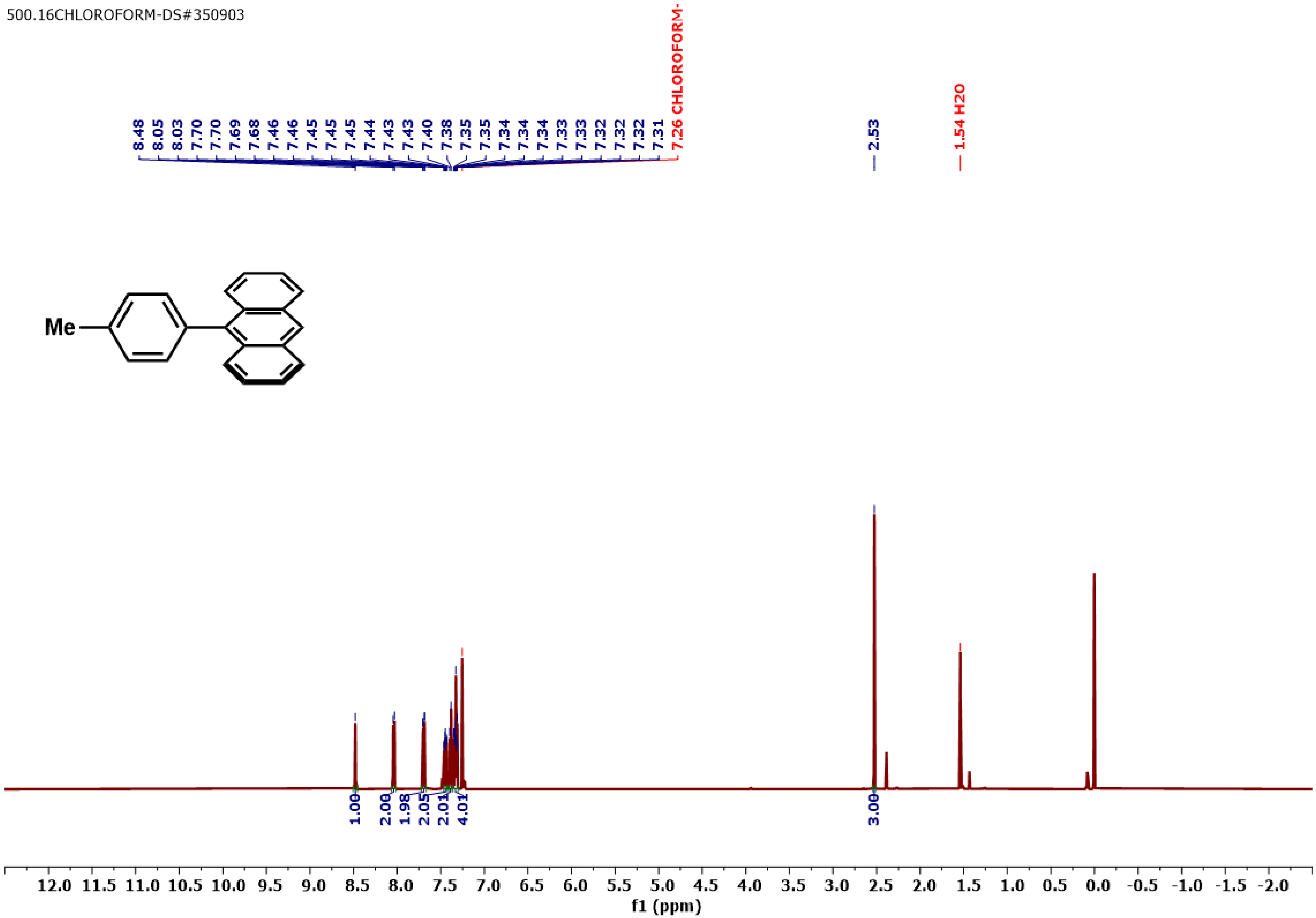

^{1}H NMR spectrum (500 MHz, $CDCl_3$, 25 °C) of **9-(*p*-tolyl)anthracene.**

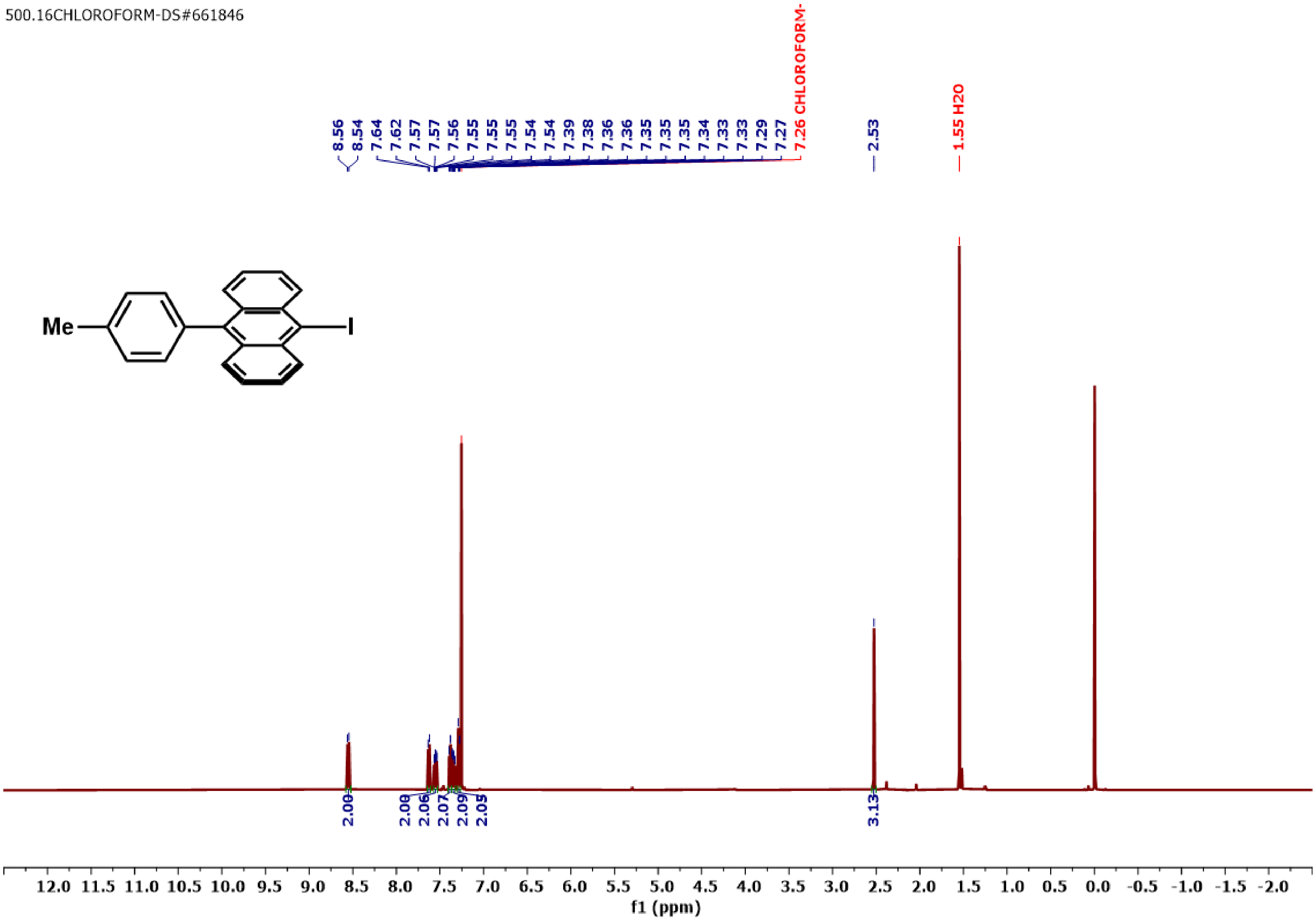

^{1}H NMR spectrum (500 MHz, $CDCl_3$, 25 °C) of **9-iodo-10-(*p*-tolyl)anthracene (1e).**

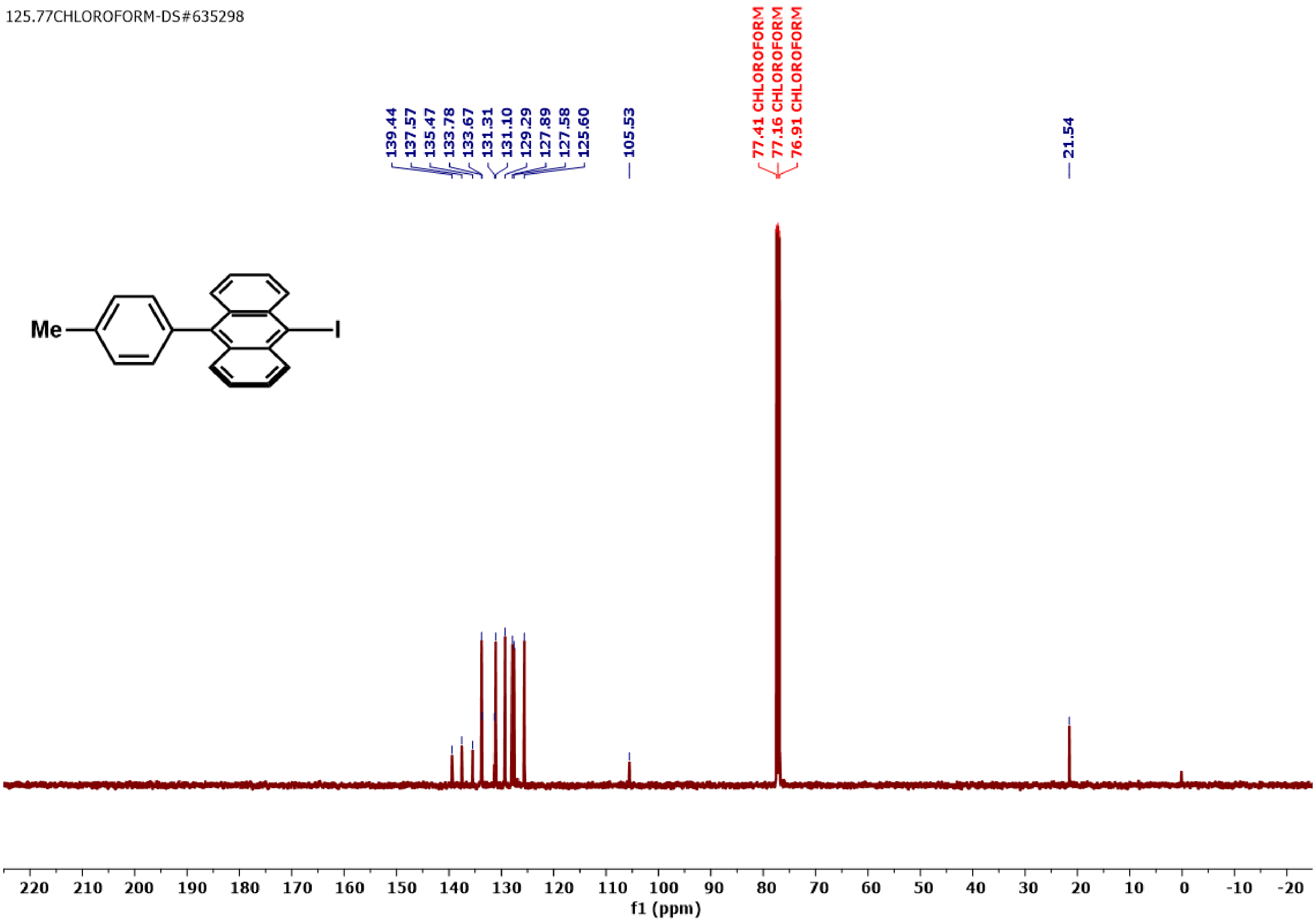

^{13}C NMR spectrum (126 MHz, $CDCl_3$, 25 °C) of **9-iodo-10-(*p*-tolyl)anthracene (1e)**.

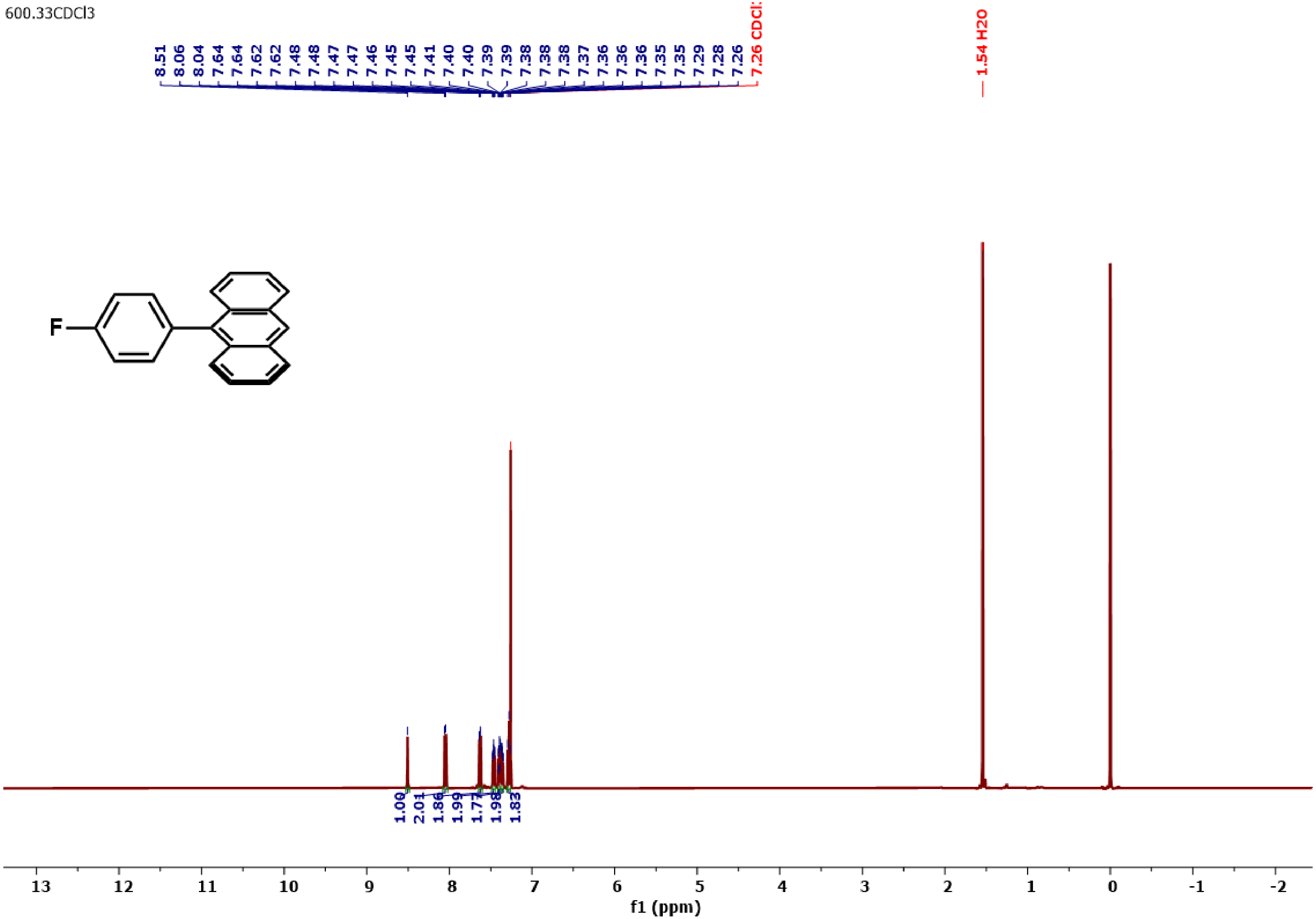

^{1}H NMR spectrum (600 MHz, $CDCl_3$, 25 °C) of **9-(4-fluorophenyl)anthracene.**

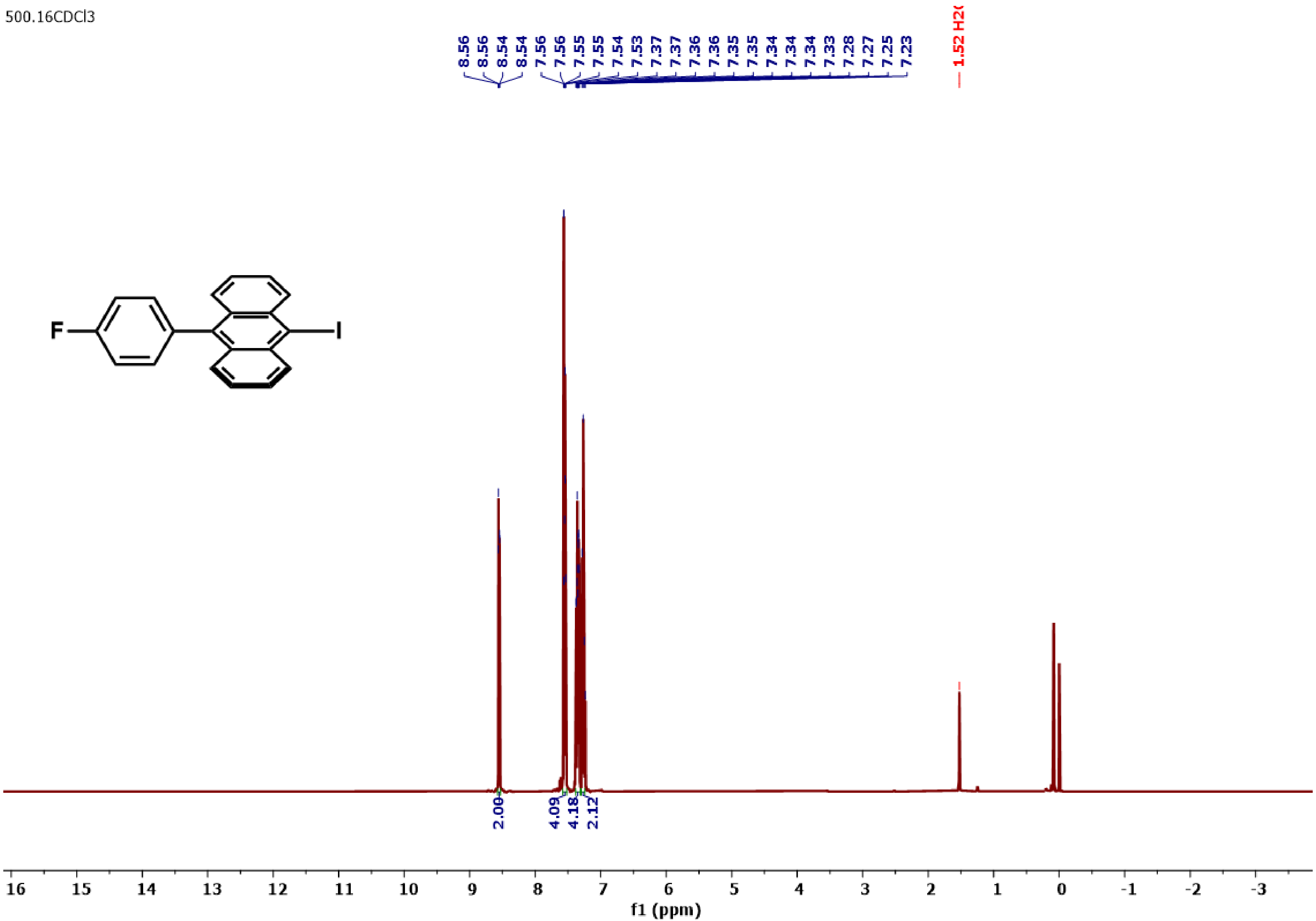

^{1}H NMR spectrum (500 MHz, $CDCl_3$, 25 °C) of **9-(4-fluorophenyl)-10-iodoanthracene (1f).**

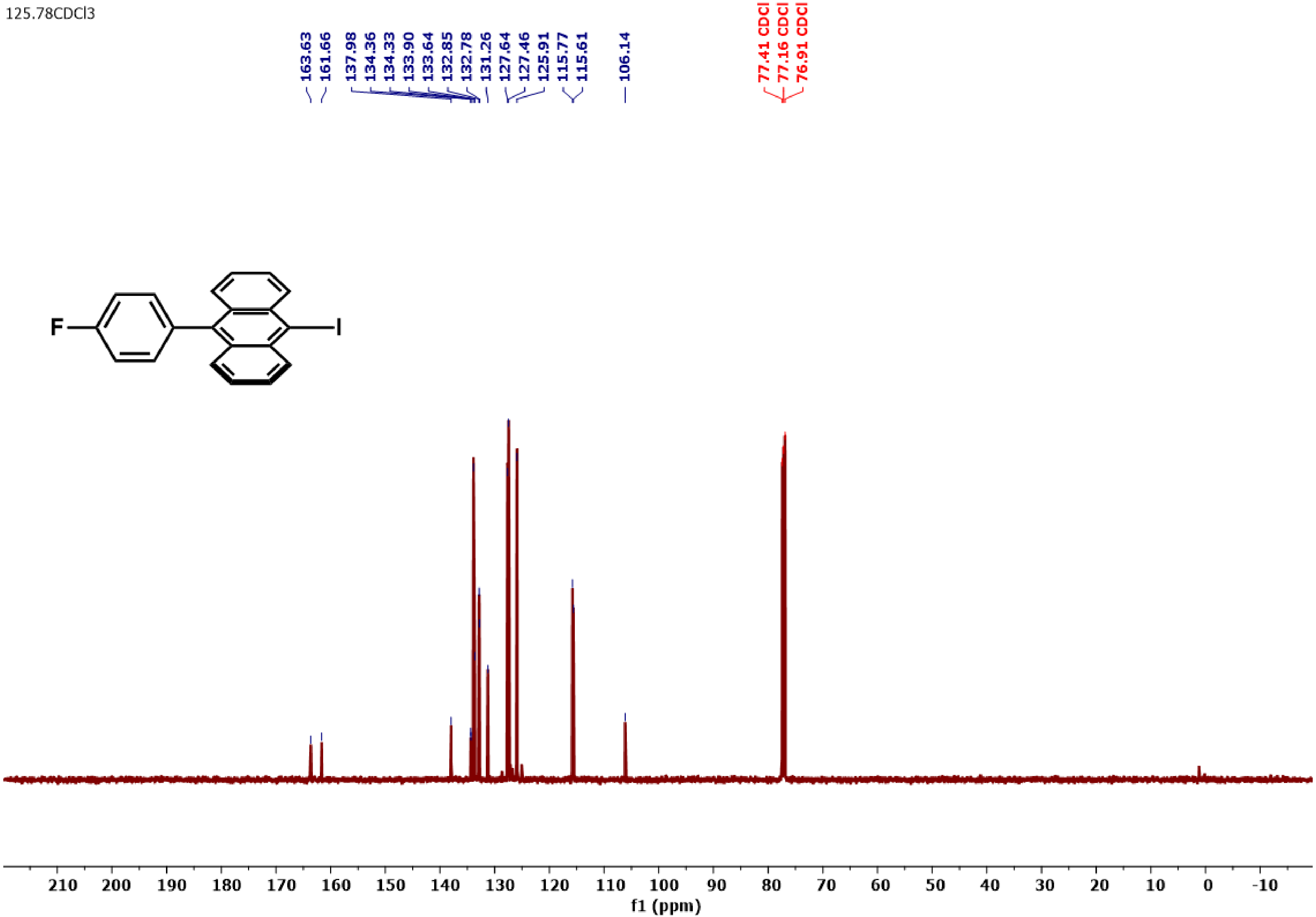

^{13}C NMR spectrum (126 MHz, $CDCl_3$, 25 °C) of **9-(4-fluorophenyl)-10-iodoanthracene (1f)**.

470.62CHLOROFORM-DS#681852

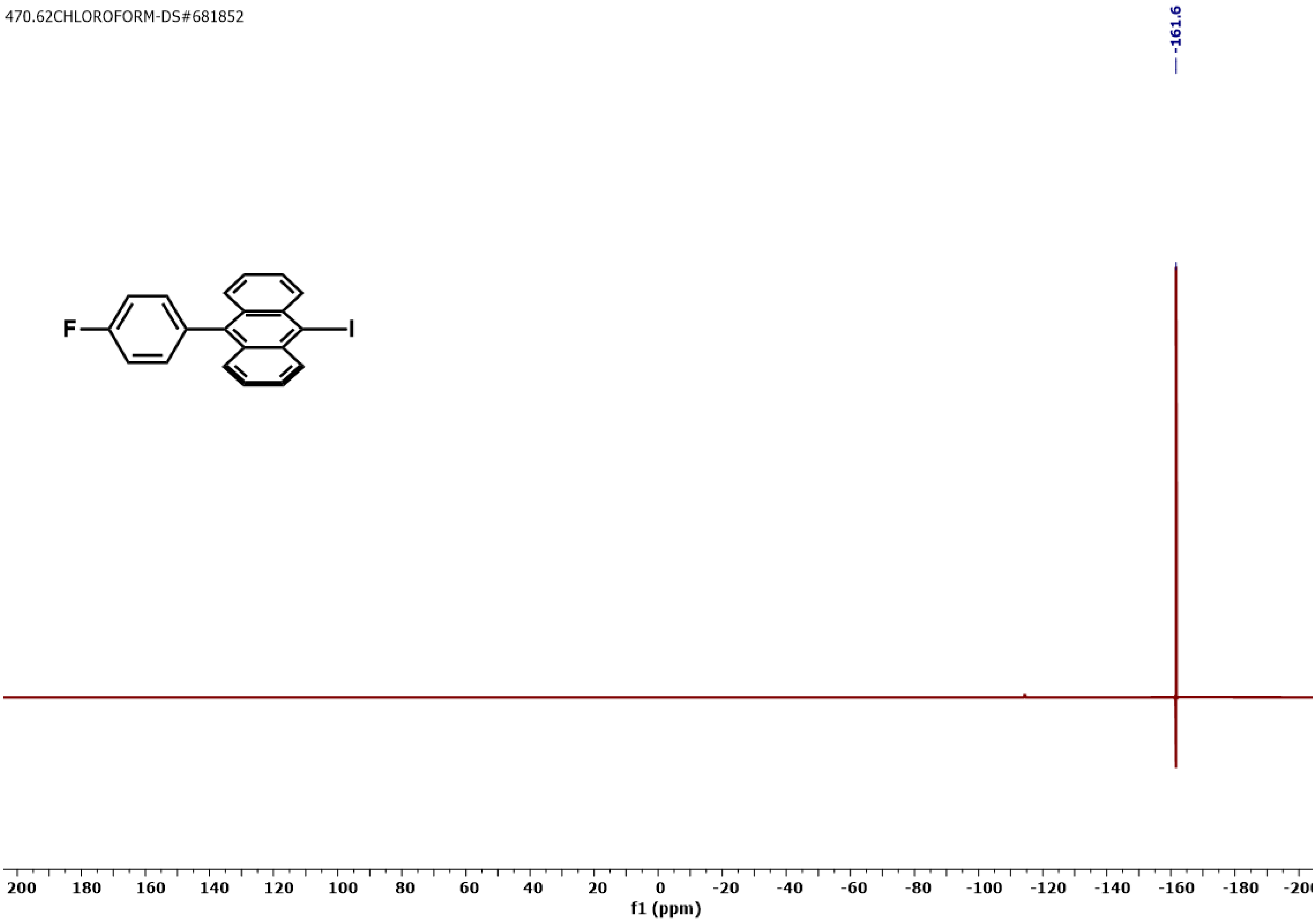

^{19}F NMR spectrum (471 MHz, $CDCl_3$, 25 °C) of **9-(4-fluorophenyl)-10-iodoanthracene (1f)**.

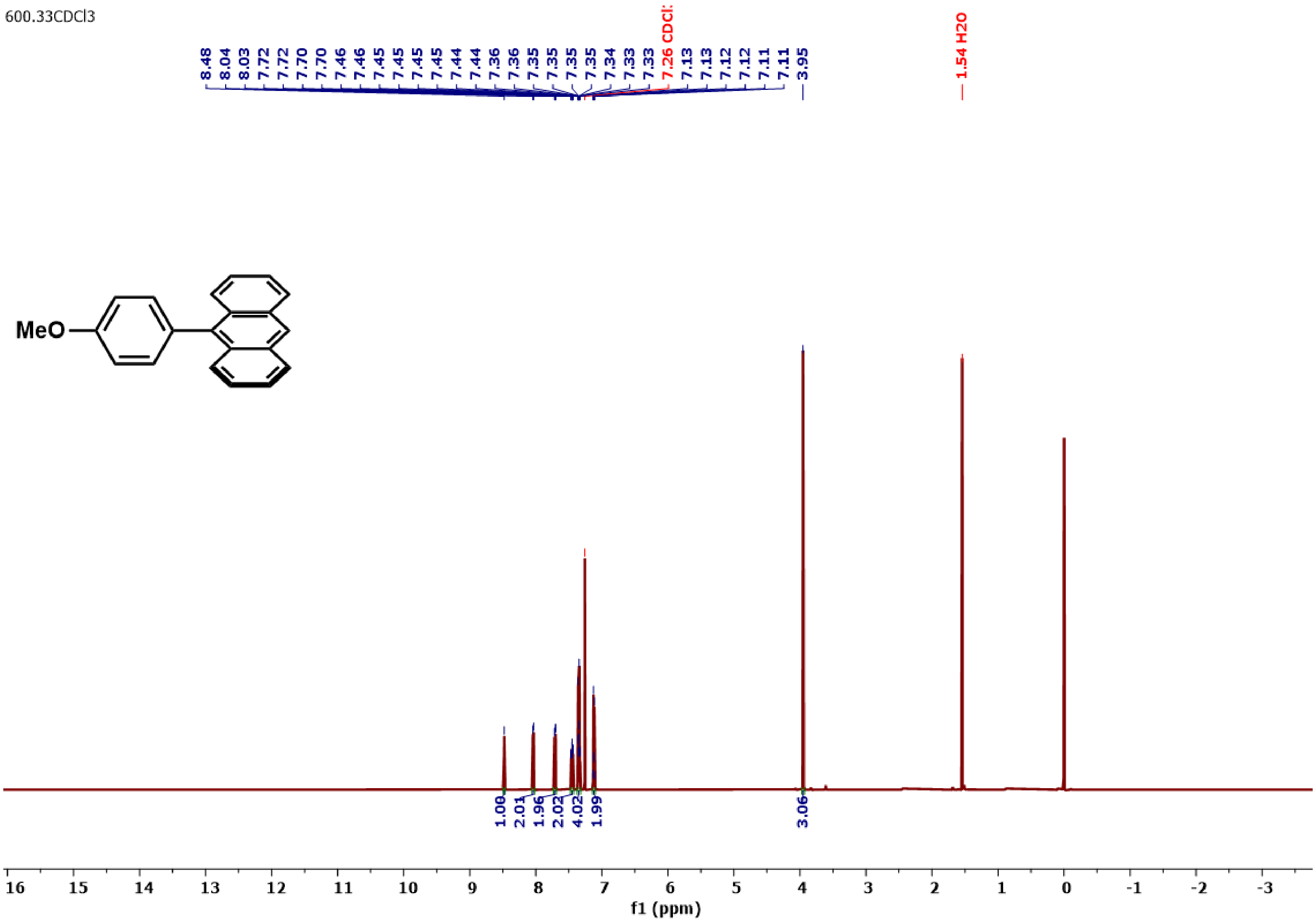

^{1}H NMR spectrum (600 MHz, $CDCl_3$, 25 °C) of **9-(methoxyphenyl)anthracene.**

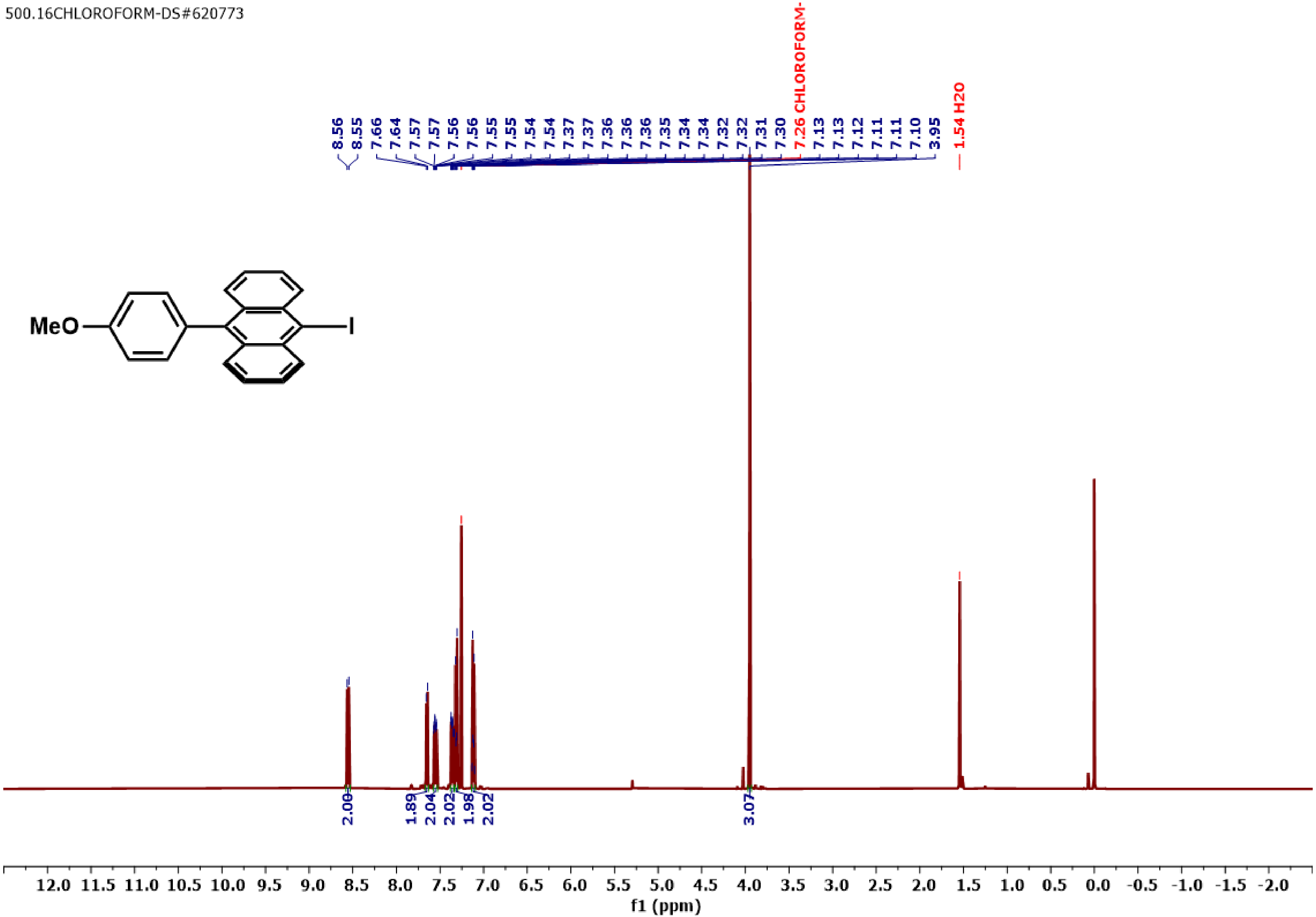

^{1}H NMR spectrum (500 MHz, $CDCl_3$, 25 °C) of **9-iodo-10-(methoxyphenyl)anthracene (1g).**

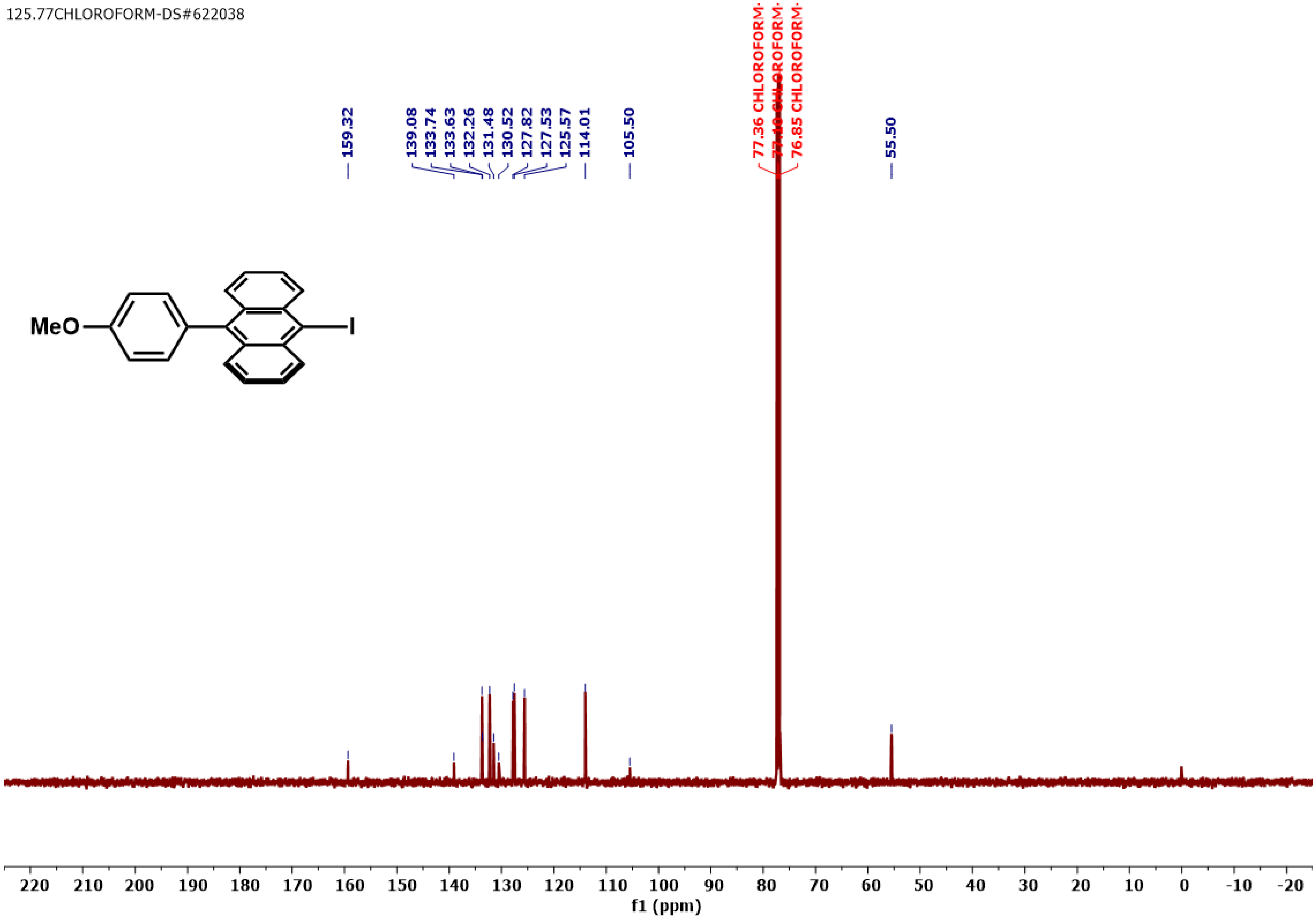

^{13}C NMR spectrum (126 MHz, $CDCl_3$, 25 °C) of **9-iodo-10-(methoxyphenyl)anthracene (1g)**.

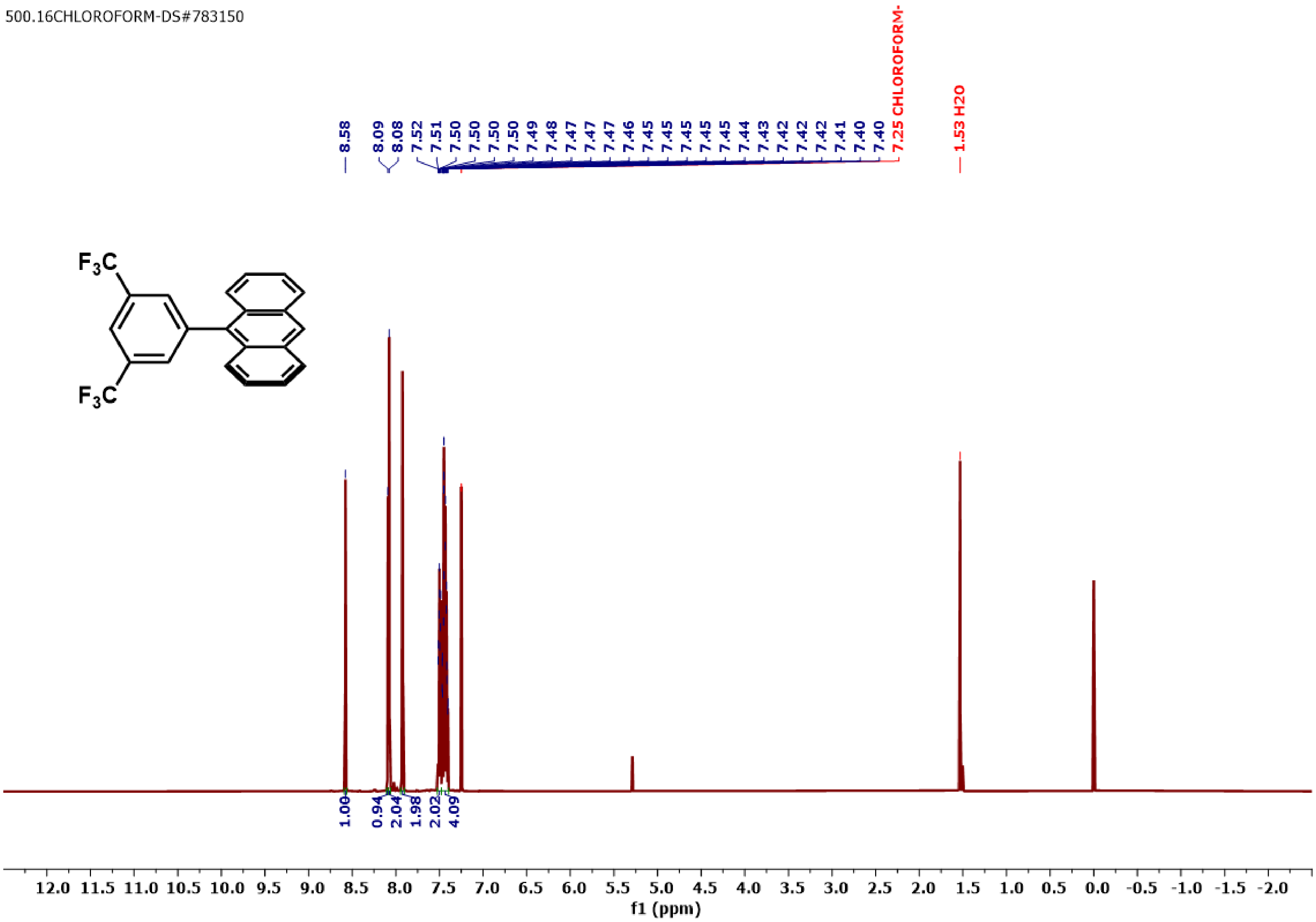

^{1}H NMR spectrum (500 MHz, $CDCl_3$, 25 °C) of **9-(3,5-bis(trifluoromethyl)phenyl)anthracene.**

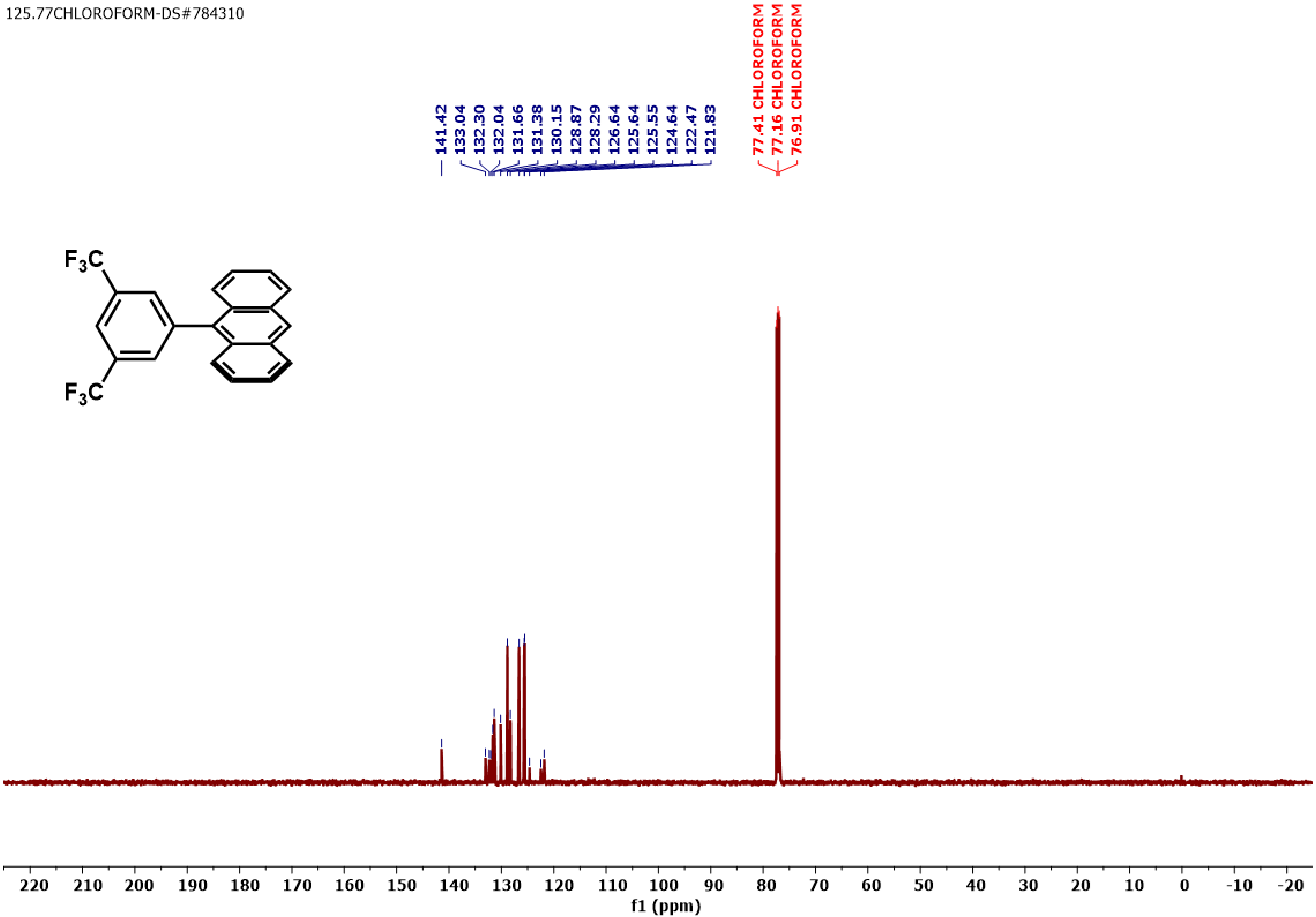

^{13}C NMR spectrum (126 MHz, $CDCl_3$, 25 °C) of **9-(3,5-bis(trifluoromethyl)phenyl)anthracene**.

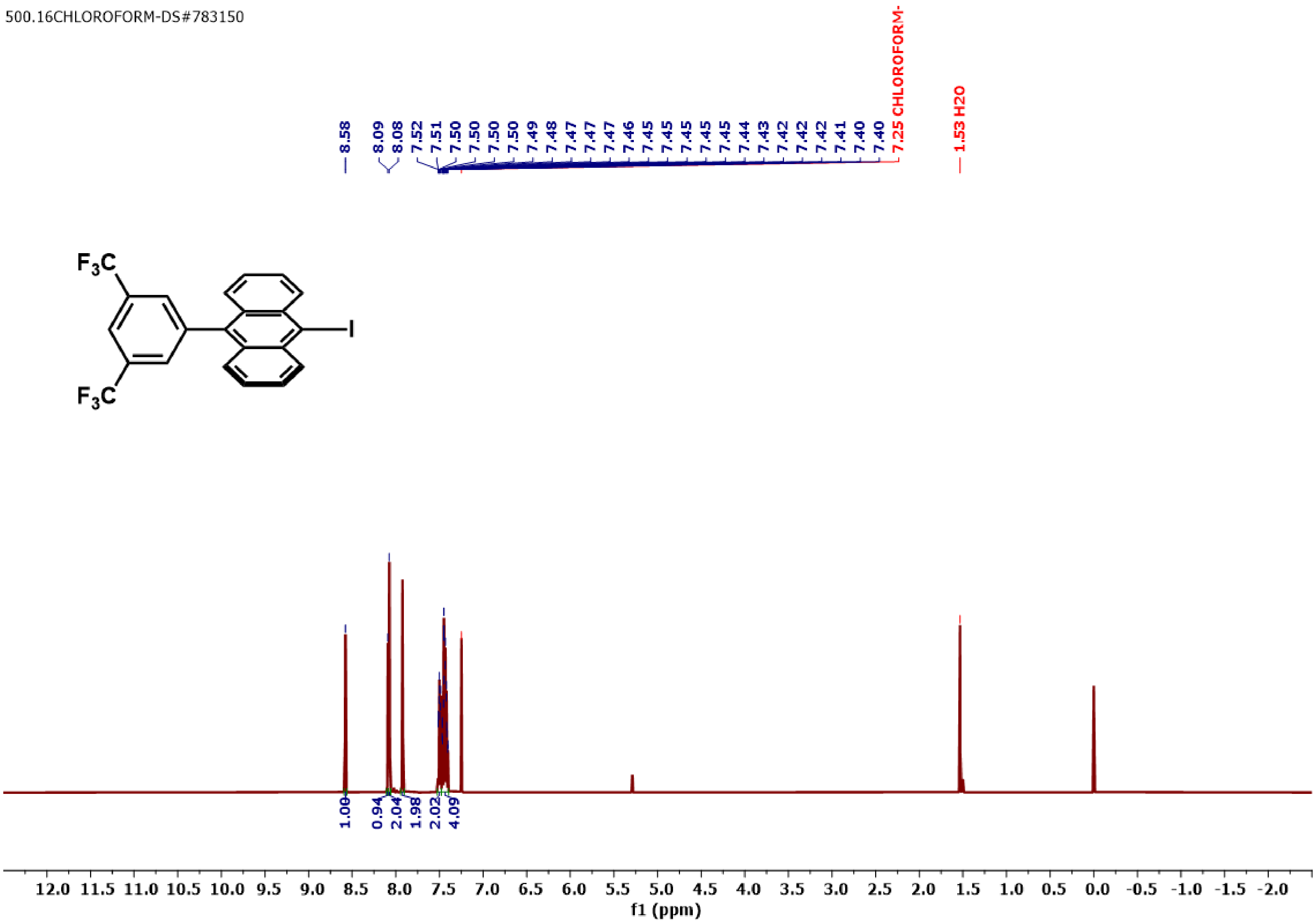

^{1}H NMR spectrum (500 MHz, $CDCl_3$, 25 °C) of **9-(3,5-bis(trifluoromethyl)phenyl)-10-iodoanthracene (1h).**

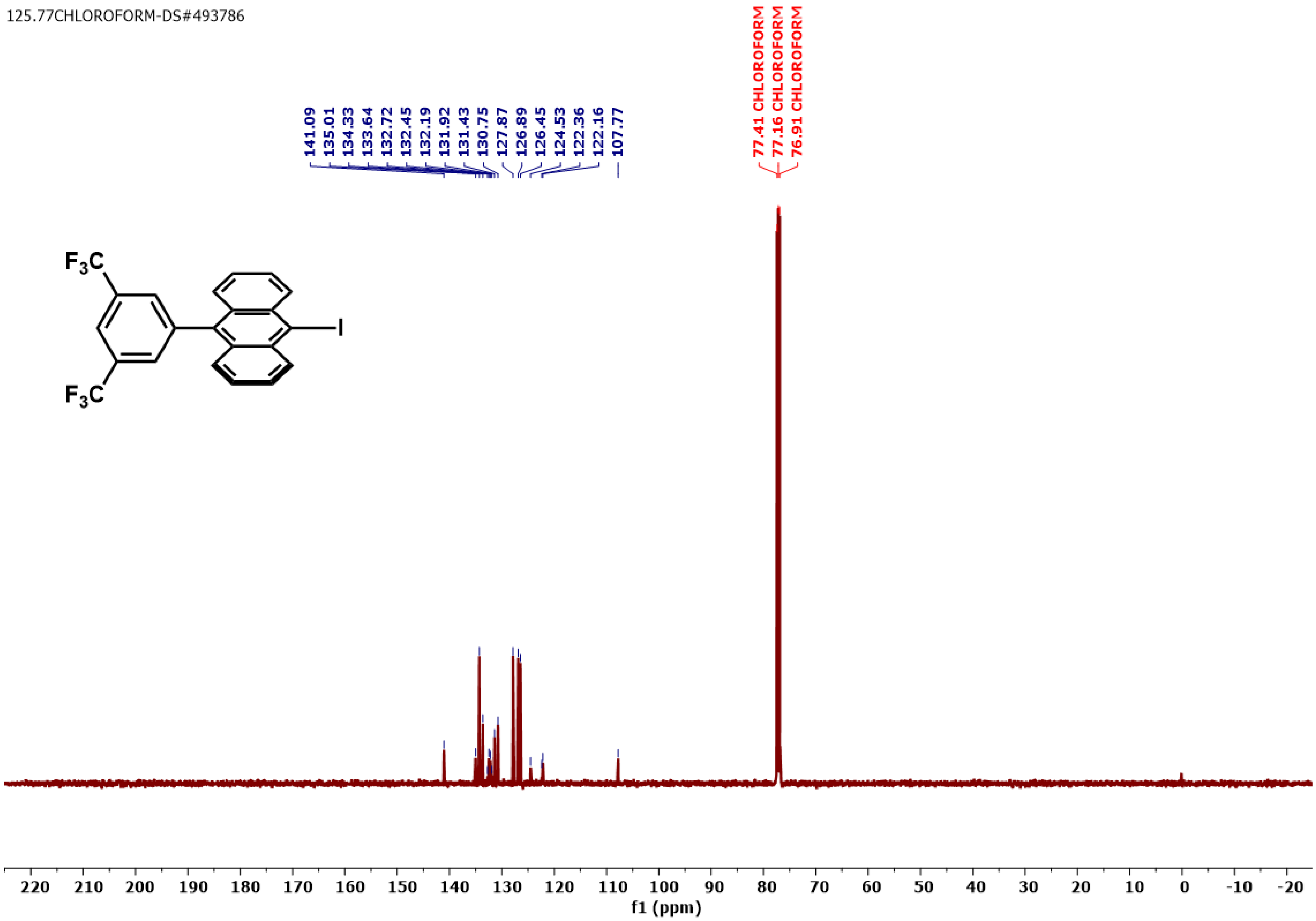

^{13}C NMR spectrum (126 MHz, $CDCl_3$, 25 °C) of **9-(3,5-bis(trifluoromethyl)phenyl)-10-iodoanthracene (1h)**.

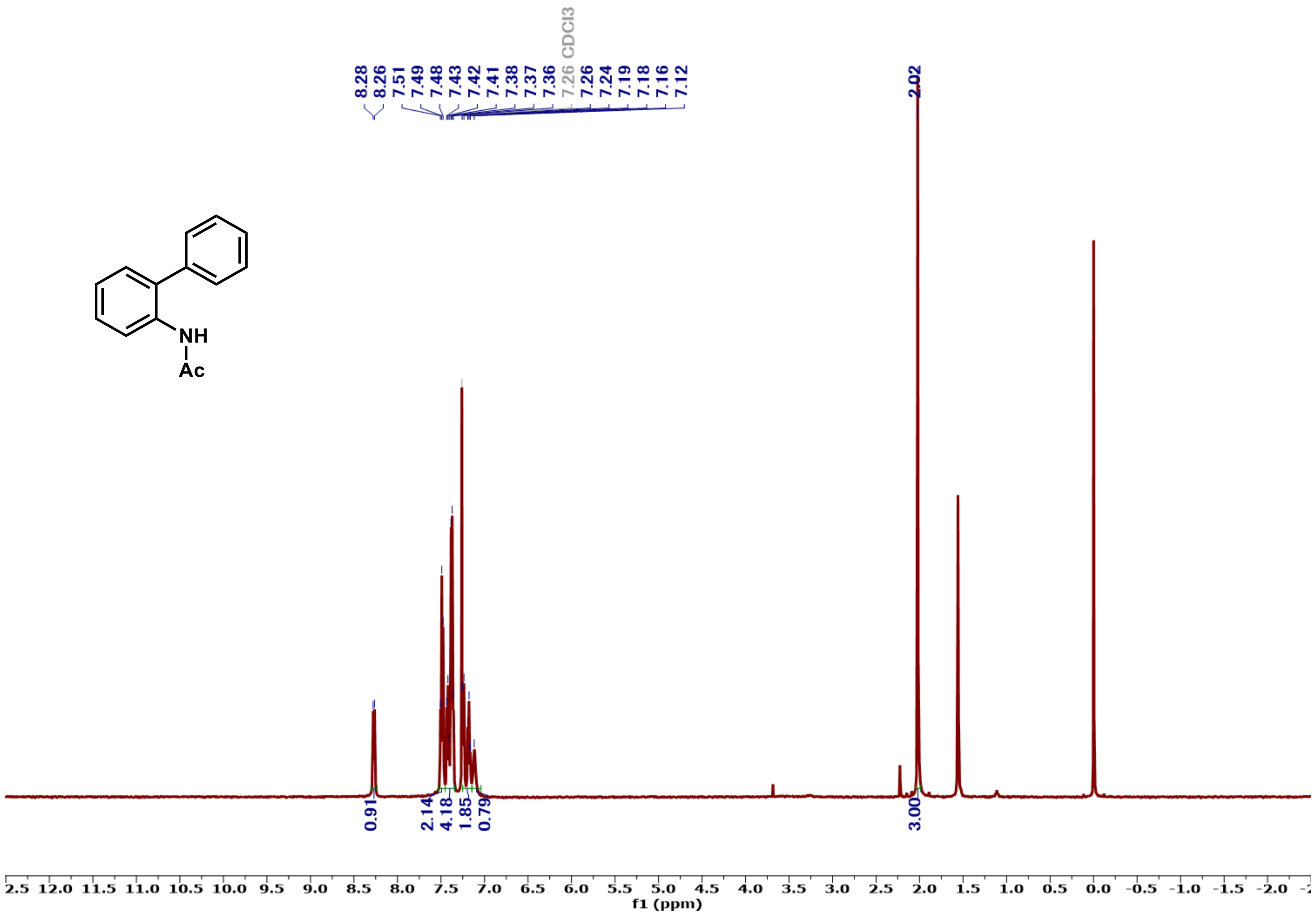

^{1}H NMR spectrum (500 MHz, $CDCl_3$, 25 °C) of **N-([1,1'-biphenyl]-2-yl)acetamide (2a)**.

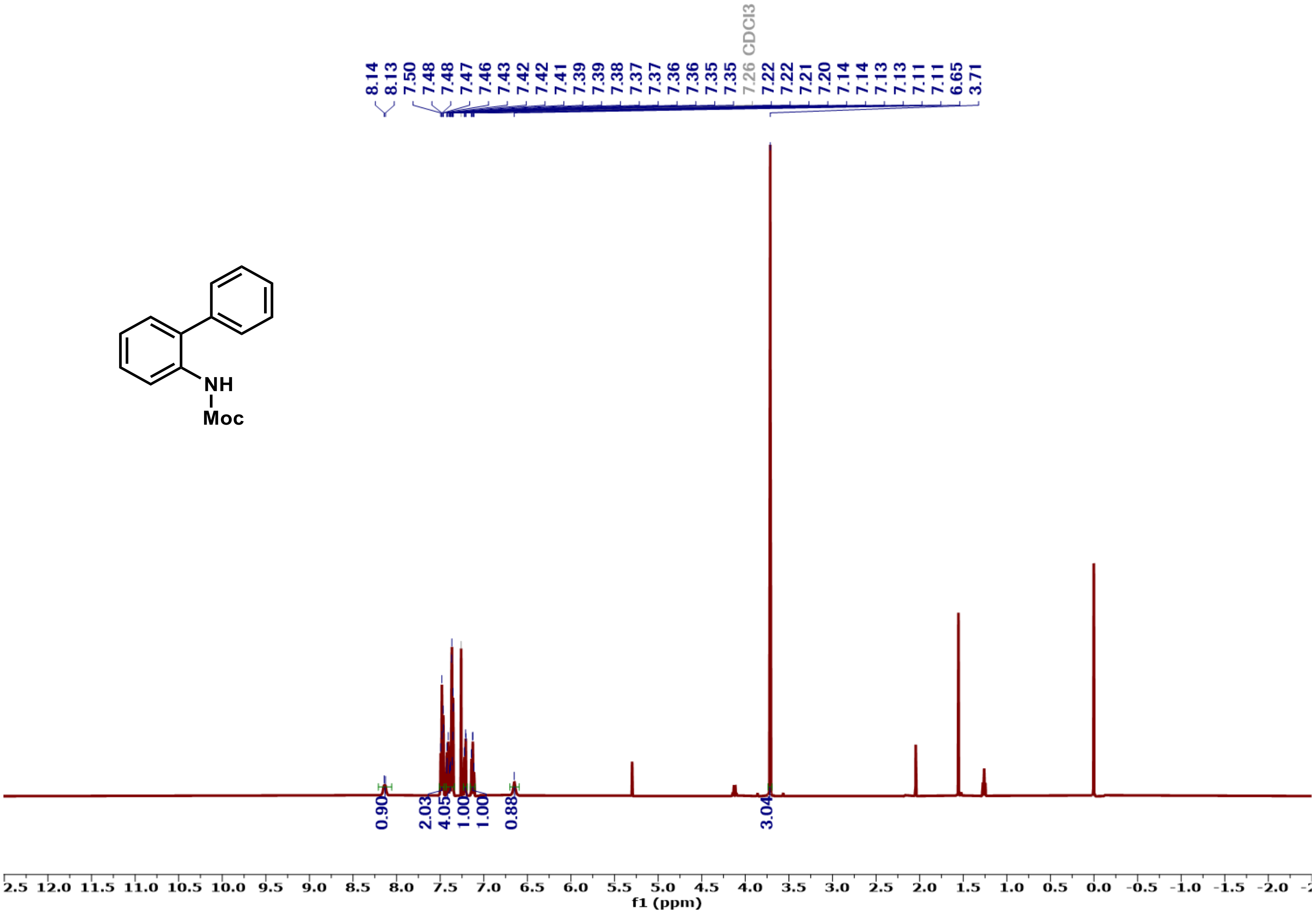

^{1}H NMR spectrum (500 MHz, $CDCl_3$, 25 °C) of **methyl [1,1'-biphenyl]-2-ylcarbamate (2b)**.

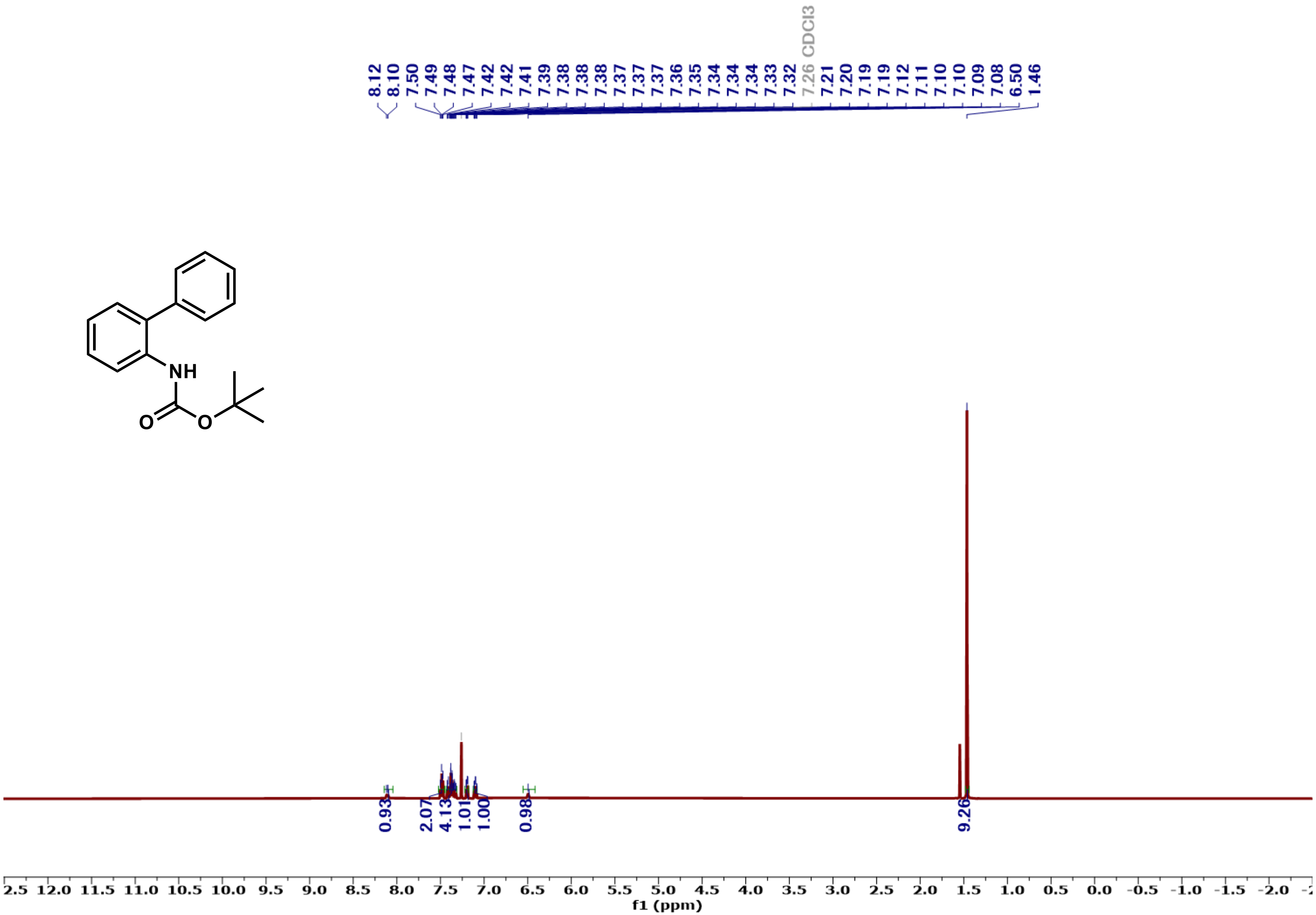

^{1}H NMR spectrum (500 MHz, $CDCl_3$, 25 °C) of ***tert*-butyl [1,1'-biphenyl]-2-ylcarbamate (2c)**.

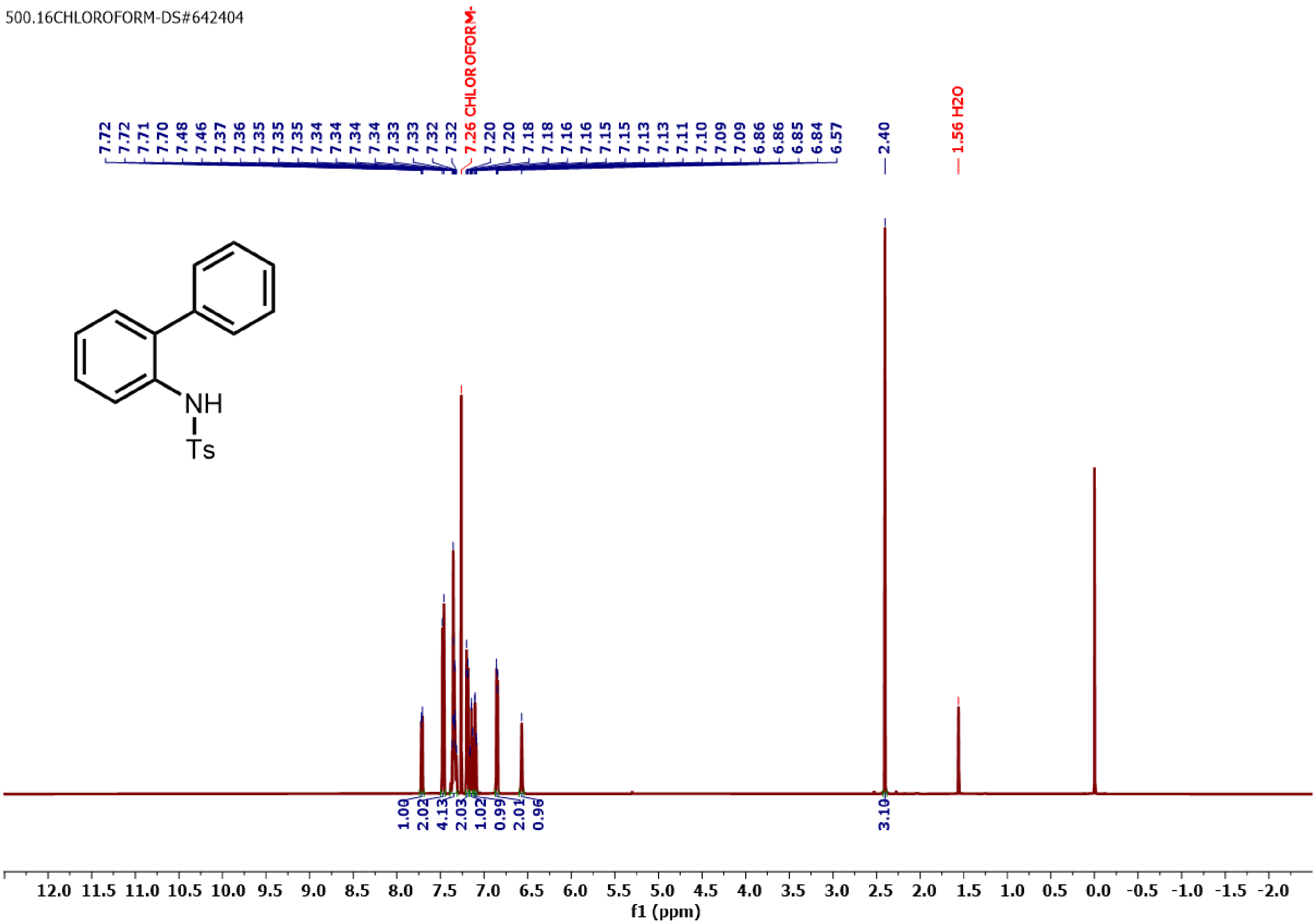

^{1}H NMR spectrum (500 MHz, $CDCl_3$, 25 °C) of ***N*-([1,1'-biphenyl]-2-yl)-4-methylbenzenesulfonamide (2d)**.

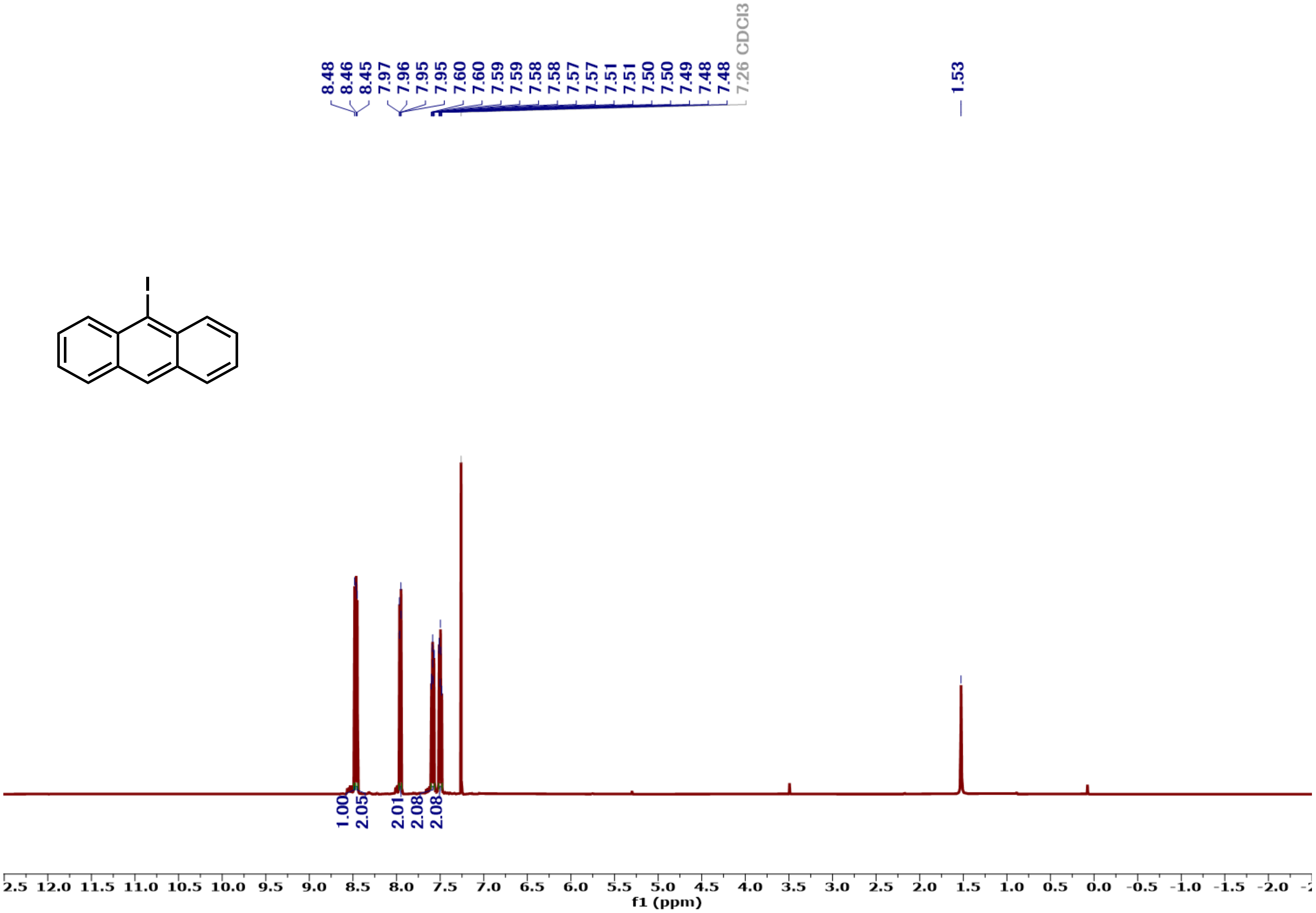

^{1}H NMR spectrum (500 MHz, $CDCl_3$, 25 °C) of **9-iodoanthracene**.

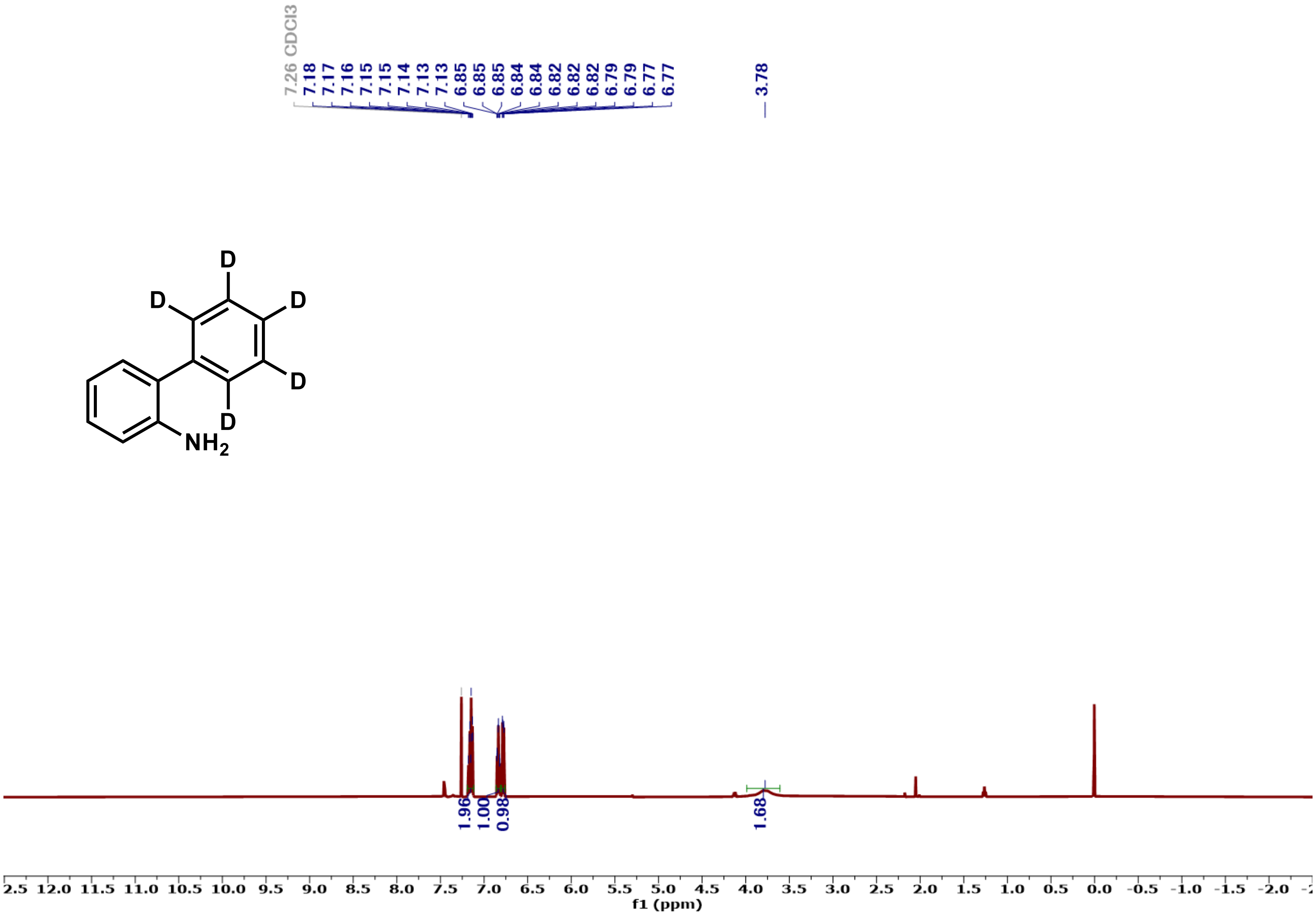

^{1}H NMR spectrum (500 MHz, $CDCl_3$, 25 °C) of **[1,1'-biphenyl]-2',3',4',5',6'-d5-2-amine**.

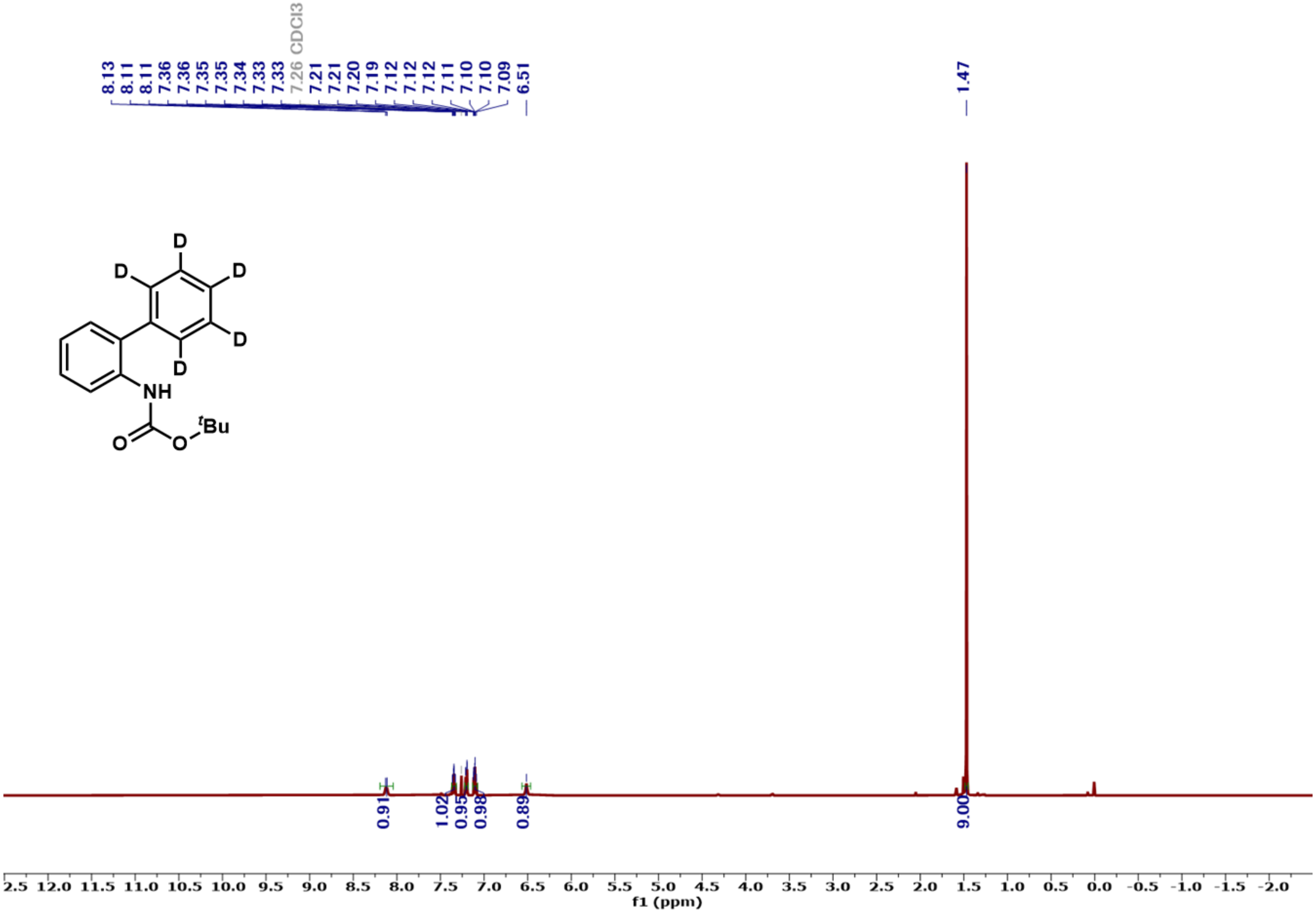

^{1}H NMR spectrum (500 MHz, $CDCl_3$, 25 °C) of ***tert*-butyl ([1,1'-biphenyl]-2-yl-2',3',4',5',6'-d5)carbamate (2-d$_5$)**.

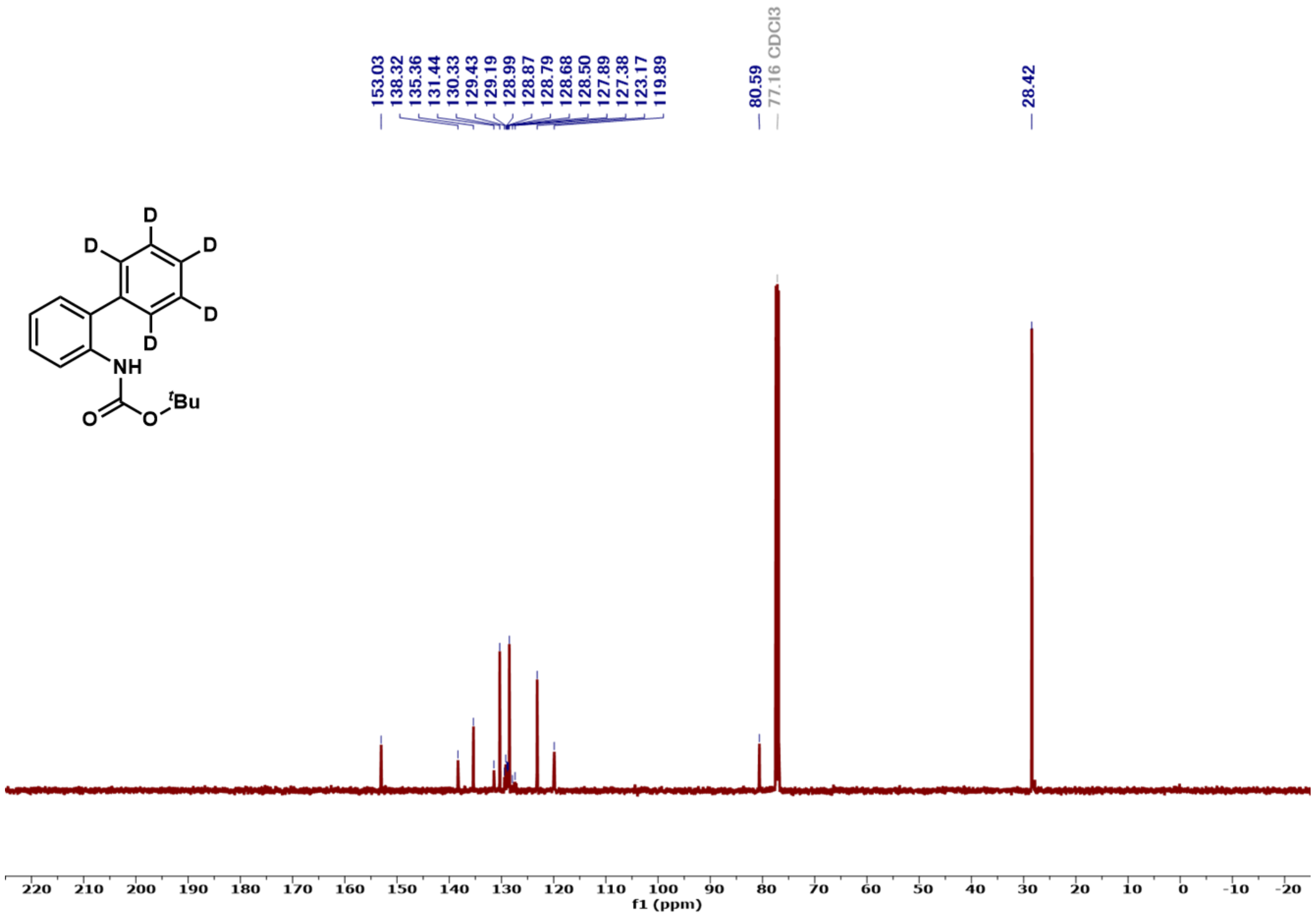

^{13}C NMR spectrum (126 MHz, $CDCl_3$, 25 °C) of ***tert*-butyl ([1,1'-biphenyl]-2-yl-2',3',4',5',6'-d5)carbamate (2-d_5)**.

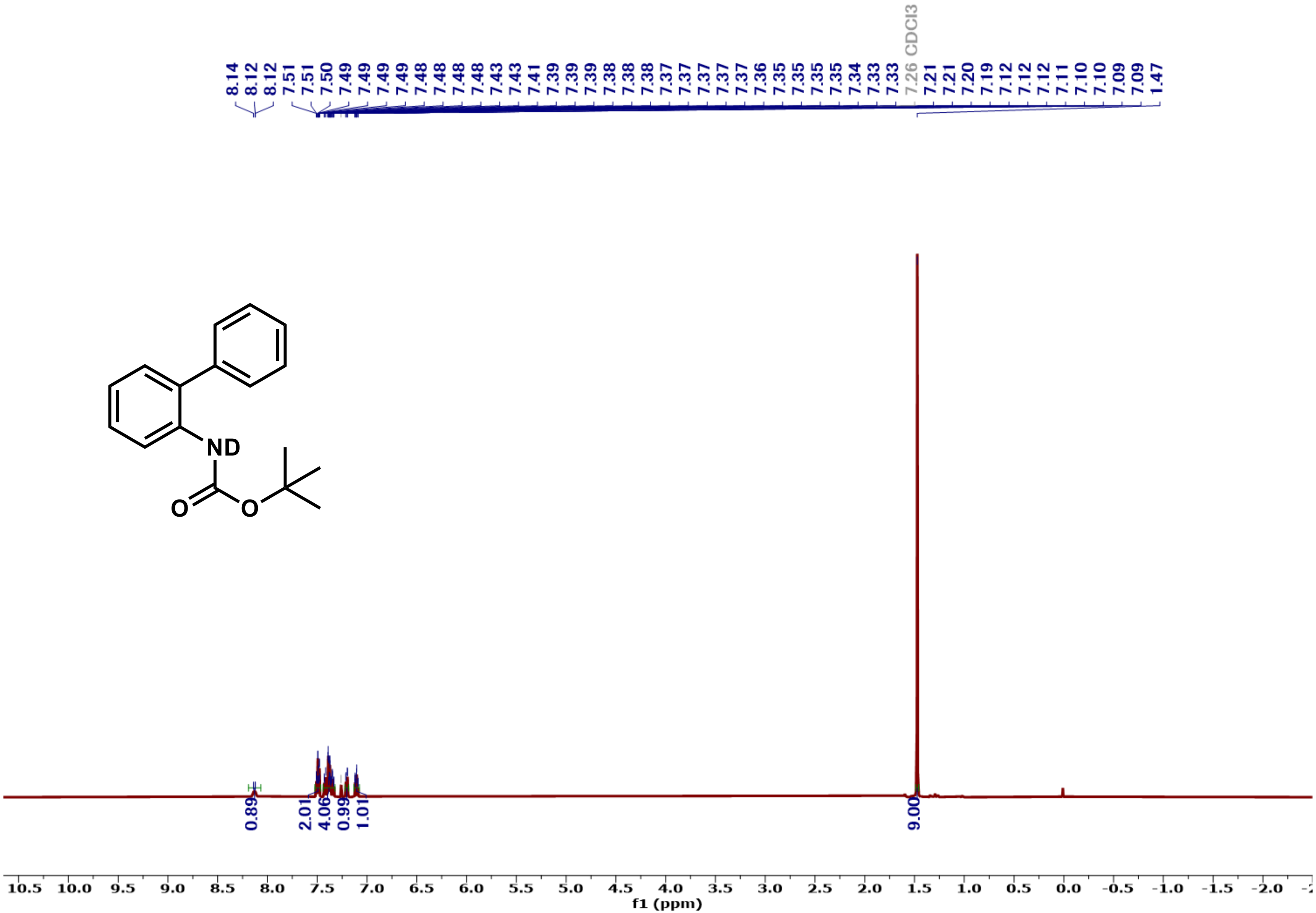

^{1}H NMR spectrum (500 MHz, $CDCl_3$, 25 °C) of ***tert*-butyl deuterium[1,1'-biphenyl]-2-ylcarbamate**.

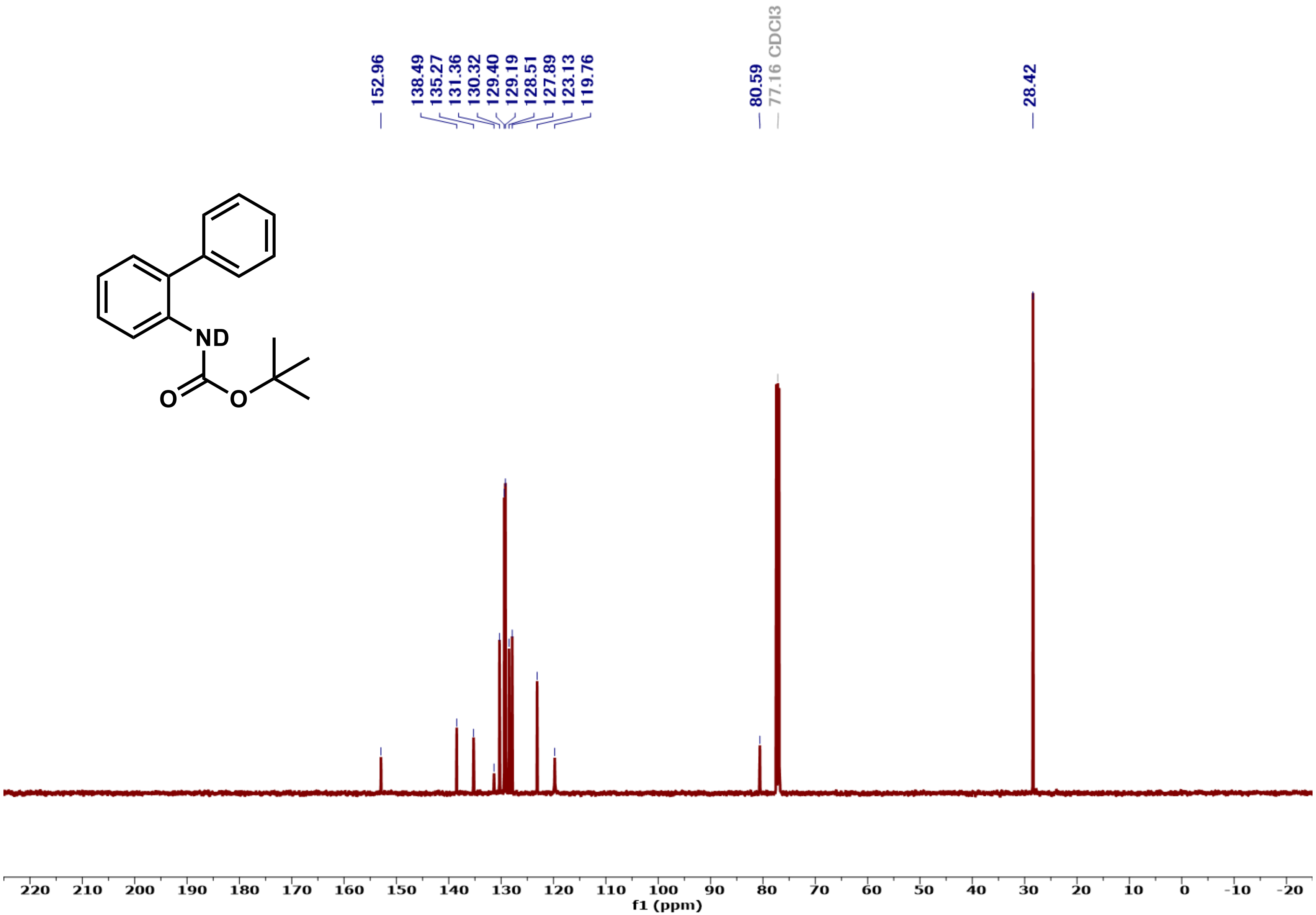

^{13}C NMR spectrum (126 MHz, $CDCl_3$, 25 °C) of ***tert*-butyl deuterium[1,1'-biphenyl]-2-ylcarbamate**.

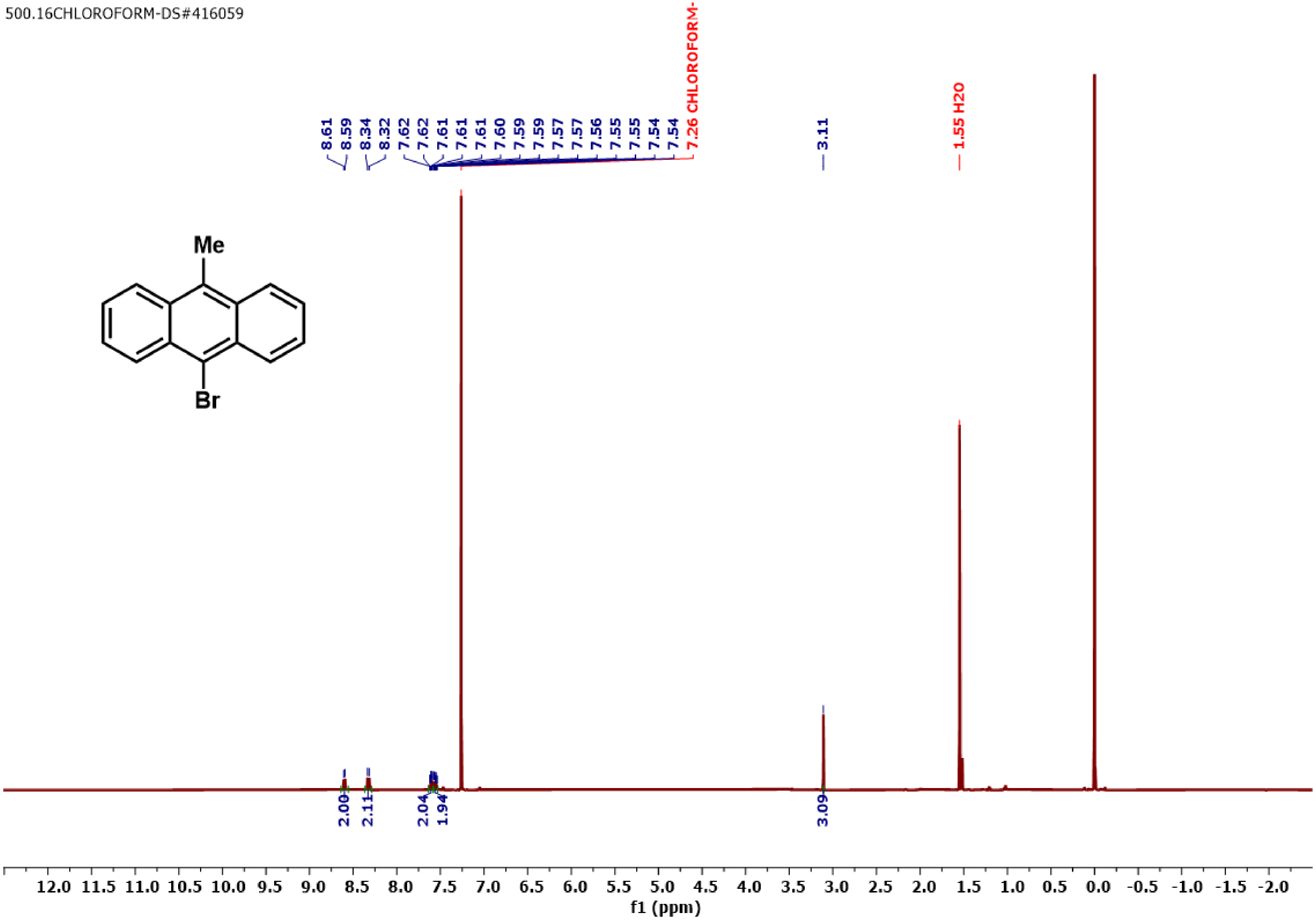

^{1}H NMR spectrum (500 MHz, $CDCl_3$, 25 °C) of **9-Bromo-10-methylanthracene**.

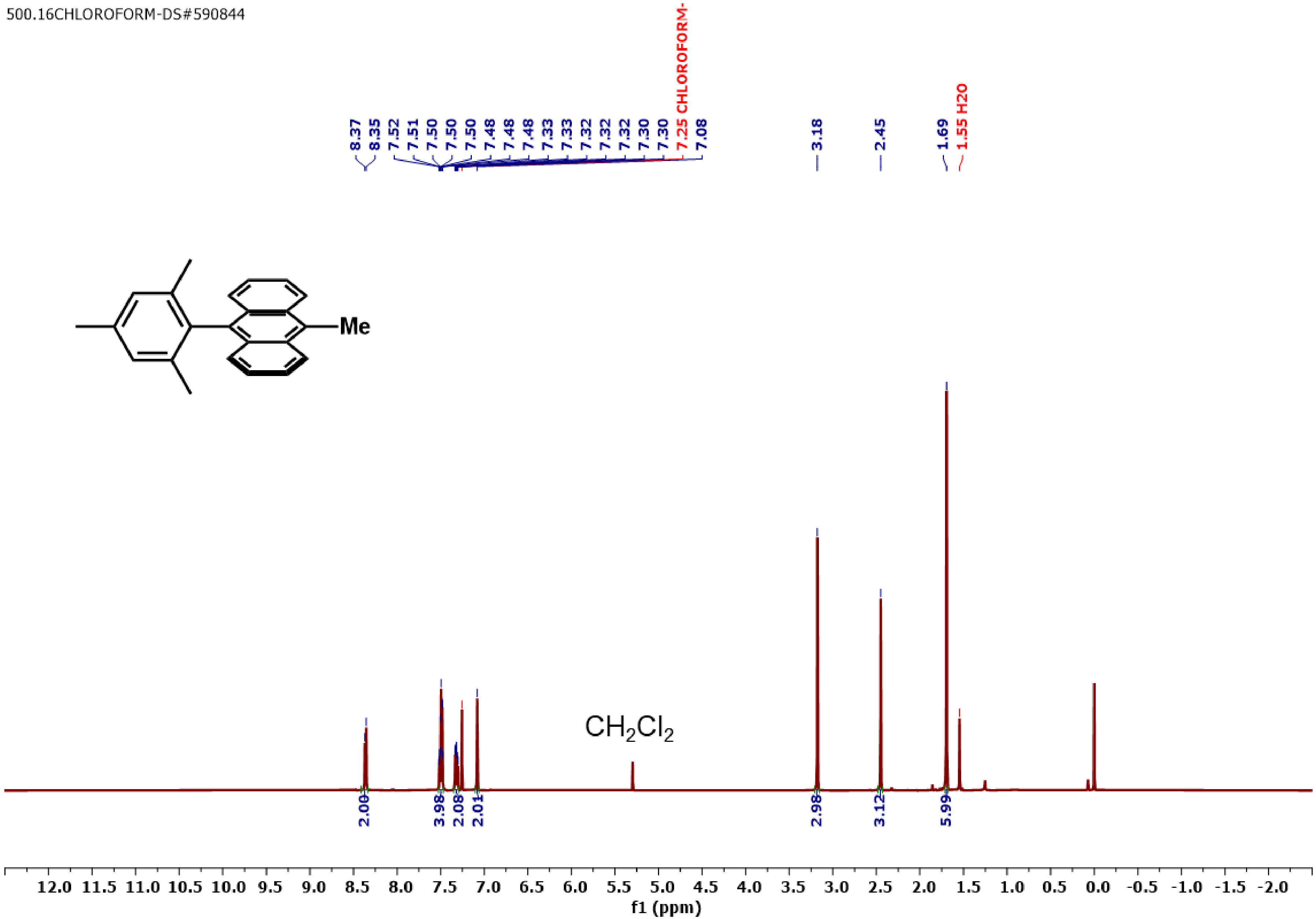

^{1}H NMR spectrum (500 MHz, $CDCl_3$, 25 °C) of **9-mesityl-10-methylanthracene (1i)**.

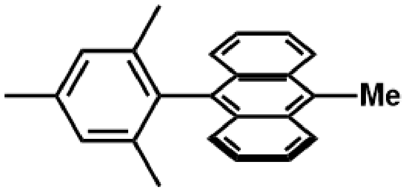

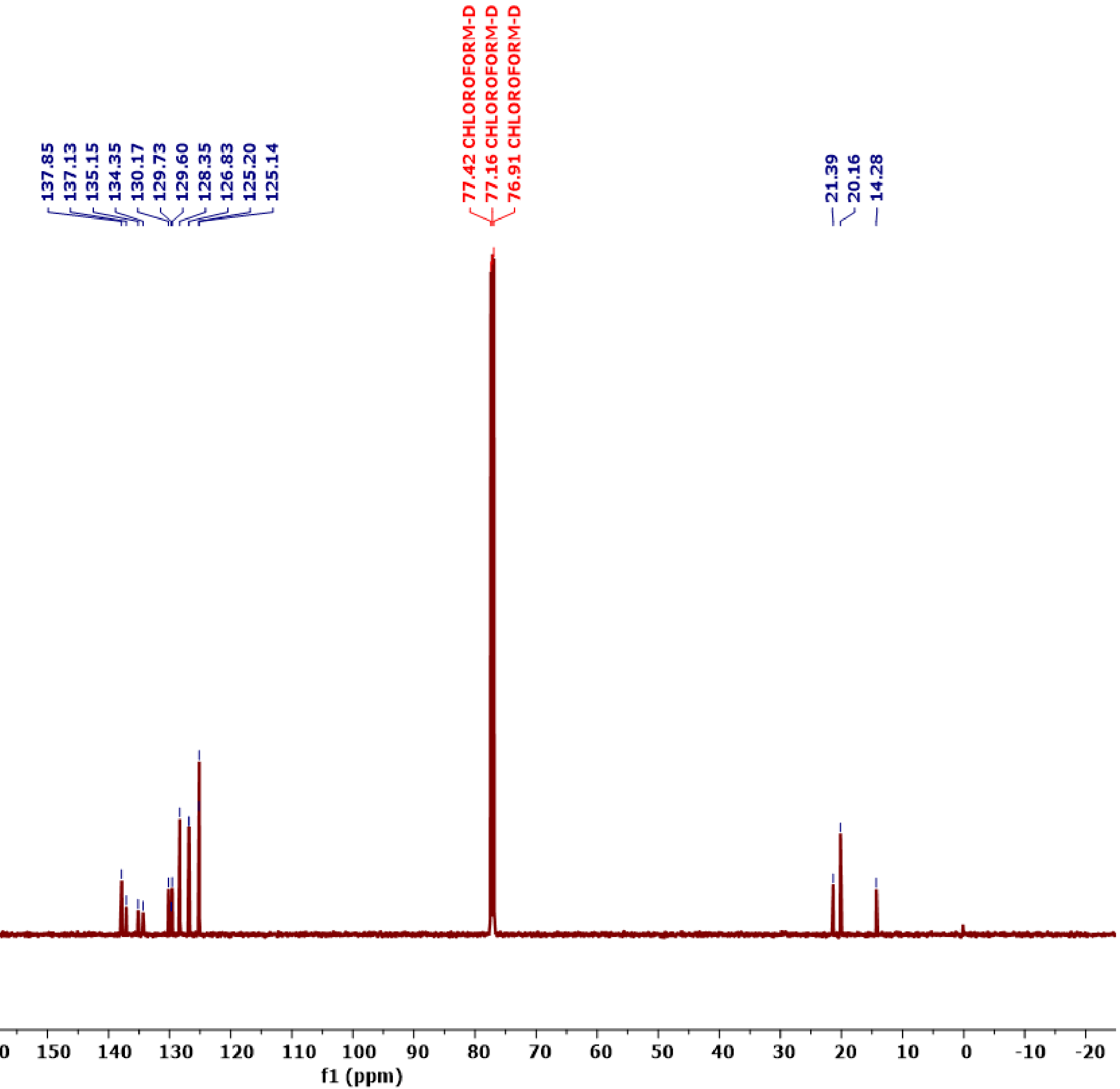

^{13}C NMR spectrum (126 MHz, $CDCl_3$, 25 °C) of 9**-mesityl-10-methylanthracene (1i)**.

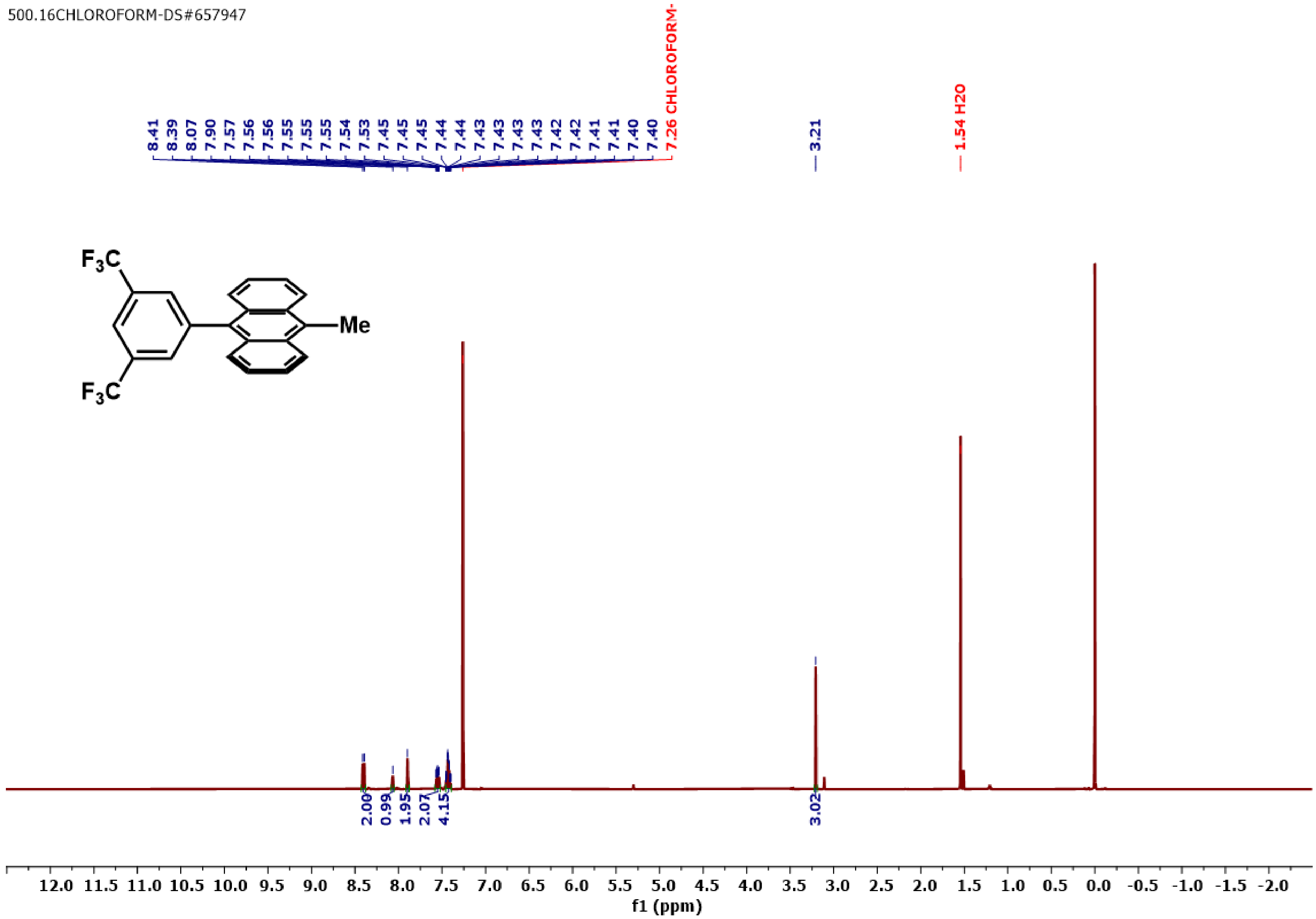

^{1}H NMR spectrum (500 MHz, $CDCl_3$, 25 °C) of 9-(3,5-bis(trifluoromethyl)phenyl)-10-methylanthracene (**1j**).

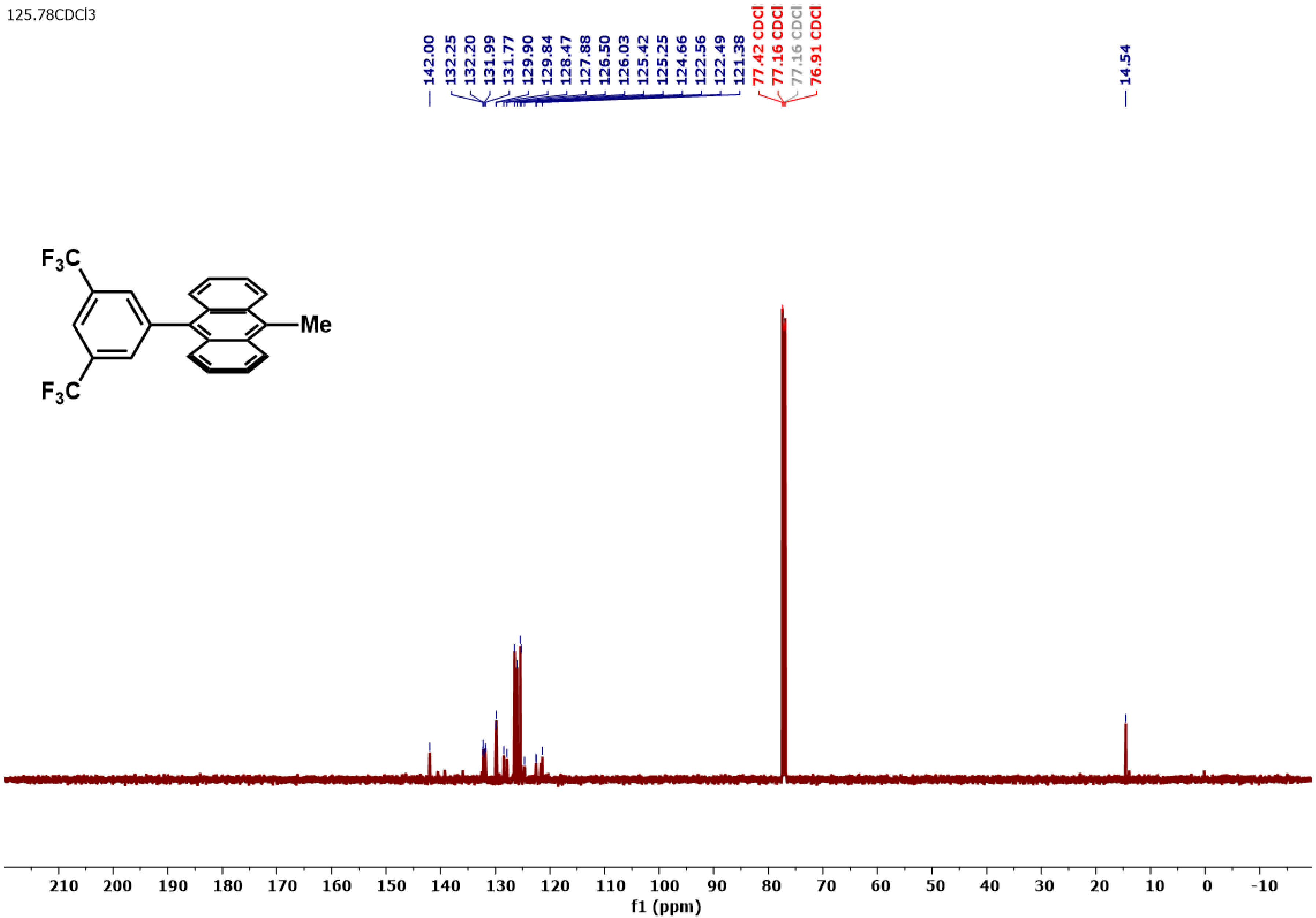

^{13}C NMR spectrum (126 MHz, $CDCl_3$, 25 °C) of 9-(3,5-bis(trifluoromethyl)phenyl)-10-methylanthracene (**1j**).

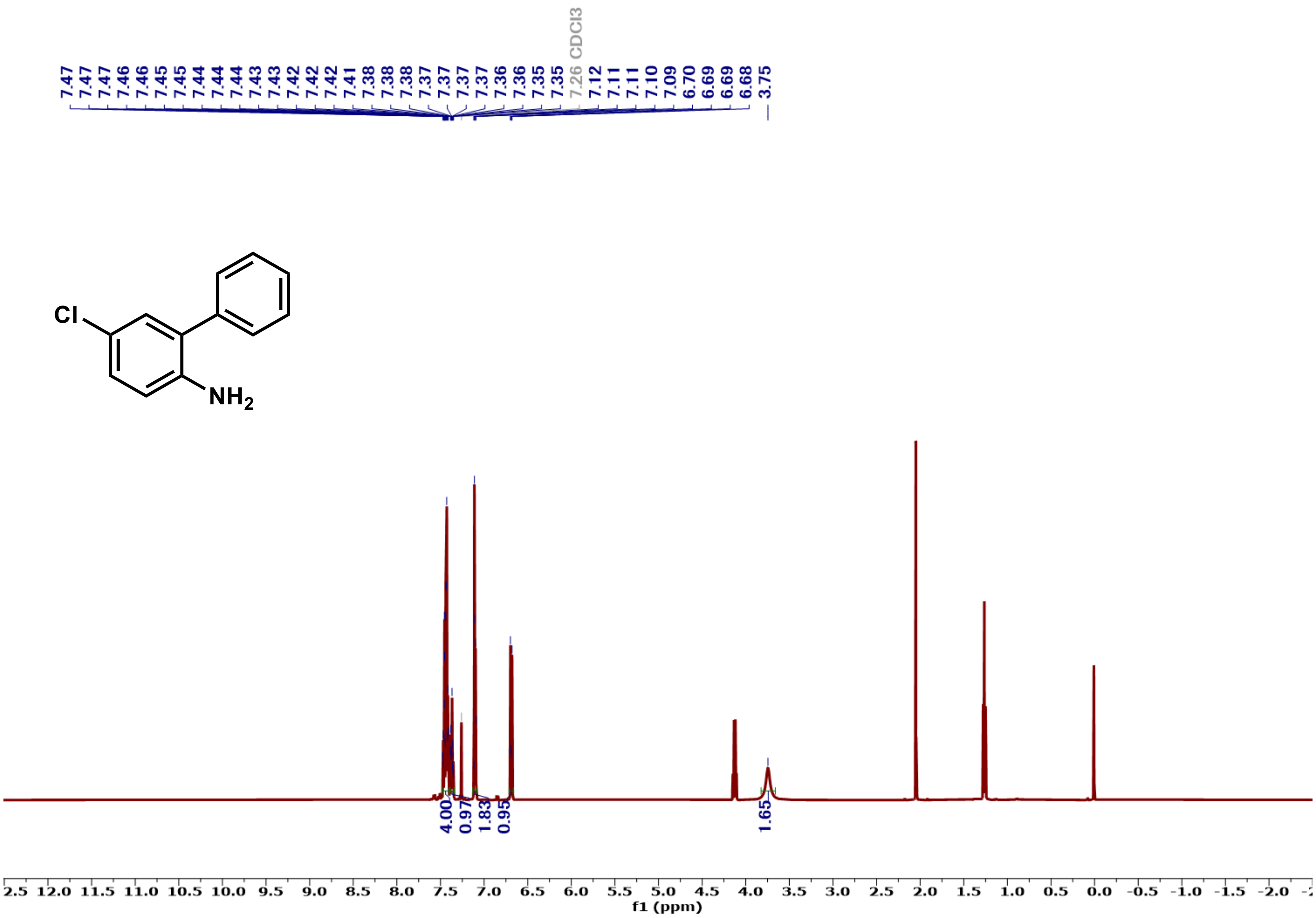

^{1}H NMR spectrum (500 MHz, $CDCl_3$, 25 °C) of **5-chloro-[1,1'-biphenyl]-2-amine**.

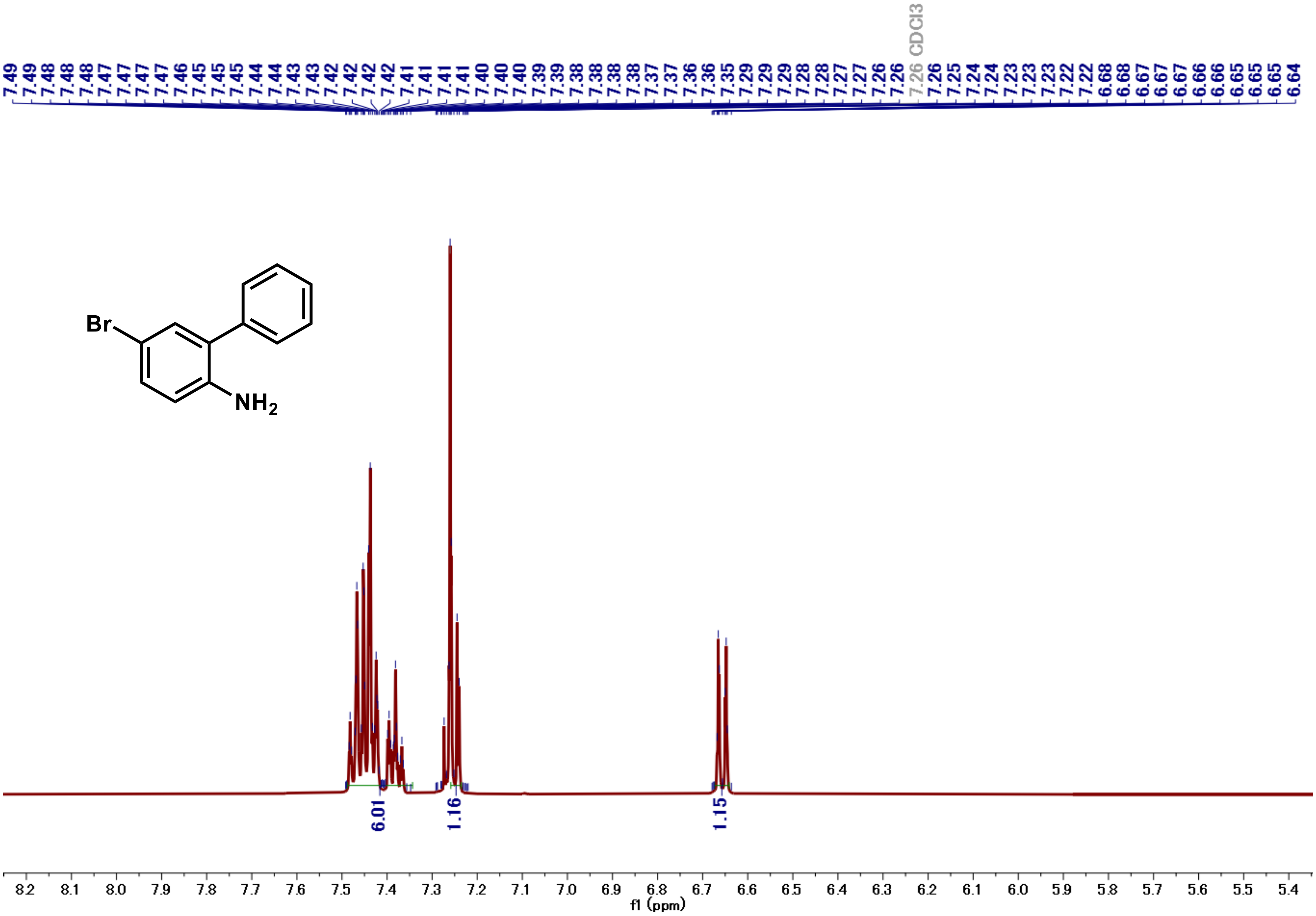

^{1}H NMR spectrum (500 MHz, $CDCl_3$, 25 °C) of **5-bromo-[1,1'-biphenyl]-2-amine**.

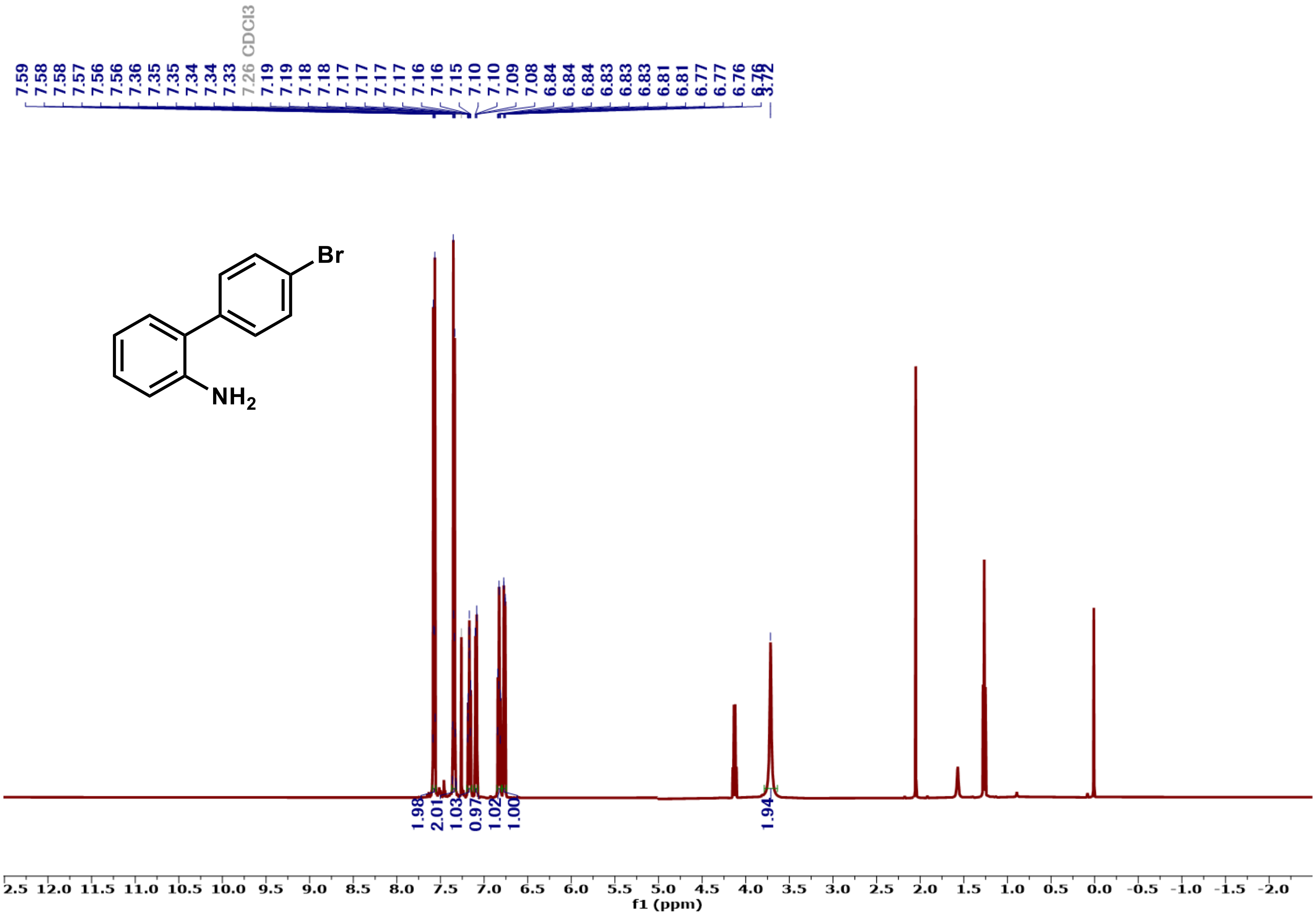

^{1}H NMR spectrum (500 MHz, $CDCl_3$, 25 °C) of **4'-bromo-[1,1'-biphenyl]-2-amine**.

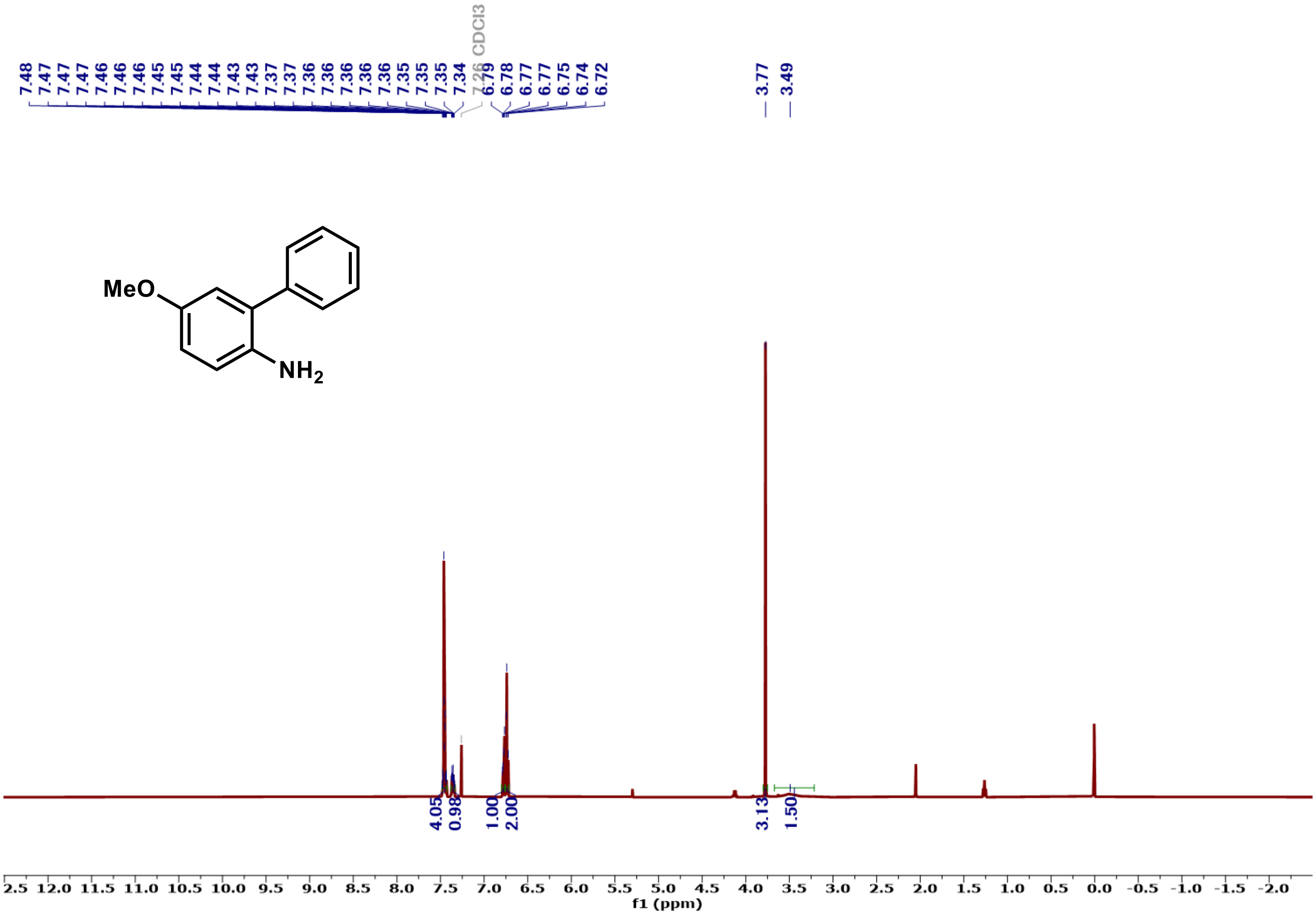

^{1}H NMR spectrum (500 MHz, $CDCl_3$, 25 °C) of **5-methoxy-[1,1'-biphenyl]-2-amine**.

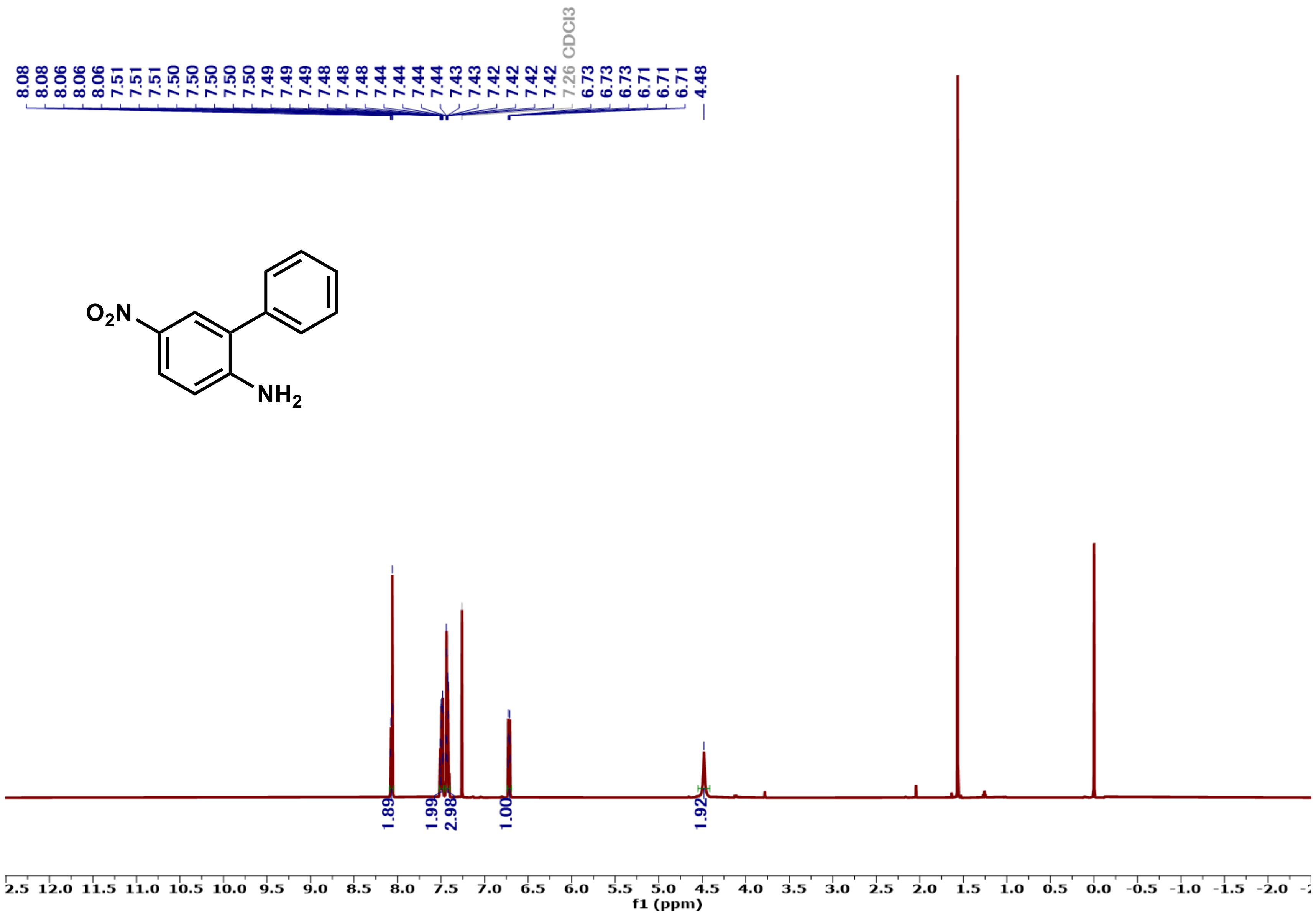

^{1}H NMR spectrum (500 MHz, $CDCl_3$, 25 °C) of **5-nitro-[1,1'-biphenyl]-2-amine**.

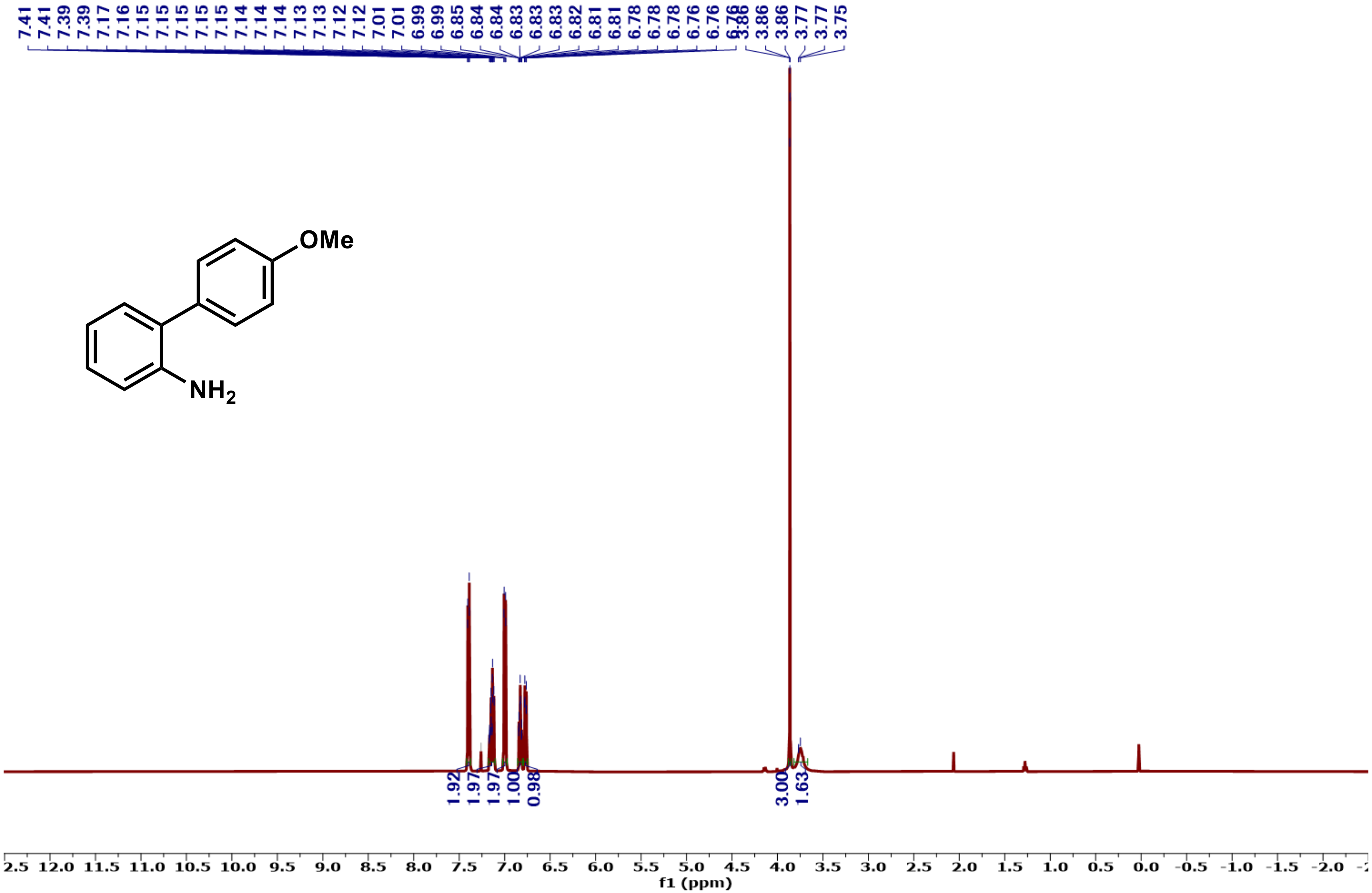

^{1}H NMR spectrum (500 MHz, $CDCl_3$, 25 °C) of **4'-methoxy-[1,1'-biphenyl]-2-amine**.

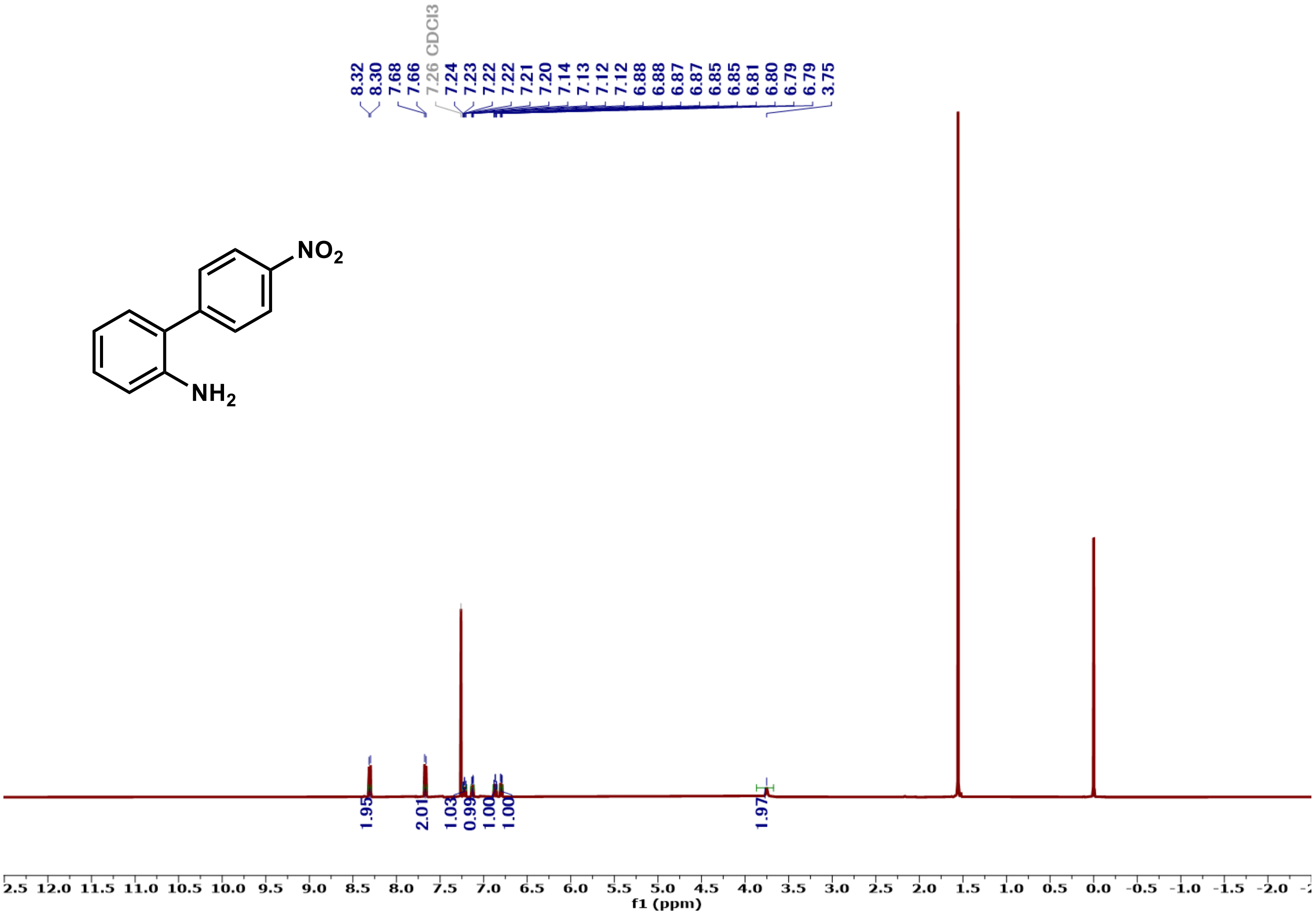

^{1}H NMR spectrum (500 MHz, $CDCl_3$, 25 °C) of **4'-nitro-[1,1'-biphenyl]-2-amine**.

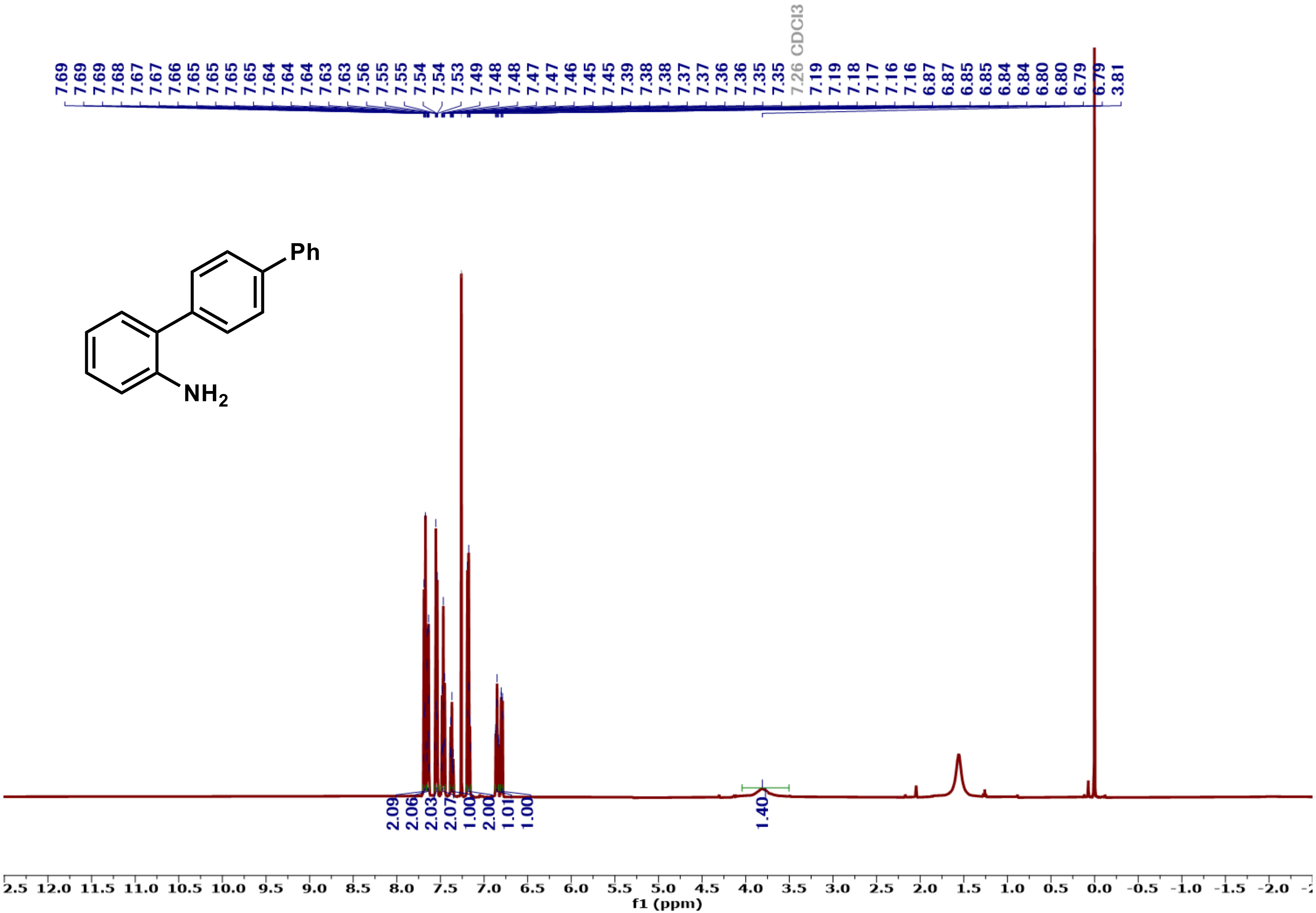

^{1}H NMR spectrum (500 MHz, $CDCl_3$, 25 °C) of **[1,1':4',1''-terphenyl]-2-amine**.

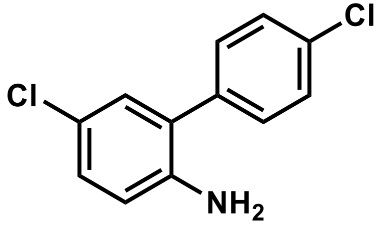

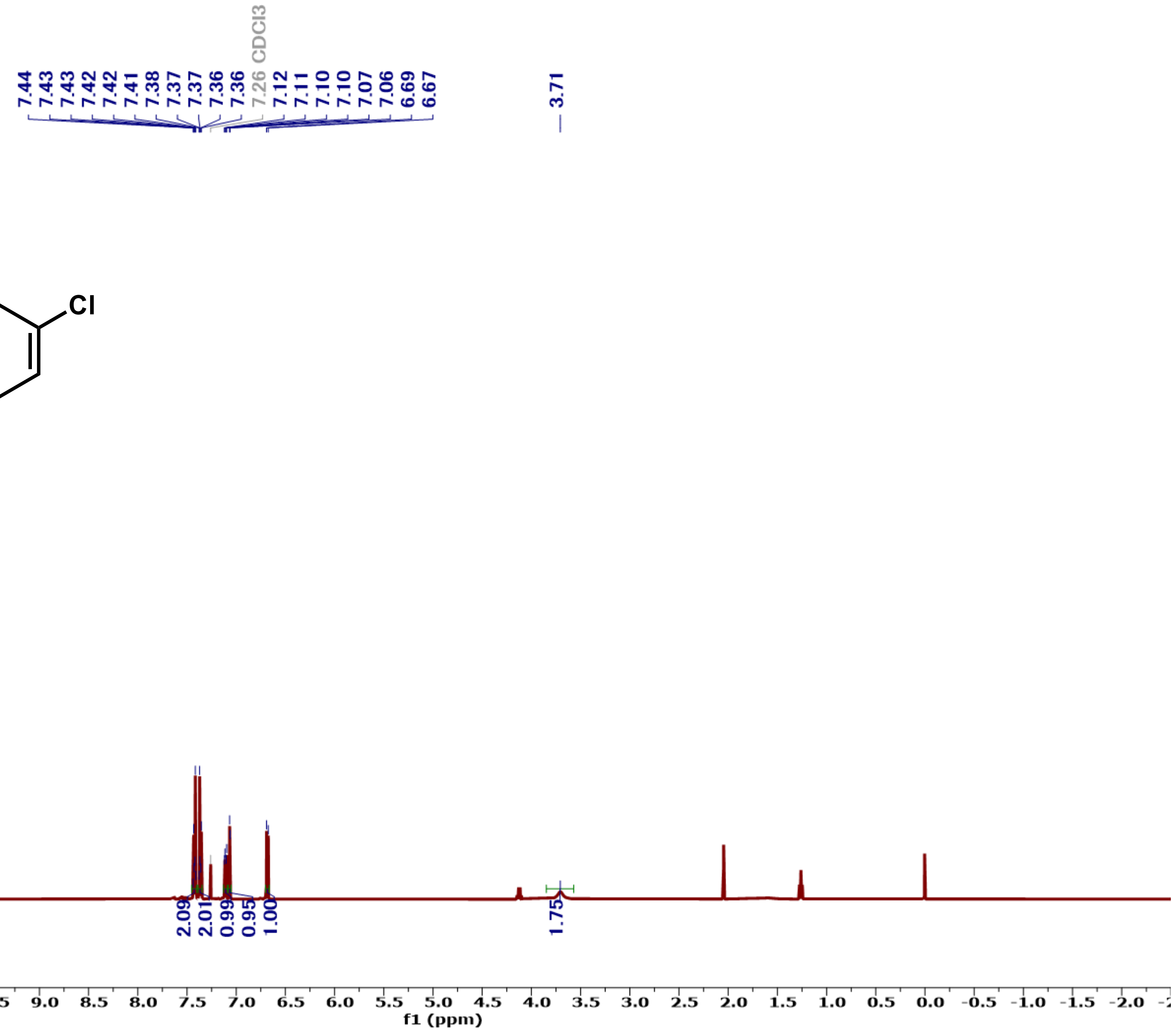

^{1}H NMR spectrum (500 MHz, $CDCl_3$, 25 °C) of **4',5-chloro-[1,1'-biphenyl]-2-amine**.

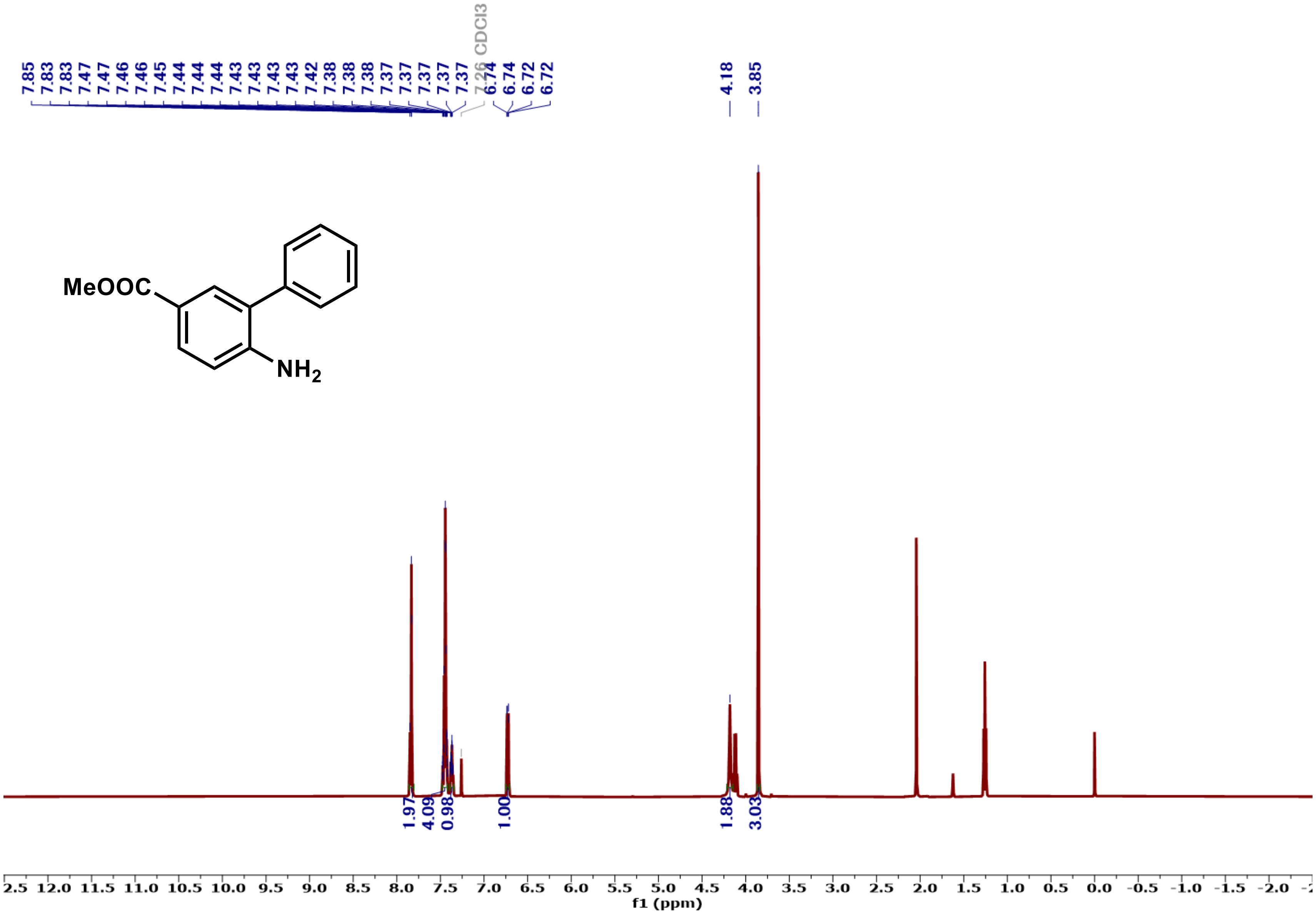

^{1}H NMR spectrum (500 MHz, $CDCl_3$, 25 °C) of **methyl 6-amino-[1,1'-biphenyl]-3-carboxylate**.

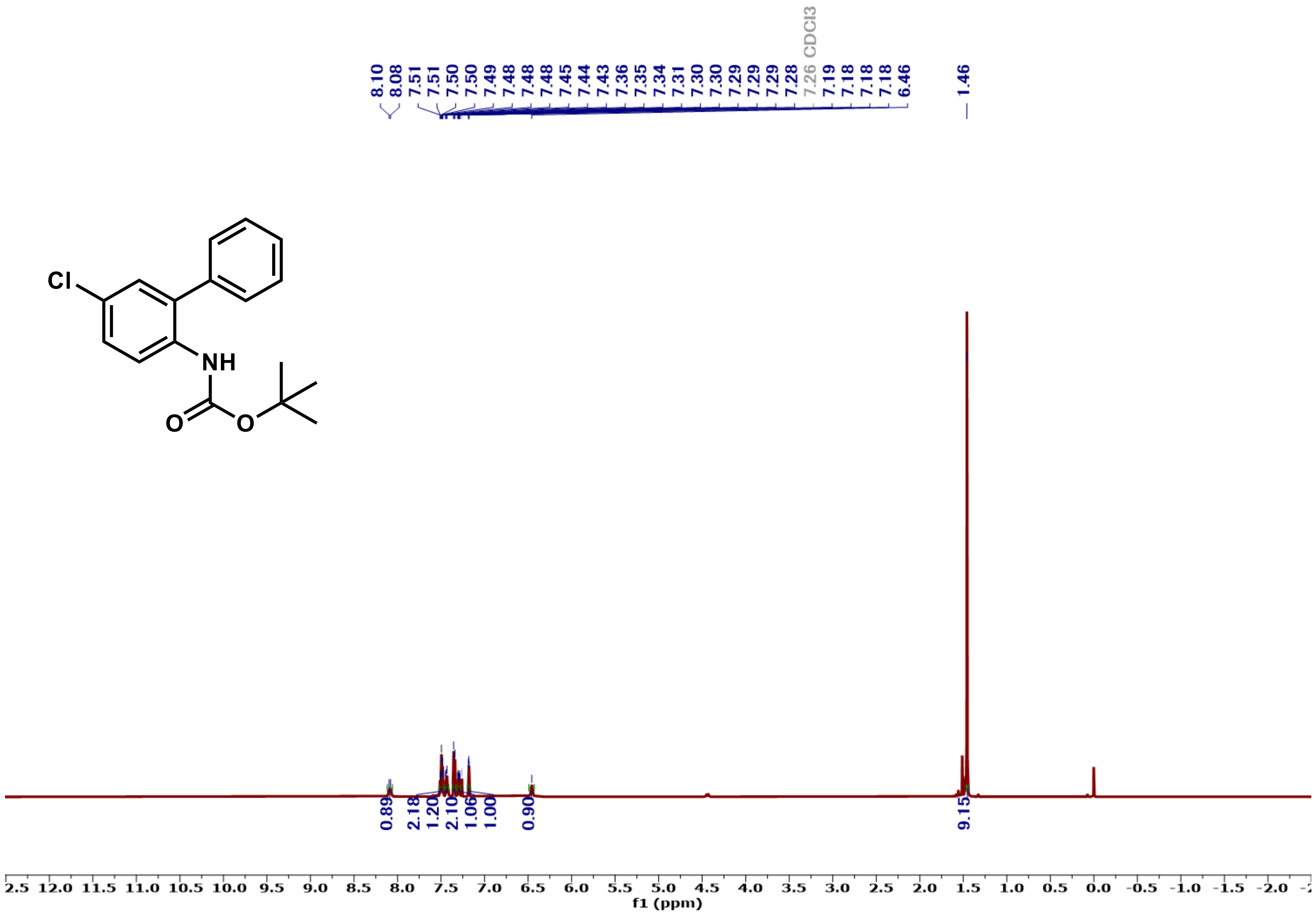

^{1}H NMR spectrum (500 MHz, $CDCl_3$, 25 °C) of ***tert*-butyl (5-chloro-[1,1'-biphenyl]-2-yl)carbamate (2e)**.

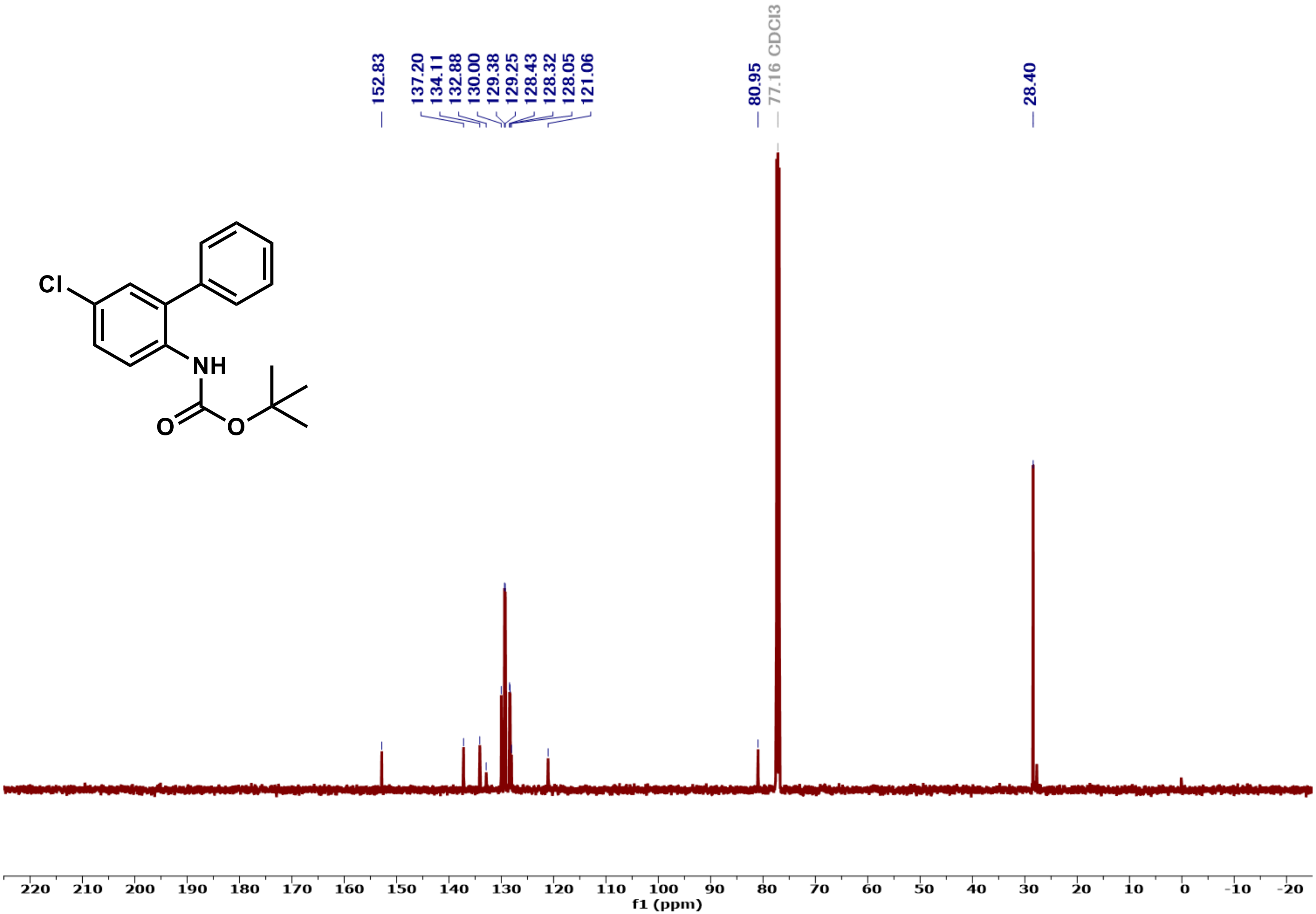

^{13}C NMR spectrum (126 MHz, $CDCl_3$, 25 °C) of ***tert*-butyl (5-chloro-[1,1'-biphenyl]-2-yl)carbamate (2e)**.

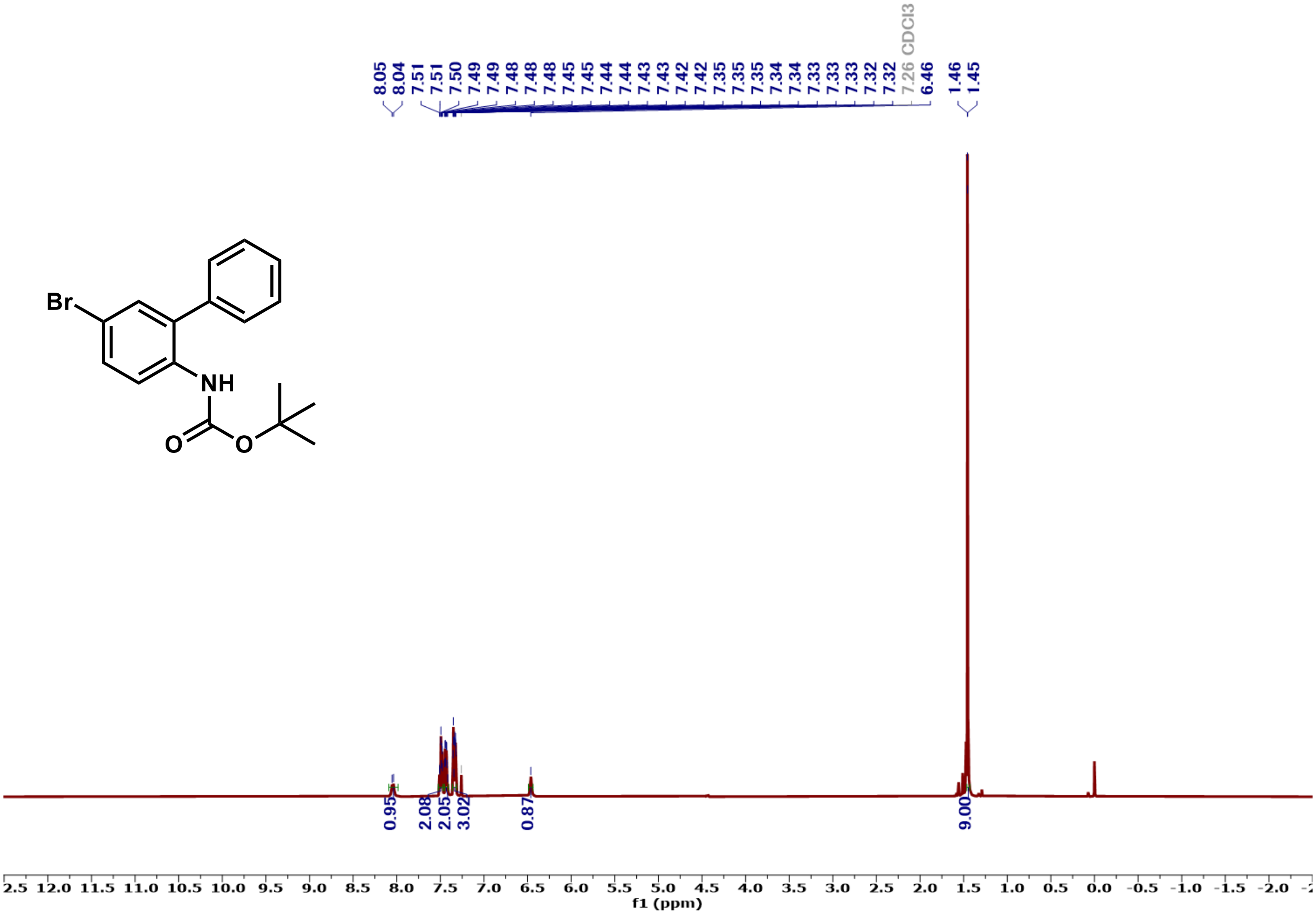

^{1}H NMR spectrum (500 MHz, $CDCl_3$, 25 °C) of ***tert*-butyl (5-bromo-[1,1'-biphenyl]-2-yl)carbamate (2f)**.

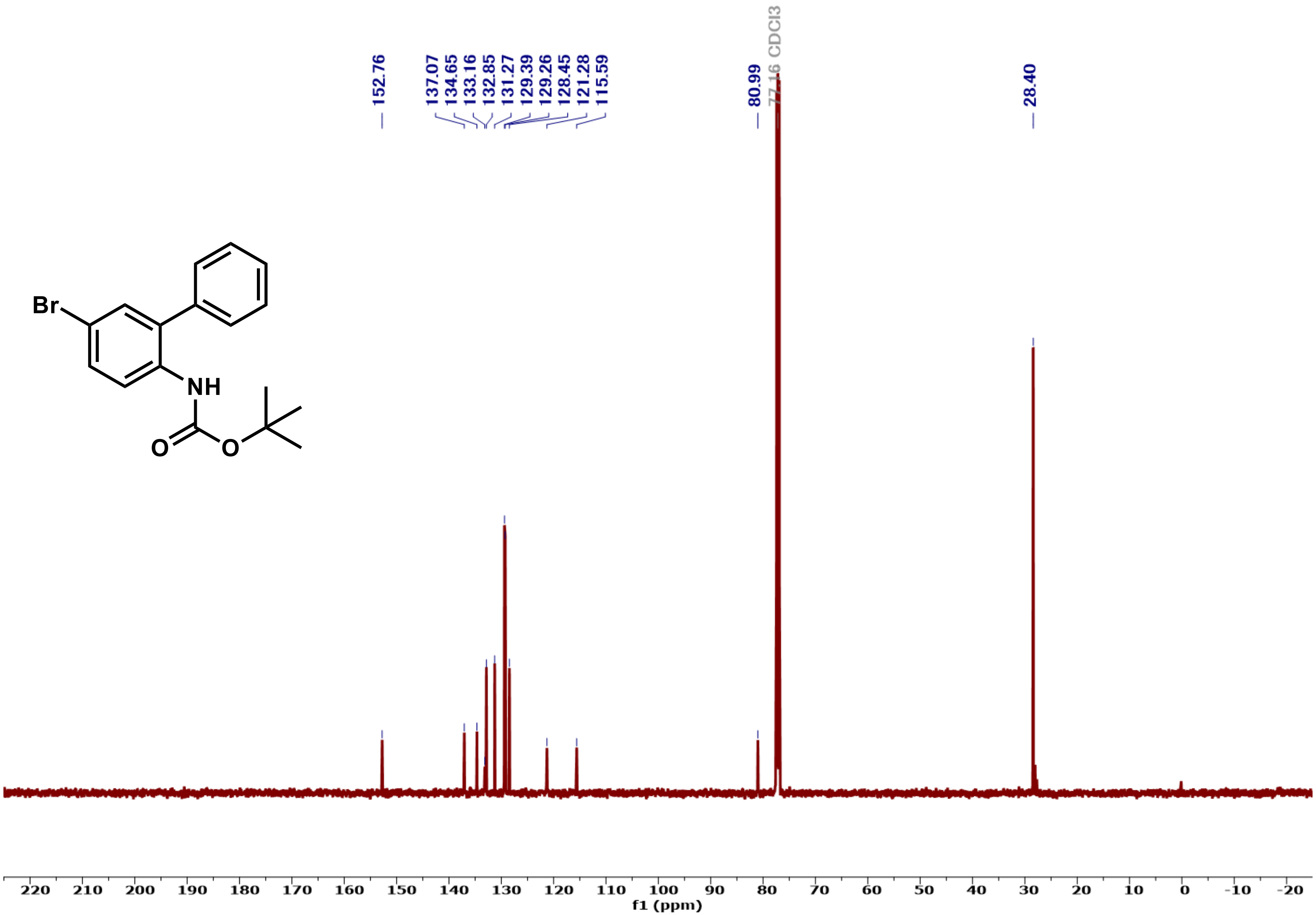

^{13}C NMR spectrum (126 MHz, $CDCl_3$, 25 °C) of ***tert*-butyl (5-bromo-[1,1'-biphenyl]-2-yl)carbamate (2f)**.

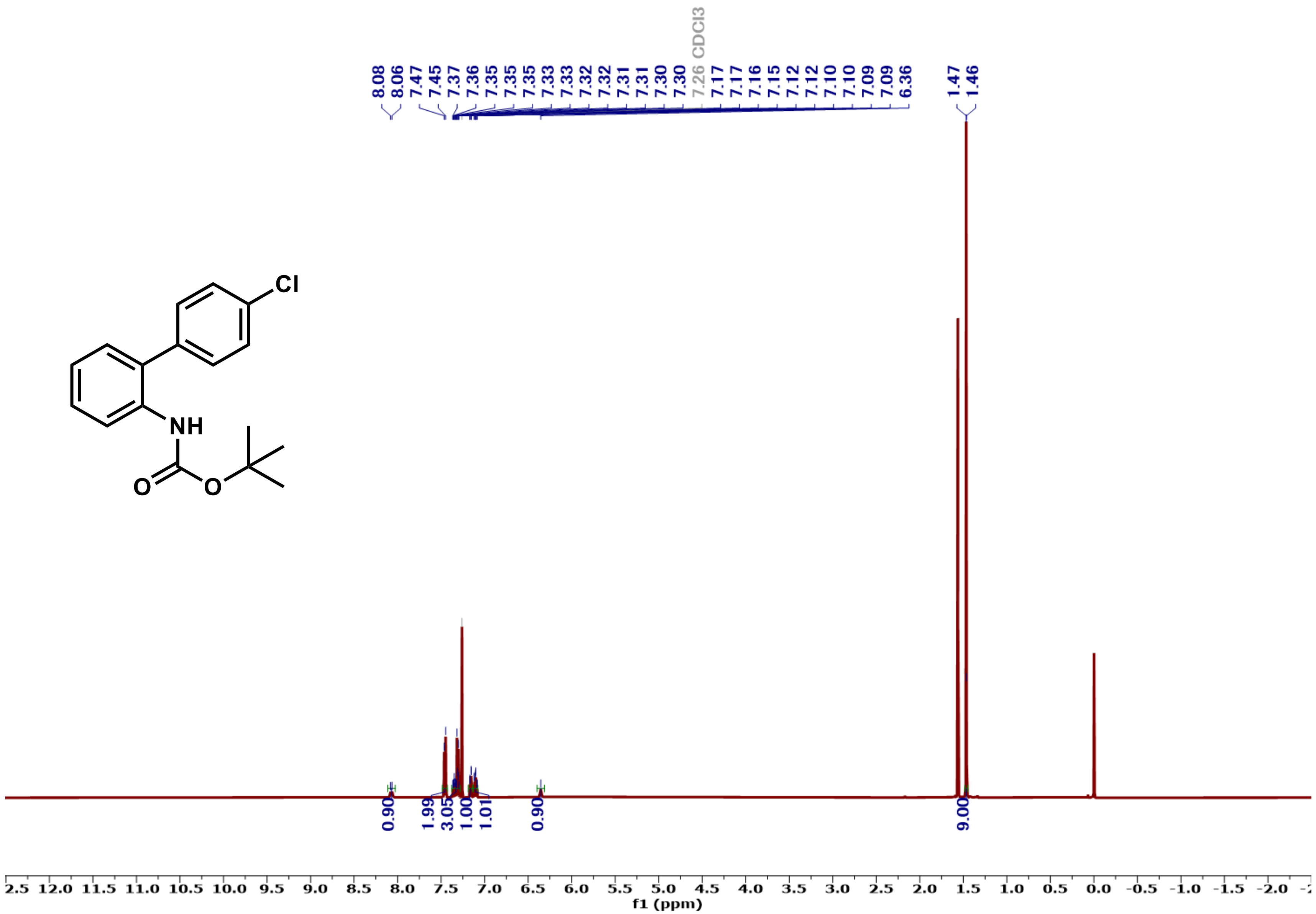

^{1}H NMR spectrum (500 MHz, $CDCl_3$, 25 °C) of ***tert*-butyl (4'-chloro-[1,1'-biphenyl]-2-yl)carbamate (2g)**.

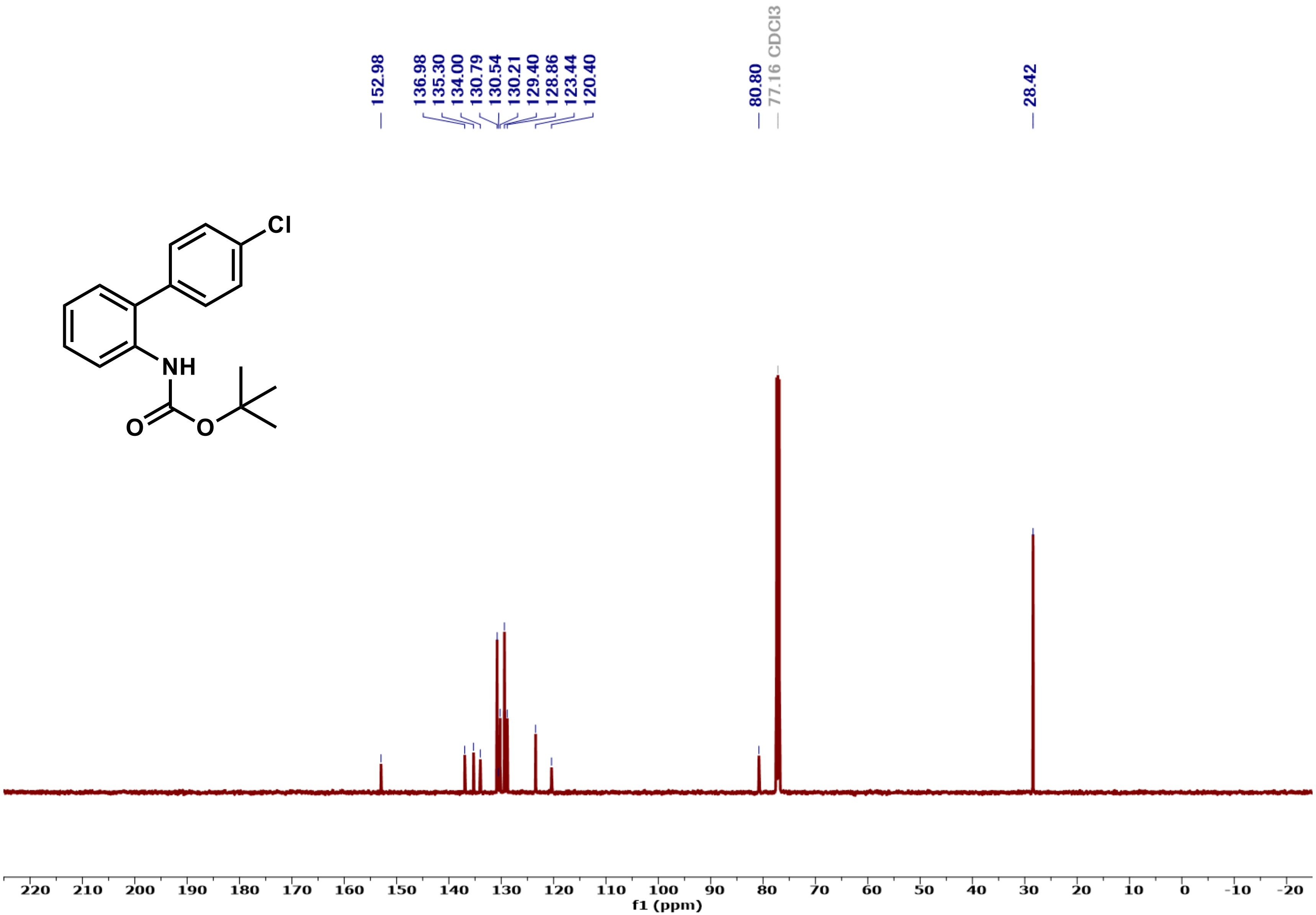

^{13}C NMR spectrum (126 MHz, $CDCl_3$, 25 °C) of ***tert*-butyl (4'-chloro-[1,1'-biphenyl]-2-yl)carbamate (2g)**.

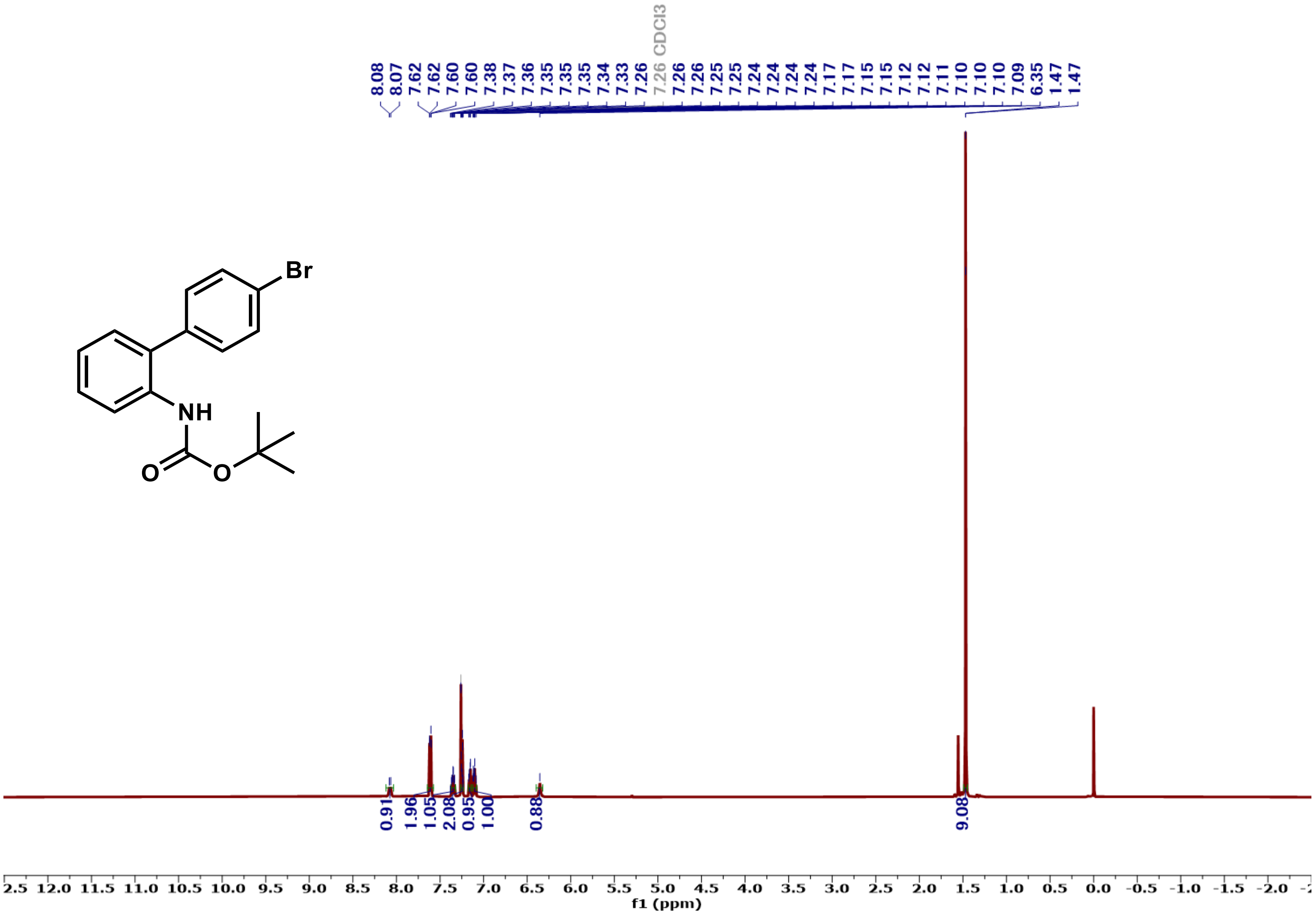

^{1}H NMR spectrum (500 MHz, $CDCl_3$, 25 °C) of ***tert*-butyl (4'-bromo-[1,1'-biphenyl]-2-yl)carbamate (2h)**.

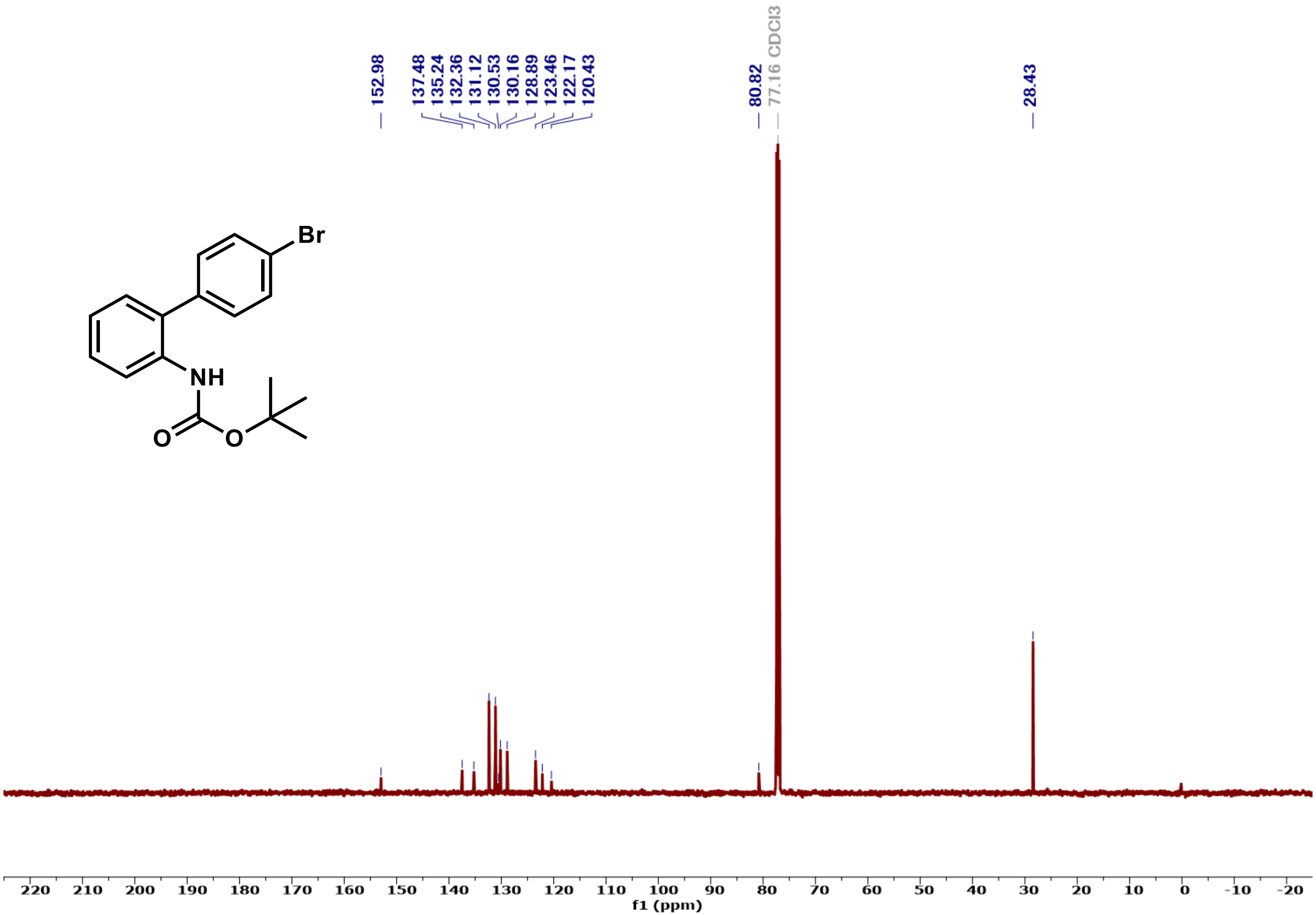

^{13}C NMR spectrum (126 MHz, $CDCl_3$, 25 °C) of ***tert*-butyl (4'-bromo-[1,1'-biphenyl]-2-yl)carbamate (2h)**.

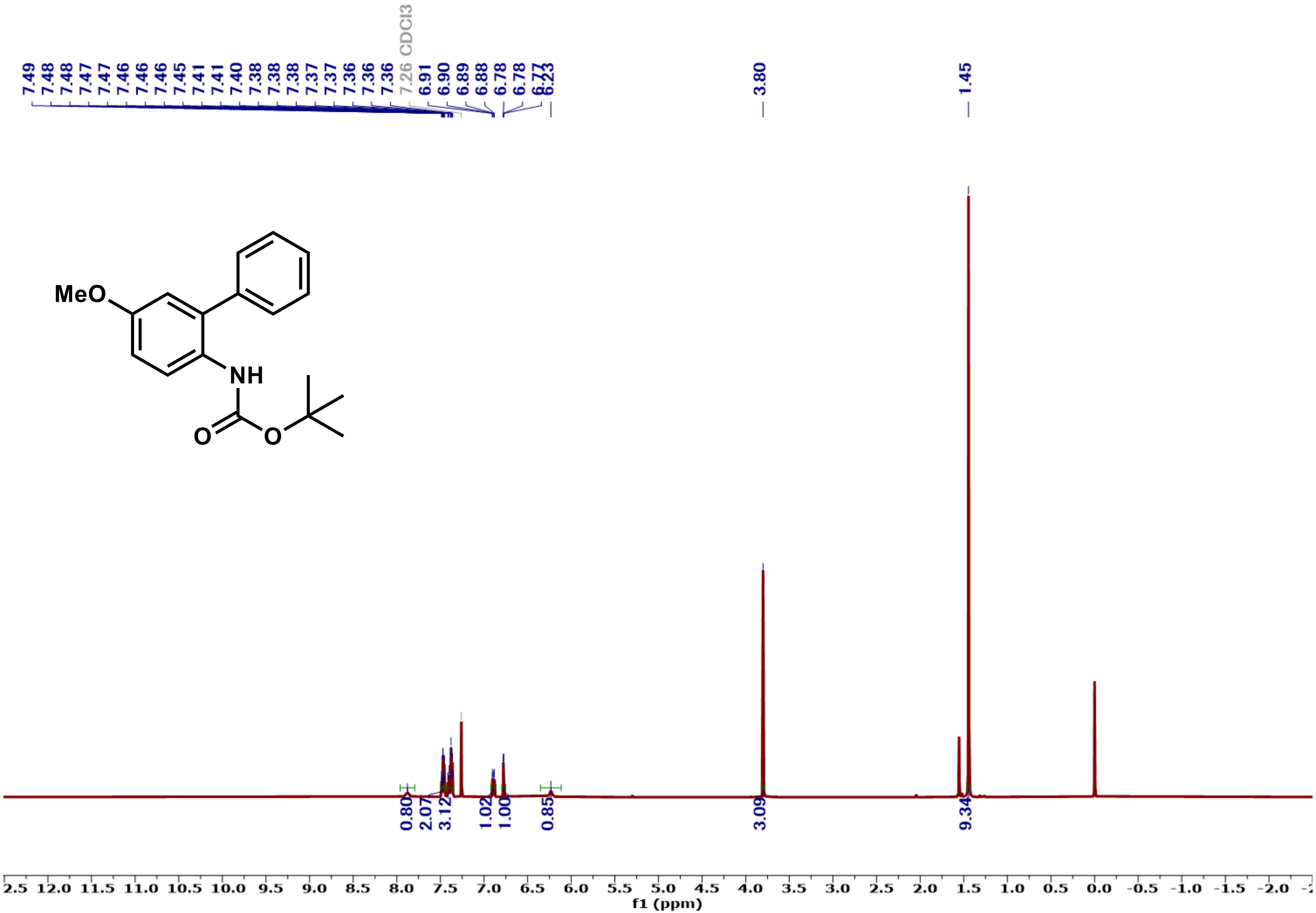

^{1}H NMR spectrum (500 MHz, $CDCl_3$, 25 °C) of ***tert*-butyl (5-methoxy-[1,1'-biphenyl]-2-yl)carbamate (2i)**.

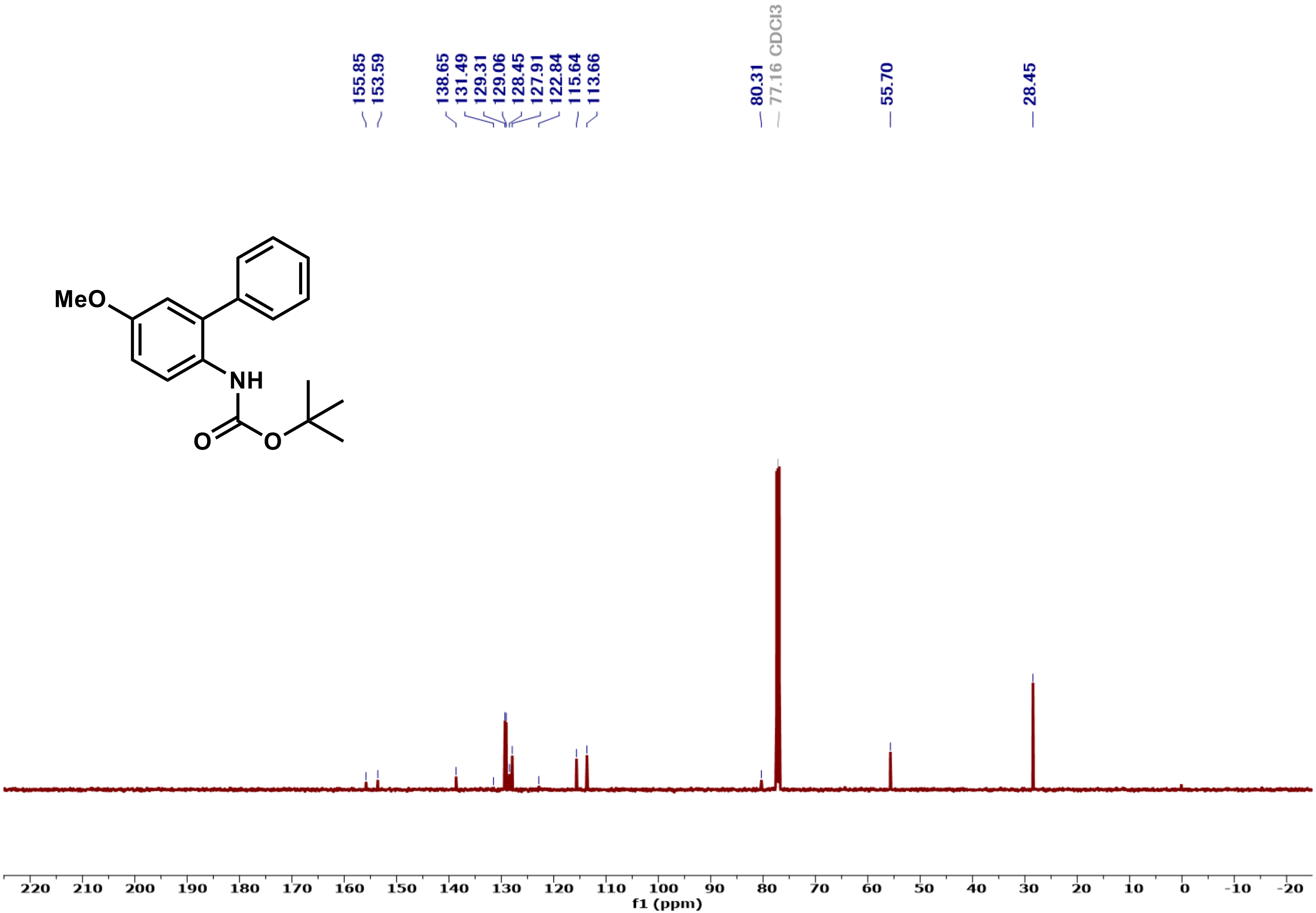

^{13}C NMR spectrum (126 MHz, $CDCl_3$, 25 °C) of ***tert*-butyl (5-methoxy-[1,1'-biphenyl]-2-yl)carbamate (2i)**.

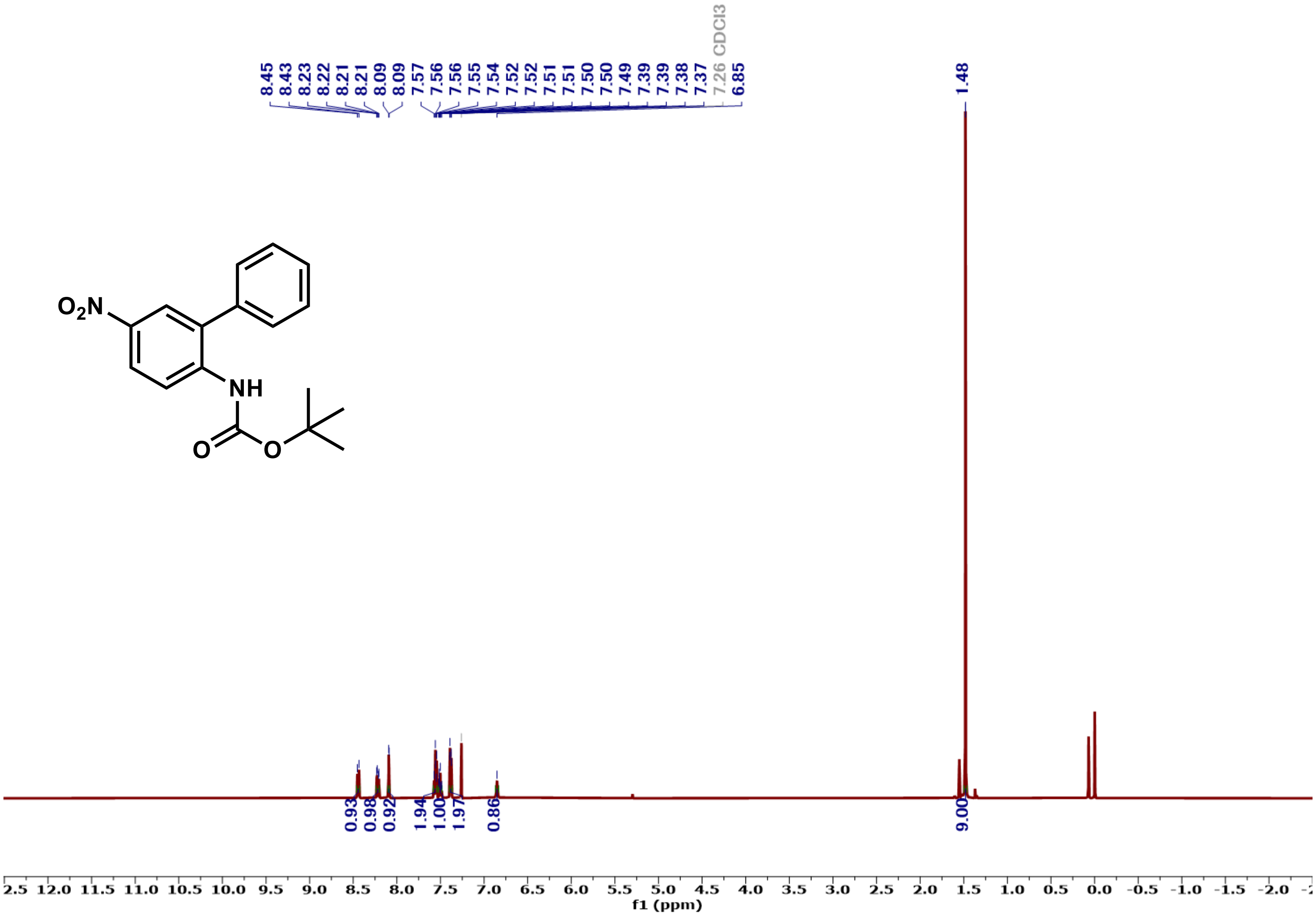

^{1}H NMR spectrum (500 MHz, $CDCl_3$, 25 °C) of ***tert*-butyl (5-nitro-[1,1'-biphenyl]-2-yl)carbamate (2j)**.

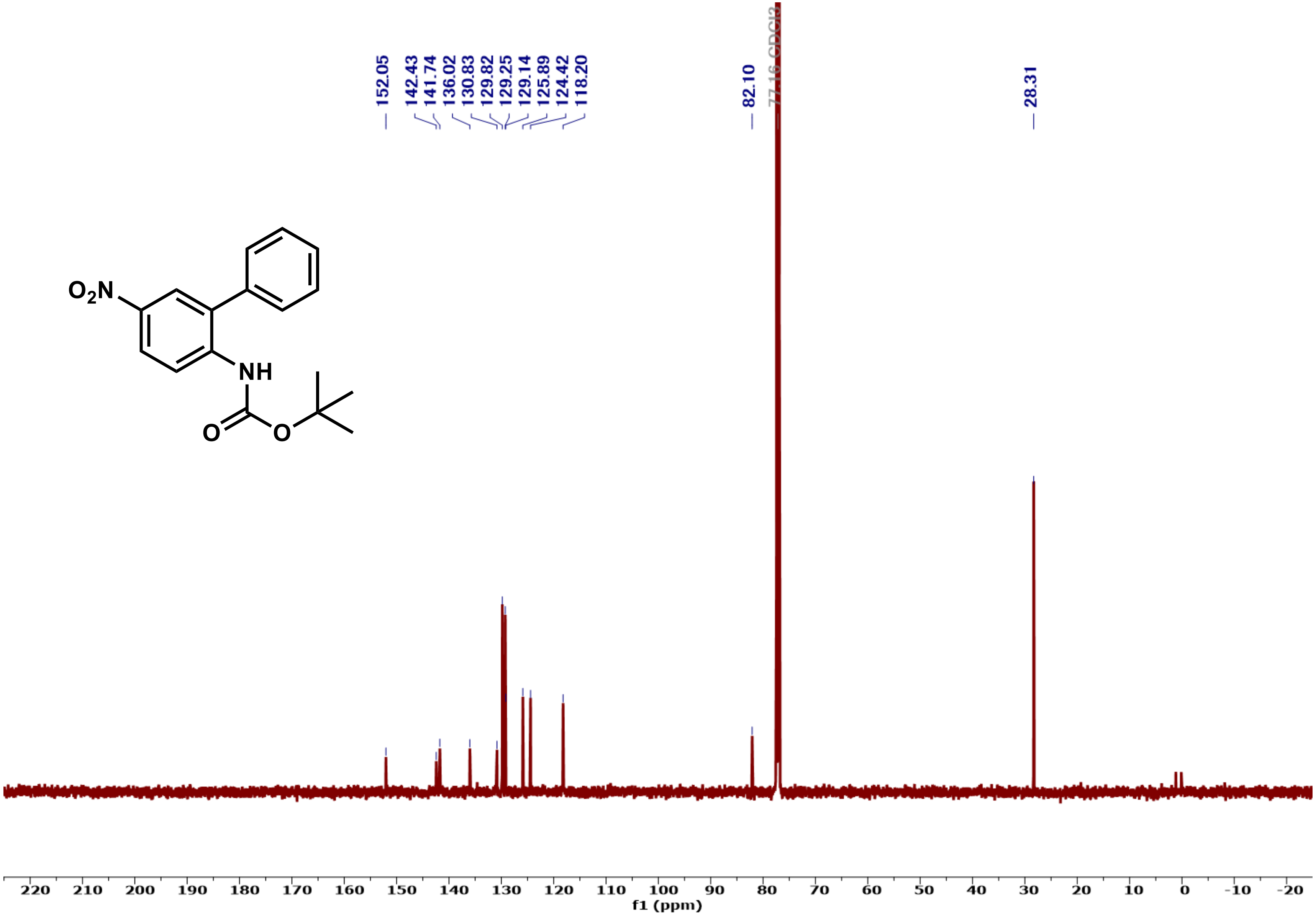

^{13}C NMR spectrum (126 MHz, $CDCl_3$, 25 °C) of ***tert*-butyl (5-nitro-[1,1'-biphenyl]-2-yl)carbamate (2j)**.

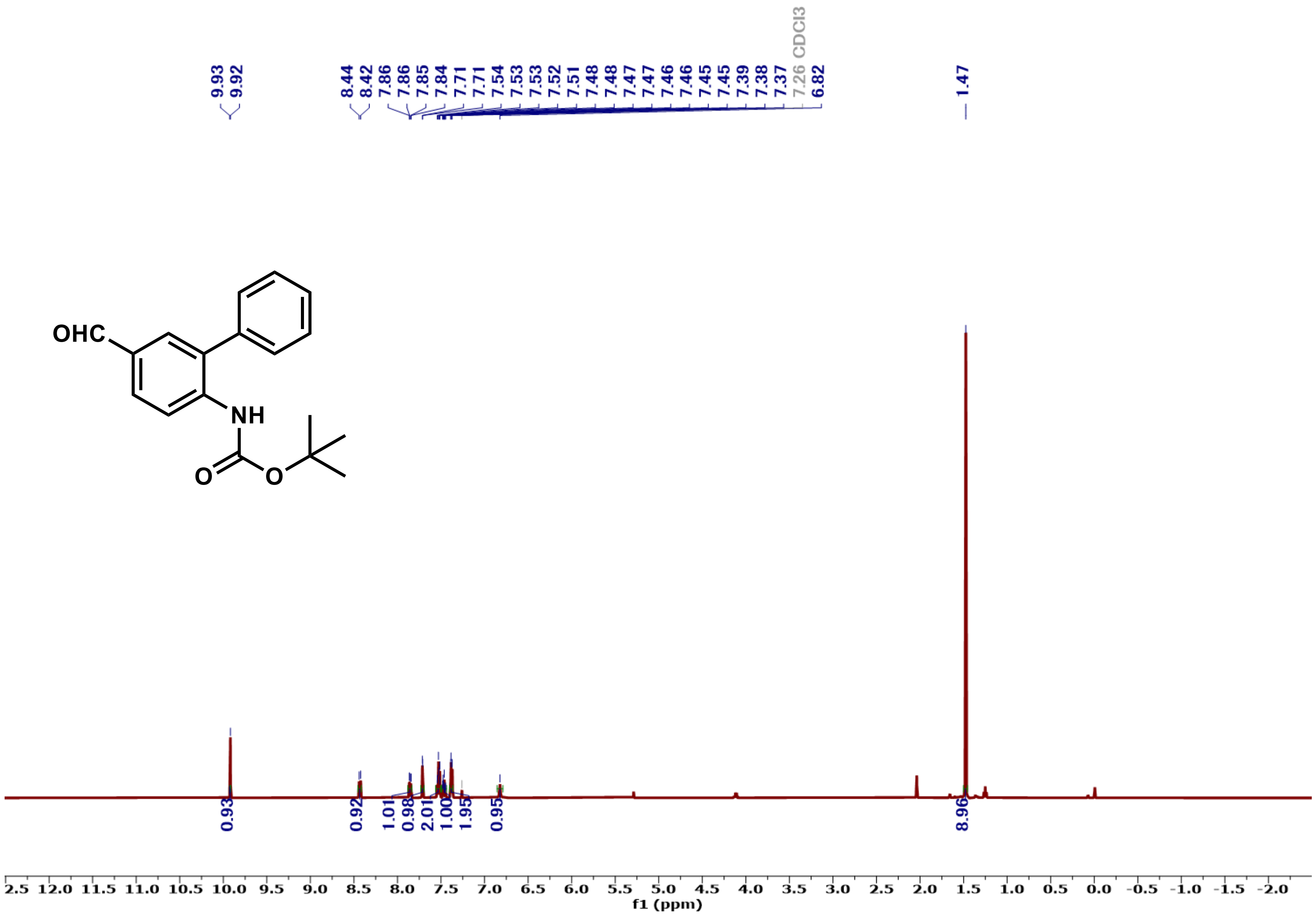

^{1}H NMR spectrum (500 MHz, $CDCl_3$, 25 °C) of ***tert*-butyl (5-formyl-[1,1'-biphenyl]-2-yl)carbamate (2k)**.

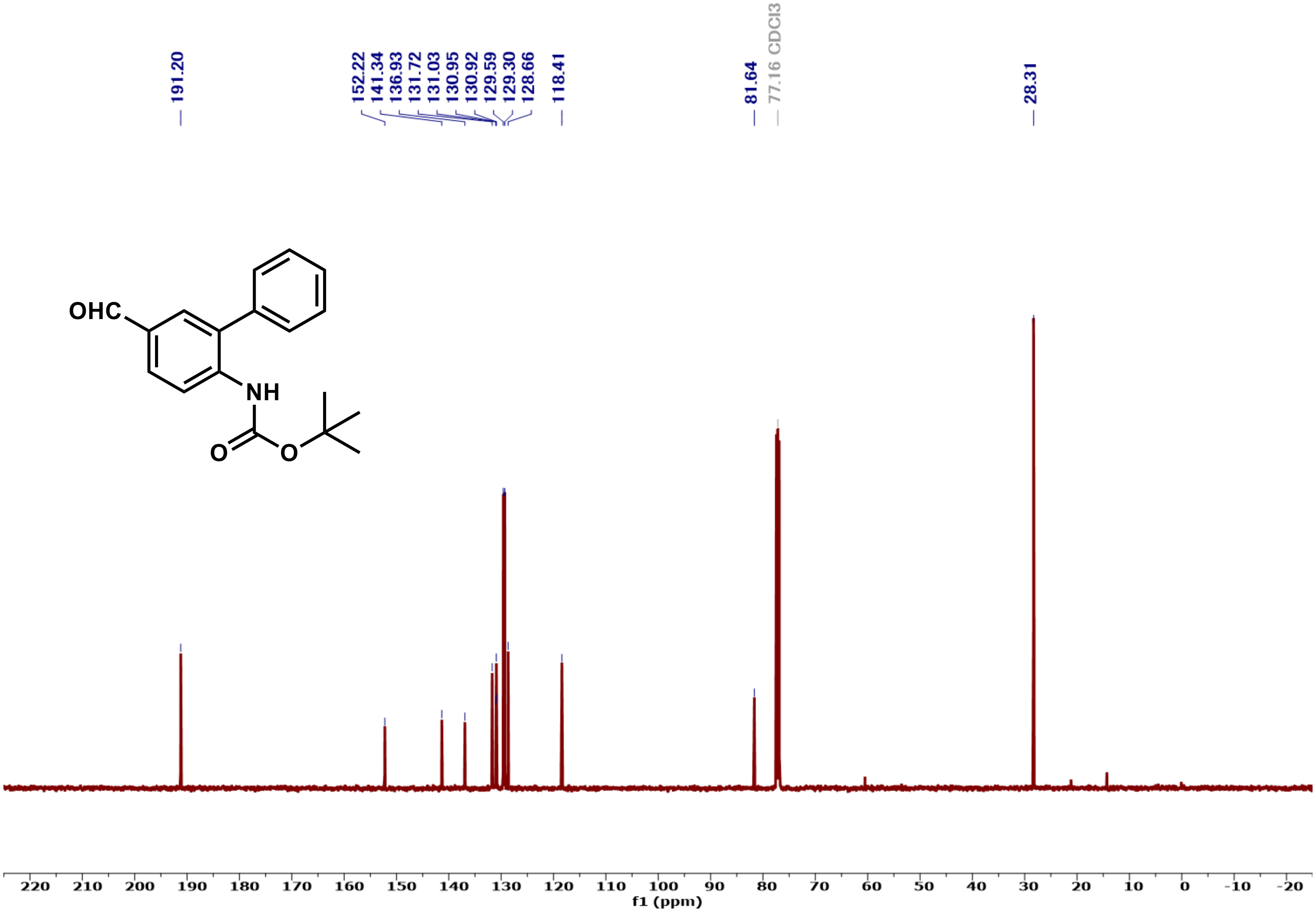

^{13}C NMR spectrum (126 MHz, $CDCl_3$, 25 °C) of ***tert*-butyl (5-formyl-[1,1'-biphenyl]-2-yl)carbamate (2k)**.

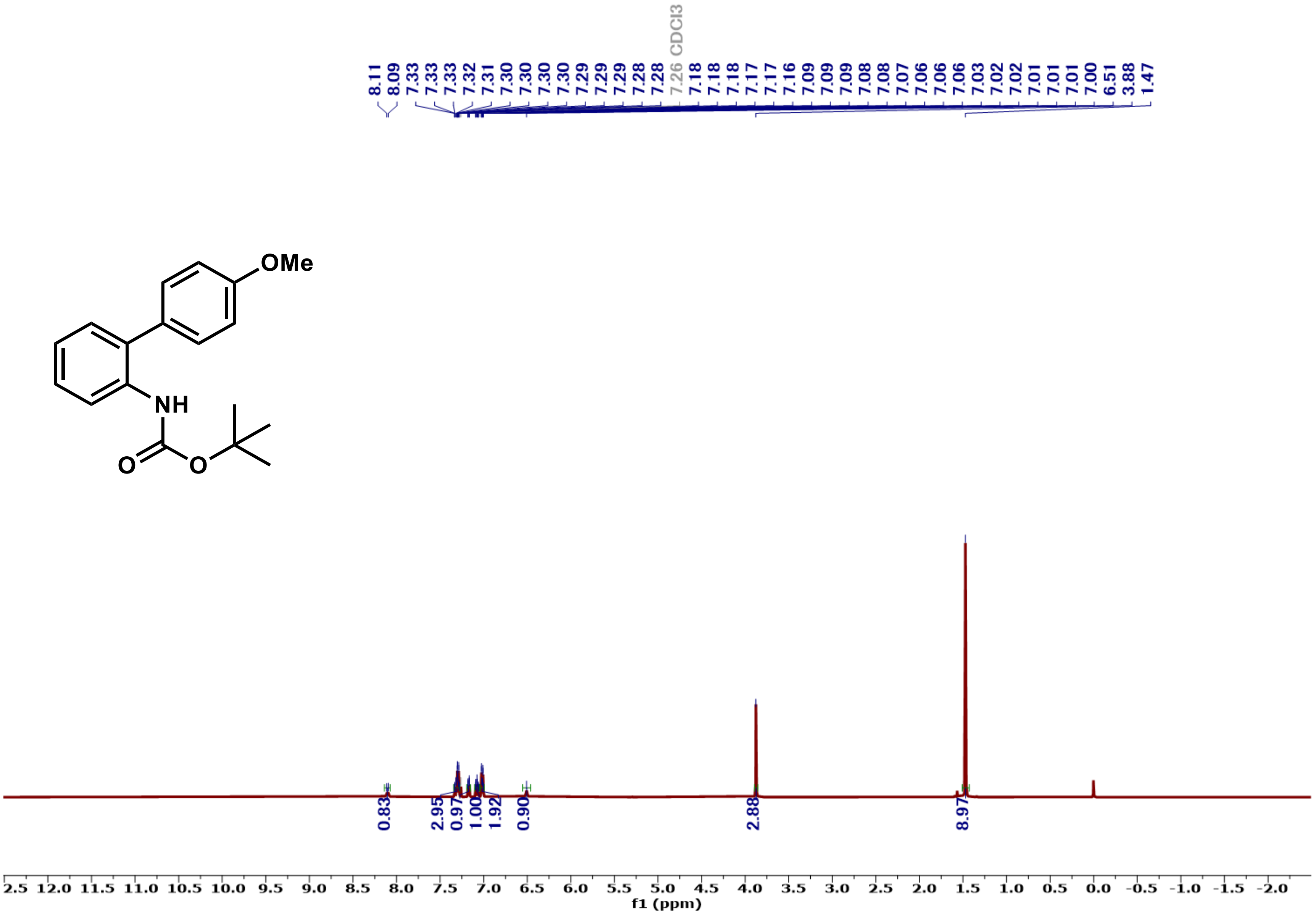

^{1}H NMR spectrum (500 MHz, $CDCl_3$, 25 °C) of ***tert*-butyl (4'-methoxy-[1,1'-biphenyl]-2-yl)carbamate (2l)**.

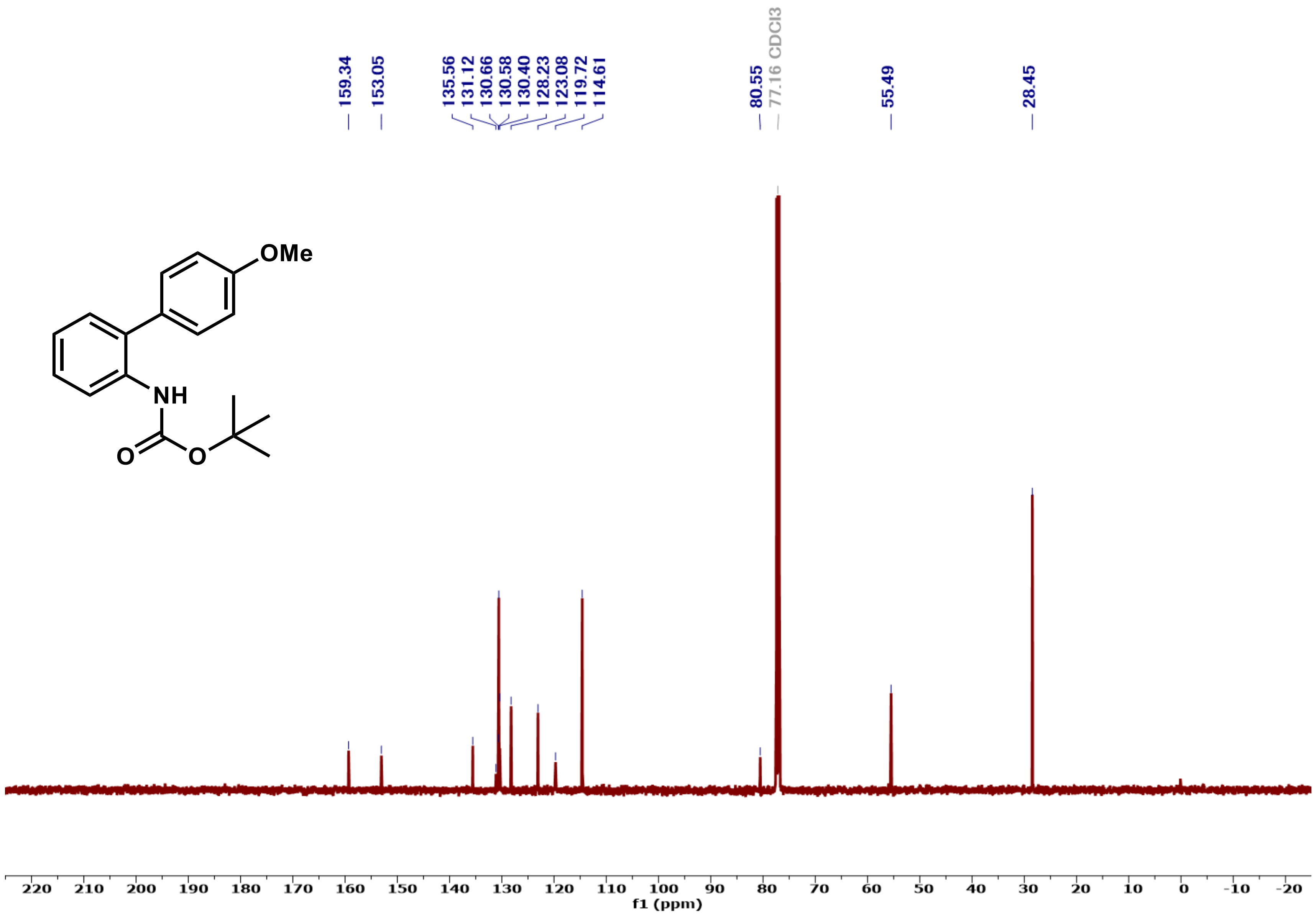

^{13}C NMR spectrum (126 MHz, $CDCl_3$, 25 °C) of ***tert*-butyl (4'-methoxy-[1,1'-biphenyl]-2-yl)carbamate (2l)**.

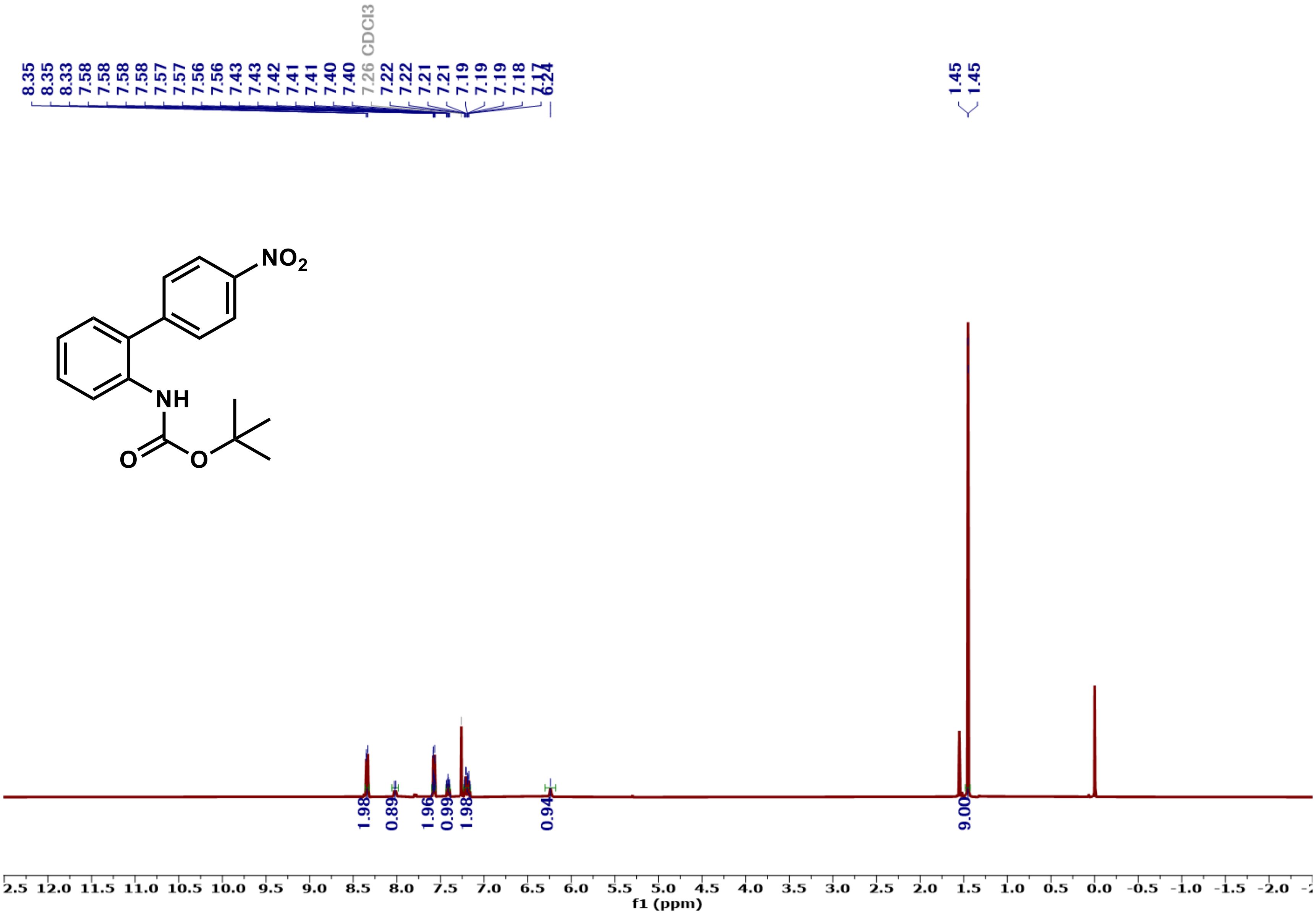

^{1}H NMR spectrum (500 MHz, $CDCl_3$, 25 °C) of ***tert*-butyl (4'-nitro-[1,1'-biphenyl]-2-yl)carbamate (2m)**.

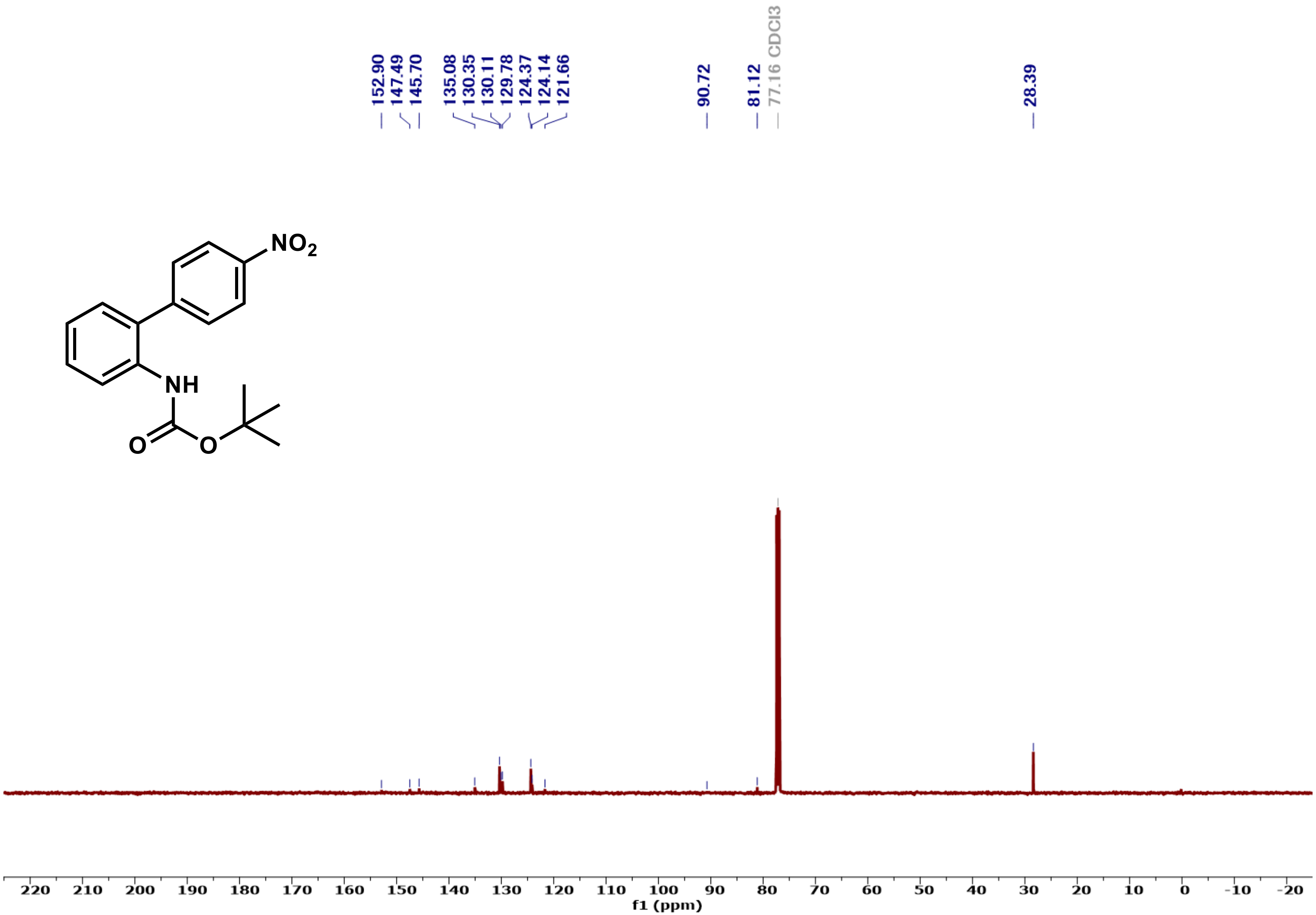

^{13}C NMR spectrum (126 MHz, $CDCl_3$, 25 °C) of ***tert*-butyl (4'-nitro-[1,1'-biphenyl]-2-yl)carbamate (2m)**.

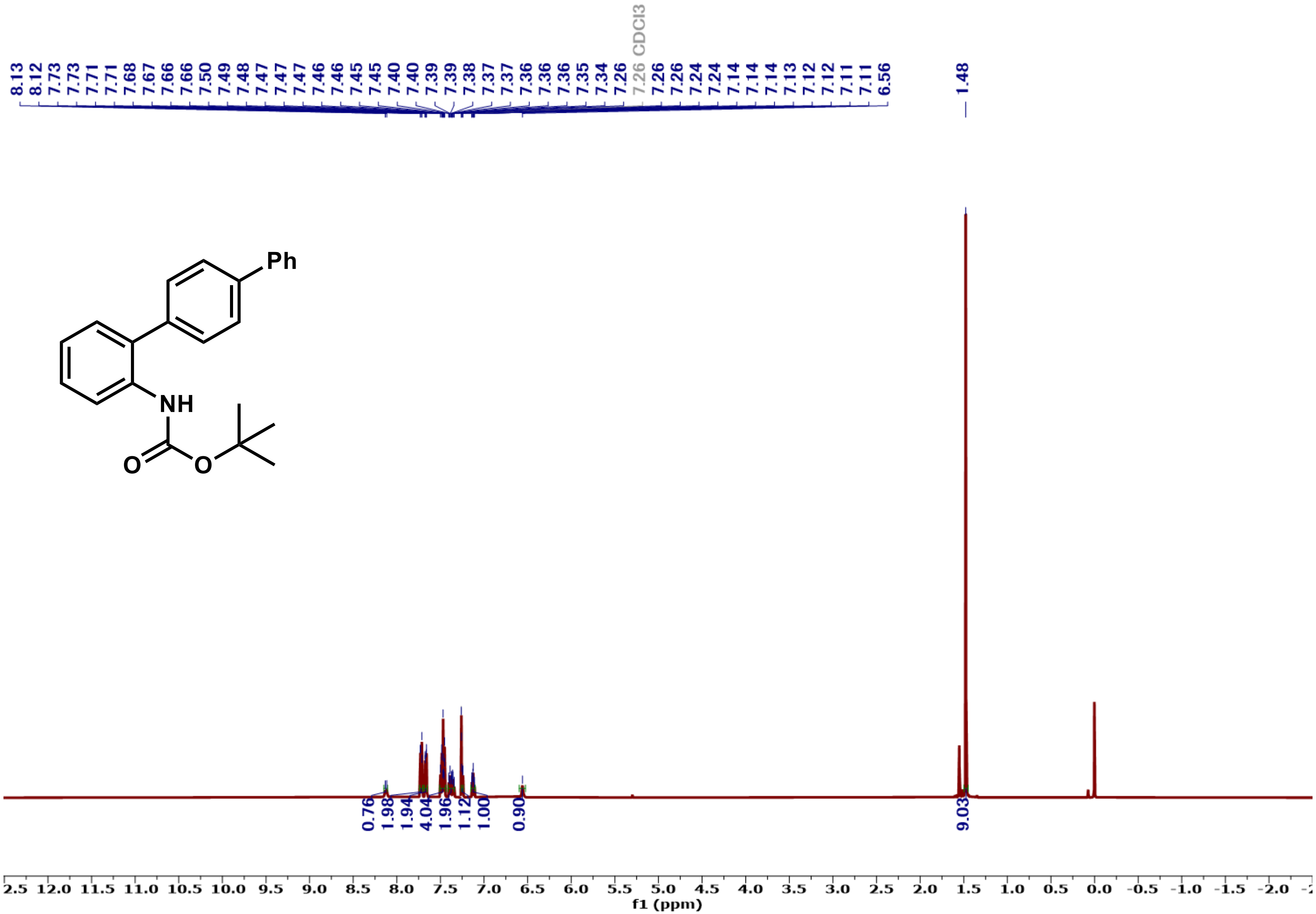

^{1}H NMR spectrum (500 MHz, $CDCl_3$, 25 °C) of ***tert*-butyl ([1,1':4',1''-terphenyl]-2-yl)carbamate (2n)**.

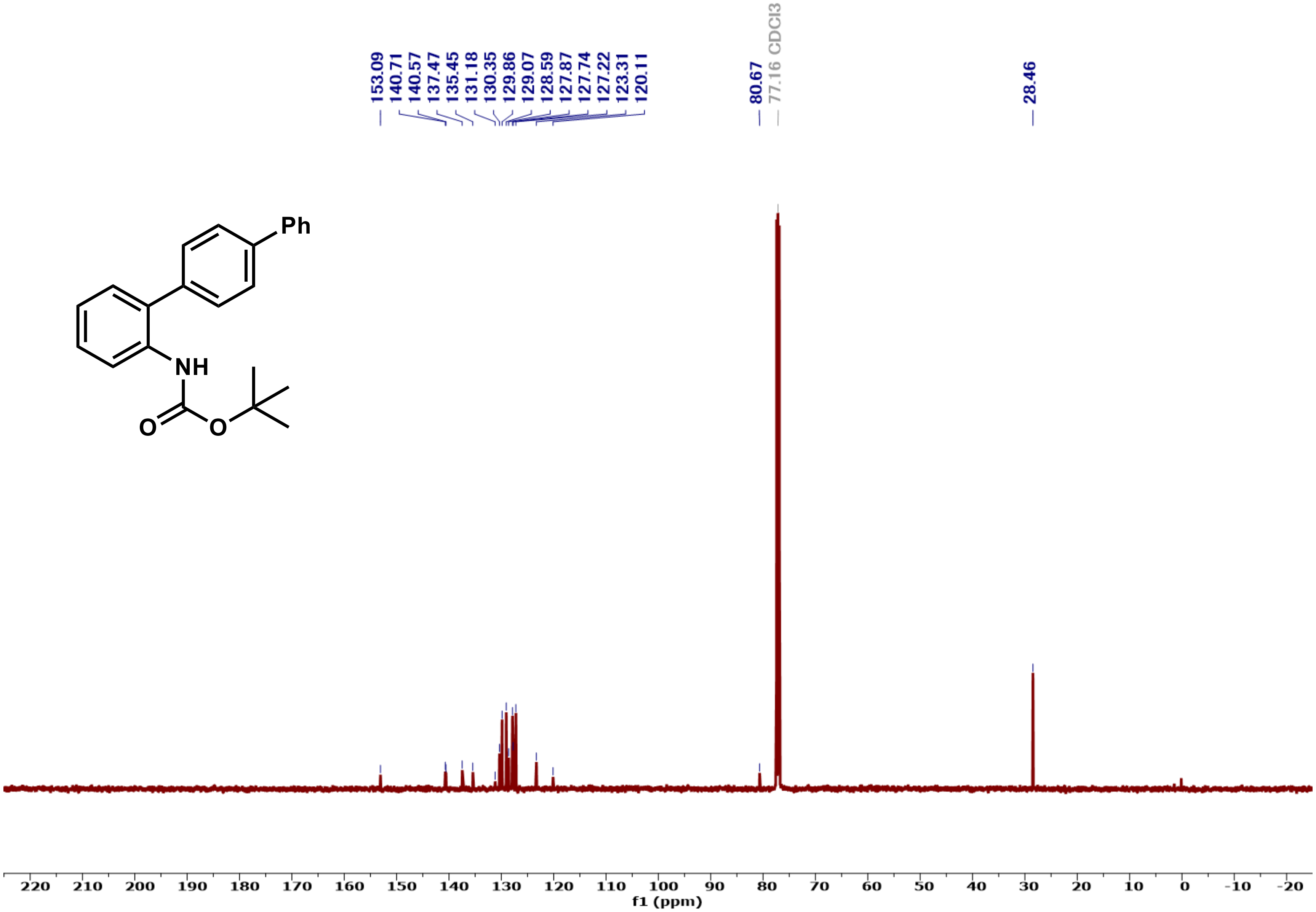

^{13}C NMR spectrum (126 MHz, $CDCl_3$, 25 °C) of ***tert*-butyl ([1,1':4',1''-terphenyl]-2-yl)carbamate (2n)**.

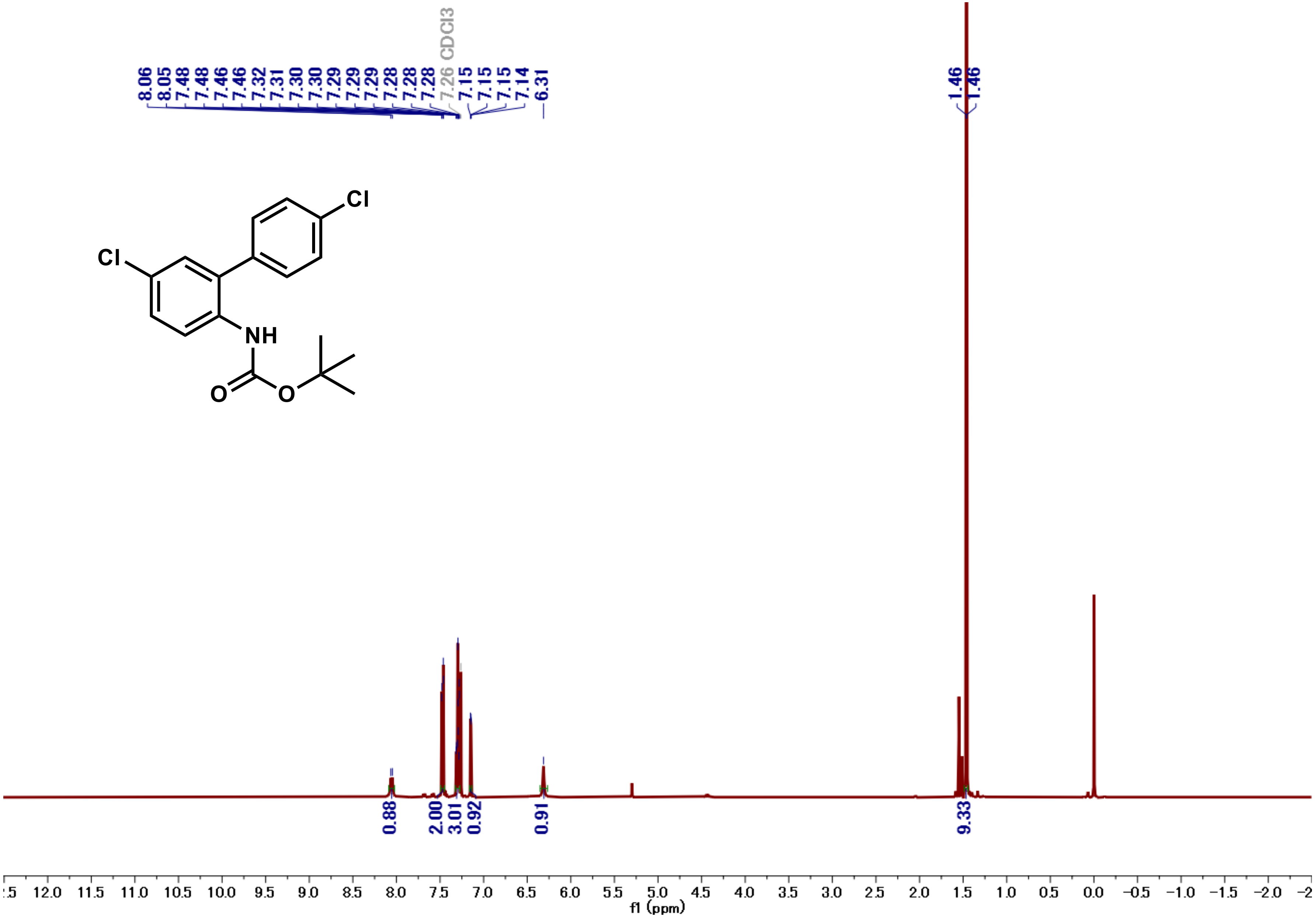

^{1}H NMR spectrum (500 MHz, $CDCl_3$, 25 °C) of ***tert*-butyl (4',5-chloro-[1,1'-biphenyl]-2-yl)carbamate (2o)**.

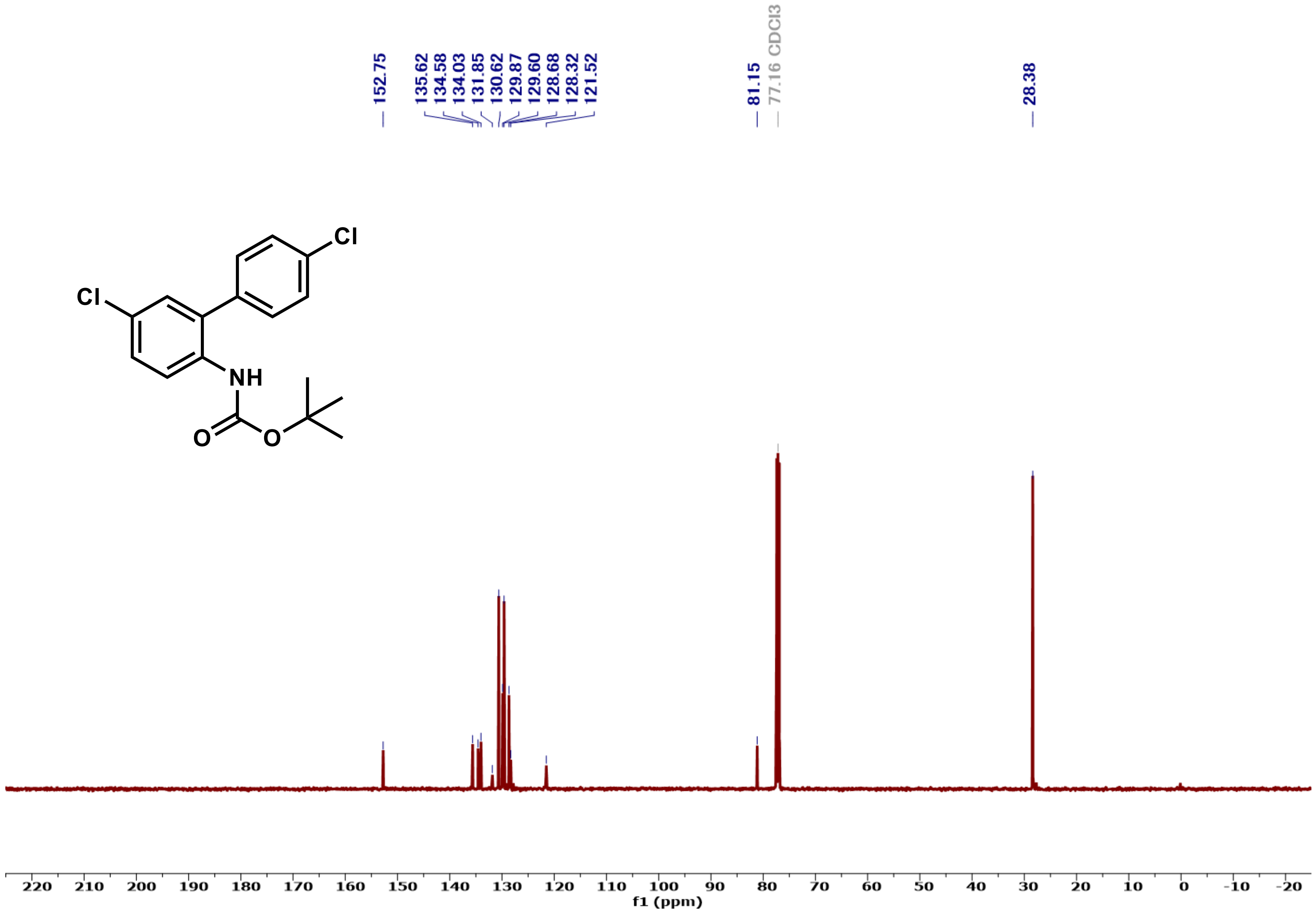

^{13}C NMR spectrum (126 MHz, $CDCl_3$, 25 °C) of ***tert*-butyl (4',5-chloro-[1,1'-biphenyl]-2-yl)carbamate (2o)**.

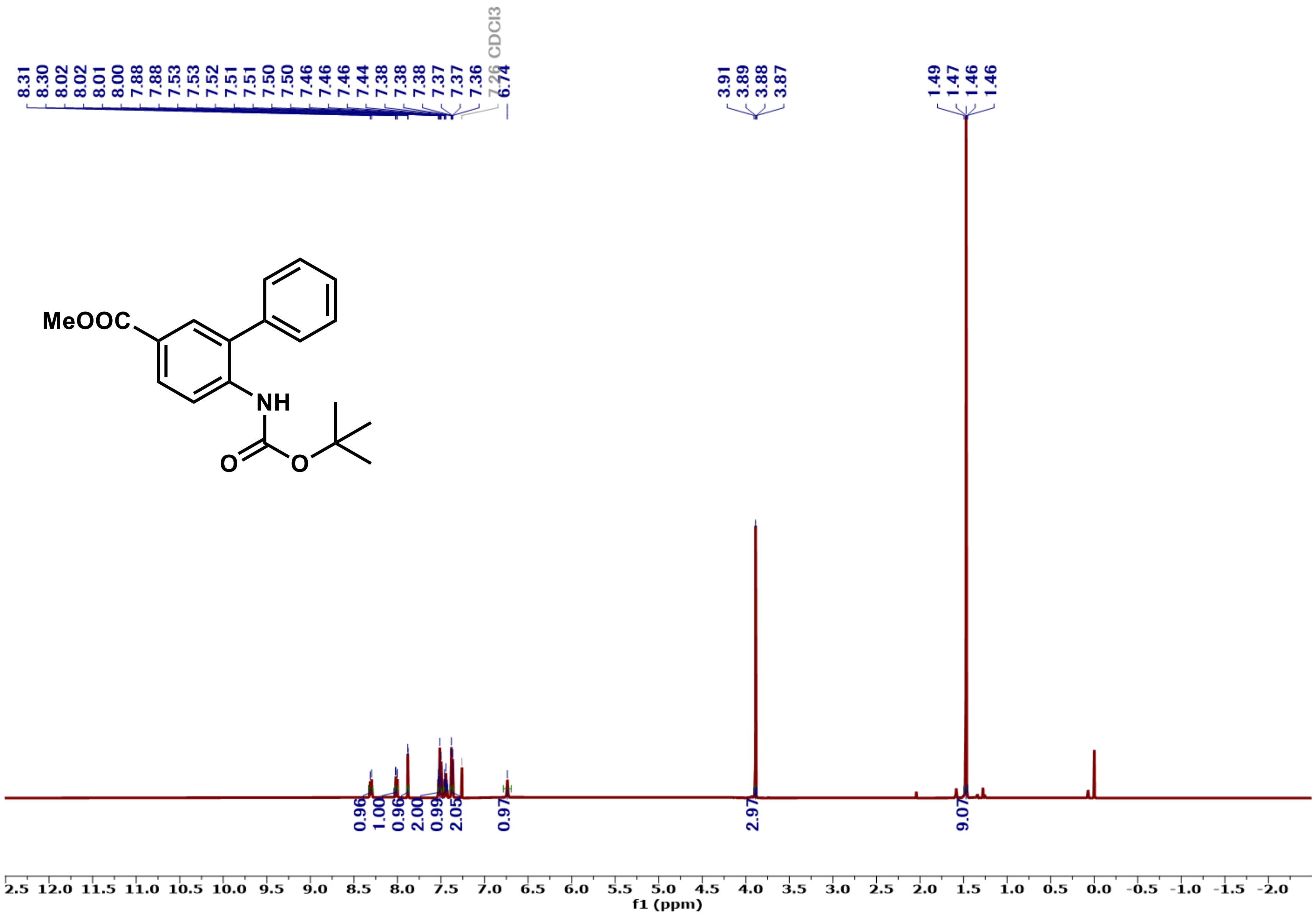

^{1}H NMR spectrum (500 MHz, $CDCl_3$, 25 °C) of **methyl 6-((*tert*-butoxycarbonyl)amino)-[1,1'-biphenyl]-3-carboxylate (2p)**.

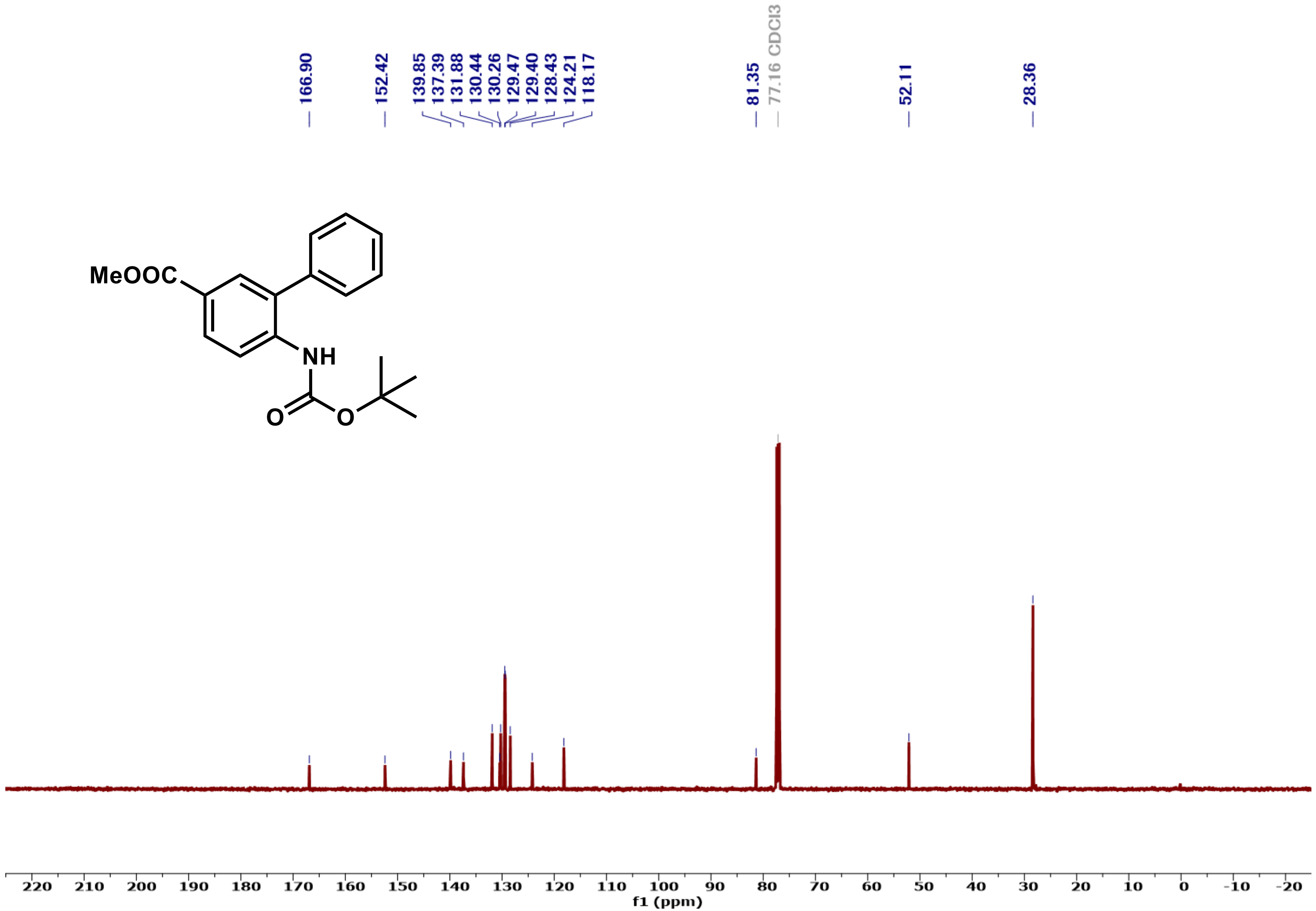

^{13}C NMR spectrum (126 MHz, $CDCl_3$, 25 °C) of **methyl 6-((*tert*-butoxycarbonyl)amino)-[1,1'-biphenyl]-3-carboxylate (2p)**.

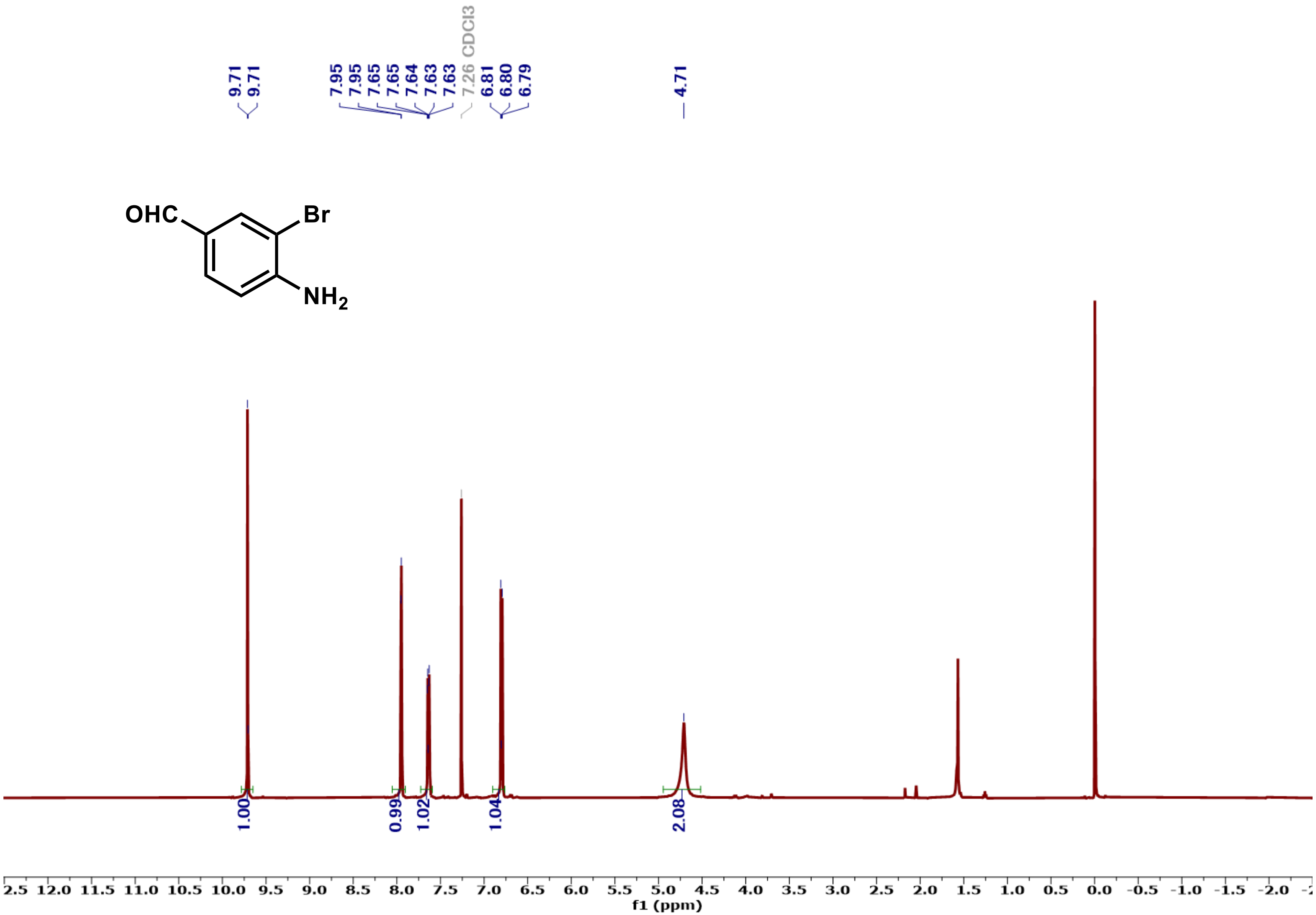

^{1}H NMR spectrum (500 MHz, $CDCl_3$, 25 °C) of **4-amino-3-bromobenzaldehyde**.

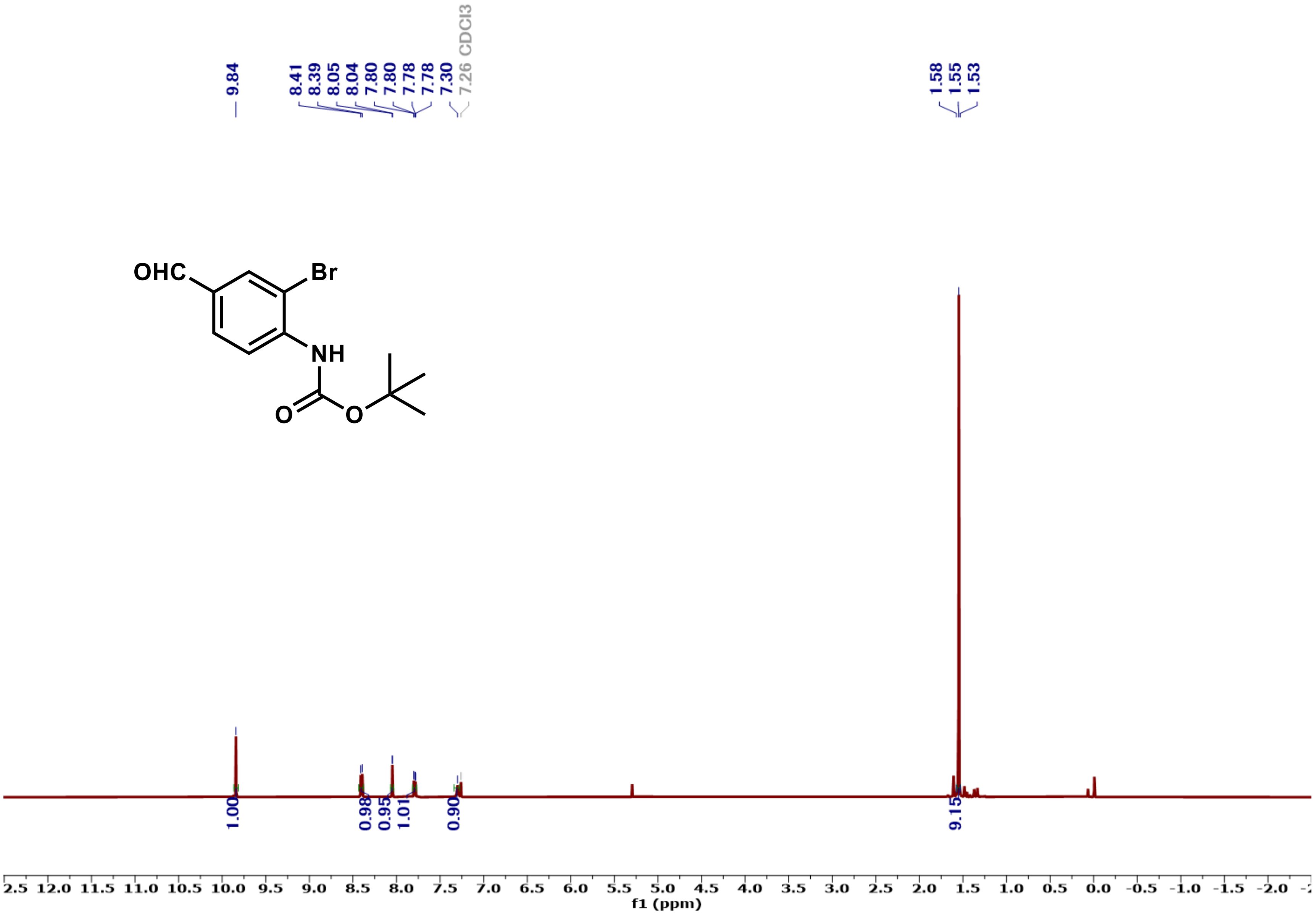

^{1}H NMR spectrum (500 MHz, $CDCl_3$, 25 °C) of ***tert*-butyl (2-bromo-4-formylphenyl)carbamate**.

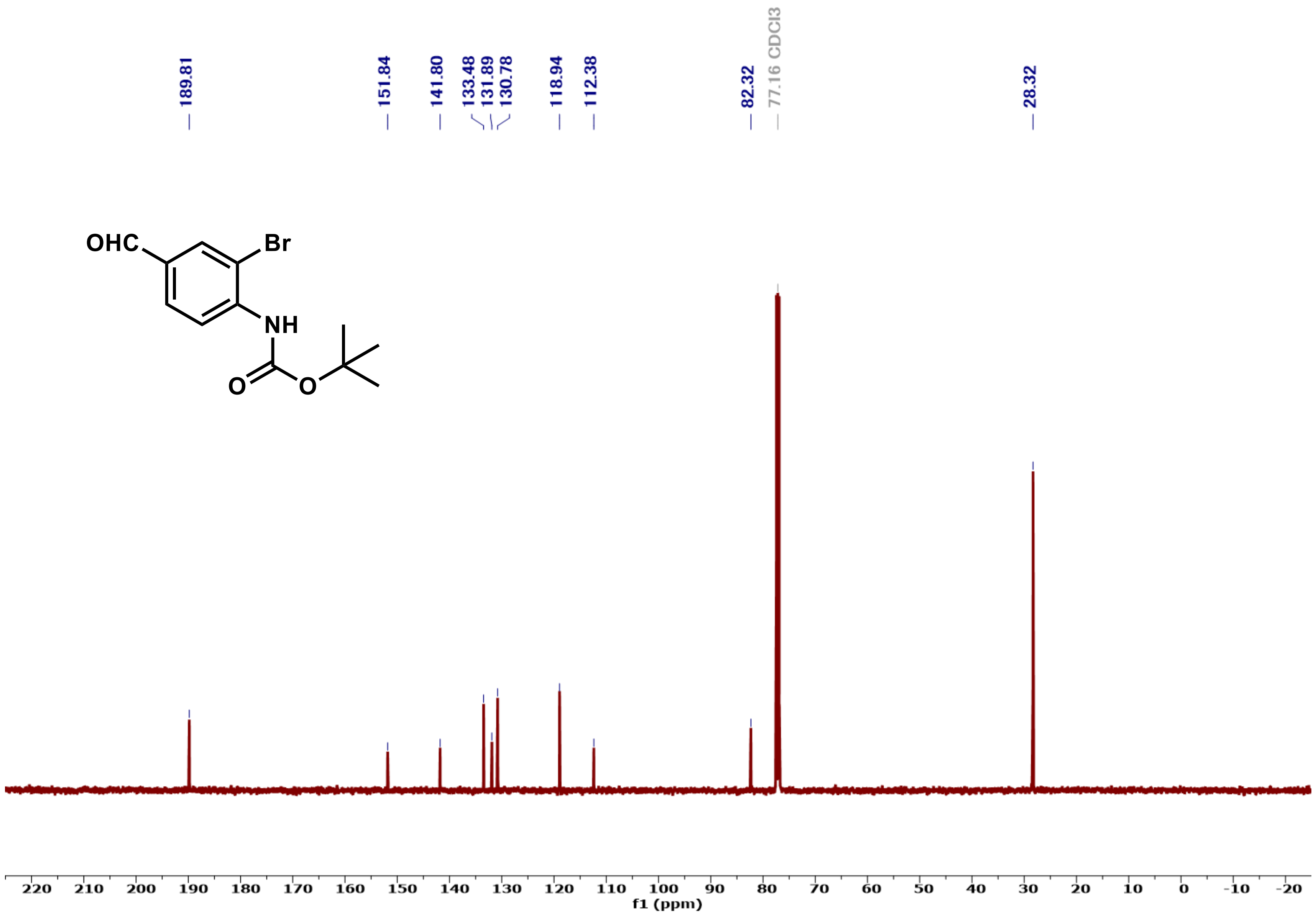

^{13}C NMR spectrum (126 MHz, $CDCl_3$, 25 °C) of ***tert*-butyl (2-bromo-4-formylphenyl)carbamate**.

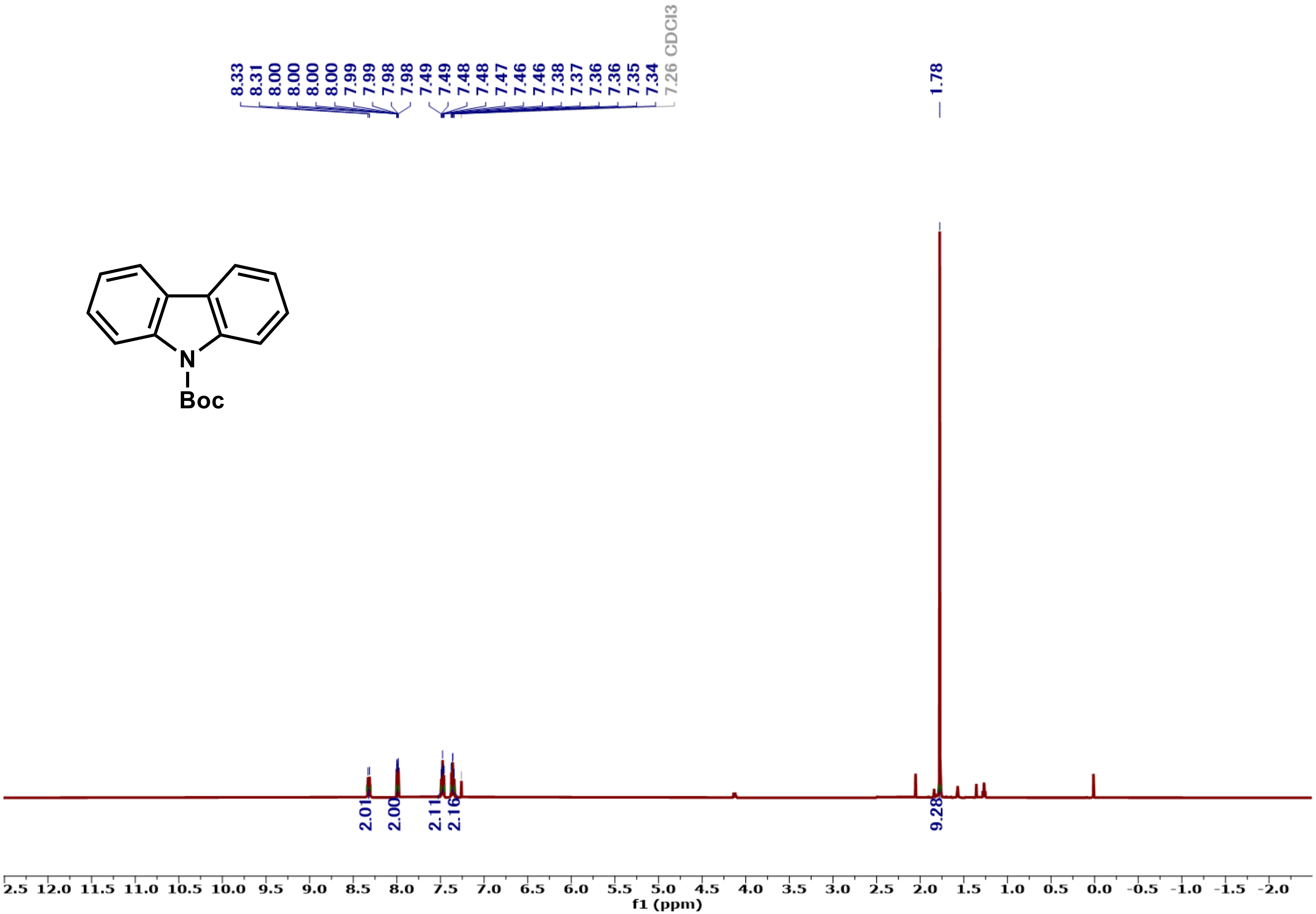

^{1}H NMR spectrum (500 MHz, $CDCl_3$, 25 °C) of ***tert*****-butyl 9*****H*****-carbazole-9-carboxylate (3c)**.

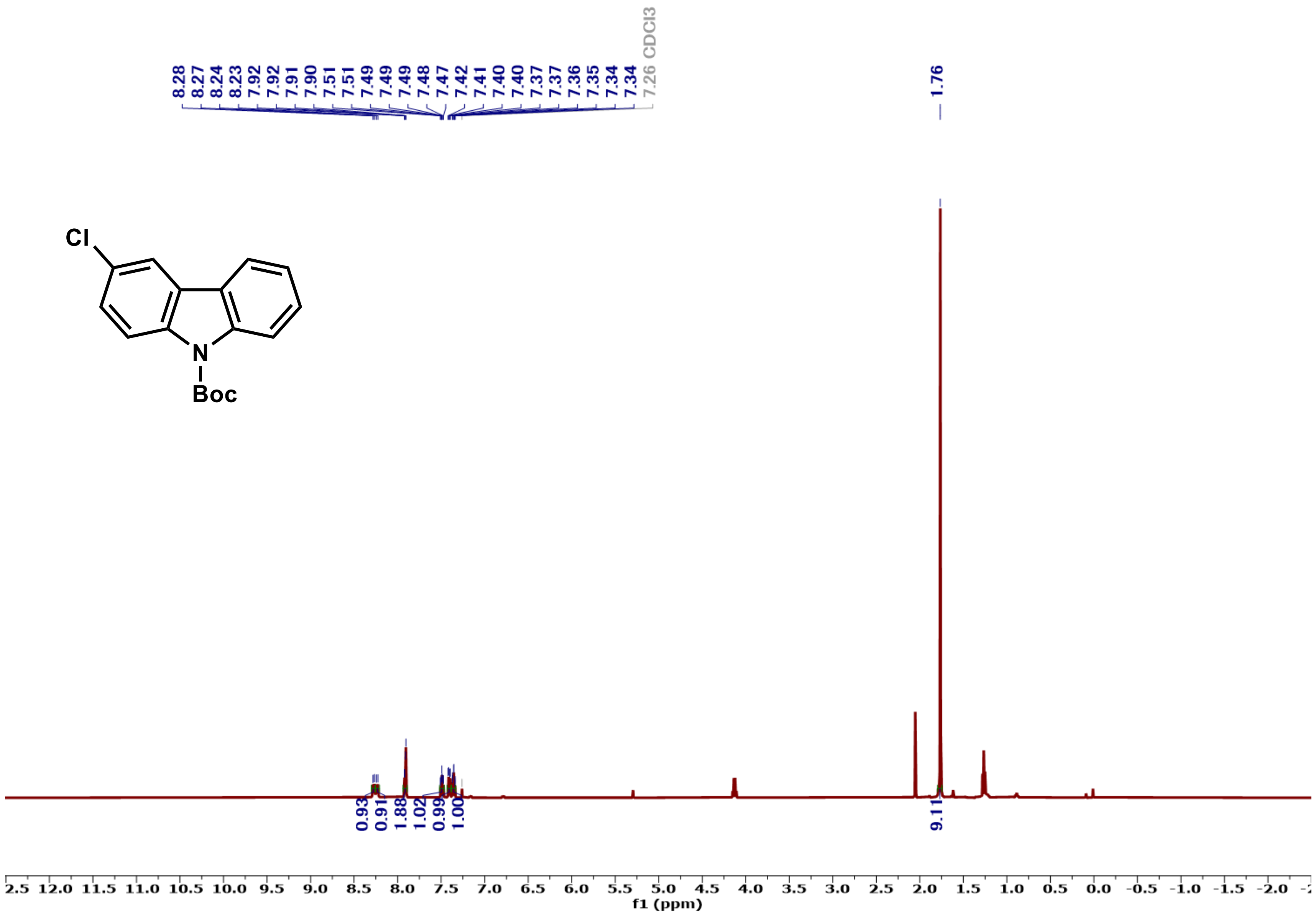

^{1}H NMR spectrum (500 MHz, $CDCl_3$, 25 °C) of ***tert*-butyl 3-chloro-9*H*-carbazole-9-carboxylate (3e)**.

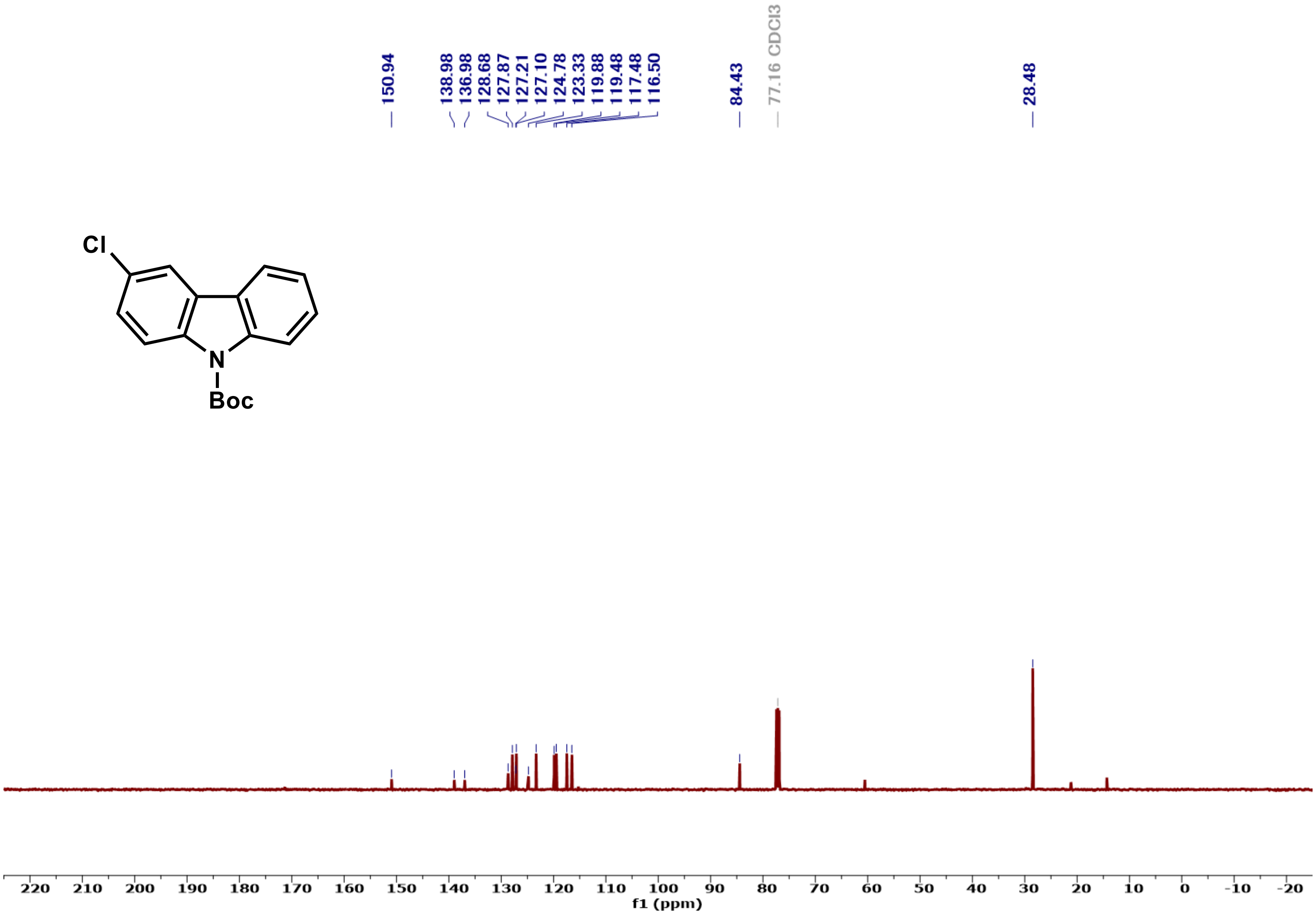

^{13}C NMR spectrum (126 MHz, $CDCl_3$, 25 °C) of ***tert*-butyl 3-chloro-9*H*-carbazole-9-carboxylate (3e)**.

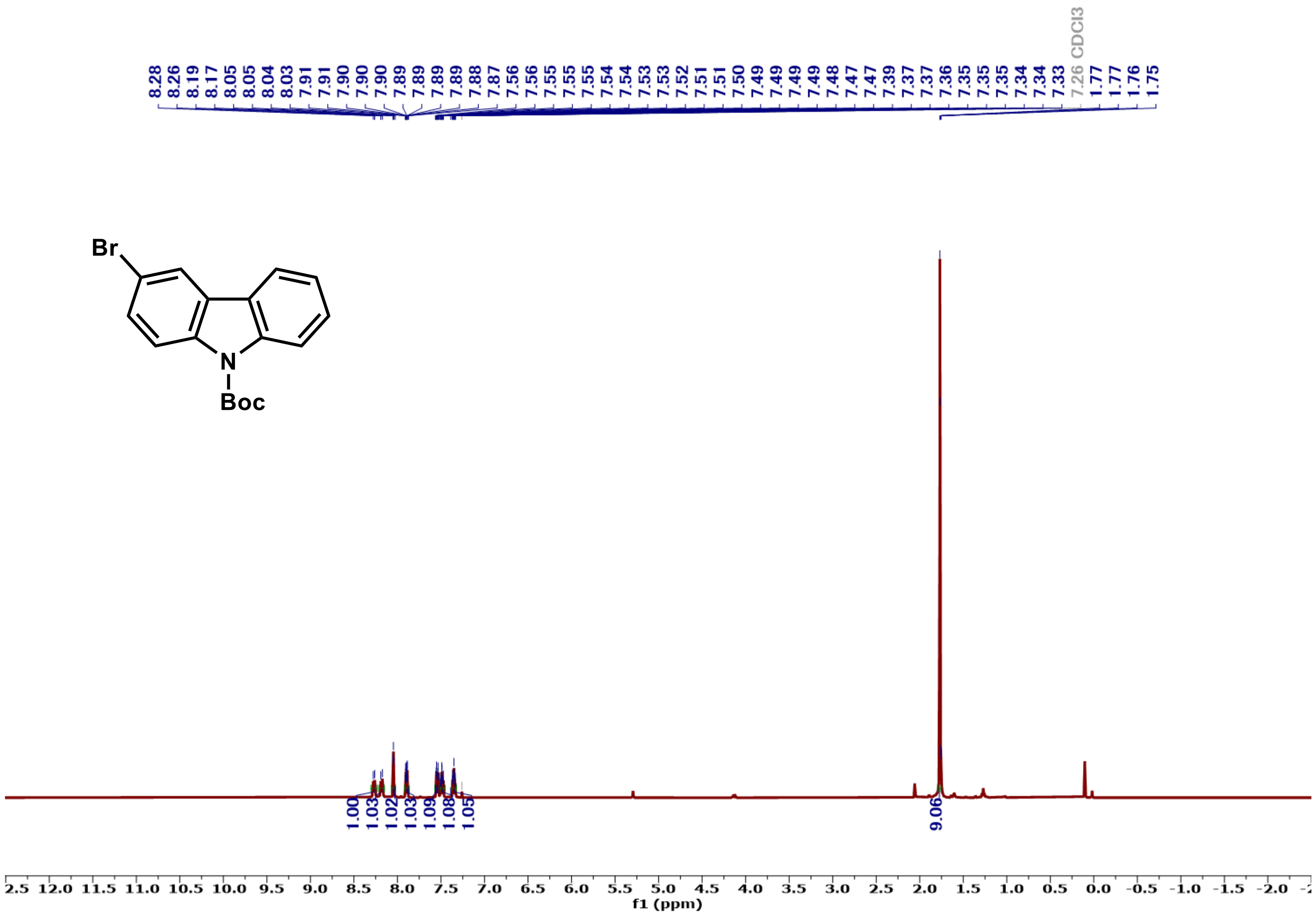

^{1}H NMR spectrum (500 MHz, $CDCl_3$, 25 °C) of ***tert*-butyl 3-bromo-9*H*-carbazole-9-carboxylate (3f)**.

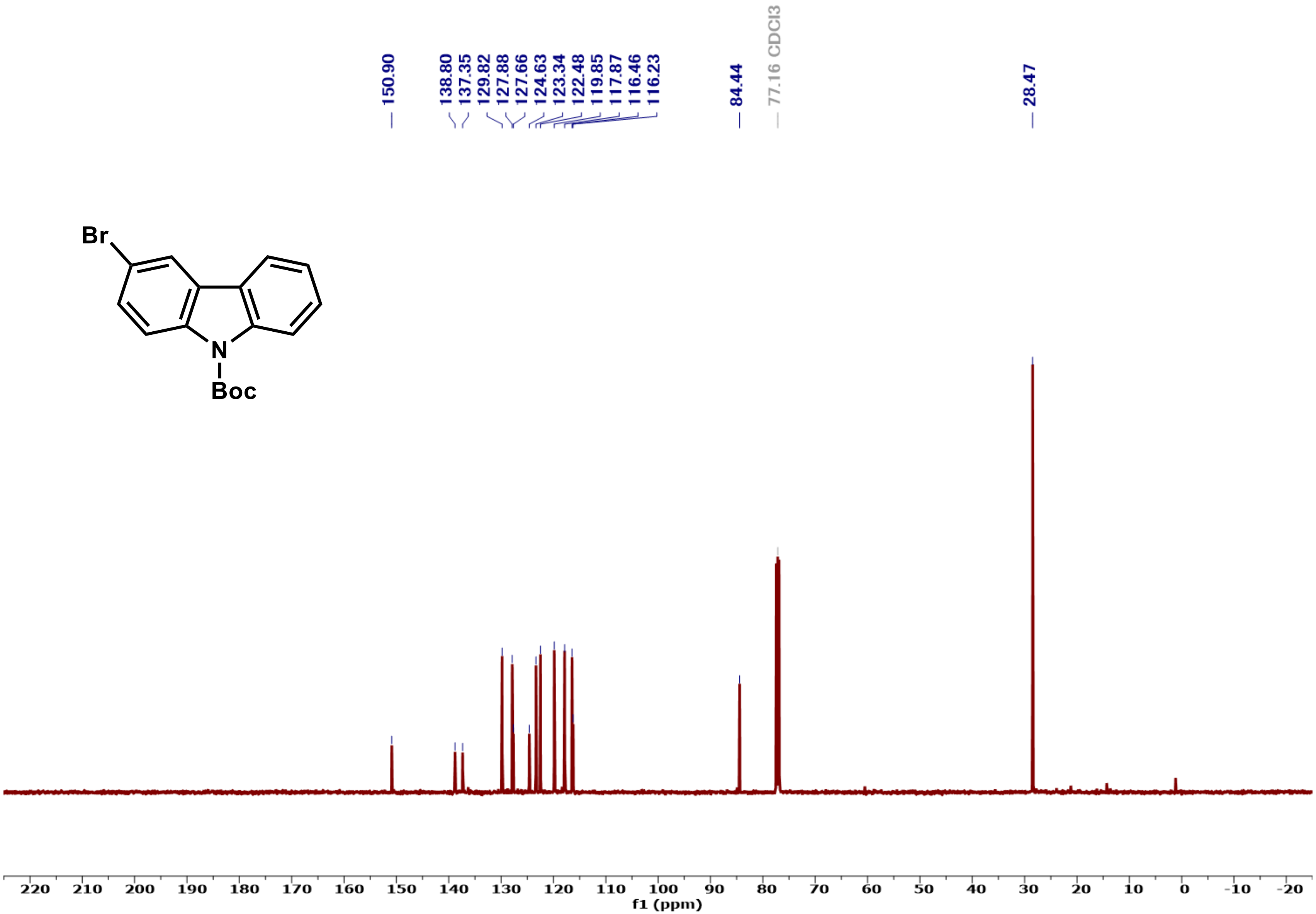

^{13}C NMR spectrum (126 MHz, $CDCl_3$, 25 °C) of ***tert*-butyl 3-bromo-9*H*-carbazole-9-carboxylate (3f)**.

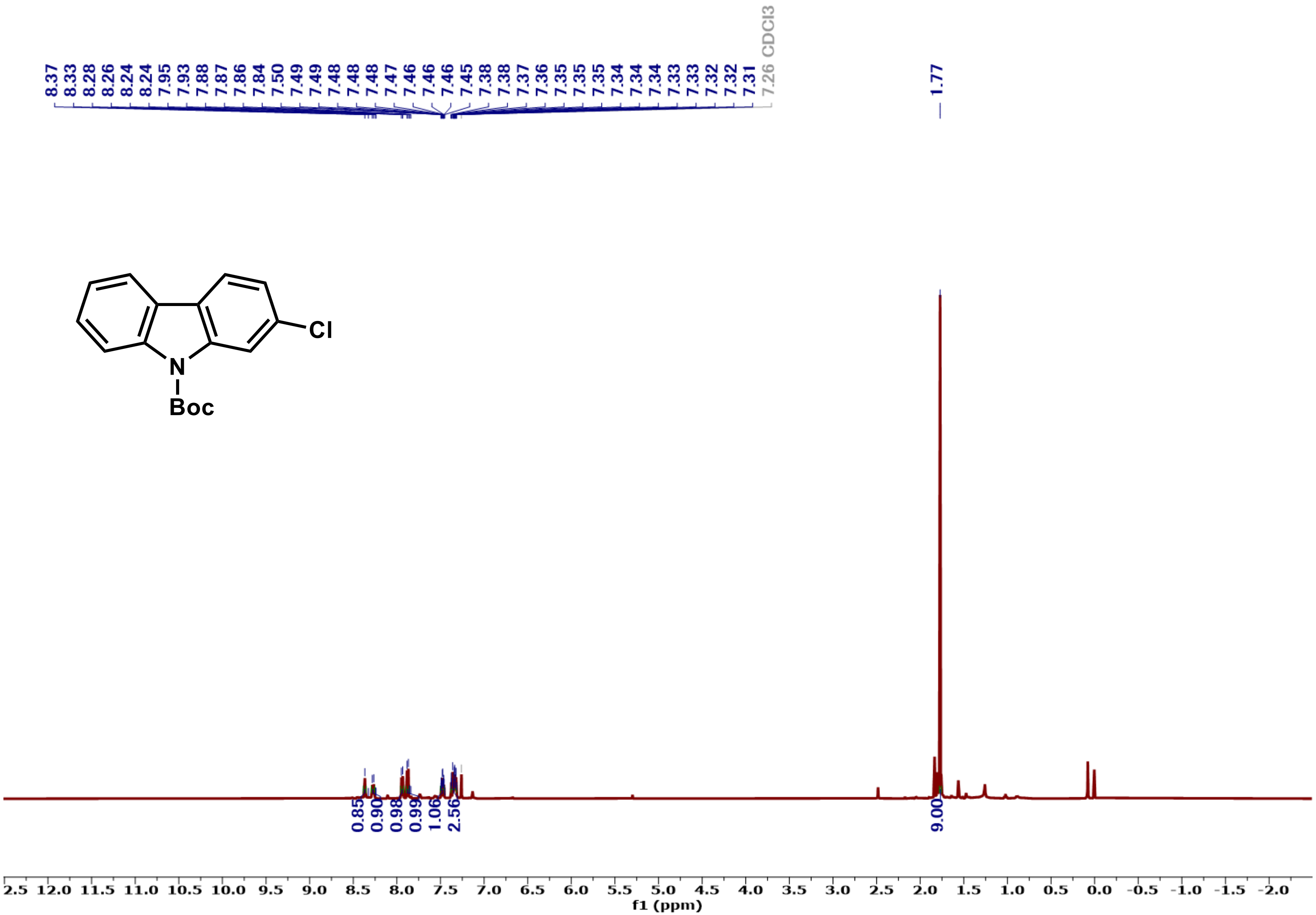

^{1}H NMR spectrum (500 MHz, $CDCl_3$, 25 °C) of ***tert*-butyl 2-chloro-9*H*-carbazole-9-carboxylate (3g)**.

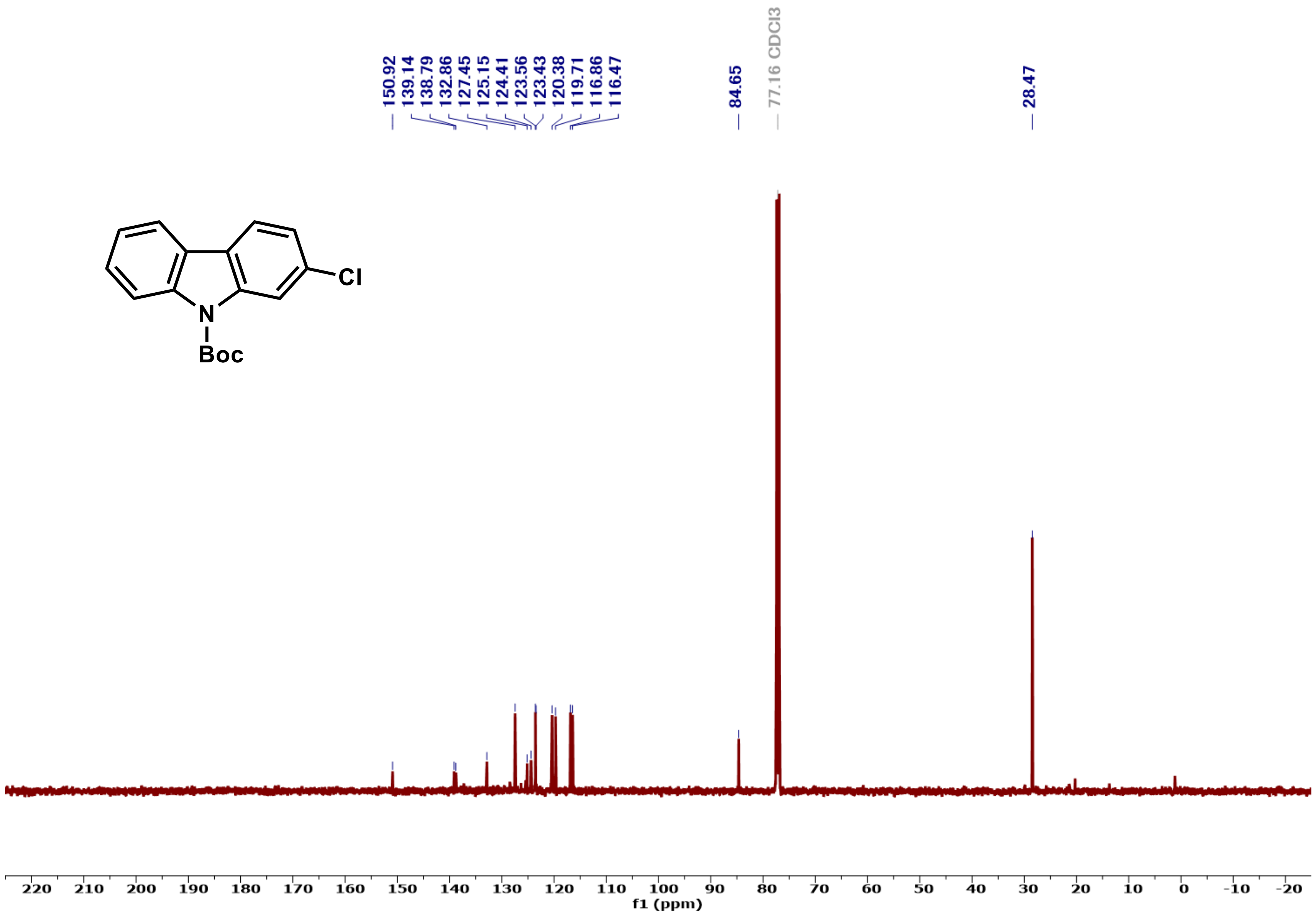

^{13}C NMR spectrum (126 MHz, $CDCl_3$, 25 °C) of ***tert*-butyl 2-chloro-9*H*-carbazole-9-carboxylate (3g)**.

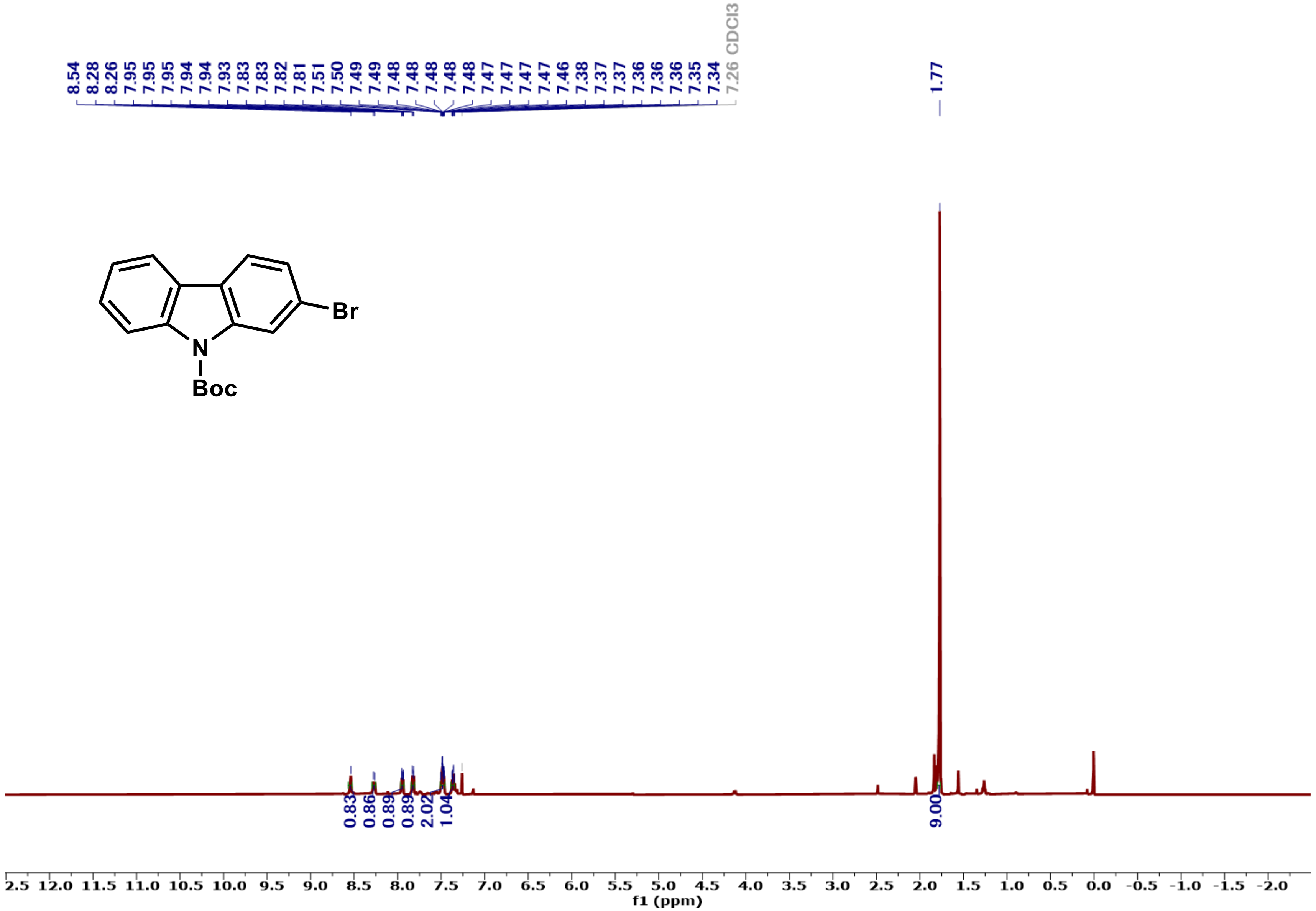

^{1}H NMR spectrum (500 MHz, $CDCl_3$, 25 °C) of ***tert*-butyl 2-bromo-9*H*-carbazole-9-carboxylate (3h)**.

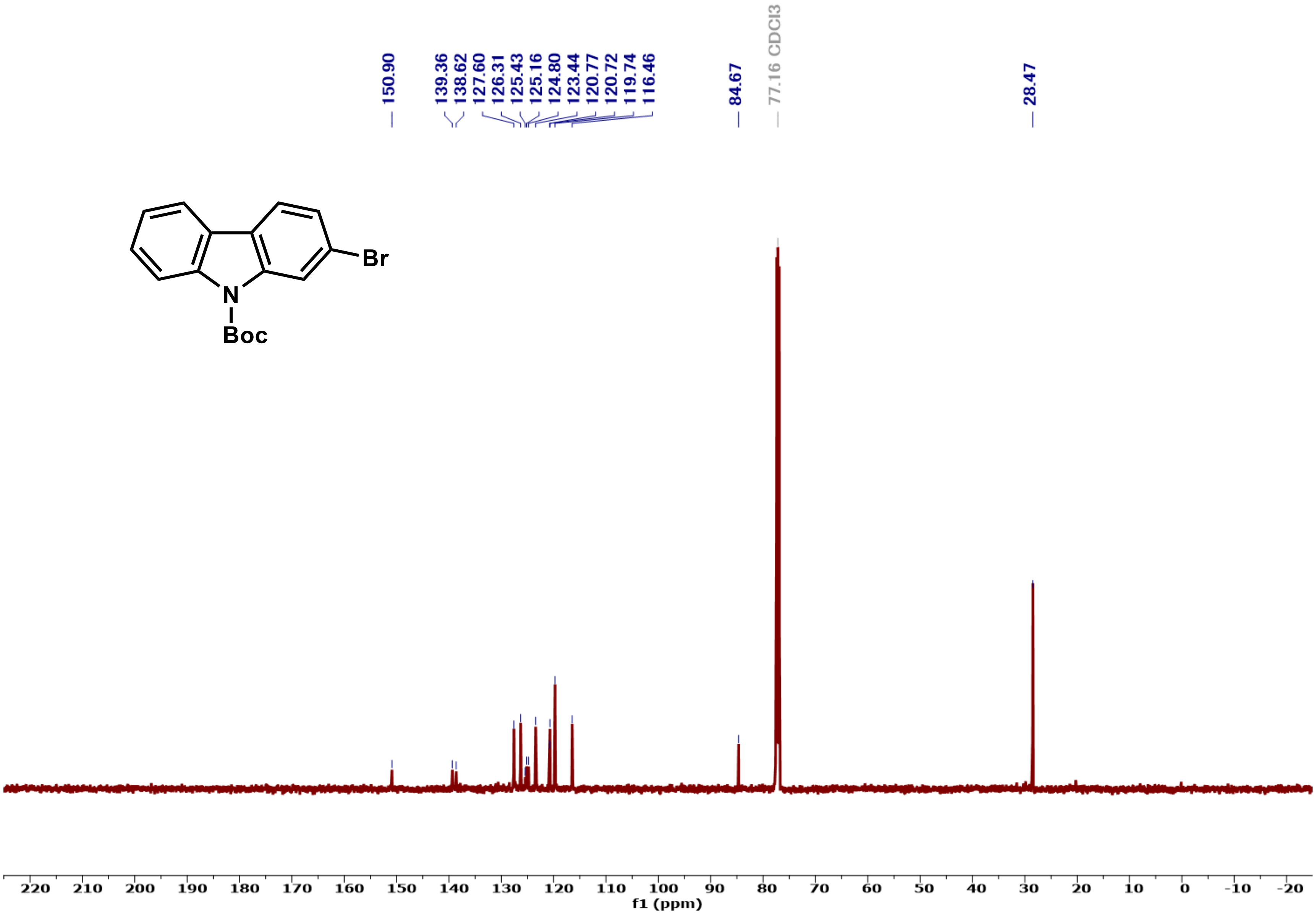

^{13}C NMR spectrum (126 MHz, $CDCl_3$, 25 °C) of ***tert*-butyl 2-bromo-9*H*-carbazole-9-carboxylate (3h)**.

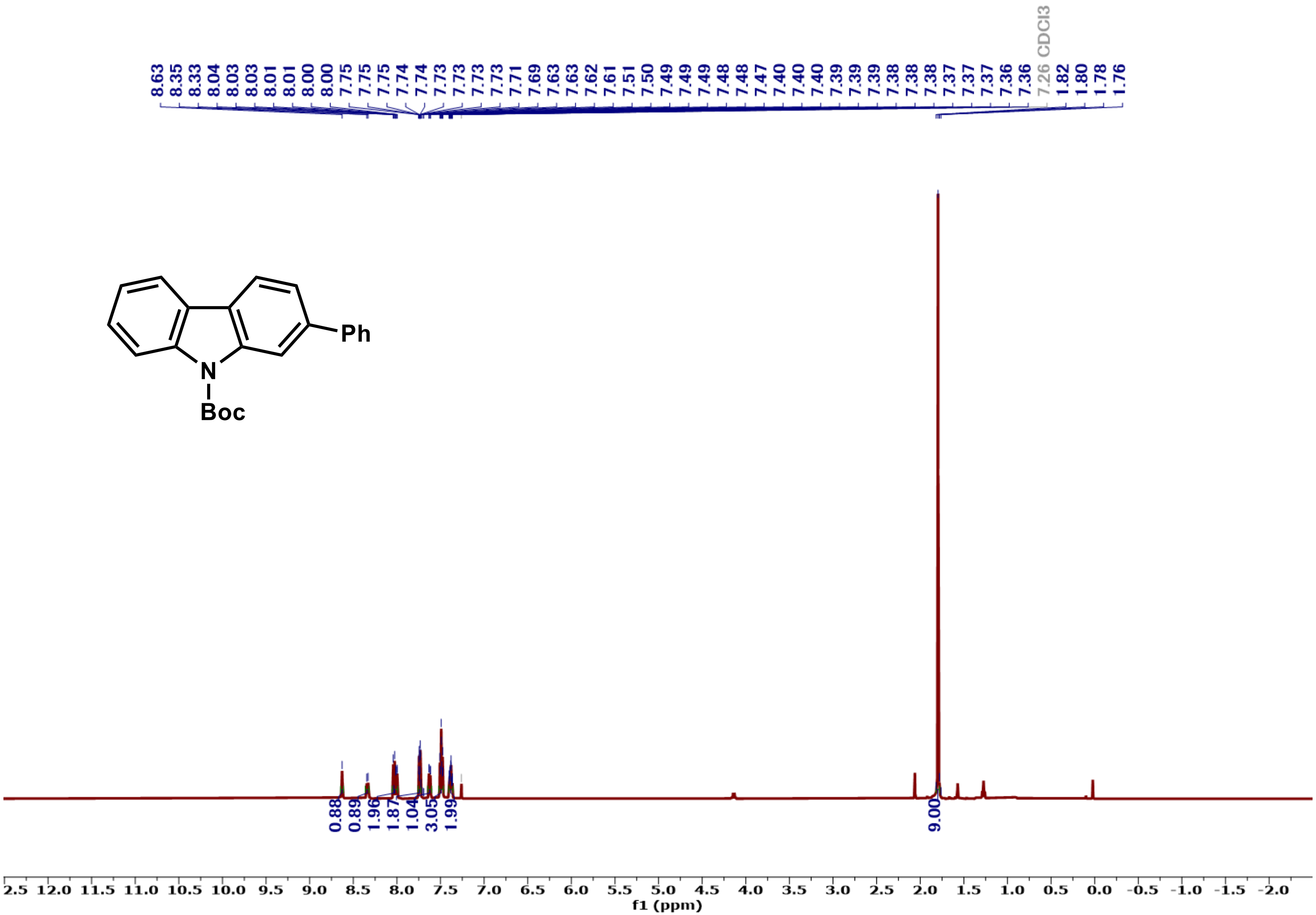

^{1}H NMR spectrum (500 MHz, $CDCl_3$, 25 °C) of ***tert*-butyl 2-phenyl-9*H*-carbazole-9-carboxylate (3n)**.

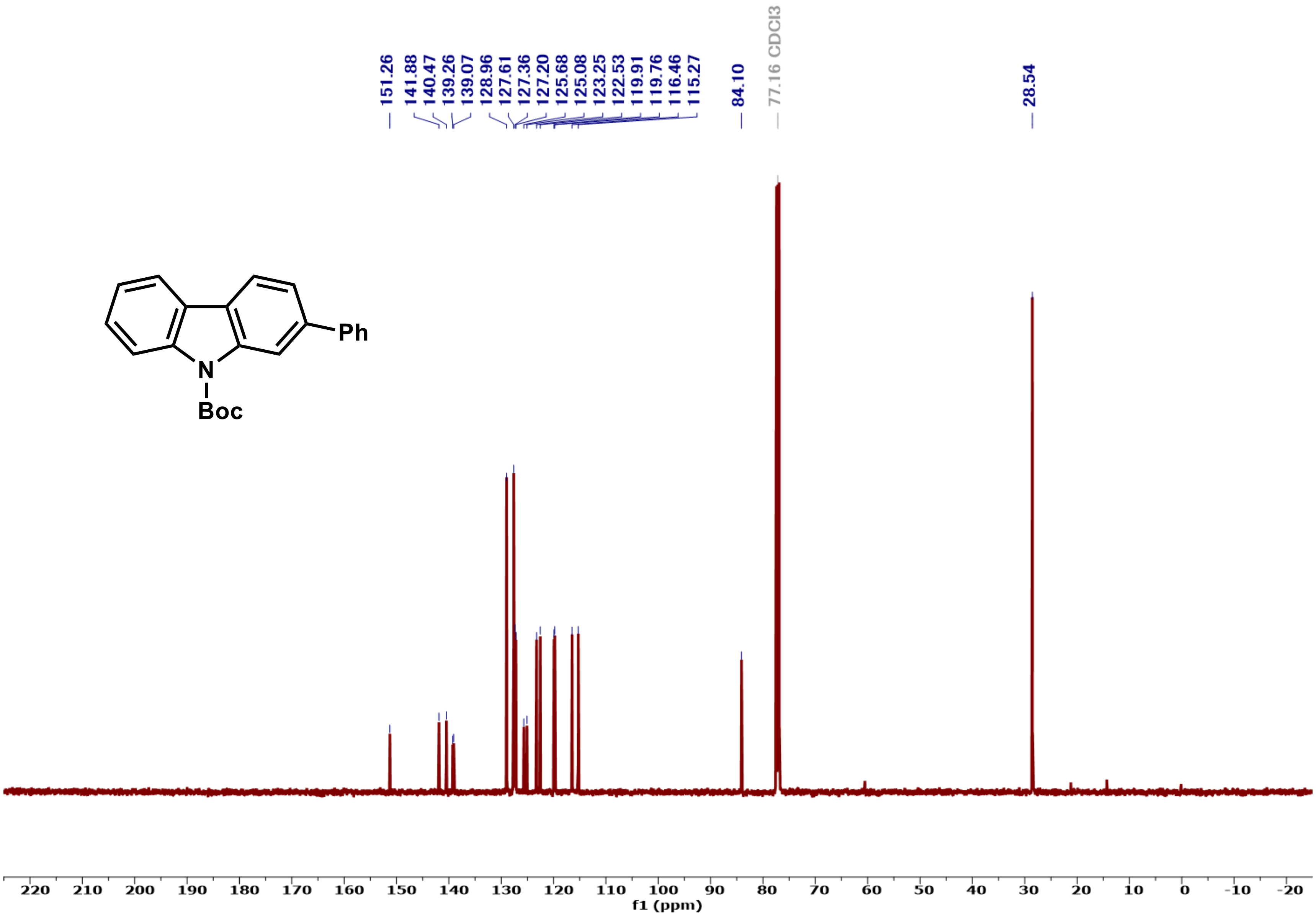

^{13}C NMR spectrum (126 MHz, $CDCl_3$, 25 °C) of ***tert*-butyl 2-phenyl-9*H*-carbazole-9-carboxylate (3n)**.

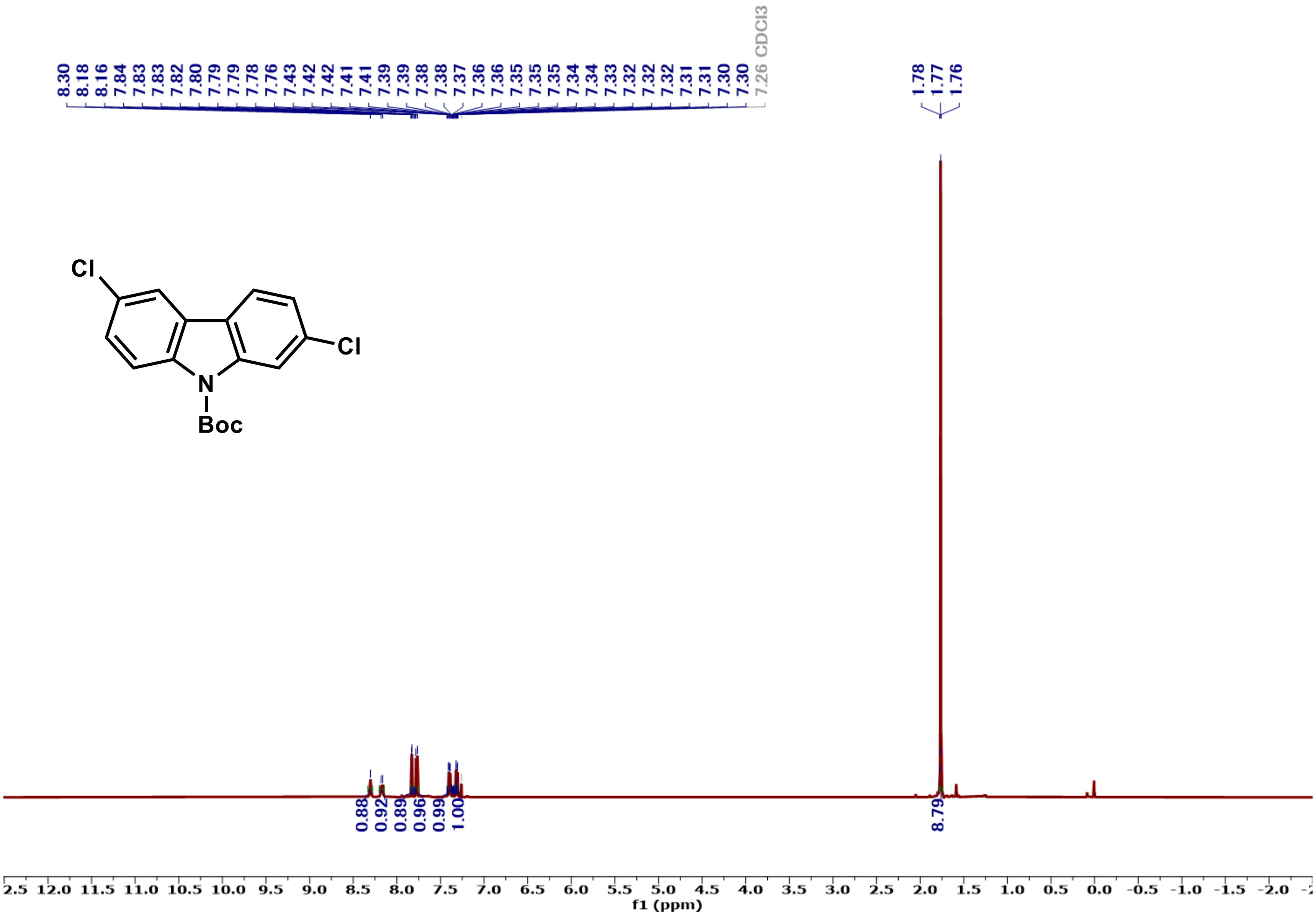

^{1}H NMR spectrum (500 MHz, $CDCl_3$, 25 °C) of ***tert*-butyl 2,6-dichloro-9*H*-carbazole-9-carboxylate (3o)**.

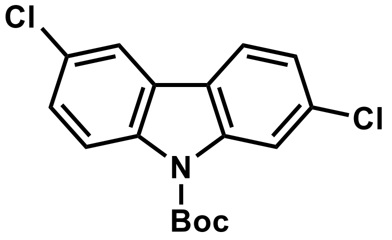

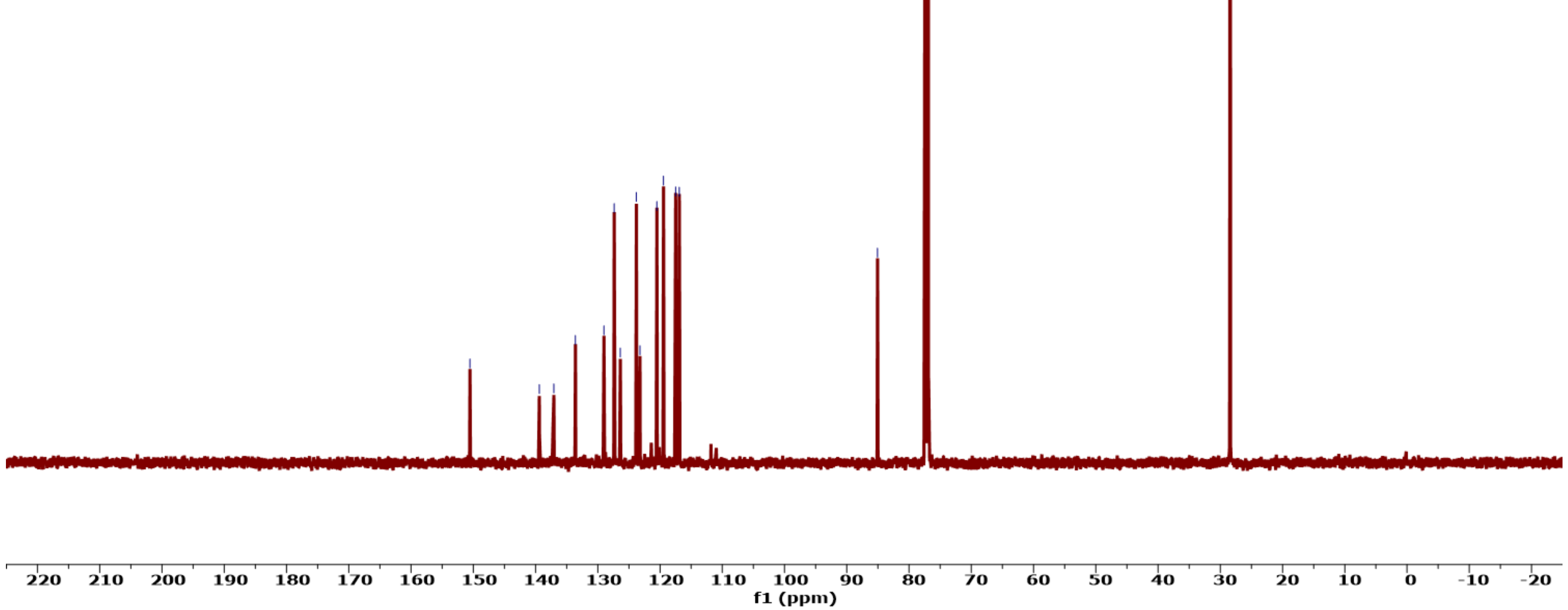

^{13}C NMR spectrum (126 MHz, $CDCl_3$, 25 °C) of ***tert*-butyl 2,6-dichloro-9*H*-carbazole-9-carboxylate (3o)**.

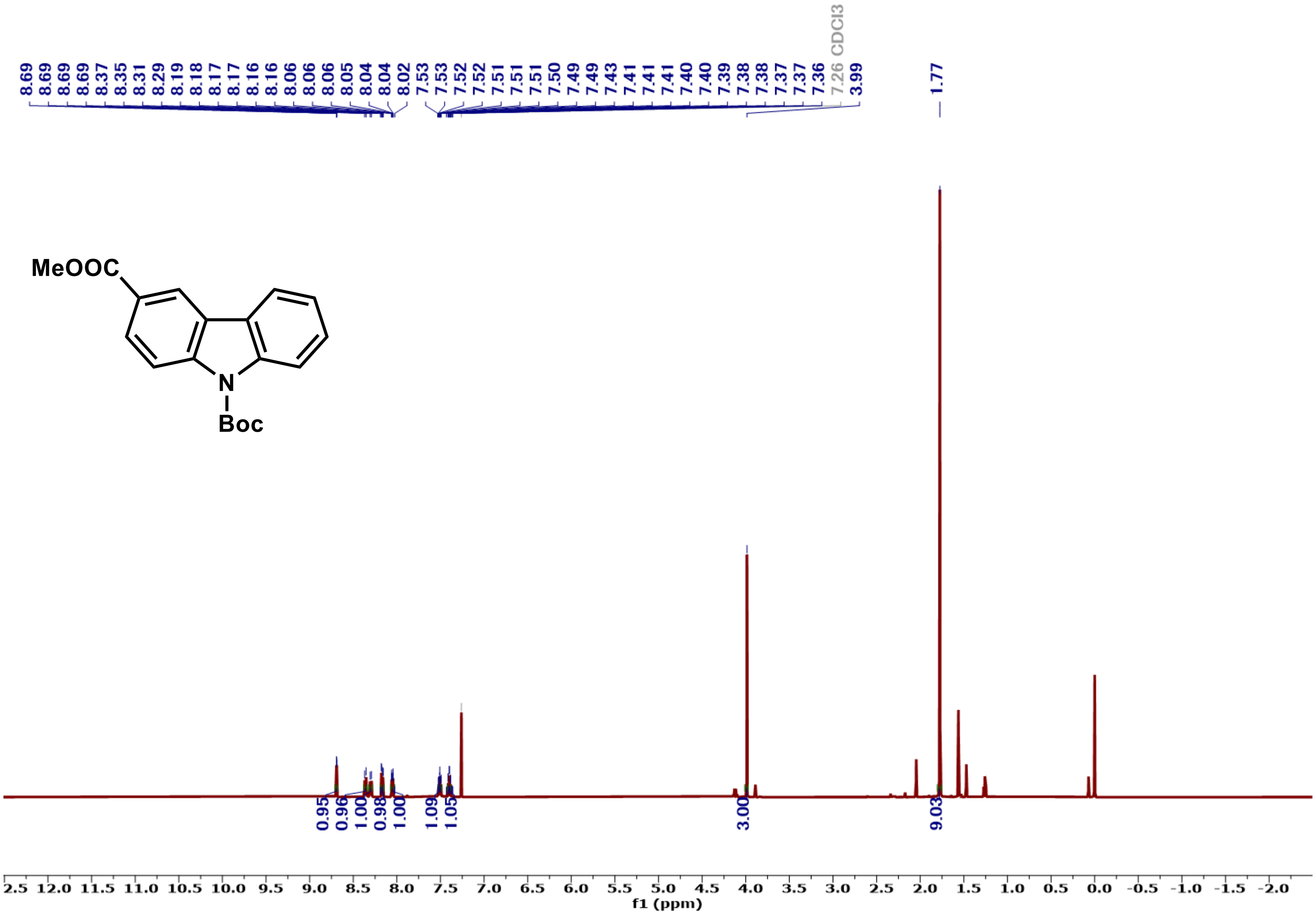

^{1}H NMR spectrum (500 MHz, $CDCl_3$, 25 °C) of **9-(*tert*-butyl) 3-methyl *9H*-carbazole-3,9-dicarboxylate (3p)**.

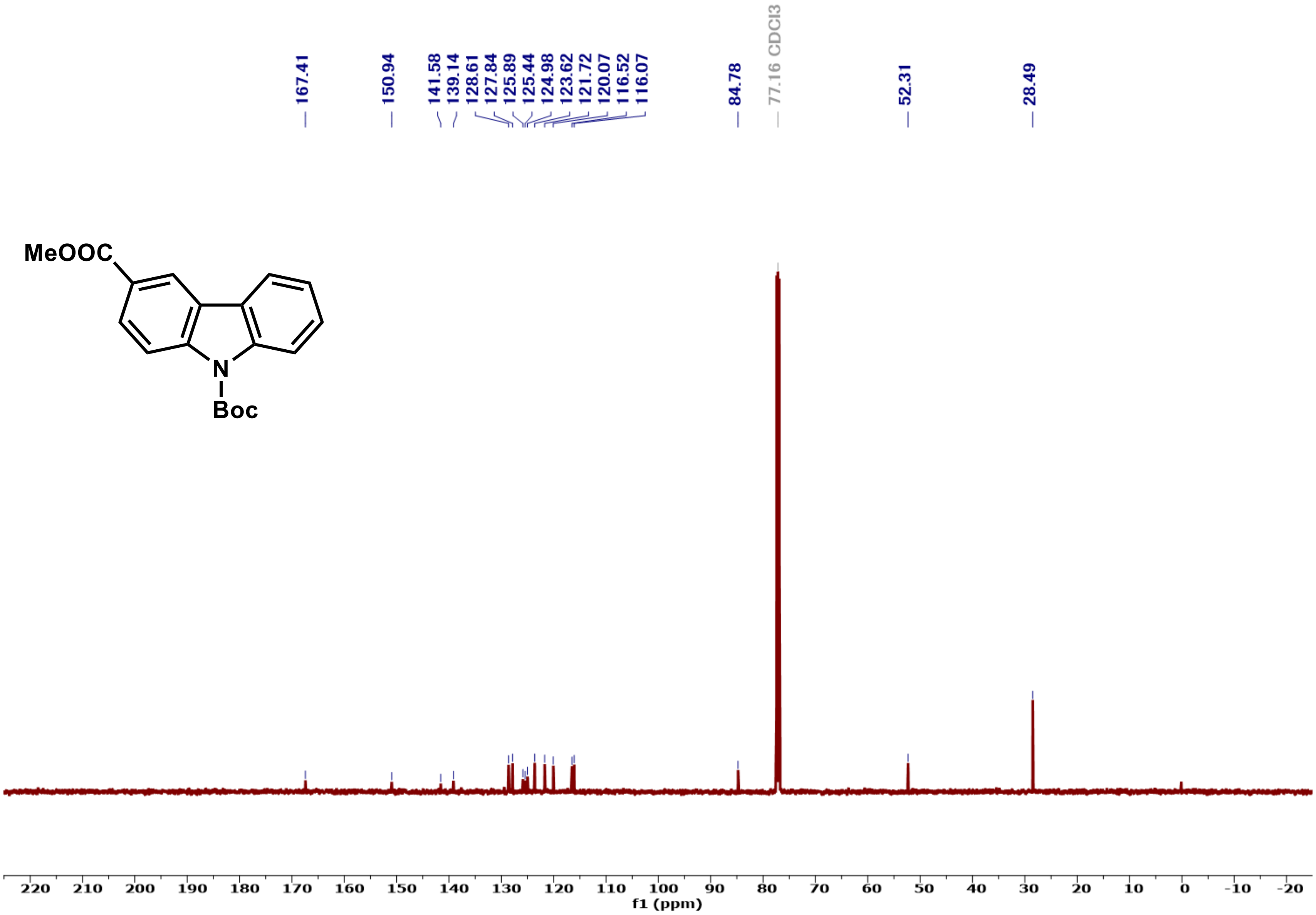

^{13}C NMR spectrum (126 MHz, $CDCl_3$, 25 °C) of **9-(*tert*-butyl) 3-methyl *9H*-carbazole-3,9-dicarboxylate (3p)**.

5. Supporting references

(1) Dolomanov, O. V.; Bourhis, L. J.; Gildea, R. J.; Howard, J. A. K.; Puschmann, H. *OLEX2*: A Complete Structure Solution, Refinement and Analysis Program. *J. Appl. Crystallogr.* **2009**, *42* (2), 339–341.

(2) Sheldrick, G. M.; Shelxtl, A. C. Sect. A: Found. Adv., 2015, 71, 3–8 CrossRef PubMed; GM Sheldrick, Shelxl. *Acta Crystallogr., Sect. C: Struct. Chem* **2015**, *71*, 3–8.

(3) Sheldrick, G. M. Crystal Structure Refinement with SHELXL. *Acta Crystallogr. C Struct. Chem.* **2015**, *71* (Pt 1), 3–8.

(4) Shapiro, N. D.; Rauniyar, V.; Hamilton, G. L.; Wu, J.; Toste, F. D. Asymmetric Additions to Dienes Catalysed by a Dithiophosphoric Acid. *Nature* **2011**, *470* (7333), 245–249.

(5) Hagiwara, K.; Akita, M.; Yoshizawa, M. An Aqueous Molecular Tube with Polyaromatic Frameworks Capable of Binding Fluorescent Dyes. *Chem. Sci.* **2015**, *6* (1), 259–263.

(6) Jana, D.; Ghorai, B. K. Side Substituent Dependence of Photophysical Properties of 9-Arylanthracene-Based π-Conjugates. *Bull. Chem. Soc. Jpn.* **2015**, *88* (1), 89–96.

(7) Yang, M.; Sheykhi, S.; Zhang, Y.; Milsmann, C.; Castellano, F. N. Low Power Threshold Photochemical Upconversion Using a Zirconium(Iv) LMCT Photosensitizer. *Chem. Sci.* **2021**, *12* (26), 9069–9077.

(8) Maity, A.; Frey, B. L.; Hoskinson, N. D.; Powers, D. C. Electrocatalytic C-N Coupling via Anodically Generated Hypervalent Iodine Intermediates. *J. Am. Chem. Soc.* **2020**, *142* (11), 4990–4995.

(9) Kao, I.-H.; Wang, C.-Y.; Chang, Y.-C.; Wu, C.-L.; Chiu, Y.-J.; Hong, F.-E. Palladium-Catalyzed Phosphination and Amination through C H Bond Functionalization on Biphenyl: Amido-Substituent as Directing Group. *Tetrahedron* **2019**, *75* (3), 387–397.

(10) Tsang, W. C. P.; Munday, R. H.; Brasche, G.; Zheng, N.; Buchwald, S. L. Palladium-Catalyzed Method for the Synthesis of Carbazoles via Tandem C-H Functionalization and C-N Bond Formation. *J. Org. Chem.* **2008**, *73* (19), 7603–7610.

(11) Laha, J. K.; Jethava, K. P.; Dayal, N. Palladium-Catalyzed Intramolecular Oxidative Coupling Involving Double C(Sp(2))-H Bonds for the Synthesis of Annulated Biaryl Sultams. *J. Org. Chem.* **2014**, *79* (17), 8010–8019.

(12) Stokes, B. J.; Jovanović, B.; Dong, H.; Richert, K. J.; Riell, R. D.; Driver, T. G. Rh(2)(II)-Catalyzed Synthesis of Carbazoles from Biaryl Azides. *J. Org. Chem.* **2009**, *74* (8), 3225–3228.

(13) Ravat, P.; Baumgarten, M. “Tschitschibabin Type Biradicals”: Benzenoid or Quinoid? *Phys. Chem. Chem. Phys.* **2015**, *17* (2), 983–991.

(14) Grunder, S.; Muñoz Torres, D.; Marquardt, C.; Błaszczyk, A.; Krupke, R.; Mayor, M. Synthesis and Optical Properties of Molecular Rods Comprising a Central Core ‐ substituted

Naphthalenediimide Chromophore for Carbon Nanotube Junctions. *European J. Org. Chem.* **2011**, *2011* (3), 478‑496.
(15) Gao, Y.; Cai, Z.; Li, S.; Li, G. Rhodium(I)-Catalyzed Aryl C-H Carboxylation of 2-Arylanilines with CO2. *Org. Lett.* **2019**, *21* (10), 3663–3669.
(16) Ma, H.; Kang, M.-S.; Xu, Q.-M.; Kim, K.-S.; Jen, A. K.-Y. Thiol-Linked Anthraquinone Anthryl Acetylene Molecule: Synthesis, Self-Assembly, and Photoelectrochemical Properties. *Chem. Mater.* **2005**, *17* (11), 2896–2903.
(17) Moorthy, J. N.; Mandal, S.; Mukhopadhyay, A.; Samanta, S. Helicity as a Steric Force: Stabilization and Helicity-Dependent Reversion of Colored o-Quinonoid Intermediates of Helical Chromenes. *J. Am. Chem. Soc.* **2013**, *135* (18), 6872–6884.
(18) Noji, T.; Fujiwara, H.; Okano, K.; Tokuyama, H. Synthesis of Substituted Indoline and Carbazole by Benzyne-Mediated Cyclization-Functionalization. *Org. Lett.* **2013**, *15* (8), 1946–1949.
(19) Cavallo, G.; Metrangolo, P.; Milani, R.; Pilati, T.; Priimagi, A.; Resnati, G.; Terraneo, G. The Halogen Bond. *Chem. Rev.* **2016**, *116* (4), 2478–2601.
(20) Oliveira, R.; Groni, S.; Fave, C.; Branca, M.; Mavré, F.; Lorcy, D.; Fourmigué, M.; Schöllhorn, B. Electrochemical Activation of a Tetrathiafulvalene Halogen Bond Donor in Solution. *Phys. Chem. Chem. Phys.* **2016**, *18* (23), 15867–15873.
(21) Tepper, R.; Schulze, B.; Jäger, M.; Friebe, C.; Scharf, D. H.; Görls, H.; Schubert, U. S. Anion Receptors Based on Halogen Bonding with Halo-1,2,3-Triazoliums. *J. Org. Chem.* **2015**, *80* (6), 3139–3150.
(22) Hein, R.; Docker, A.; Davis, J. J.; Beer, P. D. Redox-Switchable Chalcogen Bonding for Anion Recognition and Sensing. *J. Am. Chem. Soc.* **2022**, *144* (19), 8827–8836.
(23) Costentin, C.; Savéant, J.-M. Multielectron, Multistep Molecular Catalysis of Electrochemical Reactions: Benchmarking of Homogeneous Catalysts. *ChemElectroChem* **2014**, *1* (7), 1226–1236.
(24) Costentin, C.; Drouet, S.; Robert, M.; Savéant, J.-M. Turnover Numbers, Turnover Frequencies, and Overpotential in Molecular Catalysis of Electrochemical Reactions. Cyclic Voltammetry and Preparative-Scale Electrolysis. *J. Am. Chem. Soc.* **2012**, *134* (27), 11235–11242.
